# Tone and genes: New cross-linguistic data and methods support the weak negative effect of the “derived” allele of *ASPM* on tone, but not of *Microcephalin*

**DOI:** 10.1371/journal.pone.0253546

**Published:** 2021-06-30

**Authors:** Dan Dediu

**Affiliations:** Laboratoire Dynamique Du Language (DDL) UMR5596, Université Lumière Lyon 2, Lyon, France; Girne American University - Karmi Campus: Girne Amerikan Universitesi, CYPRUS

## Abstract

While it is generally accepted that language and speech have genetic foundations, and that the widespread inter-individual variation observed in many of their aspects is partly driven by variation in genes, it is much less clear if differences between languages may also be partly rooted in our genes. One such proposal is that the population frequencies of the so-called “derived” alleles of two genes involved in brain growth and development, *ASPM* and *Microcephalin*, are related to the probability of speaking a tone language or not. The original study introducing this proposal used a cross-linguistic statistical approach, showing that these associations are “special” when compared with many other possible relationships between genetic variants and linguistic features. Recent experimental evidence supports strongly a negative effect of the “derived” allele of *ASPM* on tone perception and/or processing within individuals, but failed to find any effect for *Microcephalin*. Motivated by these experimental findings, I conduct here a cross-linguistic statistical test, using a larger and updated dataset of 175 samples from 129 unique (meta)populations, and a battery of methods including mixed-effects regression (Bayesian and maximum-likelihood), mediation and path analysis, decision trees and random forests, using permutations and restricted sampling to control for the confounding effects of genealogy (language families) and contact (macroareas). Overall, the results support a negative weak effect of *ASPM*-D against the presence of tone above and beyond the strong confounding influences of genealogy and contact, but they suggest that the original association between tone and *MCPH1* might have been a false positive, explained by differences between populations and languages within and outside Africa. Thus, these cross-linguistic population-scale statistical results are fully consonant with the inter-individual-level experimental results, and suggest that the observed linguistic diversity may be, at least in some cases, partly driven by genetic diversity.

## Introduction

It is becoming increasingly accepted that, in order to fully understand language, its origins, evolution, change, and patterns of diversity, we need to re-root it into its wider environment [1–3]. This comprises not only climate [3], altitude [4] and ecology [5], but also the biology of the speakers [1]. While the *universal* effects of the species-wide shared properties of our perception, processing and production of language have a long history of intense study [6–8], much less attention has been given to *inter-individual variation* and its influences on the emergence of the observed patterns of linguistic diversity [1].

One of the first well-supported proposals linking the biological and linguistic diversities is represented by [9], which suggested that the cross-linguistic distribution of *linguistic tone* is partly explained by the population frequency of certain genetic variants (or *alleles*) of two genes involved in brain growth and development, *ASPM* and *Microcephalin*. The evidence in support of this suggestion consisted of a set of statistical analyses, showing that, indeed, after controlling for the effects of shared history and contact, these alleles have a weak negative effect on the probability that a language is a tone language. It was further speculated that this is due to a negative *bias* that is very weak at the individual level (as any normal child can acquire perfectly the phonological system of any human language she has proper exposure to), but that can be *amplified* by the repeated transmission of language across generations in populations of speakers with similar biases [9–11]. Because the frequency of these alleles varies between populations, the presence and/or strength of this bias should vary as well, helping explain why tone languages are distributed the way they are, but, emphatically, this is just one weak explanatory factor among many others, easily overwritten by other, much stronger forces, such as language contact and language-internal developments [12–14] or even climate [3].

However, this proposal has been greeted with a certain scepticism (with some exceptions; e.g., [15]) due to several factors, one of the most important being, as detailed below, methodological. This resulted in the perception that, most probably, the effect of the two alleles on tone was, at best, a false positive, an artefactual result of the inadequacy of the data and of the incapacity of the statistical techniques to effectively control for confounds, or, at worst, yet another case of “double-dipping” (or “circular analysis”) in which the same data is used to generate a hypothesis and then to test it [16]. Nevertheless, as emphasized in the original publication and in several subsequent ones [9–11], this study should be seen as *generating* hypotheses while trying to reduce the probability of false positives, using the best available data and methods.

During the intervening years, various approaches to testing this hypothesis have been tried. For example, in [17, 18] I used computer simulations of different implementations of such a genetic bias (both Bayesian and “ad-hoc”) in different types of settings (simple transmission chains, transmission chains with two agents per generation, and complex societies) to show that, under certain conditions, weak biases rooted in genetics can be amplified by the repeated transmission of language to produce correlations similar to those observed between tone and the two alleles. If tone was affected by the genetic structure of the population, and given that, in general, allele frequencies change much slower than language, then we could predict that tone should tend to be quite stable. Applying Bayesian phylogenetic methods from evolutionary biology to language structures, [19] found that, indeed, tone tends to be among the most stable features of language—a finding supported by other, sometimes widely different, methods [20, 21].

But probably the most convincing type of test for this hypothesis is represented by an *experimental* design, whereby a link can be found between the genome of an individual and her performance related to linguistic tone. (There are other types of evidence, involving for example, animal models or molecular genetics, but these are still far in the future). However, besides the high costs and the logistics of such designs, the fundamental issue is the *operationalization* of this bias in terms of actual psycholinguistic or neuro-cognitive tasks which have high reliability, show enough inter-individual variation, and are arguably related to the learning, perception, processing or production of linguistic tone. Several such attempts were made, including the perception of missing fundamental tones [22], the use of pitch variation in word segmentation [23], and the learning of an artificial tone language in an fMRI paradigm [24]—but while all these produced interesting results, they have arguably failed to shed much light on the nature of the bias.

While these experimental approaches were purely at the behavioural or neuro-cognitive levels, without any genetic component, [25] took a completely different route. They recruited a total of 32 young adults, native speakers of American English and self-identified “Caucasians”. These participants were genotyped for the alleles of interest for the two genes, *ASPM* and *Microcephalin*, and performed a set of behavioural and neuro-cognitive tasks in order to test if (and in what way) their genotype predicted their behavioural and/or neural responses after controlling for various confounds (age, sex, auditory working memory, and phonemic awareness). In a nutshell, the behavioural task of interest was a “tone perception task” where the participant heard a resynthesized Mandarin vowel with one of three Mandarin tones superimposed. The participant had to indicate which way the pitch was going, by selecting the correct arrow among two shown on the screen (→ for Mandarin tone 1 level, ↗ for tone 2 rising, and ↘ for tone 4 falling). 13 of these participants also took part in an fMRI adaptation experiment, which looked at how the brain’s response “adapts” to the repeated presentation of the same Mandarin tone. Despite being relatively underpowered, this experiment did find an effect of *ASPM* on the “tone perception task”, but apparently in the opposite direction to that predicted by [9], and failed to find any effect for *Microcephalin*. The opposite sign of *ASPM*’s effect could be due to several factors, including the genetic background of the participants and the fact that they did not speak a tone language [25], but most probably it is because the “tone perception task”, instead of operationalising what the native speakers of a tone language such as Mandarin Chinese do, captures what speakers of an intonation language (such as English) do, namely separate the pitch contour from the segments [23, p. 340]—if this is the case, then [25]’s results are actually precisely in the direction predicted by [9].

Building on this work, [26] recruited a massive sample of 426 native speakers of Cantonese (a language with a complex tone system), mostly from Hong Kong. All these participants were genotyped not only for the two alleles of interest for *ASPM* and *Microcephalin*, but for a further 20 more variants in a total of 10 genes that have been involved in the brain, cognition, speech and language (*CDK5RAP2*, *COMT*, *DRD1*, *DRD2*, *CNTNAP2*, *ATP2C2*, *CMIP* and *FOXP2*). After a hearing test, they provided the number of years of musical training, performed a test of non-verbal intelligence, and four experimental tasks: “lexical tone perception” (an ABX task matching the last tone to the first or the second), “musical pitch perception” (judging if pairs of short melodies were identical or different in one note), “rhythm perception” (as above, but the difference was in rhythm), and a working memory task. Among all the possible associations between the genetic variants and the measures, only that between “lexical tone perception” and *ASPM* was significant, even after controlling for non-verbal IQ and years of musical training; moreover, the effect was in the direction predicted by [9], and its effect size was compatible with a weak bias and with other known genetic effects [26].

Therefore, taken together, [25] and especially [26], seem to suggest that the cross-linguistic effect of *ASPM* on linguistic tone, proposed by the earlier exploratory study in [9], may have an individual basis. Citing previous work, [26] suggests that this is mediated by *ASPM*’s effects on the structure of the auditory cortex (including Heschl’s gyrus), influencing thus the perception and processing of pitch. Thus, we could conclude that, while not the last word on the matter, this may be a canonical example of a hypothesis-generating exploratory study leading, more than 10 years later, to an experimental hypothesis-testing design, supporting the initial proposal using completely different data and methods. However, this scientific success story generated two nagging questions for me: (i) what is going on with *Microcephalin*?, and (ii) the data, methods and results in [9] are from 2005–2006: how would they look in 2020?

For (i), both experimental studies [25, 26] fail to find any evidence for an effect of *Microcephalin* and, while this could be a false negative (its effect is too weak to be detected even with more than 400 participants) or simply not captured by the tasks, it is worthwhile taking it at face value and assuming that *Microcephalin* could have very well been a false positive in the original [9] study. For (ii), the intervening years have seen a revolution in the methods used to ask cross-cultural questions [27], ranging from the generalisation of mixed-effects/hierarchical regression [28, 29], to the use of Bayesian methods [2, 30], permutation/randomisation [31], and of phylogenetics [32] and machine learning [33]. Likewise, the availability and quality of linguistic (and cultural) data has dramatically improved, with databases such as *WALS Online*; [34], *PHOIBLE*; [35], *LAPSyD* and *D-PLACE* [36] being easily accessed by humans and machines. On the genetic side, while the genomic coverage of the data has exploded (we now have not only full exomes and genomes, but epigenetic data as well), both in modern and archaic humans (including Neanderthals and Denisovans), it has remained rather circumscribed geographically and ethno-linguistically, despite efforts such as the *1000 genomes project* [37], the *Simons Genome Diversity Project* [38], and the *The ALlele FREquency Database* (*ALFRED*) [39]. So, if we were to collect new linguistic and genetic data, and use the methods now available, how would the relationship between *ASPM*, *Microcephalin* and tone look like?

This paper first summarises the original data, methods and results in [9], then describes the new data collected (as of early 2020), the methods used and their results, ending with a discussion and conclusions.

## A summary of the original 2007 study

In this section, I briefly review and summarize the 2007 study [9] that originally introduced the hypothesis of a relationship between tone, *ASPM* and *Microcephalin*. (To facilitate reading, I prefix the titles of this section’s subsections with “2007:”).

### 2007: Two papers in *Science*

**Fig 1:**
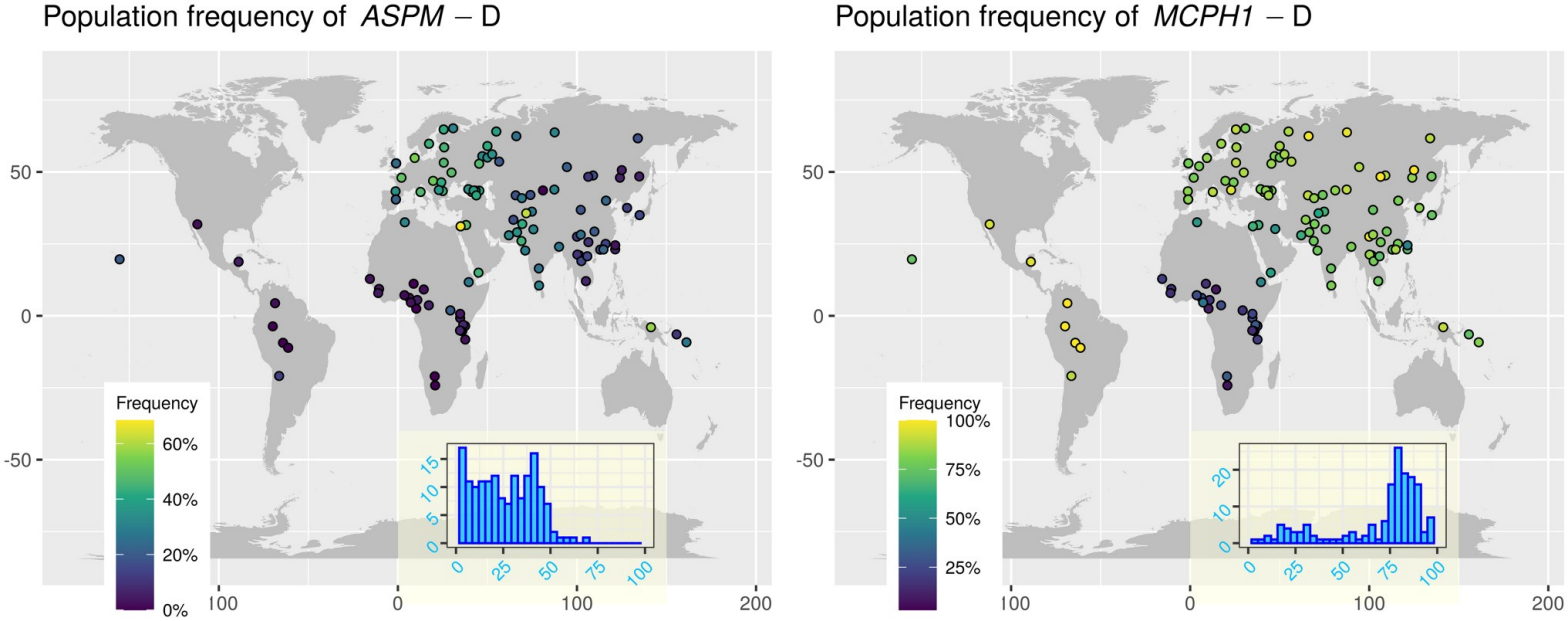
The distribution of the two “derived” alleles in the newer data. Left: *ASPM*-D, right: *MCPH1*-D. Each circle on the maps represents a unique sample, and its colour represents the frequency (%) of the “derived” allele in that sample. The inset shows the overall histogram of the allele frequency across all the samples. Please note that the maximum percentage (corresponding to the lightest colour) differs between the two panels. The maps were generated with the R package maps which uses public domain data from the Natural Earth project https://www.naturalearthdata.com/.

In 2005, two papers from the same research group were published in the same issue of *Science*, each detailing the story of one of two genes: *ASPM* [40] and *Microcephalin* (or *MCPH1*; [41]). These two genes are involved in brain growth and development, as shown by the fact that several mutations result in *microcephaly* [42], and they seem to have played an important role in the evolution of the brain [43–45]. However, the two *Science* papers focused on variants that are *not* involved in microcephaly or, in general, in any other pathology [40, 41], but instead seem to be part of the range of normal genetic variation in our species [46]. These so-called “derived” variants (or *alleles*) of the two genes (henceforth denoted as the “derived” alleles, or as *ASPM*-D and *MCPH1*-D, respectively) are characterised each by a change in the sequence of its respective gene, change that is relatively recent and contrasting with the “ancestral” version from which they derive. The papers estimated that *ASPM*-D emerged some 5,800 years ago (95% confidence interval between 14,100 and 500 years ago), and *MCPH1*-D some 37,000 years ago (between 60,000 and 14,000 years ago). However, what was truly striking about these alleles was the geographic distribution of their population frequency; please see [40, Fig 1., p. 1721] and [41, Fig 3., p. 1719] for the original maps, and Fig 1 for maps using newer data. As a reminder, the vast majority of locations on our genome (or *loci*), including genes such as *ASPM* and *MCPH1*, come in pairs, with one copy inherited from the mother and one from the father. Thus, in simple cases as discussed here, each individual can have 0, 1 or 2 “derived” alleles in her genome—independently for each of the two genes. In a population (or group) of people, we can thus compute the frequency of the “derived” allele (for each gene independently), *f*_*D*_, by diving the number of “derived” alleles across individuals, *N*_*D*_, to twice the total number of individuals genotyped in the population, 2*N* (as each individual can have up to two such alleles): $f_{D}=\frac{N_{D}}{2N}$. This can vary between 0% (the “derived” allele is absent from the population) and 100% (everybody has it) but, importantly, its estimation can be affected by many types of errors and biases, the most important being the number of people that are genotyped, the genealogical relationships between them, and their representativity for the considered (meta)population. (Please see [46] for a gentle introduction to population and evolutionary genetics). Also, while not directly relevant to the hypothesis in [9], it is nevertheless important to mention that the original papers claimed that (i) the “derived” alleles of both *ASPM* and *Microcephalin* were evolving under positive natural selection (i.e., that there is a selective advantage to the individuals having them in their genomes), and (ii) that this selective advantage was probably related to “cognition”. However, the method they used to test for positive selection is not robust, and the selection signal is probably an artefact [47, 48]. More importantly, given the controversies the original papers generated (including accusations of implicit racism) [49], subsequent work did not find any influence of these “derived” alleles on cognition, brain size or any other such phenotypes [50, 51] (with some debatable exceptions; [52, 53]). Therefore, while the crucial roles played by these two *genes* in the evolution and ontogeny of the human brain are clear, with very probable episodes of positive selection in our lineage [43–45], it is currently unclear what phenotypic effects their *“derived” alleles* may have [26, 54] and odds are that the recent and current evolution of these particular alleles is mainly driven by neutral demographic processes and not by selective pressures [47, 48, 54].

### 2007: The hypothesis

There were three factors that prompted us to explore the relationship between these two “derived” alleles and linguistic tone back in 2005–2006:

1. the striking visual resemblance between the maps of the two “derived” alleles (Fig 1) and that of linguistic tone (Fig 3 and https://wals.info/feature/13A)
2. the probable involvement of the two genes in brain growth and development, and
3. the evidence of a genetic basis for language and speech was rapidly increasing, not only in terms of heritability studies [55], but also of candidate genes [56], including the relatively recently discovered *FOXP2* [57]; while most of these concerned “universal” aspects of speech and language or their pathologies, a few did focus on normal inter-individual variation (see [58] for a contemporary review and discussion).

Thus, while the suggestion that these two “derived” alleles influence tone was *not* an *a priori* hypothesis that pre-existed seeing the data, but instead was prompted by the data itself, it did nevertheless have pre-existing theoretical roots, especially in the prediction that, given their genetic bases and the widespread inter-individual and inter-group genetic variation, there should be aspects of language and speech whose patterns of diversity are influenced by genetic diversity [58, 59]. Therefore, it is important to see [9] as an attempt to *reject* the hypothesis of a link between the geographic distributions of *ASPM*-D, *MCPH1*-D and tone, using a super-set of the data partly responsible for the generation of this hypothesis. The failure to achieve this rejection can be taken, *in extremis* [60], as a confirmation that human visual perception is really good at detecting matching patterns and that [9] is a futile and misleading exercise in “dubble dipping”/”circular analysis”, or, as repeatedly highlighted [9, 10, 58], as the generation of a hypothesis from a combination of data and theoretical expectations, followed by the reduction of the probability of false positives.

### 2007: The data

In order to check this hypothesis, we started from the genetic data in [41, 40], consisting of the frequency of the two “derived” alleles in 59 populations, relatively widespread across the globe, but with a clear bias towards Africa and Eurasia, and very little data from the Americas and the Pacific, and no data from Australia. We manually mapped these populations to languages (using the meta-data in the two papers), and we collected data concerning not only their tone systems (using a binary coding of “no tone” versus “tone”) but also various other structural aspects of language (mostly from [61], supplemented with data from other secondary and primary sources, including questionnaires sent to specialists in various languages); likewise, we collected data about the frequency of many more genetic loci spread across the genome in these populations from databases such as *ALFRED* [39] and *HGDP* [62] (see [9, 58] for details). Due to missing and ambiguous data, we included only 49 of these 59 populations in our analyses (in particular, we did not include the 5 populations from the Americas in the statistical analyses, but we used them as an informal test of the results, in the sense that they have low frequencies of *ASPM*-D, high of *MCPH1*-D, and show both tonal and nontonal languages).

### 2007: The methods

With these, we estimated the association between tone and the population frequencies of *ASPM*-D and *MCPH1*-D either individually, using Pearson’s *r* correlation coefficients, and together, using logistic regression. Besides evaluating the effect sizes and the statistical significance of these measures of association as such, we also compared their effect sizes against those of all possible associations between all the linguistic features and all the genetic markers in our database. This procedure quantifies “how special” the relationship between tone and the two “derived” alleles is relative to what would be expected when repeatedly picking random aspects of language and our genome, allowing us to *implicitly* control for multiple confounds, such as climate, environment, contact, language family diversification, and past demographic processes and events.

On top of this, we also *explicitly* controlled for two major confounds [27]: shared inheritance and contact. The first refers to the fact that the features of related languages are not independent due to inheritance from their common ancestor (“Galton’s problem”; [63]), a point also applicable to the genetic makeup of populations that descend from a common ancestor. The second captures the fact that languages in contact tend to exchange features, just as populations may exchange genes. For this explicit control, we used the *Mantel test* [64], which computes the (partial) correlations between two distance matrices (possibly controlling for others), and repeatedly permutes the data in order to compensate for the non-independence of the observations. The distances used were (the first two are of interest for the hypothesis, the last two are the confounds):

- the “structural distance” between languages, defined as the Euclidean distance on the space defined by one or more structural features: small distances reflect higher structural similarities between the languages
- the “genetic distance” between populations, defined as Nei’s *D* [65]: small distances mean that the populations have very similar allele frequencies
- the “geographic distance” between languages, computed as the great circle distances constrained by the geography of the continents: presumably languages closer in space would have had more chances for linguistic and genetic exchanges, and
- the “historical linguistic distance”, quantifies the degree of genealogical closeness between two languages using the classification in the 15^th^ edition of the *Ethnologue* [66].

### 2007: The main results

The Pearson correlations between tone and *ASPM*-D (*r* = −0.53, *p* = 9.63 ⋅ 10^−5^) and *MCPH1*-D (*r* = −0.54, *p* = 7.22 ⋅ 10^−5^) are not only highly significant, but also stronger than most (> 98.5%) of all the possible 25,558 such correlations. Likewise, the logistic regression of tone on both *ASPM*-D and *MCPH1*-D simultaneously was very good (Nagelkerke *R*^2^ = 52.8%, *β*_*ASPM*−*D*_ = −7.2, *p* = 0.010, *β*_*MCPH*1−*D*_ = −4.9, *p* = 0.026) and better than 97.3% of all the 11,582,690 possible such logistic regressions.

Turning to the Mantel correlations: *r* = 0.17, *p* = 0.015 for tone–geography, *r* = 0.07, *p* = 1.0 for *ASPM*-D–geography, *r* = 0.54, *p* < 0.001 for *MCPH1*-D–geography; *r* = 0.33, *p* < 0.001 for tone–(*ASPM*-D, *MCPH1*-D), *r* = 0.29, *p* = 0.003 for tone–(*ASPM*-D, *MCPH1*-D) while controlling for geography, and *r* = 0.28, *p* < 0.001 for tone–(*ASPM*-D, *MCPH1*-D) while controlling for geography and history.

### 2007: Potential issues

However, besides the nature of the hypothesis and its testing discussed above, there are several potential issues with the data and the methods. Concerning the data, first, the main constraint was the availability of frequency information for the “derived” alleles, limiting the study to the skewed and rather small sample in [41, 40]. Second, the identification of some samples was far from unambiguous, resulting in uncertain judgements with respect to the linguistic variables (including tone).

While the comparison with the empirical distribution of many other similar associations should control for many potential confounds, the technique used for their explicit control, namely the Mantel test, is far from ideal. First, it does not quantify the association between the actual values, but between distances derived from these values, introducing extra degrees of freedom and potential noise. For example, the way geographical distance is computed assumes a linear effect on contact, and the historical linguistic distance assumes that unrelated languages are only slightly more dissimilar than languages from the same family but in different “genera” (a distance of 4 versus 3). Second, controlling for the historical and geographical distances does not fully model their effects on the relationship between tone and the two “derived” alleles. For these (and other) reasons, the Mantel test is very rarely used nowadays, the preference being to explicitly model the sources of statistical non-independence using, for example, phylogenetic approaches, mixed effects/hierarchical models, permutation/randomisation or restricted sampling.

I now turn to the current study, detailing its data, methods and results.

## Data

Please note that all the data, and the R and Rmarkdown code need to reproduce the results reported here, as well as the full analysis report (as a self-contained HTML document) are freely available on Zenodo at doi:10.5281/zenodo.4762169 and (except the HTML full analysis report, due to its size) also in the GitHub repository https://github.com/ddediu/tone-genes-update. All external file paths referenced here are relative to the root of this repository (e.g., ./data/genetics/code/00_preprocesses_genetics.R).

Despite the advances of the last 14 years, the availability of frequency data for the “derived” alleles of *ASPM* and *MCPH1* is still the limiting factor for this update. Therefore, I first collected (hopefully, all) the currently available genetic data, followed by its merging with the linguistic data. Please note that I focus here only on the relationship between tone and the two “derived” alleles, and not on its comparison with other comparable relationships as in the original paper [9]; therefore, I only collected and analysed data on *ASPM*-D, *MCPH1*-D and tone.

### The “derived” alleles

#### Definition

The original paper [40, p. 1720] identified the “derived” allele of *ASPM* in relation to “haplotype 63” and two of its polymorphic nonsynonymous sites in exon 18 in an open reading frame (ORF), A44871G and C45126A, with ancestral alleles A and C, respectively, and the derived ones, G and A. More recent publications however, use SNP (single nucleotide polymorphism) *rs41310927* with ancestral allele T and derived allele C. Likewise, the original paper [41, p. 1717] identified the “derived” allele of *MCPH1* (or *Microcephalin*) in relation to G37995C in exon 8 in an ORF with ancestral allele G and derived one C. More recent publications use SNP *rs930557* with ancestral allele G and derived allele C.

#### Data sources

For the collection of population frequency data concerning the two “derived” alleles, I used a total of 7 sources (see Table 1): besides the original papers [41, 40], I extracted information from the experimental study [26] on Cantonese speakers, as well as from several large genetic databases. However, it is important to note that, while most databases contain information about the actual “derived” alleles or the corresponding SNPs (*rs41310927* and *rs930557*), not all do, but instead contain data on other SNPs that are in very tight LD (linkage disequilibrium) with them. I used *LDlink*’s “LDproxy Tool” to obtain the list of all SNPs in LD with the target ones across all the populations in that database—see Table 2 for the list of retained “proxy” SNPs. For *ASPM*-D, these 5 “proxy” SNPs represent 289 unique extra genetic samples out of 396 (73%), and for *MCPH1*-D, the single “proxy” SNPs represents 141 unique extra genetic samples out of 245 (57.6%). Please note that, while increasing the coverage of the data, this procedure is not perfect, in that LD may differ between populations and geographic regions, potentially introducing noise. However, running the analyses excluding the “proxy” SNPs produce very similar, but somewhat weaker results to those obtained including them (see the full HTML analysis report), suggesting that they are, indeed, good proxies for the “derived” alleles.

**Table 1:** Data sources used for estimating the population frequency of the “derived” alleles, including the number of samples/populations (column “#”) for which such data was available.

Source	URL	Description	#
[40]	https://science.sciencemag.org/content/309/5741/1720	original source for *ASPM*-D	59
[41]	https://science.sciencemag.org/content/309/5741/1717	original source for *MCPH1*-D	59
[26]	https://advances.sciencemag.org/content/6/22/eaba5090	> 400 Cantonese speakers	1
*LDLink*	https://ldlink.nci.nih.gov/?tab=home	“proxy” SNPs and frequency data	26
*gnomAD*	https://gnomad.broadinstitute.org	v2.1.1; very broad populations	7
*dbSNP*	https://www.ncbi.nlm.nih.gov/snp	info form multiple databases	15
*1000 genomes*	https://www.internationalgenome.org	info included in *gnomAD*	-
*ALFRED*	https://alfred.med.yale.edu/alfred/index.asp	many populations and samples	141

**Table 2:** The original loci and their “proxy” SNPs, with their “derived” allele and the data sources (with the number of unique samples/populations) from which frequency information was obtained.

Target	Proxy	“D” allele	Position and LD	Data sources (#)
*ASPM*-D	haplotype 63	“D”	the target	[40] (59)
*ASPM*-D	*rs41310927*	C	the target	[26] (1), *LDLink* (26), *gnomAD* (7), *dbSNP* (14)
*ASPM*-D	*rs41308365*	A	1:197070707; 1.0, 1.0	*LDLink* (26), *gnomAD* (7), *dbSNP* (2)
*ASPM*-D	*rs3762271*	T	1:197070442; 1.0, 1.0	*LDLink* (1), *gnomAD* (7), *dbSNP* (14), *ALFRED* (141)
*ASPM*-D	*rs41304071*	T	1:197063352; 1.0, 1.0	*LDLink* (26), *dbSNP* (7)
*ASPM*-D	*rs147068597*	A	1:197058136; 1.0, 1.0	*LDLink* (26)
*ASPM*-D	*rs61819087*	G	1:197084857; 1.0, 1.0	*LDLink* (26), *dbSNP* (6)
*MCPH1*-D	G37995C	C	the target	[41] (59)
*MCPH1*-D	*rs930557*	C	the target	[26] (1), *LDLink* (32), *dbSNP* (18)
*MCPH1*-D	*rs1129706*	G	8:6304814; 0.99, 0.94	*ALFRED* (141)

Position is given as chromosome:location. LD is given as *D*′ and *R*^2^.

#### The samples and (meta)populations

The unit (the “group”) for which allele frequency information is given varies between, and even within, these data sources, and cannot be considered *a priori* equivalent nor unique between and within data sources. For example, *1000 genomes* (and *LDLink*) contain populations such as “African/African-American”, “Han Chinese in Beijing, China” and “Mende in Sierra Leone”, while *ALFRED* may contain very specific samples from arguably the same (meta)populations, such as SA004380P and SA004595X, both “Khanty”. Thus, I manually matched these units within and between samples using the meta-information available in each database, resulting in 175 unique *samples* contained in 129 unique *(meta)populations*, where possible based on those available in *ALFRED*; each sample has a unique ID, while for each (meta)population, besides a unique ID, I also provide a “readable” name. With these, a (meta)population may contain one or more samples: e.g., “Abkhaz” [PO000844Q] contains just the *ALFRED* sample SA004584V, while “Adygei” [PO000017I] contains three samples, SA001509P, SA004373R and SA004585W. The meta-information used for matching samples and (meta)populations is contained in the TAB-separated file ./data/genetics/input/populations.tsv, and is used in the pre-processing of the genetic data by the R script ./data/genetics/code/00_preprocesses_genetics.R. For *ASPM*-D, I added 111 new samples representing 84 unique (meta)populations, while for *MCPH1*-D, there are 107 new samples from 85 unique (meta)populations (compared to the original 59 samples from 56 (meta)populations); the distribution of the original and new samples and (meta)populations is given in Table 3, while the full HTML analysis reports also plots their maps.

**Table 3:** The original genetic samples and (meta)populations (in parentheses) used in [9], and the new ones added in this study, by macroarea.

Study	Gene	Africa	Eurasia	America	Papunesia	Total
original [9]	both	15 (14)	37 (35)	5 (5)	2 (2)	59 (56)
new to this study	*ASPM*	12 (12)	90 (63)	5 (5)	4 (4)	111 (84)
new to this study	*MCPH1*	12 (12)	86 (64)	5 (5)	4 (4)	107 (85)

#### The frequencies of the “derived” alleles

For each unique sample there might be frequency information available for more than one locus; for example, for *ASPM*-D in the “Adygei” [PO000017I] sample SA001509P, there is information for *A44871G* from [40] (frequency *f* = 0.40 from *N* = 30 alleles) and for *rs3762271* from *ALFRED* (*f* = 0.41, *N* = 34). In such cases, I computed the *weighted average frequency* as in Eq 1:

$$\begin{array}{c} wavg=\frac{\sum_{i}f_{i}\cdot N_{i}}{\sum_{i}N_{i}}, \end{array}$$ (1)

where *i* goes over all data sources with relevant information, *f*_*i*_ is the allele frequency and *N*_*i*_ the number of genotyped alleles (normally twice the number of genotyped individuals) in the data source *i* (in the example above, *wavg*(SA001509P) ≈ 0.405). While this procedure does not assume subjective preferences between data sources, nor does it take into account the potentially imperfect LD between some of the “proxy” loci and the “target”, it does give more credence to larger samples, resulting in an aggregate frequency estimate that should be more robust than each estimate independently.

#### The distribution of the “derived” alleles

The patterns of distribution remain similar to those in the original *Science* papers (Fig 1). The frequency of *ASPM*-D is globally below ≈60%, is very low in sub-equatorial Africa and the Americas, higher in eastern Eurasia, and highest in western Eurasia. *MCPH1*-D is almost absent in sub-equatorial Africa and relatively high everywhere else, reaching fixation (100%) in some samples.

### Tone

#### Matching samples to languages

In most cases, the mapping of a given genetic sample to one (or more) language(s), using the provided metadata, is far from obvious. To this end, I used all the available information about the sample in the data source giving the allele frequency, in combination with other sources such as *Wikipedia*, the *Ethnologue*, the *Glottolog*, *WALS*, and the *ISO 639–3 Registration Authority* website, plus general knowledge about the distribution of the world’s languages and ethnic groups. (For example, the description of the population “Adygei” [PO000017I] in *ALFRED*, available at https://alfred.med.yale.edu/alfred/recordinfo.asp?UNID=PO000017I, gives general data about the population, including links to the *Ethnologue*, as well as more specific information about each particular sample, possibly including references to the actual publications). While this resulted in clear matches in many cases, with 139 samples (80%) being uniquely matched to a single language (e.g., the samples of the “Adygei” [PO000017I] population were uniquely matched to the *Adyghe* language, Glottocode adyg1241, ISO-639–3 ady), and 23 samples to 2 languages each (e.g., the “Bakola Pygmy” were mapped to either/both *Gyele* [gyel1242, gyi] and *Kwasio* [kwas1243, nmg]), there were also more ambiguous mappings: 4 samples mapped to 3 languages each, 4 to 3, 1 each to 5, 6, 7 and 9 languages, 1 to 23 languages (the *ALFRED*/*HGDP* sample SA001501H “Papuan”, where the best I could do was to map it to a whole set of potential *Sepik*, *Ndu*, *Ap Ma*, and *Lower Speik-Ramu* languages), and 1 mapping to no less than 144 languages (this “monster” is the SA004382R “Micronesians” sample, which I could only map to a whole subset of the *Austronesian* family).

As this ambiguous mapping may introduce an extra level of uncertainty, for an extra analysis, I removed all the genetic samples that do not uniquely map to a single language. Please note that this procedure is very conservative, given that several “ambiguous” mappings are in fact concordant for tone, as is the case, for example, for *Church Slavic* [chur1257, chu] and *Russian* [russ1263, rus] which correspond to sample SA004603N, or for *Modern Hebrew* [hebr1245, heb], *South Levantine Arabic* [sout3123, ajp] and *Standard Arabic* [stan1318, arb], which correspond to SA004371P. The full results are reported in the HTML analysis report, but they are very similar to those obtained using the whole set of genetic samples, suggesting that using all the available information is worth the cost of added noise.

#### Data sources

**Fig 2:**
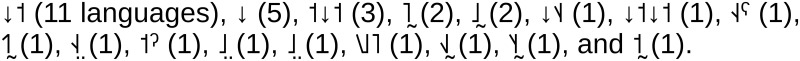
The tone symbols in *PHOIBLE* that were removed because they appeared too rarely in the database (with the number of occurrences in parentheses).

I collected data from five sources (see Table 4). I used the 2014 version of the *WALS Online* [34], which gives tone as a 3-way classification (“Feature 13A”) in a machine-readable format (CSV). I manually collected the tone data from the *LAPSyD* website in early 2020, noting, for each language, the 5-way classification and the given number of tones. The binary classification of tone (present/absent) used in [9] is available in Annex 6 of my PhD thesis [58]; it was manually curated from several primary and secondary sources (see [9, 58] for details). For *PHOIBLE* [35], I downloaded the 2.0.1 version of the database and I extracted only the symbols used for tone (SegmentClass = “tone”), manually removing some symbols that appear only very rarely (see Fig 2). Unfortunately, the data of *The World Phonotactics Database* ([67]; *WPHON*) was not accessible as of April 2020, so I used instead the last available snapshot from the Internet Archive/the WayBackMachine from 8 June 2019, available at https://web.archive.org/web/20190608215845/http://phonotactics.anu.edu.au/features.php, marked as “Updated: 4 May 2017”, which I converted to CSV by adapting the Python script phonotactics.py available from https://gist.github.com/xflr6/800401204fe15a6d1b9289149725b790 (download_and_convert_WPD.py); this database gives the actual number of “tonal contrasts” in a language.

I also used the *Glottolog* [68] version 4.1 for the genealogical classification of languages in *language families*, and in *macroareas*.

**Table 4:** Data sources for linguistic tone with the number of languages (the “#” column).

Source	URL	Content	#
*WALS Online* [34]	https://wals.info/feature/13A	3-way classification: “No tones”, “Simple tone system” & “Complex tone system”	513
*LAPSyD*	http://www.lapsyd.ddl.cnrs.fr/lapsyd/index.php	5-way classification: “None”, “Marginal”, “Simple”, “Moderately complex” & “Complex”, as well as the actual number of tones	569
*DL2007* [9]	[58, Annex 6, p. 373–386]; https://doi.org/10.5281/zenodo.4252896	Binary classification: “No” vs “Yes”	60
*PHOIBLE* [35]	https://phoible.org	Actual tone symbols (https://phoible.org/parameters)	2030
*WPHON* [67]	https://web.archive.org/web/20190608215845/http://phonotactics.anu.edu.au/features.php	Number of tones	3160

#### Tone classifications

Given these sources, I decided to compile information about tone in three formats (codings):

1. a *binary* coding, opposing languages that do not use any tone system to those that do (i.e., absence vs. presence): “No”/“Yes”
2. a *3-way ordered* coding of tone system complexity: “None” < “Simple” < “Complex”, and
3. a *count* of the number of tones (or tone symbols) used to describe a language, ranging from 0 (no tone) to a maximum dependent on the data source.

The rules for obtaining these coding for each data source are given in Table 5. Please note that the counts were *rebased*, in the sense that those few languages reported as having 1 tone/symbol were recoded as having 2 (the fact that they are very few, 6 for *LAPSyD*, 4 for *PHOIBLE*, and 3 for *WPHON*, coupled with a case-by-case analysis, suggested that they can be safely collapsed with the languages with 2, all being considered as having simple tone systems), followed by the subtraction of 1 for all languages with at least 2 tones/symbols (i.e., the languages with 0 tones/symbols stay at 0, but all languages with *n* ≥ 2 tones/symbols are recoded as having *n* − 1 tones/symbols)—in this way, we have a continuum of counts from 0 up to the original maximum—1.

**Table 5:** How I coded tone for each data source. The given rules are of the form *original value(s)* ↦ *coded value*; * means “all other original value(s)”; “as is” means the value as given by the data source without change; “rebase” applies only to the counts (see text for details); “–” means that the data source did not contribute to the coding (does not contain useful information).

Source	Binary	3-way	Count
*WALS*	“None” ↦ “No”; * ↦ “Yes”	as is	–
*LAPSyD*	“None” ↦ “No”; * ↦ “Yes”	“None” ↦ “None”; “Marginal” & “Simple” ↦ “Simple”; * ↦ “Complex”	rebase
*DL2007*	as is	–	–
*PHOIBLE*	0 ↦ “No”; * ↦ “Yes”	0 ↦ “None”; ≤2↦ “Simple”; * ↦ “Complex”	rebase
*WPHON*	0 ↦ “No”; * ↦ “Yes”	0 ↦ “None”; ≤3↦ “Simple”; * ↦ “Complex”	rebase

#### Reconciliation of the sources

While the agreement between these five sources is very good (see S1 Fig), there are some languages for which they disagree (e.g., *Angaataha* [anga1290, agm] is coded as “Moderately complex” in *LAPSyD*, but as “Simple” in *WALS*). Moreover, few languages are coded in all sources. Therefore, it would be preferable to arrive at an *agreement* coding of tone that (a) reconciles any existing conflicts and (b) can retain as many languages as possible. To this end, I implemented the following algorithm.

#### For the categorical classifications (binary and 3-way)

I implemented a set of rules based on a hierarchy of the sources and the pattern of agreements and disagreements between them; while still subjective, this is fully replicable, transparent and can be easily modified. I preferred to use manually-curated categorical classifications over the count-level sources, resulting in the following (rough) ordering in terms of precedence: *LAPSyD* ≥ *WALS* ≥ *DL2007* ≥ *WPHON* ≥ *PHOIBLE*. The actual rules are coded as patterns of information about a given language in the available sources and the actions to be taken (e.g., if a language has information in *LAPSyD*, then this is used no matter what other information is available); in this way, the coding conflicts are implicitly solved by picking the “highest” available source.

#### For the counts

I used a similar approach, in that the sources are ordered as *LAPSyD* ≥ *WPHON* ≥ *PHOIBLE*, with the added twist that for the last two (*WPHON* and *PHOIBLE*) I do not pick the “raw” value as given by the database itself, but instead a “corrected” value. More exactly, given that I consider the tone counts in *LAPSyD* as the “gold standard”, I performed first the quadratic regression of the *LAPSyD* counts on the *WPHON* counts and, separately, on the *PHOIBLE* counts, and used the (rounded) value predicted by these regression models from the “raw” value in the corresponding database. These regressions are:

$$\begin{array}{c} L=0.08\left(\pm 0.04\right)+0.92\left(\pm 0.06\right)W-0.04\left(\pm 0.01\right)W^{2} \end{array}$$ (2)

$$\begin{array}{c} L=0.39\left(\pm 0.05\right)+0.68\left(\pm 0.09\right)P-0.04\left(\pm 0.02\right)P^{2}, \end{array}$$ (3)

where *L* is the predicted *LAPSyD* count from the “raw” *WPHON* (*W*) or *PHOIBLE* (*P*) count. This procedure attempts to “align” and “scale” the counts (also allowing a non-linear quadratic term) so that they better map between sources. (Please note that the distribution of the unrounded predicted counts is extremely similar to that of their rounded values overall and within macroareas, as is their relationship with the frequency of the two “derived” alleles; however, as most of the statistical techniques used here for the counts, Poisson regression in particular, need integers, I did not conduct any further statistical analyses on these unrounded predicted values).

#### The agreement classification

**Fig 3:**
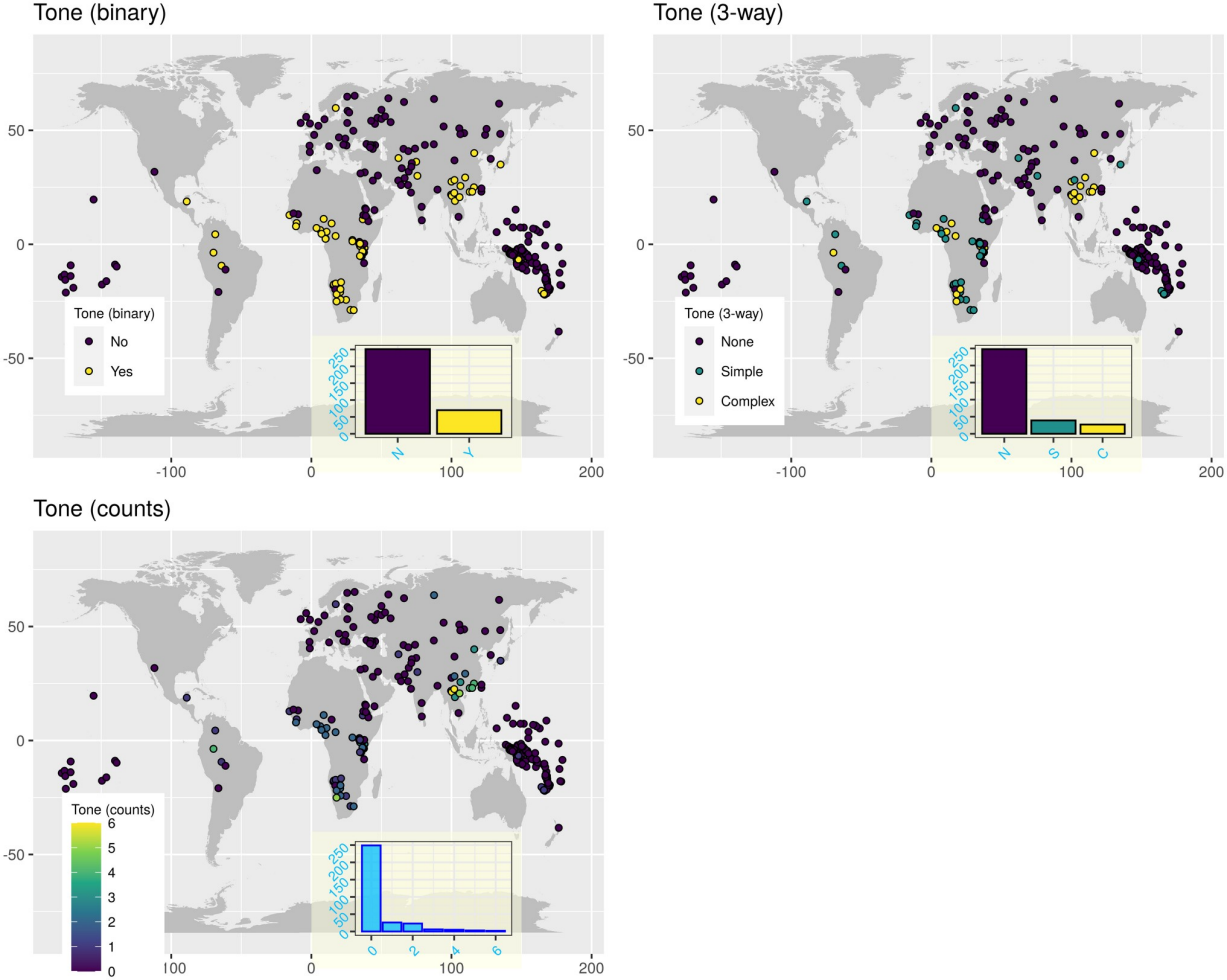
The distribution of the agreement tone. Each circle on the maps represents a unique language, and its colour represents is value. The insets show the overall distributions across all the languages. Top-left: binary tone (inset horizontal axis: “N” = “No”, “Y” = “Yes”); top-right: 3-way classification (inset horizontal axis: “N” = “None”, “S” = “Simple”, “C” = “Complex”); bottom-left: tone counts. The maps were generated with the R package maps which uses public domain data from the Natural Earth project https://www.naturalearthdata.com/.

With these, I obtained three agreement classifications (one binary, one 3-way, and one count), that agree very well with the original sources. These have the following distributions (see also Fig 3):

- *binary* (321): “No” (251), and “Yes” (70).
- *3-way* (314): “None” (248), “Simple” (39), and “Complex” (27).
- *counts* (314): “0” (249), “1” (26), “2” (23), “3” (6), “4” (5), “5” (3), and “6” (2).

There are different numbers of languages with *binary* (321) versus *3-way* and *count* (314) data because of the different patterns of missing data in the five original sources used, the type of coding they contain, and the possibility of deducing “coarser” codings from more “fine-grained” ones (e.g., *binary* from *3-way* and *count*, and *3-way* from *count*) but not the other way around (e.g., *3-way* from *binary*); 314 languages have data for all three codings.

While arguably justified, the hierarchy of the sources used to derive this agreement classification is only one of the many possible, and to check if it unduly biases the results I compared it to an alternative hierarchy of sources (please see the HTML analysis report for full details). This alternative can also be justified by the nature and coverage of the sources, and is, for *binary* and *3-way*: *WALS* ≥ *WPHON* ≥ *LAPSyD* ≥ *DL2007* ≥ *PHOIBLE*, and for *counts*: *WPHON* ≥ *LaPSyD* ≥ *PHOIBLE*. However, on the full dataset, these two agreement codings are very similar (only 27 out of 3798 languages disagree for the *binary* coding, 51 of 3785 for *3-way*, and 59 of 3785 differ by more than 1 symbol for tone *counts*), and on the reduced datasets that also include the “derived” alleles they are virtually identical (2 of 181 differ for *tone1*, 4 of 180 for *tone2*, and 6 of 183 by more than 1 for *counts*).

For the purposes of the analyses reported here, I considered the following variables referring to tone:

- *tone1*: this is a shorter name for the binary coding of tone as “No” (251) vs “Yes” (70).
- *tone2*: this is a binary variable derived from the 3-way coding of tone by opposing “Complex” tone systems (coded as “Yes”, 27 languages) vs “None” and “Simple” collapsed together (coded as “No”, in 287 languages).
- tone *counts*: the unchanged tone counts from above.

While *tone1* is the direct equivalent of the binary presence/absence coding of tone used in the original paper [9], and allows the re-testing of the negative bias against the presence of tone due to high frequencies of the the “derived” alleles formulated there, *tone2* and *counts* are new codings introduced here, potentially reflecting refinements of the original hypotheses, or even new ones. As such, it is possible that the “derived” alleles may (also) have an effect on the complexity of the tone system, in the sense that they may influence the probability that a language has a “complex” tone system or not; the variable *tone2* captures precisely this new hypothesis, and its analysis should be seen as complementing and partially overlapping that of *tone1*. Moreover, it is possible that the “derived” alleles influence not just the presence/absence of tone or of “complex” tone, but, more generally, the actual number of tone distinctions (potentially in a non-linear manner): this justifies the inclusion of the *counts* variable. In fact, the recent experimental evidence that *ASPM*-D influences tone perception in speakers of *Cantonese* (a complex tone language) [26], seems to suggest that the effects of the “derived” alleles (in particular, *ASPM*-D’s) on tone may go beyond “blocking” or “allowing” the emergence of tone in the first place, and may also affect the internal structure and complexity of tone systems (e.g., their “simplification” or “complexification”).

### Putting tone and genes together

Combining the tone and genetic data inevitably results in a loss of a few observations due to missing data, especially in what concerns the frequency of the “derived” alleles. As a reminder, we have three main units of analysis: the actual *genetic sample*, representing a particular group of people for which allele frequency data is available (e.g., using the example given above, SA001509P). The *(meta)population*, which may contain more than one sample (e.g., “Adygei” [PO000017I], containing not only SA001509P but also SA004373R and SA004585W), but one sample belongs to a single (meta)populations. And the “language”, or, more precisely, the *Glottocode* (e.g. the *Adyghe* language with Glottocode adyg1241 and ISO-639–3 code ady): one sample/(meta)populations might speak more than one “language” (in reality or due to ambiguities in mapping), and one “language” can be spoken by more than one sample/(meta)population.

With these, there are in total 175 unique samples in 129 unique (meta)populations speaking 321 unique Glottocodes with data for at least one of the three tone codings and one of the two “derived” alleles; I will denote such counts as 175:129:321. Of these, 175:129:321 (i.e., all) have data for tone *binary*, and 170:124:314 for tone *3-way* and *counts* (5 genetic samples from the 5 (meta)populations “Burunge”, “Hazara”, “Mozabite”, “Oroqen” and “Xibe” speaking 7 “languages” buru1320, efee1239, gyel1242, haza1239, oroq1238, tumz1238 and xibe1242 do not have this info). 170:127:319 have info for *ASPM*-D (5 samples FINRISK, GenDan, GenNed5, KRGDB and Qatari, 2 (meta)populations “Dutch” and “Qatari” and 2 Glottocodes dutc1256 and gulf1241 lack it), 166:128:320 have info for *MCPH1*-D (9 samples, 1 (meta)populations “Bulgarian” and 1 “language” bulg1262 lack it), and 161:126:318 have info for both *ASPM*-D and *MCPH1*-D (14 samples, 3 (meta)populations “Bulgarian”, “Dutch”, “Qatari” and 3 Glottocodes bulg1262, dutc1256, gulf1241 lack this info).

Requiring also information for tone *binary* does not change this (161:126:318), while for *3-way* and *counts* it drops to 156:121:311 (19 samples, 8 (meta)populations “Bulgarian”, “Burunge”, “Dutch”, “Hazara”, “Mozabite”, “Oroqen”, “Qatari” and “Xibe” and 10 Glottocodes bulg1262, buru1320, dutc1256, efee1239, gulf1241, gyel1242, haza1239, oroq1238, tumz1238, xibe1242 are lost).

However, as some of these samples map to more than one Glottocode, for each such sample I only retain the corresponding Glottocodes that have different tone values. Moreover, one (meta)population might have more than one sample, and for each such (meta)population I only retain those with different frequencies for *ASPM*-D and *MCPH1*-D. While this procedure can be criticised, it is conservative in that it gives more weight to divergent signals. With this, the final dataset on which the analyses were conducted contained a total of 181 observations representing 161:126:119 samples:(meta)populations:Glottocodes for tone *binary*, 180 observations for 156:121:118 for *3-way* and, 184 observations for 156:121:121 for *counts*.

Thus, this drop in the number of observations (understood as number of samples, (meta)populations or Glottocodes), from 175:129:321 to 161:126:119 for *binary*, from 170:124:314 to 156:121:118 for *3-way*, and from 170:124:314 to 156:121:121 for *counts* is in large part due to real missing genetic data, but also to the sometimes ambiguous mapping between genetic samples and linguistic varieties, some with the same value for tone. An extreme case is sample SA004382R (“Micronesians”) mapped to no less than 144 Glottocodes, all *Austronesian* languages, the vast majority of which (137) do not use tone—arguably, reducing these to a single “No” observation does not represent a loss of actual information. On the other hand, the loss of populations without genetic information for one of the “derived” alleles (5:2:2 for *ASPM*-D, 9:1:1 for *MCPH1*-D and 14:3:3 for both) is a real loss of information, but (a) it is arguably rather small, and (b) I decided against using various missing data imputation techniques as not to introduce biases in the data. (Moreover, my using of “proxy” SNPs for the “derived” alleles already is a form of very specific missing data imputation, informed by genetic theory and data).

## Methods

I analysed separately the three variables related to tone, namely *tone1*, *tone2* and tone *counts*. For each variable, I selected only the entries with non-missing data for it as well as for the two “derived” alleles; if, for a given sample, there is more than one possible tone or allele frequency values (i.e., corresponding to more than one language with different tone coding or genetic samples with different allele frequency data), I only kept those entries that have different tone and/or allele information. This procedure maintains the uncertainty in the data, and, while it might be seen as giving too much voice to the “exceptions”, it avoids giving too much voice to entries that happen to be similar due to close genealogical relatedness. With these, there are 181 observations among 119 unique Glottolog codes in 35 families for *tone1*, 165 observations among 108 unique *Glottolog* codes in 35 families for *tone2*, and 184 observations among 121 unique *Glottolog* codes in 35 families for tone *counts*.

### Modelling (meta)populations, macroareas and families

As detailed below, I used several non-Bayesian and Bayesian statistical methods for the analysis of the data.

The *(meta)populations* are fully included in the language families, and most have just a few samples: of the 129 unique (meta)populations, 100 have only 1 sample, 17 have 2 samples, 8 have 3, 3 (“Finns”, “Jews_Ashkenazi” and “Russians”) have 4, and there is only 1 (“Han”) with 5 samples. Therefore, I did not explicitly model them in the non-Bayesian approaches, as they add too little information to the samples and the language families (and, in very few cases, cause convergence issues), but I did include them explicitly, as embedded within the language families, in the Bayesian mixed-effects regressions.

However, the *families* do need to be considered (even if most have only one or a few languages: of all the 39 unique families, 16 are represented by a single language, 6 by 2, 5 by 3, 3 by 5, 2 by 7, 3 by 10, and 1 each by 16 (“Afro-Asiatic”), 26 (“Indo-European”), 31 (“Atlantic-Congo”) and 146 (“Austronesian”) languages, respectively), as it is essential to properly model the genealogical non-independence between related languages (and, arguably, genetic samples): therefore, I modelled them as *random effects* (in the now standard approach; e.g. [27]).

*Macroareas*, on the other hand, need special consideration for several reasons:

- there is a very skewed geographical sampling (e.g, there is nothing from Australia, and very few data points from the Americas), the dataset being dominated by Eurasia and Africa;
- despite the obvious fact that geography strongly influences language contact and results in geographic clustering, this is far from a simple phenomenon captured by a limited number of clear-cut “areas”; therefore, there are several proposals of such areas that are arguably independent of each other for the purposes of language contact (e.g., [69–71]), the one used here (based on the macroareas defined in the *Glottolog*) being relatively widely used but still open to criticism (e.g, “Africa” and “Eurasia” are treated as unitary, while PNG and Oceania are placed together within “Papunesia”);
- the distribution of the two “derived” alleles is also highly skewed geographically, being almost completely absent from Africa.

Therefore,

- as “North America” and “South America” each have very few data points, I collapsed them into a single macroarea, “America”;
- in order to control for the influence of the macroareas, I modelled them as *fixed effects* in the non-Bayesian approaches, as *random effects* (crossed with the families and (meta)populations) in a set of Bayesian mixed-effects regressions, and as a *2-dimensional Gaussian Process* (with the families sand (meta)populations) in a different set of Bayesian mixed-effects regressions;
- because the genetic data suggests that the major split is between “Africa” and the rest of the world, coupled with the fact that some methods cannot gracefully handle multi-valued factors, made me dichotomise, for some analyses, macroarea in into “Africa” vs “non-Africa”.

### Operationalization of the hypotheses

Following [9, 10], and the direct experimental tests in [25, 26], I am testing here the following hypotheses:

- there is a **weak negative influence** of the population frequency of *ASPM*-D on *tone1* above and beyond the effects of shared ancestry (*language family*) and contact (*macroarea*);
- there should be **no effect** of *MCPH1*-D on *tone1* independent of *language family* and *macroarea*, or it should be much smaller.

There are no clear predictions concerning the complexity of tone systems (*tone2* and tone *counts*), but we might expect a negative effect of *ASPM*-D:

- there might be a **weak negative influence** of *ASPM*-D on *tone2* above and beyond *language family* and *macroarea*;
- there might be a **weak negative influence** of *ASPM*-D on tone *counts* above and beyond *language family* and *macroarea*;
- there is **no particular influence** of *MCPH1*-D on *tone2* independent of *language family* and *macroarea*;
- there is **no particular influence** of *MCPH1*-D on tone *counts* independent of *language family* and *macroarea*.

### Throwing the causal baby with the confounding bathwater?

However, the “above and beyond the effects of shared ancestry (*language family*) and contact (*macroarea*)” are rather tricky in this particular case, and should be treated with care. These are indeed potential confounds in any cross-linguistic/cross-cultural/cross-population statistical studies and usually result in artificially inflated (i.e., artificially statistically significant) associations [27]. This is due to the fact that the languages/cultures/populations are not statistically independent, but more similar than expected by chance due to “genealogical inertia” (“Galton’s problem”; [63]) and contact. In our case, tone seems to be both stable genealogically (i.e., the daughter languages have a strong tendency to conserve the tonal system of their proto-language) and relatively easy to borrow between neighbouring languages [13, 19–21, 60]. Likewise, given that the two “derived” alleles, *ASPM*-D and *MCPH1*-D, are very probably evolving neutrally [47], it is to be expected that their frequencies in any given population are shaped by drift and admixture [46]. While in small isolated populations their frequency might fluctuate widely across generations (possibly ending in fixation or complete loss), in larger ones they are expected to be relatively stable. Moreover, genetic exchanges between populations (due to inter-marriage, migrations…) lead to more similar frequencies, while various barriers (physical or cultural) might lead to increased differentiation [46, 72]. Thus, any apparent association between tone and the frequency of the “derived” alleles in present-day populations might be *non-causal* but *spurious*, due to a fortuitous accident through which it just so happened that a few populations, some generations ago, had a high frequency of the “derived” alleles and no tone, accident later amplified by language expansions, demographic processes and contact, making the proper control for these non-independencies a must [15, 27].

On the other hand, if the hypothesis that the “derived” alleles induce a weak individual bias that can be amplified by the repeated use and transmission of language across multiple generations [9–11] is true (as strongly suggested by the experimental evidence; [25, 26]), then the cross-linguistic/cross-population effects of this *causal* link might look very similar to the very confounds discussed above. This is so because in populations with a low frequency of the “derived” alleles, tone is “free” to change (i.e., being innovated, complexified, simplified or lost) under the influence of other processes (internally-motivated sound change, language contact, or even climate; [3, 12, 13]), but in populations with a higher frequency of these alleles, there is an extra (weak) force against tone (thus, either a “push” away from tone through increased rates of tone simplification and loss, or a “pull” towards a lack of tone through a low probability of tonogenesis). The effects of this “extra” force (the *negative bias*) would not be instantaneous, but would presumably require several generations.

There are four important scenarios to consider: in the first, there is a significant *increase* in the frequency of the “derived” alleles (due to drift or gene flow), in the second there is a *stable high* frequency of the alleles, in the third, there is a significant *decrease* in this frequency, and in the fourth, a *stable low* frequency of the alleles. However, please note that it is currently unclear what a “significant” change in the frequency of the “derived” alleles should be, if this change is independent or not of the frequency itself, if there are thresholds or more general non-linearities, and if the frequency is modulated by the structure of the communicative network itself [17, 18, 73]—all in need of specific computational and cross-linguistic work that go beyond the scope of this paper; for my purposes here suffices that there are changes in allele frequency that result in changes in the negative bias towards tone at the language level.

In the first scenario, when the frequency of the “derived” alleles increases sufficiently in a population so that the negative bias becomes active, then we would expect that the original language(s) of the population would start simplifying or losing tone (if they had it), or fail to develop or complexify it (even if other conditions hold). If this process is on the same timescale as language differentiation or faster (a few generations/hundreds of years), it might retrospectively look like a “regular” sound change (or, even much harder to ascertain, a failure to change despite favouring conditions), followed (second scenario) by a period of relative stability (lack of tone) across the history of the ensuing language family. Moreover, given that it is highly improbable that such significant changes in allele frequency happen just in a single population that is large enough to support a viable language, or that it would stay confined there, they would likely affect (relatively simultaneously), or spread among, bigger groups of people speaking multiple languages across larger areas; retrospectively, this might look like a period of tone simplification or loss among several languages in contact which can be interpreted as contact-induced, followed by a period of relative stable lack of tone (second scenario).

In contrast, in the third scenario, the frequency of the “derived” alleles decreases sufficiently to remove the negative bias against tone, “allowing” the other forces to resume affecting tone, effectively “opening” up tonogenesis and tone complexification as possible pathways of language change, possibly followed by the fourth scenario, where the frequency of the “derived” alleles is low and the negative bias inactive. Retrospectively, scenario three would be hard to distinguish from the “usual” evolution of tone systems, except by being preceded by a period characterised by a lack of tone in the history of the languages. Scenario four is that of language change “as usual” in the absence of a genetic bias, where tone is gained and lost, complexified and simplified as driven by other factors.

Given what we know about the two “derived” alleles, namely that they emerged relatively recently (*ASPM*-D ≈ 6,000 years ago, and *MCPH1*-D ≈ 37,000 years ago) presumably somewhere in Eurasia, and have since spread and increased in frequency (presumably due to demographic processes of expansion, migration and inter-marriage) slowly and unequally around the globe, it is probable that all scenarios are applicable in different circumstances (linguistic families and geographic areas), but that scenario three is rather improbable.

**Fig 4:**
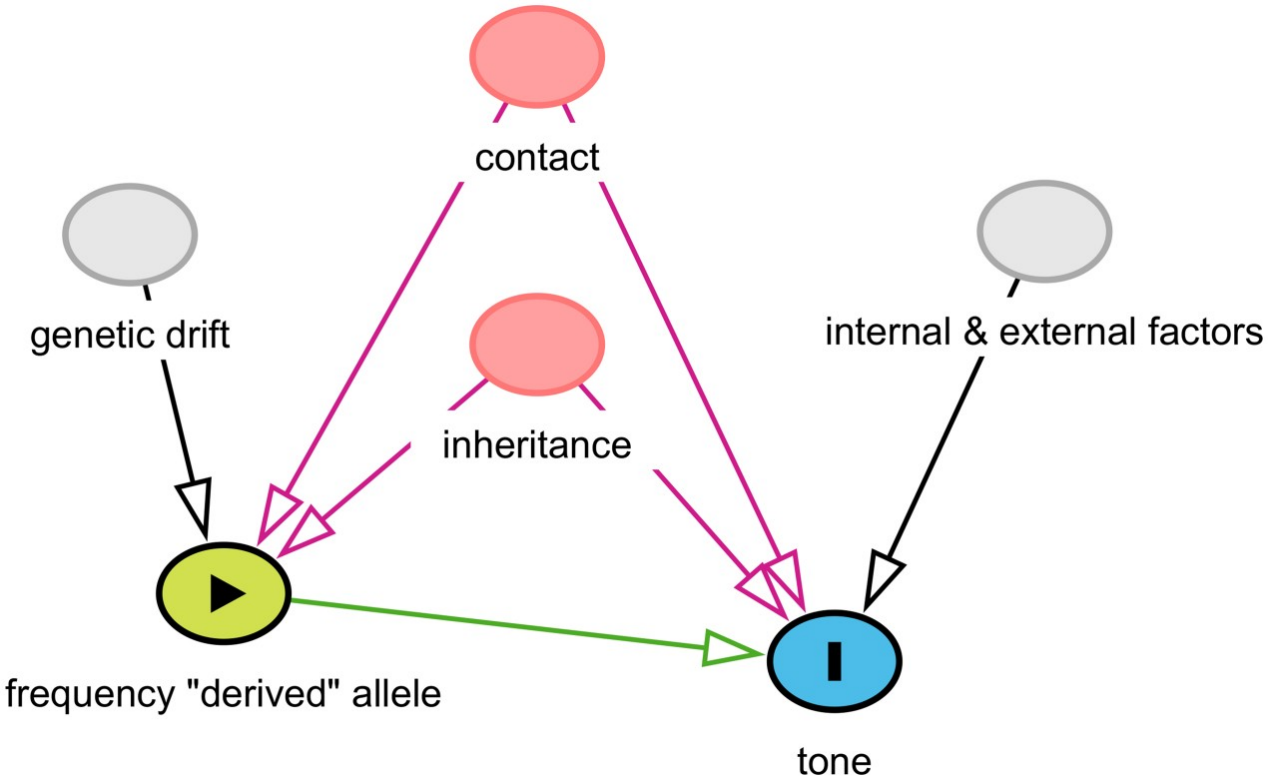
Simplified model of the causal and spurious relationships between the frequency of the “derived” alleles (the *exposure*) and tone (the *outcome*) using Directed Acyclic Graphs (DAGs). The arrows represent direct causal effect; the grey nodes are unmeasured variables.

Thus, because of the intrinsic population dynamics of these “derived” alleles, of the weakness of the bias they produce, and of the complex multi-factorial nature of language change (combining many types of influences and a good dose of serendipity), the causal effects of this bias will largely overlap with those of genealogical inertia (because, just as tone, the allele frequencies of the daughter populations are highly correlated between them and with those of the mother population) and areal effects/language contact (because, just as tone, the “derived” alleles are spread by people moving and interacting with each other). Fig 4 is a representation of these relationships using Judea Pearl’s Directed Acyclic Graph (DAG) approach to causality [30, 74, 75].

### Statistical methods

Given the limitations of the data available (not only in terms of sample size, but also of geographic and linguistic representativeness, and sometimes ambiguous mapping), the complexity of the causal model, and the roles of inheritance and contact discussed above, I used several complementary methods to try to test these hypotheses.

I used both Bayesian and non-Bayesian methods, as each have advantages and disadvantages and comparing them should allow a better estimation of the robustness of the results. On the one hand, the *Bayesian methods* (as implemented here by the brms package [76] in R [77] and Stan [78]) are more flexible and, in this case, allow the *(meta)population* to be included among the random effects, and the *macroarea* to be modelled as a random effect or as a 2D Gaussian Process, but are more computationally expensive. On the other, the *“classic” implementations* (as available through (g)lmer and glmmTMB in, respectively, the lme4 [79] and glmmTMB [80] packages in R) have much lower computational costs, especially relevant for the *randomisation* and *restricted sampling* approaches. Nevertheless, it is important to point out that the Bayesian and non-Bayesian methods produce largely convergent results.

For *tone1* and *tone2* (binary variables) I performed *logistic regressions* (as implemented, in the “classic” approach, by glm and glmer when there is no random structure and where there is one, respectively, and by brms in the Bayesian approach), for the tone *counts* I used *Poisson regression* (glmer and brms, respectively), and for the population frequencies of the “derived” alleles I used *Beta regression* (as implemented by glmmTMB and brms, respectively; please note that, due to the restrictions of Beta regression on the dependent variable, I systematically replaced all 0.0 frequencies by 10^−7^ and all 1.0 frequencies by 1.0 − 10^−7^).

While such an approach would usually require some type of *multiple testing correction*, I decided here against it because (a) at least for *tone1* this is based on a pre-existing hypothesis, and *tone2* and tone *counts* are arguably related to this hypothesis, (b) the various methods and approaches are interpreted *together* rather than independently, (c) as shown by the power analysis and the results themselves, the dataset is small and the effects weak enough for multiple testing correction to effectively make the whole enterprise futile purely for statistical power reasons, and (d) it is technically difficult to correct across such different methods, and especially across frequentist and Bayesian ones (for the latter, it is not entirely clear even if this is a meaningful issue; e.g. [81]). However, when interpreting the results the fact that there is no such correction should be kept in mind.

#### (Mixed-effects) regressions

This implements the largely current “standard” approach in cross-linguistic studies, where the dependent variable (DV) is regressed on the independent variable(s) (IVs), while the potential confounds are modelled as fixed or random effects [27, 28].

In the Bayesian approach, the DV is either *tone1*, *tone2* or *counts*, and the IVs of interest are the population frequencies of *ASPM*-D and *MCPH1*-D (and their interaction), while the confounds are *macroarea*, language *family* and (nested within family), the *(meta)population*. The last two of these confounds (*family* and *(meta)population*) were modelled as random effects, but I modelled *macroarea* either as yet another (crossed) random effect (in one set of regressions) or as a bi-dimensional (2D) *Gaussian Processes* [82]. Here, I use a Gaussian Process to model the effects of geographical space as implemented by the gp() function in package brms [76], using the longitude and the latitude of each sample grouped separately for each macroarea; this allows the continuous modelling of the effects of geographic distance within each macroarea (see, for example, [30] for such an application). However, I consider this approach as “experimental” here as it seems to suffer from too few data for too many parameters and results in very wide posterior distributions. The importance of the IVs of interest is captured by, first, their posterior distribution (plotted and summarised by its mean and 89% Highest Density Interval (HDI); [30]), second, by formal hypothesis testing of the one-sided expected direction of the effect (here, < 0) and versus the point (i.e., two-sided) value 0 (these tests produce both probabilities and evidence ratios; see function hypothesis in package brms for details), third, through comparisons with the ROPE (the region of practical equivalence, a small interval around 0.0, usually [-0.1, 0.1] but that can vary depending on the particular model) which produces the percent of the HDI within this interval and probabilities that can be interpreted similar to frequentist *p*-values (see functions rope and p_rope in package bayestestR and [81] for details), and, fourth, by the comparison of the model with the IV and the nested model without it (using multiple criteria: the Bayes Factor, leave-one-out cross-validation (LOO), the Widely Applicable Information Criterion (WAIC), and K-Fold Cross-Validation; please see, for example, [30] for details).

In the “classic” approach, the DVs and the IVs of interest are the same as above, while the confounds are *macroarea* (modelled as a fixed effect) and its interactions with *ASPM*-D and *MCPH1*-D, and language *family* (as a random effect); the (meta)populations are not explicitly modelled. I individually tested the significance of each fixed effect separately by performing a likelihood ratio test and comparing the Akaike Information Criteria (AIC) between the model with the fixed effect of interest and the model without it.

In all regression models that include the frequency of either or both “derived” alleles as IVs, these frequencies are *z*-scored (i.e., transformed into a variable with mean 0.0 and standard deviation 1.0 through $\frac{f-mean(f)}{sd(f)}$).

Only in the “classic” approach, for each DV:

*All data*. I first fitted the regression model on all the available data;
*Randomization*. followed by a permutation approach where I repeatedly (*n* = 1000) shuffle the data and re-fit the regression model, resulting in a null distribution of model fits that can be compared to the original fit to the unshuffled data. In fact, there are 18 different scenarios resulting from combinations of three parameters that test slightly different null hypotheses (see Tables 6 and 7);
*Restricted sampling*. finally, a popular approach in typology is represented by sampling only one language from each genealogical and/or areal unit [69, 83, 84], which I implemented here by repeatedly picking only one language from each family, fitting a regression model (without any random effects structure, as each family now has exactly one member) while controlling or not for macroarea, and analysing the distribution of the model fits (this time, the comparison is done against the null hypothesis of no effect).

**Table 6:** The parameters of the randomisation approach, defining, on the one hand, what is permuted and within what constraints, and what to control for in the regression models, on the other.

Parameter	Meaning	Possible values
*permute*	what is permuted	*tone* = permute the tone variable → destroys the patterning of tone
		*alleles-together* = permute the two alleles together → destroys the patterning of the two “derived” alleles but not the correlation between them
		*alleles-independent* = permute the two alleles independently → destroys the patterning of the two “derived” alleles and the correlation between them
*within*	constraints on permutations	*unrestricted* = all observations are freely permuted → destroys all structure in the data
		*families* = observations are permuted within their family → destroys the within-family structure, but conserves the between-families variation, i.e., conserves the genealogical signal
		*macroareas* = observations are permuted within their macroarea → destroys the within-area structure, but conserves the between-areas variation, i.e., conserves the areal (contact) signal
*macroarea*	is there control for macroareas?	*none* = no control at all
		*fixef* = yes, as fixed effect

**Table 7:** The 18 randomisation scenarios. Parameter names and values (columns 2–3) are defined in Table 6, Name (1^st^ column) is a 3-letter shorthand: 1^st^ letter: T = tone, L = alleles-together (“linked”) and I = alleles-independent (“independent”); 2^nd^ letter: U = unrestricted, M = macroareas, F = families; 3^rd^ letter: N = none, F = fixef. –”– means as above.

Name	Permute	Within	Macroarea	Interpretation
TUN	*tone*	*unrestricted*	*none*	tone is permuted across the whole sample → destroys the family and macroarea structure of tone, but keeps it for the alleles; macroarea is not included
LUN	*alleles-together*	*unrestricted*	*none*	the frequencies of the two “derived” alleles are permuted across the whole sample together → destroys the family and macroarea structure of the two alleles (while preserving correlations between them), but keeps it for tone; macroarea is not included
IUN	*alleles-independent*	*unrestricted*	*none*	the two alleles are permuted across the whole sample independently → destroys the family and macroarea structure of the two alleles and the correlations between them, but keeps it for tone; macroarea is not included
TUF	*tone*	*unrestricted*	*fixef*	as TUN but controlling for macroarea
LUF	*alleles-together*	*unrestricted*	*fixef*	as LUN –”–
IUF	*alleles-independent*	*unrestricted*	*fixef*	as IUN –”–
TMN	*tone*	*macroareas*	*none*	as TUN but only data points from the same macroarea are permuted
LMN	*alleles-together*	*macroareas*	*none*	as LUN –”–
IMN	*alleles-independent*	*macroareas*	*none*	as IUN –”–
TMF	*tone*	*macroareas*	*fixef*	as TMN but controlling for macroarea
LMF	*alleles-together*	*macroareas*	*fixef*	as LMN –”–
IMF	*alleles-independent*	*macroareas*	*fixef*	as IMN –”–
TFN	*tone*	*families*	*none*	as TUN but only data points from the same family are permuted
LFN	*alleles-together*	*families*	*none*	as LUN –”–
IFN	*alleles-independent*	*families*	*none*	as IUN –”–
TFF	*tone*	*families*	*fixef*	as TFN but controlling for macroarea
LFF	*alleles-together*	*families*	*fixef*	as LFN –”–
IFF	*alleles-independent*	*families*	*fixef*	as IFN –”–

Please note that because permutations and restricted sampling involve randomness, the results might differ slightly between runs.

#### Mediation and path analysis

**Fig 5:**
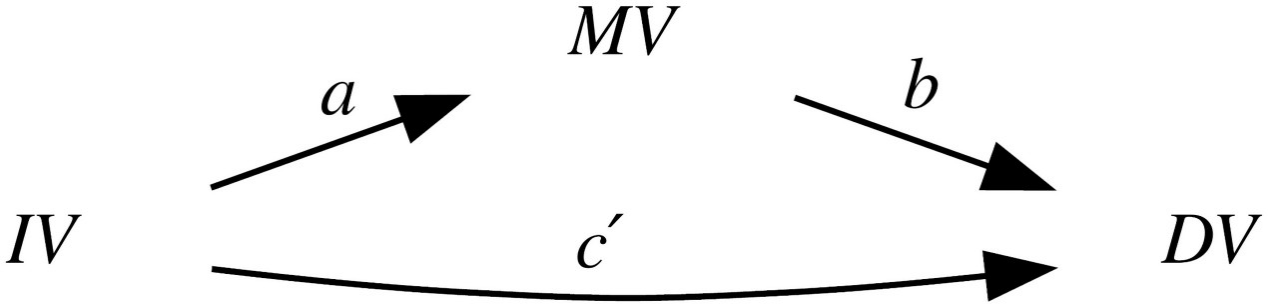
Visual representation of the mediation model. *IV* is the independent variable (here, dichotomised *macroarea*: inside vs outside Africa), *DV* is the outcome of interest (here, the various codings of tone, *tone1*, *tone2* or tone *counts*), and *MV* is the mediator (here, the population frequency of one of the “derived” alleles, *ASPM*-D or *MCPH1*-D). The average direct effect *ADE* = *c*′, the average indirect effect *ACME* = *a* × *b*, and the total effect is their sum, *TE* = *ADE* + *ACME* = *c*′ + *a* × *b*. The coefficients *a*, *b* and *c*′ are obtained from fitting two regression models simultaneously (in R formula notation): *DV* ∼ *IV* + *MV* and *MV* ∼ *IV*. These path plots are generated using Graphviz (https://graphviz.org/) as implemented by DiagrammeR::grViz in R.

**Fig 6:**
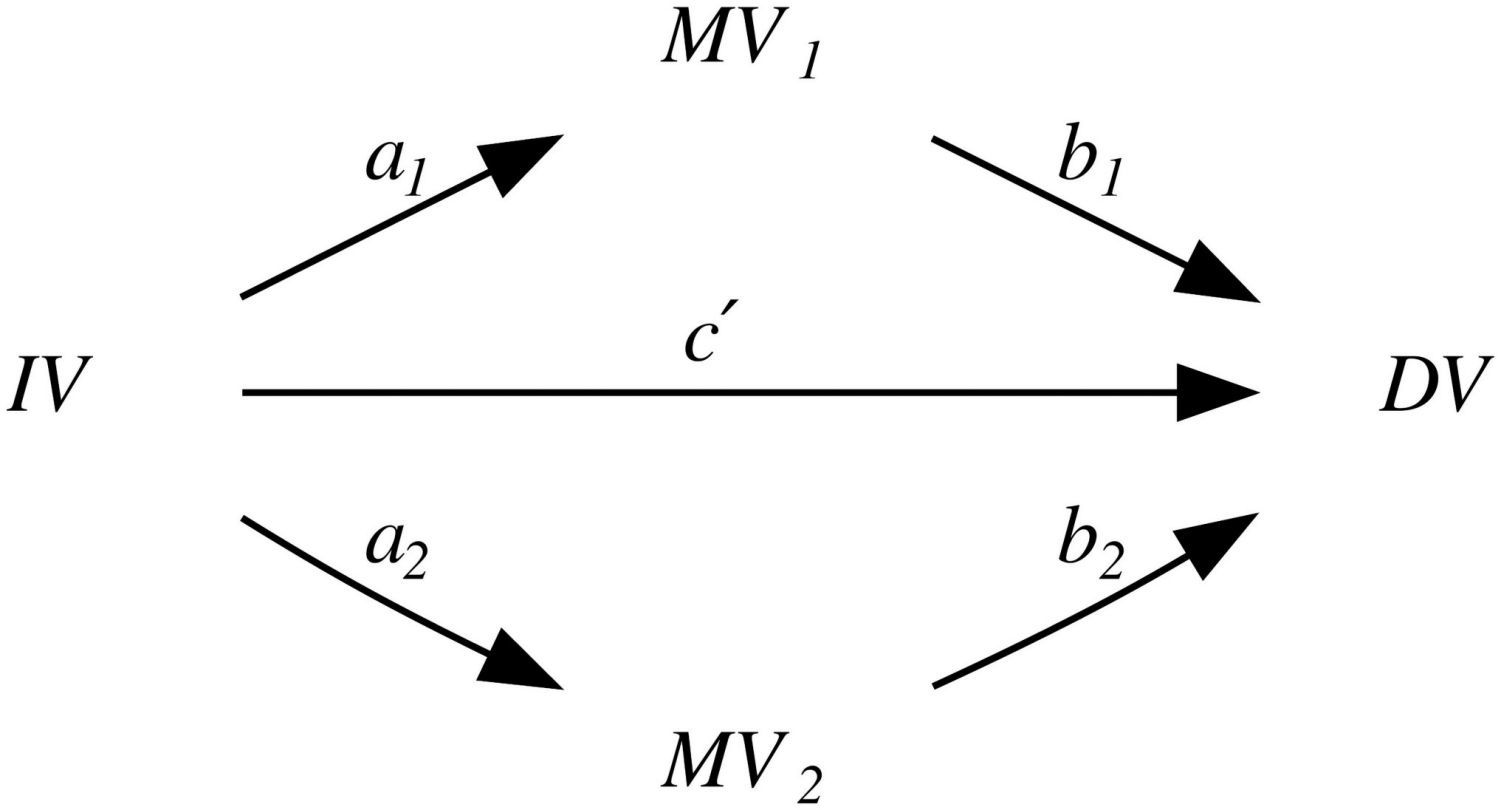
Visual representation of the path model showing the *IV* (Africa vs the rest of the world), the *DV* (tone) and the two mediators *MV*_1_ and *MV*_2_ (*ASPM*-D and *MCPH1*-D), as well as all the path coefficients (*a*_1_, *a*_2_, *b*_1_, *b*_2_ and *c*′). Please note that a similar model was also experimentally implemented in a Bayesian framework as simultaneous multiple mediation which explicitly controls for *family* and *(meta)population* as random effects.

Because *macroarea*, and especially its dichotomisation as Africa vs the rest of the world, predicts both the distribution of tone and the frequency of the two “derived” alleles, it confounds any potential causal effect that the alleles may have on tone. *Mediation analysis* [85] allows the partitioning of the *total effect* (TE) of a variable of interest (here, dichotomised *macroarea*: Africa vs the rest of the world) on the outcome (here, tone) into its *direct effect* (ADE) and its *mediated* (or indirect) *effect* (ACME), the latter being “channelled”, usually, through one mediator variable (here, *ASPM*-D or *MCPH1*-D)—see Fig 5. Here, I performed mediation analysis both in a “classic” approach (using the R package mediation [86], with logistic regressions for *tone1* and *tone2*, linear regressions for the *z*-scored frequencies of *ASPM*-D and *MCPH1*-D, and Poisson regression for tone *counts*, controlling for language *family* indirectly, through restricted sampling), and in a Bayesian framework (using the package brms and a custom-written extension of the mediation function from package sjstats [87], with logistic regressions for *tone1* and *tone2*, Beta regressions for the actual, i.e., non-*z*-scored, frequencies of *ASPM*-D and *MCPH1*-D, and Poisson regression for tone *counts*, with language *family* and *(meta)population* as random effects). This extension of the mediation function allows the *simultaneous* modelling of two or more mediators, so that I could implement a model similar to the one discussed below for *path analysis* (Fig 6) while explicitly controlling for *family* and *(meta)population* as random effects, but this should be considered as experimental.

*Path analysis* [88] is even more flexible, here allowing the simultaneous modelling of the mediation effects of both “derived” alleles simultaneously besides the direct effect of *macroarea* on tone, as shown in Fig 6 (but, as described above, I experimentally implemented this simultaneous mediation in a Bayesian framework). Again, the *macroarea* was dichotomised as Africa vs the rest of the world, but due to the limitations of the lavaan package [89], the binary *IV* (the dichotomised *macroarea*) and the binary *DV* (*tone1* and *tone2*) were coded either as numeric (0 vs 1; “rest of the world” = 0, “Africa” = 1, and “No” = 0, “Yes” = 1) or as ordered categorical (“rest of the world” < “Africa”, and “No” < “Yes”), while tone *counts* were considered numeric; here, the “derived” allele frequencies are *z*-scored.

Both the mediation (the “classic” approach) and path analyses have trouble modelling the language family as a random effect, so, for each DV and type of model:

*all data:*. I first fitted the model on all the available data, where there is no control for *family*;
*restricted sampling*. I repeatedly fitted the model to a reduced dataset obtained by picking only one language from each family, the resulting distribution of model fits controlling thus for family.

(Please note that I do not need to control for *macroarea*, as it is explicitly modelled as the IV).

#### Machine learning

For the two binary outcomes, *tone1* and *tone2*, I also implemented two classification techniques widely used in machine learning, *decision trees* and *random forests*. Both are used to find the subset of predictors and rules that best predict the binary outcome, and result not only in measures of how well the model fits/predict the data, but also in a set of explicit rules (decision trees) and ranking of how important the predictors are (random forests).

The measures of fit that I am using are *accuracy*, *sensitivity* (or *recall*), *specificity* and *precision*; given the observed (true) binary outcome and the model’s predictions (i.e., what the model “thinks” or “labels as”), the relationship between the two is described by four measures:

- the number of *true positives* (TP): observation and prediction agree on “Yes”,
- the number of *true negatives* (TN): observation and prediction agree on “No”,
- the number of *false positives* (FP): observation is “No” but prediction is “Yes”,
- the number of *false negatives* (FN): observation is “Yes” but prediction is “No”.

With these, the total number of observations *N* = (*TP* + *FP* + *FN* + *TN*), and:

- *accuracy* = (*TP* + *TN*)/*N*, i.e., what proportion of observations were correctly labelled (= true positives and true negatives) by the model?
- *sensitivity* = *TP*/(*TP* + *FN*), i.e., what proportion of the actual “Yes” observations (= true positives and false negatives) are labelled “Yes” (= true positives)?
- *specificity* = *TN*/(*TN* + *FP*), i.e., what proportion of the actual “No” observations (= true negatives and false positives) are labelled “No” (= true negatives)?
- *precision* = *TP*/(*TP* + *FP*), i.e., what proportion of the observations labelled “Yes” (= true positives and false positives) are actually “Yes” (= true positives)?

Ideally, these measures of fit should be close to 100%, but deviations point to different types of failures and biases.

One important issue for such models is *overfitting*, where the model “overlearns” the data, fitting it very well, but doing very poorly at predicting new, unseen observations generated by the same processes. One popular technique to overcome this is to repeatedly split the original dataset into two complementary subsets: a training and a testing one. While the training subset is usually larger and is used to fit the model, the testing subset contains observations not yet “seen” by the model, and is used to estimate the fit measures that capture the capacity of the model to generalise to new situations. Here I used an 80%:20% split of the observations between training and testing, stratified by *macroarea* (i.e., making sure that the distribution of the observations in each subset reflects the distribution of the observations by macroareas in the full dataset), repeated 100 times. Please note that because the random forests have an internal bootstrap mechanism, this repeated training/testing procedure was not applied to them.

The decision trees were implemented by ctree in R’s package partykit [90], while for random forests I used randomForest in package randomForest [91] as well as the conditional random forests implemented by cforest in package partykit. randomForest provides two measures of relative variable importance: one is based on the mean decrease in accuracy if the variable is permuted, while the second is based on the mean decrease in node impurity (measured by the Gini index) when splitting on the variable; while the first captures how much a variable helps in making accurate predictions, the second focuses on producing more homogeneous splits. For cforest, I used the unconditional variable importance, which is similar to the mean decrease in accuracy.

Finally, given the importance of the *macroarea* as a confound, I fitted these models with and without *macroarea* as a predictor. (Please note that these models do not control for language family at all).

#### Phylogenetic approaches

While these hypotheses seem naturally amenable to phylogenetic approaches, including regression while controlling for phylogeny (as implemented, for example, in brms [76]) and correlated evolution (as implemented, for example, in phytools [92] and RevBayes [93]), these data are not appropriate for such analyses. First, there are too few language families with enough languages and phylogenetic structure; essentially, just 6 families have at least 5 languages: *Turkic* (10 languages), *Atlantic-Congo* (15), *Indo-European* (20), *Afro-Asiatic* (9), *Uralic* (8) and *Sino-Tibetan* (7). Second, even these families show very little internal variation in tone and in the frequencies of the two “derived” alleles. Third, most phylogenetic methods also require branch lengths, which are notoriously controversial and hard to obtain for language families [5, 94]. However, even collecting more data might still not make such methods applicable, especially if the observation that families are internally very homogeneous for tone and the two “derived” alleles is confirmed, in which case we will either need to use highly controversial language trees above the level of the family [95–97] or exploit tiny variations within very large families.

## Results

The results are presented by outcome and method. While such structure makes this section rather dense and relatively hard to follow, it has the advantage that it does not emphasize any particular narrative thread, outcome or method. Instead, the reader is presented with all the results, leaving the highlighting of the various narratives for the *Discussion and conclusions* at the end. Each method has its own strength and limitations in relation to these data and questions, and they should be seen as complementary rather than in competition, and their results should be interpreted together.

While being, in certain ways, the fundamental type of approach used here, the individual *regressions* do not correctly model the postulated flow of causality between tone, the “derived” alleles, and other factors (including historical accidents) influencing the demographic and linguistic processes. Among the multiple types of regression used here, the “classic” mixed-effects models using glmer are probably the least reliable, with the Bayesian approach (brms) modelling *macroarea* as a random effect the most informative, while using a 2D Gaussian process should be seen as more “experimental” in nature. The randomization approach is very flexible and allows the fine control of various confounds, the most informative for our purposes here being: TUF (tone: unrestricted: macroareas; please see Table 7 for details) and IUF (alleles-independent: unrestricted: macroareas), which check if the overall clustering of tone or the alleles drives the observed association after controlling for macroareas; TMN (tone: macroareas: none) and IMN, which check if the association is due to their clustering specifically within macroareas; and TFF (tone: family: macroareas) and IFF, which check if the assocation is due to their clustering specifically within families after controlling for macroareas. The restricted sampling implements a different way to control for family and macroareas.

*Mediation* and *path* analyses do a much better job at modelling the flow of causality, and should therefore have more weight than the individual regressions, but mediation analysis deals with each allele separately, while path analysis and the “experimental” simultaneous mediation analysis treat them simultaneously. The Bayesian mediation analysis is the most appropriate and allows the control for family and macroarea, followed by restricted sampling (which also controls for these confounds), with the “classic” mediation using the full dataset being seen as suggestive. The path analysis on the whole dataset should be seen as suggestive (as it does not control for family and macroarea), but the restricted sampling does allow the control for these confounds.

Finally, the *machine learning* techniques should be seen as “experimental” and suggestive, complementing the others, as they do not control for the effect of family at all, but allow the explicit modelling of the macroarea and the quantification of the predictive importance of the “derived” alleles and of the macroarea.

Thus, the interpretation of the results is easy when various approaches “say the same thing”, but when there are disagreements, it should be driven by the mediation and path analyses (especially the Bayesian and restricted sampling), followed by the regressions (Bayesian, restricted sampling, and the randomisation scenarios highlighted above), but still in the context of all the results taken together.

### The “derived” alleles are structured by macroarea and family

Before we analyse the relationships between the population frequencies of the “derived” alleles and tone, it is important to understand how they are structured by *macroarea* (a proxy for contact) and language *family* (a proxy for genealogy). To this end, I regressed *ASPM*-D and *MCPH1*-D separately on *macroarea* (as fixed effect) using mixed-effects Beta regression with *family* as random effect (in R’s notation: *a* ∼ 1 + *M* + (1∣*F*) and *m* ∼ 1 + *M* + (1∣*F*), where *a* is the population frequency of *ASPM*-D and *m* of *MCPH1*-D, *M* is *macroarea*, *F* is *family*; ∼ is the regression operator linking the DV on the left to the fixed and random effects on the right; 1 represents the intercept, + adds new predictors; (1 ∣ *F*) denotes the random effects structure, here varying intercepts by family), and I found that their distribution is very strongly clustered within *families* (the *intra-class correlation* coefficients are: *ICC*(*ASPM*-D) = 71.5% and *ICC*(*MCPH1*-D) = 100.0%), and that *macroarea* predicts their distribution very well (for *ASPM*-D: *p* = 3.4 ⋅ 10^−16^, *R*^2^ = 49.5%; for *MCPH1*-D: *p* = 3.1 ⋅ 10^−12^, *R*^2^ = 73.2%). As a reminder, the intra-class correlation coefficient, *ICC*, represents the proportion of the variance explained by the grouping due to the random effects, and varies between 0% (the grouping contains no information) to 100% (basically all individual observations in a given group are identical). The *adjusted* ICC only considers the random effects, while the *conditional* ICC also considers the fixed effects as well, and they are equal when there are no fixed effects (i.e., for the null models *DV* ∼ 1 + (1 ∣ *F*)). Here, I report only the adjusted ICC computed on the null models, because we are interested in the clustering of the variance due to the random effects.

Separating Africa from the rest of the world seems to drive most of this effect, due to the overall lower frequencies of these alleles in Africa: for *ASPM*-D: *p* = 2.3 ⋅ 10^−14^, *R*^2^ = 34.5%; for *MCPH1*-D: *p* = 3.2 ⋅ 10^−9^, *R*^2^ = 33.7%.

Thus, the population frequencies of the “derived” alleles are strongly confounded by *macroarea*, and, in fact, seem mostly driven by the difference between Africa and the rest of the world.

### Is there tone? (*tone1*)

**Fig 7:**
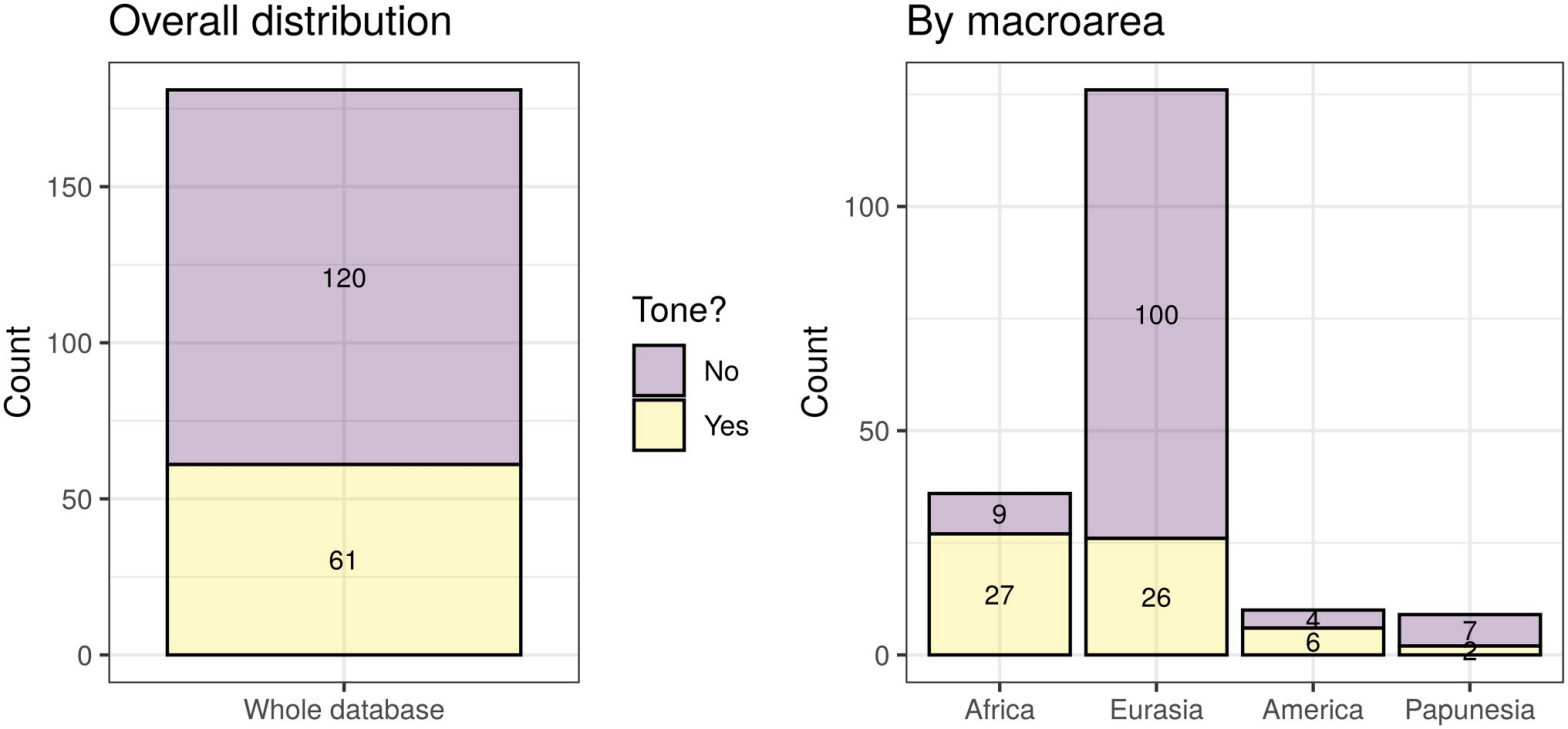
Distribution (as number of languages) of *tone1* (no tone vs any type of tone system) across the full database (left) and by macroarea (right). Please note that the vertical axes have different scales to avoid the macroareas with small sample sizes to be visually “squashed” by the whole database.

When selecting all unique observations with non-missing data for *tone1*, *ASPM*-D and *MCPH1*-D, there are 181 observations, distributed among 119 unique Glottolog codes (languages) in 35 families (the number of languages per family ranges from 1 to 48, with mean 5.2 and median 2). There are 61 (33.7%) languages with tone (“Yes”) in the dataset ($\chi_{1}^{2}=19.2$, *p* = 1.2 ⋅ 10^−5^), and the distribution by macroarea is 36 (27 = 75% “Yes”) in Africa, 126 (26 = 20.6%) in Eurasia, 10 (6 = 60%) in America, and 9 (2 = 22.2%) in Papunesia (between macroareas $\chi_{3}^{2}=40.7$, *p* = 7.5 ⋅ 10^−9^); see Fig 7.

**Fig 8:**
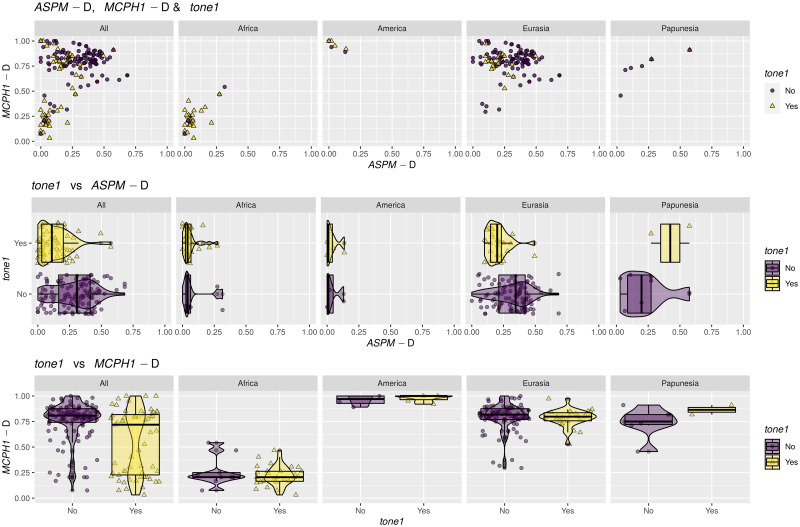
Relationship between tone1 (colour & shape) and the population frequency of *ASPM*-D and *MCPH1*-D in the whole database and separately by macroarea (columns). Top row: scatter plots of *ASPM*-D (horizontal axis) and *MCPH1*-D (vertical axis) versus *tone1* (dot colour & shape). Next two rows show the “derived” allele frequency (as actual jittered observations, violin plots and boxplots) versus *tone1* values (color) for *ASPM*-D (middle row) and *MCPH1*-D (bottom row); please note that the orientation of the axis showing the “derived” allele frequency is the same as in the top row plots (i.e., x-axis for *ASPM*-D, and y-axis for *MCPH1*-D.

The relationship between *tone1* and the population frequency of the two “derived” alleles is shown in Fig 8. It can seen that, globally, there seems to be a difference between languages with and without tone: while tone languages (*tone1* == “Yes”) tend to be found when *ASPM*-D has a low frequency, the others (*tone1* == “No”) tend to be found at high frequencies of both “derived” alleles. Zooming in on each macroarea shows different patterns: while there seems to be a difference for *ASPM*-D in Eurasia (higher for “No”) and Papunesia (lower for “No”), there seems to be no differences in Africa and America. However, such plots can be very misleading because these points represent related languages and/or alternative genetic and linguistics values for the same sample—actual statistical analyses are needed.

#### Regressions

I fitted a “classic” mixed-effects logistic regression model of *tone1* on *macroarea*, the *z*-scored population frequencies of the two “derived” alleles and their interaction as fixed effects, and *family* as random effect to the whole data (building on the notations introduced previously: *t*1 ∼ 1 + *a* + *m* + *M* + *a*: *m* + (1 ∣ *F*), where *t*1 is *tone1*, and: represents interaction). I performed hierarchical model comparisons to test each relevant predictor by comparing the models with and without the predictor (including with the null model with no fixed effects) producing *p*-values using an appropriate test as implemented by R’s anova() function (here, for logistic regressions, a *χ*^2^ test); I also considered the quadratic effects of the “derived” allele frequencies but while I will report here only on their linear effects these are in the full HTML report document. First, as expected, *tone1* is strongly clustered within families (*ICC*(*tone1*) = 70.4%). Second, the interaction does not contribute (*p*(*ASPM*-D:*MCPH*-D) = 0.86) and was removed from the model. Third, as expected, *macroarea* predicts *tone1* by itself (*p* = 0.00082, *R*^2^ = 23.3%; for mixed-effects models, the proportion of variance explained by the model is represented by *Nakagawa’s* *R*^2^, where the *marginal* estimate considers only the fixed effects, while the *conditional* also considers the random effects as well. Here, I only show the marginal *R*^2^, as we are interested in the fixed effects). Fourth, when excluding *macroarea* from the model, the two “derived” alleles together predict tone (*p* = 0.0049, *R*^2^ = 13.4%), with each by itself having negative significant effects on *tone1* (*ASPM*-D: *β* = −1.00±0.37, *p* = 0.0041, *R*^2^ = 10.0%; *MCPH1*-D *β* = −1.04±0.39, *p* = 0.0064, *R*^2^ = 9.4%). However, adding *macroarea* as a fixed effect makes the specific contributions of the two “derived” alleles not significant (*ASPM*-D: *β* = −0.37 ± 0.45, *p* = 0.42; *MCPH1*-D: *β* = −0.39 ± 0.57, *p* = 0.50).

**Fig 9:**
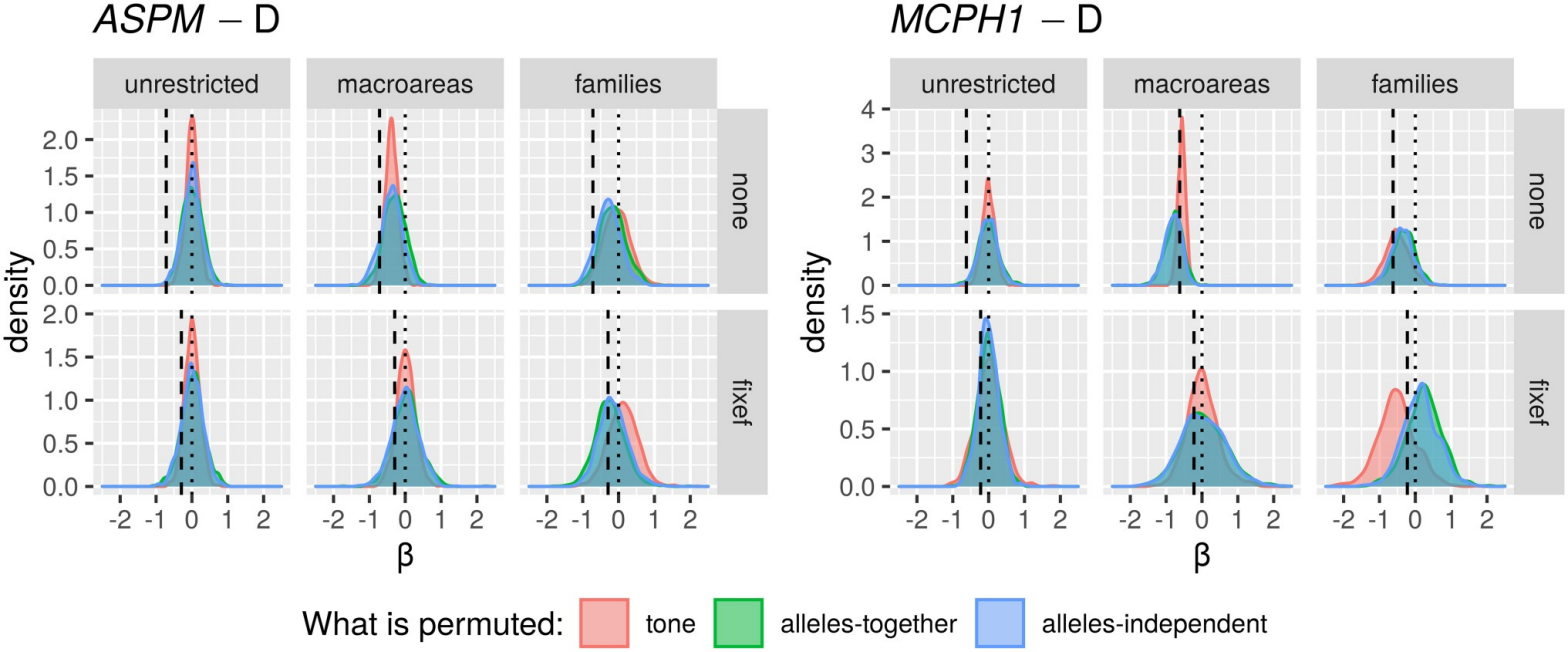
Distribution of the effect size *β* (horizontal axis) of *ASPM*-D (left) and *MCPH1*-D (right) on *tone1* in the permutation regressions without restrictions (“unrestricted”), only within macroareas (“macroareas”), and only within families (“families”), without controlling for macroarea (“none”) or when including macroarea as a fixed effect (“fixef”). Each individual plot shows the original *β* obtained on the unpermuted data (vertical dashed black line), 0.0 (vertical dotted black thin line), and the distribution of the permuted *β* for the three variables to be permuted (the coloured areas): tone by itself (red), the two “derived” alleles together as a unit (green), and the two “derived” alleles shuffled individually (blue). Please note that the y-axes vary between plots, but the x-axes are fixed for comparability. If a distribution is centred on 0.0 (e.g., “ASPM”/“unrestricted”/“none” in the top left) then the permuted data show no effect of the “derived” allele (here, *ASPM*-D) on tone (i.e., the regression slope *β* is 0.0). The less overlap is between the observed *β* (dashed black line) and such a distribution, the more “special” the relationship between the “derived” allele and tone is relative to the structure destroyed by that specific permutation; conversely, if the observed *β* is largely included in a distribution, the more such a value is expected by chance given the constraints of the permutation.

Permuting the observations (Fig 9 and Table 8), shows that *ASPM*-D has a negative effect on *tone1*, detectable even when controlling for *macroarea* as a fixed effect, except when restricting the permutations within families (which is due, on the one hand, to the many families with few languages for which permuting the data does not change much, and, on the other, to the strong within-family clustering of both *ASPM*-D and *tone1*). In contrast, for *MCPH1*-D, there seems to be a negative effect only for unrestricted permutations when not controlling for *macroarea*.

**Table 8:** The results of the regressions on the randomisation scenarios. The 3-letter scenario name as in 7. For each of *tone1*, *tone2* and tone *counts*, I show the percent of models fitted on permuted data that, compared to the model fitted on the original data, have a better AIC, a smaller (i.e., more negative) regression coefficient *β* for *ASPM*-D, and a smaller *β* for *MCPH1*-D, respectively. The lower these percentages, the more “special” is the relationship on the original dataset relative to the structure that is destroyed in each scenario. In **bold** are the values < 5%.

Name	*tone1*			*tone2*			tone *counts*		
	*AIC*	*β*_*ASPM*−*D*_	*β*_*MCPH*1−*D*_	*AIC*	*β*_*ASPM*−*D*_	*β*_*MCPH*1−*D*_	*AIC*	*β*_*ASPM*−*D*_	*β*_*MCPH*1−*D*_
TUN	**0%**	**0%**	**0%**	**0%**	**1%**	**0%**	**0%**	**4%**	**2%**
LUN	**0%**	**0%**	**2%**	31%	10%	6%	**1%**	**0%**	**0%**
IUN	**1%**	**0%**	**1%**	32%	9%	4%	**1%**	**0%**	**0%**
TUF	**0%**	8%	25%	**0%**	6%	23%	**0%**	33%	30%
LUF	68%	15%	23%	84%	17%	25%	81%	16%	12%
IUF	68%	16%	20%	83%	16%	21%	81%	13%	6%
TMN	**0%**	**4%**	28%	**0%**	**1%**	**1%**	**0%**	12%	7%
LMN	26%	7%	73%	40%	21%	42%	18%	6%	20%
IMN	32%	15%	78%	44%	28%	45%	20%	7%	19%
TMF	**0%**	11%	29%	**0%**	7%	22%	**0%**	33%	35%
LMF	65%	19%	35%	80%	28%	38%	79%	23%	26%
IMF	66%	20%	35%	80%	25%	37%	81%	24%	26%
TFN	**2%**	**3%**	36%	31%	28%	16%	24%	28%	8%
LFN	**2%**	5%	16%	20%	29%	15%	9%	32%	**4%**
IFN	**2%**	10%	24%	25%	22%	25%	10%	40%	8%
TFF	**1%**	16%	74%	45%	54%	43%	18%	63%	54%
LFF	66%	46%	16%	80%	43%	18%	83%	61%	5%
IFF	66%	37%	22%	80%	34%	24%	82%	59%	11%

**Fig 10:**
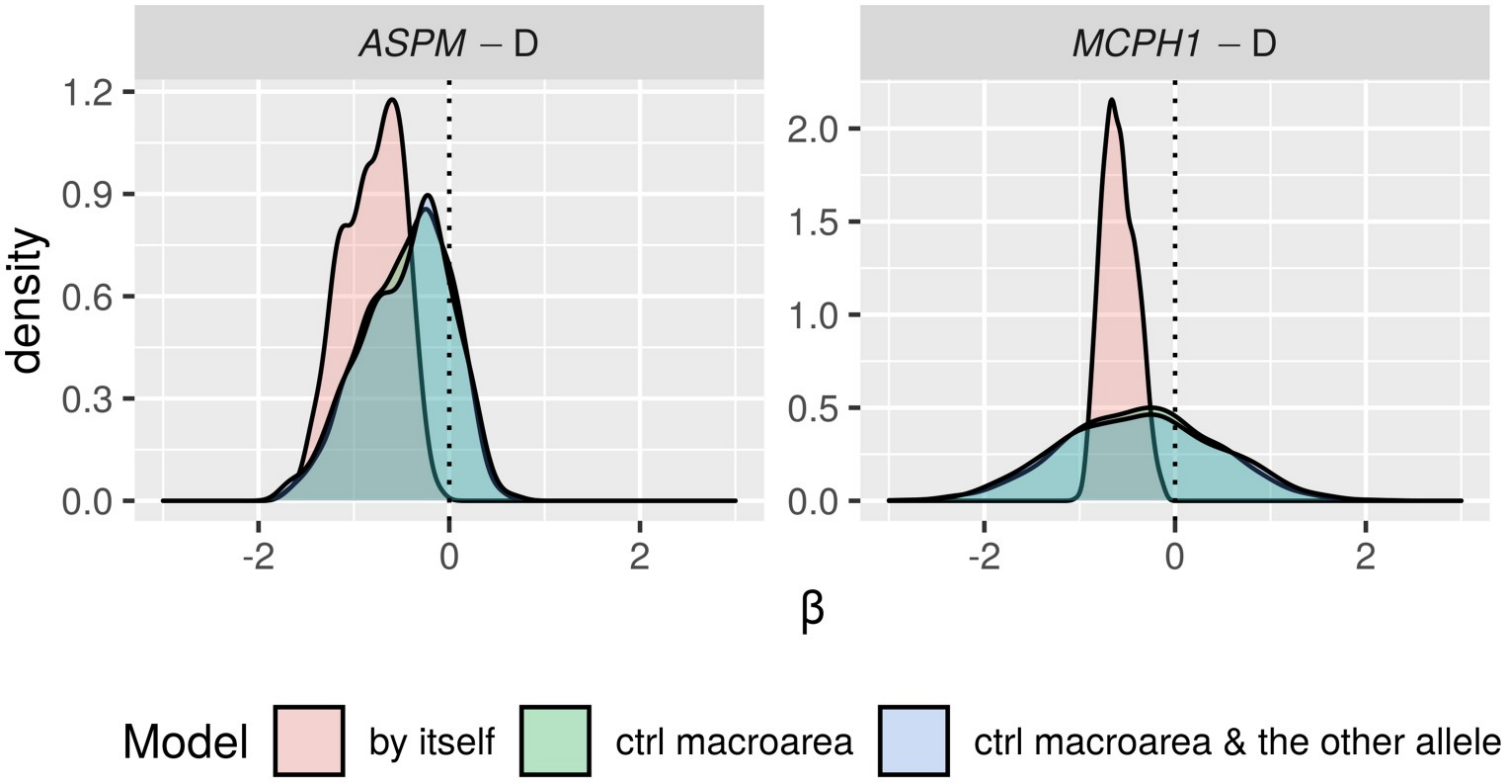
Distribution of the effect size *β* (horizontal axis) of *ASPM*-D (left) and *MCPH1*-D (right) on *tone1* in the restricted sampling regressions without controlling for macroareas (“by itself”, red areas), including macroarea as a fixed effect (“ctrl macroarea”, green areas), and including both macroarea and the other “derived” allele as fixed effects (“ctrl macroarea & the other allele”, blue areas). Each individual plot shows 0.0 (vertical dotted black thin line) and the distribution of the *β*’s for the three “models” (the coloured areas). Please note that the y-axes vary between plots, but the x-axes are fixed for comparability. The more to the left of 0.0 a distribution is, the clearer the negative effect is.

Repeated restricted sampling, where only one sample is randomly chosen per family, also shows a clear negative effect of *ASPM*-D on *tone1* even when controlling for *macroarea* and *MCPH1*-D (≈82% of the *β*’s < 0, with a mean $\bar{\beta }\approx -0.45$), and a weaker but still discernible one for *MCPH1*-D even when controlling for *macroarea* and *ASPM*-D (≈68% of the *β*’s < 0, with a mean $\bar{\beta }\approx -0.37$); see Fig 10.

Fitting a Bayesian mixed-effects logistic regression model of *tone1* on *ASPM*-D, *MCPH1*-D and their interaction as fixed effects, and *macroarea*, *family* and *(meta)population* (nested within family) as random effects, found that the interaction is not needed, but that each “derived” allele seems to have a negative effect on tone. For *ASPM*-D: *β* = −0.69, 89%*HDI* = [−1.59, 0.25], posterior probability *p*(*β* < 0) = 0.89, evidence ratio = 7.9, % HDI inside ROPE = 13.3%, *p*_*ROPE*_ = 0.12; model comparisons to the “null” model (i.e., intercept and confounds-only) suggest that they are roughly equivalent (Bayes Factor = 3.83, LOO = 0.70 [SE = 1.43], WAIC = 0.92 [SE = 1.19], KFOLD = 4.48 [SE = 3.46]). For *MCPH1*-D: *β* = −0.63, 89%*HDI* = [−1.66, 0.46], posterior probability *p*(*β* < 0) = 0.83, evidence ratio = 5.0, % HDI inside ROPE = 15.2%, *p*_*ROPE*_ = 0.14; model comparisons to “null” suggest rough equivalence (Bayes Factor = 3.17, LOO = -0.01 [SE = 1.03], WAIC = 0.45 [SE = 0.77], KFOLD = 2.33 [SE = 2.31]). Please note that the posterior probability for the directional hypothesis *p*(*β* < 0) should be as large as possible (this is the actual probability that the effect is negative) and the associated evidence ratio as large as possible, the % HDI inside ROPE should be as small as possible (a value of 0% meaning the HDI entirely falls outside the ROPE), and that *p*_*ROPE*_ should also be as small as possible (a value of 0.0 meaning that the whole posterior distribution falls entirely outside the ROPE) and can be interpreted somewhat like a frequentist *p*-value by comparing it to a threshold of say, 0.05.

Modelling *macroarea* as a 2D Gaussian Processes in the Bayesian mixed-effects logistic regression model found that the interaction is not needed, but that each “derived” allele seems to have a negative effect on tone. For *ASPM*-D: *β* = −0.88, 89%*HDI* = [−1.63, −0.06], posterior probability *p*(*β* < 0) = 0.96, evidence ratio = 25, % HDI inside ROPE = 3.5%, *p*_*ROPE*_ = 0.065; model comparisons to the “null” model suggest that they are roughly equivalent (Bayes Factor = 1.3, LOO = -0.05 [SE = 2.69], WAIC = -0.69 [SE = 2.74], KFOLD = -1.21 [SE = 3.41]). For *MCPH1*-D: *β* = −1.03, 89%*HDI* = [−1.63, −0.45], posterior probability *p*(*β* < 0) = 0.99, evidence ratio = 170, % HDI inside ROPE = 0%, *p*_*ROPE*_ = 0.011; model comparisons to “null” suggest moderate evidence for *MCPH1*-D (Bayes Factor = 0.3, LOO = -2.13 [SE = 1.73], WAIC = -2.22 [SE = 1.73], KFOLD = -2.02 [SE = 2.70]).

#### Mediation analysis

I ran two main separate mediation analyses (see Fig 5) with the treatment (*IV*) = *dichotomised macroarea* (Africa vs the rest of the world), the outcome (*DV*) = *tone1*, and the *mediator* (*MV*) = either *ASPM*-D or *MCPH1*-D, respectively, as well as the “experimental” Bayesian simultaneous bi-mediated model (see Fig 6) with both *ASPM*-D or *MCPH1*-D as mediators.

Using the full dataset and the “classic” approach, both main models find a significant positive *total effect* (*TE*) of macroarea on tone (*ASPM*-D: 0.49, 95%CI (0.33, 0.63), *p* < 2 ⋅ 10^−16^; *MCPH1*-D: 0.50, 95%CI (0.34, 0.65), *p* < 2 ⋅ 10^−16^), confirming that African languages tend to be more tonal in our database than languages outside Africa, as well as a significant positive *average direct effect* (*ADE*; coefficient *c*′ in Fig 5) of *macroarea* on *tone1* (*ASPM*-D: 0.27, 95%CI (0.08, 0.47), *p* = 0.008; *MCPH1*-D: 0.55, 95%CI (0.19, 0.75), *p* = 0.002). However, the average *indirect effects* (*ACME*), mediated by the “derived” allele frequencies, differ strongly between them: for *ASPM*-D, the mediated effect is significant and positive (0.22, 95%CI (0.11, 0.34), *p* < 2 ⋅ 10^−16^), representing 44.9% of the total effect and resulting from a significant negative effect of being in Africa on *ASPM*-D (coefficient *a*; −1.25 ± 0.16, *p* = 7.7 ⋅ 10^−13^), and a significant negative effect of *ASPM*-D on tone (coefficient *b*; −0.90 ± 0.24, *p* = 0.00015). For *MCPH1*-D, the mediated effect is not significant (−0.05, 95%CI (− 0.22, 0.25), *p* = 0.49), composed of a significant negative effect of being in Africa on *MCPH1*-D (coefficient *a*; −2.19 ± 0.09, *p* = 9.9 ⋅ 10^−59^), and a non-significant effect of *MCPH1*-D on tone (coefficient *b*; 0.20 ± 0.38, *p* = 0.6). Taken together, this shows that there’s a positive effect of being in Africa on tone, but that about half of it is mediated by the negative effect of *ASPM*-D, but not by *MCPH1*-D; moreover, the frequency of *MCPH1*-D is shaped much more by being in or outside Africa than that of *ASPM*-D.

**Fig 11:**
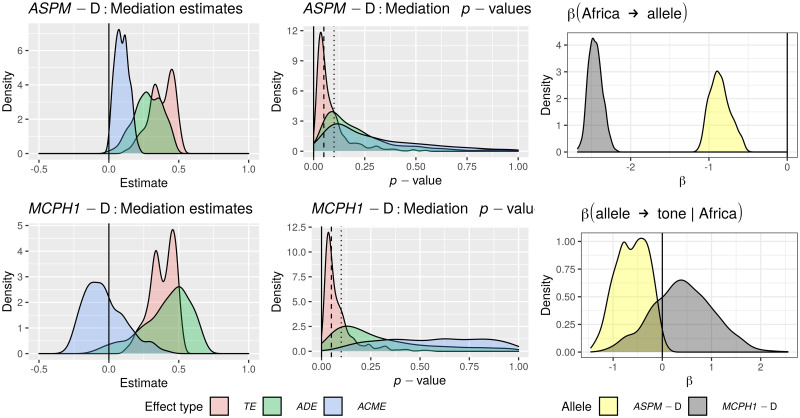
Mediation analysis for 1,000 restricted samples for *tone1*. The leftmost two panels show the distribution of point estimates (coloured areas) of the *Total Effect* (*TE*), the *Direct Effect* (*AD*E) and the *Indirect Effect* (*ACM*E) for *ASPM*-D and *MCPH1*-D; the middle two panels show the distribution of the *p*-values (coloured areas) for the same effects, while the two rightmost panels (on white background) show the distribution of the regression slopes (*β*) for the two alleles (top: for the regression of the allele frequency on being within or outside Africa, and bottom: for the regression of tone on the allele while controlling for being within or outside Africa). The black vertical lines are: 0.0 (solid), 0.05 (dashed) and 0.10 (dotted). Please note that the y-axes vary between plots, but the x-axes of the four left panels (but not of the two right panels) are fixed for comparability. Mediation estimates (leftmost panels): *ACME* (blue) should be away from 0 for a sizeable mediation through the “derived” allele. Mediation *p*-values (mid panels): *ACME* (blue) should be below 0.05 (or 0.10) for a statistically significant mediation through the “derived” allele. (Partial) regression slopes *β* (rightmost panels): should be different from 0. See Table 9 for the numeric summaries.

Restricted sampling (repeated 1,000 times) finds the same pattern (see Fig 11 and Table 9), with only *ASPM*-D mediating part of the influence of macroarea on tone, but not *MCPH1*-D; also, macroarea has a much stronger effect on the latter. This procedure does control for language family, but, on the other hand, drastically reduces the sample size to only *N* = 35 unique families, resulting in a low power of the individual mediation analyses, with relatively few effect sizes being large enough for statistical significance, but even so, there are many more significant indirect effects for *ASPM*-D (10.2% at *α*-level 0.05, and 27.7% at *α*-level 0.10) than for *MCPH1*-D (0.2% and 1.5%, respectively).

**Table 9:** Summaries of the mediation analysis for 1,000 restricted samples for *tone1*. For each “derived” allele the table shows the three types of effects (total, direct and mediated) and the two (partial) regression coefficients. For each, it gives the mean, median and the results of the relevant comparison with 0.0 (smaller or bigger) in terms of the percent of the estimates smaller (or bigger) than 0.0 and the one-sided *t*-test (*df* = 999); the direction of the comparison is based on *a priori* expectancies: positive effects but negative *β*s.

Allele	Estimate	Mean	Median	Compared to 0
*ASPM*-D	*TE*	0.38	0.38	> 0: 100.0%, *t* = 134.2, *p* < 2.2 ⋅ 10^−16^
	*ADE*	0.28	0.28	> 0: 99.6%, *t* = 87.2, *p* < 2.2 ⋅ 10^−16^
	*ACME*	0.094	0.091	> 0: 99.5%, *t* = 61.4, *p* < 2.2 ⋅ 10^−16^
	*β*(Africa → allele)	-0.86	-0.87	<0: 100.0%, *t* = −211.9, *p* < 2.2 ⋅ 10^−16^
	*β*(allele → tone ∣ Africa)	-0.61	-0.60	<0: 99.1%, *t* = −60.0, *p* < 2.2 ⋅ 10^−16^
*MCPH1*-D	*TE*	0.38	0.39	> 0: 100.0%, *t* = 133.1, *p* < 2.2 ⋅ 10^−16^
	*ADE*	0.41	0.44	> 0: 96.9%, *t* = 74.0, *p* < 2.2 ⋅ 10^−16^
	*ACME*	-0.029	-0.052	> 0: 35.7%, *t* = −6.2, *p* = 1
	*β*(Africa → allele)	-2.5	-2.5	<0: 100.0%, *t* = −884.5, *p* < 2.2 ⋅ 10^−16^
	*β*(allele → tone ∣ Africa)	0.42	0.42	<0: 25.5%, *t* = 21.4, *p* = 1

**Fig 12:**
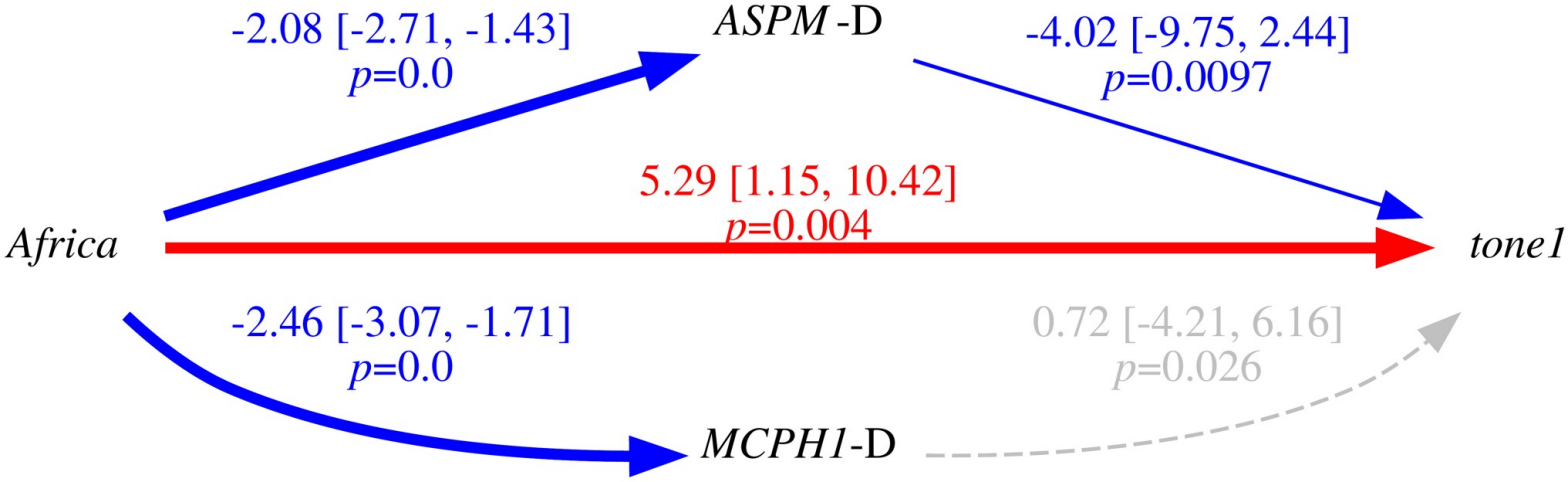
The simultaneous mediation model for *tone1* through both “derived” alleles. Each edge shows the posterior mean and 89%HDI of the partial regressions, and their *p*_*ROPE*_-values; solid thick coloured = effect clearly present, solid thin coloured = effect probably present, gray dashed = probably no effect; red = positive and blue = negative effect. Please note that this controls for *family* and *(meta)populations* as random effects, and that the populations frequencies of the “derived” alleles are not *z*-scored.

The Bayesian approach is more flexible and allows Beta regressions for the (non *z*-scored) “derived” allele frequencies as well as the modelling of language *family* and *(meta)population* as random effects. It also found a positive *TE* (*ASPM*-D: 12.04, 89%HDI [1.36, 24.40], *p*_*ROPE*_ = 0.0005; *MCPH1*-D: 6.10, 89%HDI [−4.57, 16.89], *p*_*ROPE*_ = 0.0075), and a positive *ADE* (*ASPM*-D: 4.57, 89%HDI [1.36, 8.52], *p*_*ROPE*_ = 0.0015; *MCPH1*-D: 5.46, 89%HDI [1.26, 10.13], *p*_*ROPE*_ = 0.00075). However, while there seems to be a positive *ACME* for *ASPM*-D (7.44, 89%HDI [−4.55, 19.85], *p*_*ROPE*_ = 0.0048) resulting from a negative effect of being in Africa on *ASPM*-D (−2.08, 89%HDI [−2.71, −1.42], *p*_*ROPE*_ = 0.0) and a negative effect of *ASPM*-D on tone (−3.75, 89%HDI [−9.43, 1.96], *p*_*ROPE*_ = 0.00925), this is arguably absent for *MCPH1*-D (*ACME*: 0.69, 89%HDI [−10.63, 13.93], *p*_*ROPE*_ = 0.013; negative effect of being in Africa on *MCPH1*-D: −2.45, 89%HDI [−3.10, −1.78], *p*_*ROPE*_ = 0.0; null effect of *MCPH1*-D on tone: −0.30, 89%HDI [−5.02, 4.76], *p*_*ROPE*_ = 0.028). Simultaneously modelling the mediation of both “derived” alleles produces similar results (Fig 12): positive *TE* (11.61, 89%HDI [−2.54, 26.06], *p*_*ROPE*_ = 0.0038) and *ADE* (5.29, 89%HDI [1.15, 10.42], *p*_*ROPE*_ = 0.004), with an arguably positive *ACME* for *ASPM*-D (8.01, 89%HDI [−4.47, 21.47], *p*_*ROPE*_ = 0.0047), resulting from a negative effect of being in Africa on *ASPM*-D (−2.08, 89%HDI [−2.71, −1.43], *p*_*ROPE*_ = 0.0) and a negative effect of *ASPM*-D on tone (−4.02, 89%HDI [−9.75, 2.44], *p*_*ROPE*_ = 0.0097), but much less convincing evidence of an *ACME* for *MCPH1*-D (−1.69, 89%HDI [−15.49, 10.09], *p*_*ROPE*_ = 0.011) resulting from a negative effect of being in Africa on *MCPH1*-D (−2.46, 89%HDI [−3.07, −1.71], *p*_*ROPE*_ = 0.0) and no effect of *MCPH1*-D on tone (0.72, 89%HDI [−4.21, 6.16], *p*_*ROPE*_ = 0.026).

#### Path analysis

I ran path analyses (see Fig 6) with the treatment (*IV*) = *dichotomised macroarea* (Africa vs the rest of the world), the outcome (*DV*) = *tone1*, and the *mediators* *MV*_1_ = *ASPM*-D and *MV*_2_ = *MCPH1*-D, separately for the binary and the ordered codings of the *IV* and the *DV* (but given that the results are extremely similar, I only report here the numeric coding).

**Fig 13:**
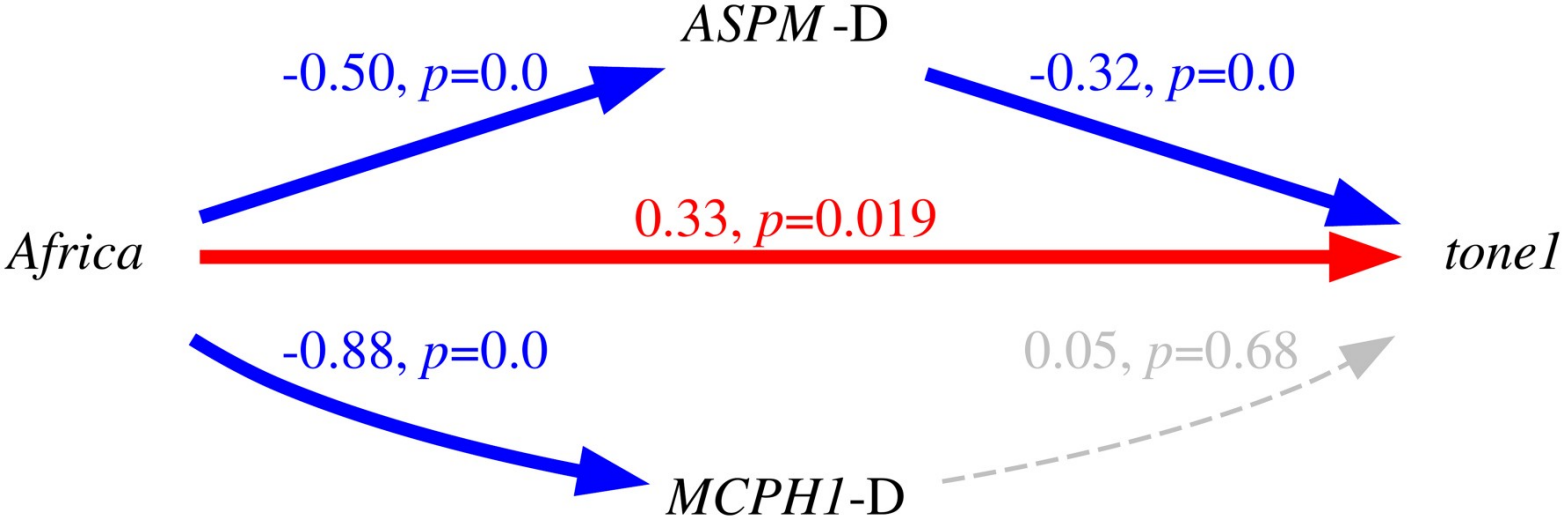
The path model for *tone1* (numeric coding) fitted on all the data. Each edge shows the standardized path coefficients and their *p*-values; gray dotted = not significant, red = significant positive, and blue = significant negative.

Using the full dataset, the model fits the data very well ($\chi_{1}^{2}=0.22$, *p* = 0.64; *CFI* = 1.00, *TLI* = 1.01, *NNFI* = 1.01 and *RFI* = 1.00), and, as shown in Fig 13, being in Africa has a significant positive direct effect on *tone1*, a significant negative effect on *ASPM*-D which has a negative significant effect on *tone1*, but while it has a stronger significant negative effect on *MCPH1*-D this has no effect on *tone1*. Please note that, for path analyses/SEM models, we want the *χ*^2^ goodness-of-fit test to be *not* significant, meaning that there is no reason to reject the hypothesis that the model fits the data; this is a binary decision. On the other hand, there are several *fit indices* (I only show a few: *CFI*, *TLI*, *NNFI* and *RFI*), which are continuous, the closer to 1.00, the better the model fits to the data.

**Fig 14:**
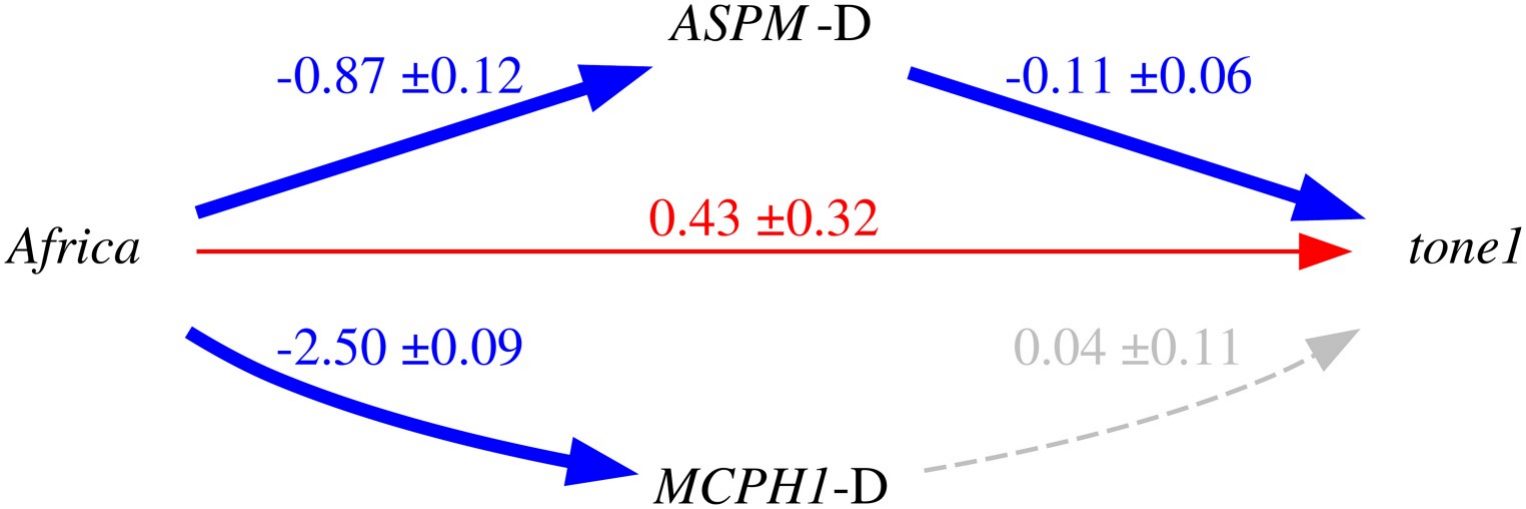
Summary of the 1,000 path models for *tone1* fitted to restricted samples. Each edge shows the means of the standardized path coefficients and ± their standard deviation; conventions as in Fig 13.

As for the mediation analysis above, restricted sampling finds a similar pattern (see Fig 14 and Table 10).

**Table 10:** Summaries of the path analysis for 1,000 restricted samples for *tone1*. Similar conventions as for Table 9.

Type	Estimate	Mean (SD)	Median (IQR)	Compared to 0
**Fit**	*χ*^2^ test	94.7% n.s.	-	-
	*CFI*	0.99 (0.01)	1.00 (0.02)	-
	*TLI*	0.97 (0.10)	0.99 (0.16)	-
	*NNFI*	0.97 (0.10)	0.99 (0.16)	-
	*RFI*	0.90 (0.09)	0.92 (0.14)	-
**Path**	Africa → *tone1*	0.43 (0.32)	0.44 (0.46)	89.5% > 0
	Africa → *ASPM*-D	−0.87 (0.12)	−0.89 (0.17)	100.0% < 0
	*ASPM*-D → *tone1*	−0.11 (0.06)	−0.11 (0.09)	98.6% < 0
	Africa → *MCPH1*-D	−2.5 (0.09)	−2.5 (0.12)	100.0% < 0
	*MCPH1*-D → *tone1*	0.04 (0.11)	0.04 (0.17)	36.7% < 0

#### Decision trees

**Fig 15:**
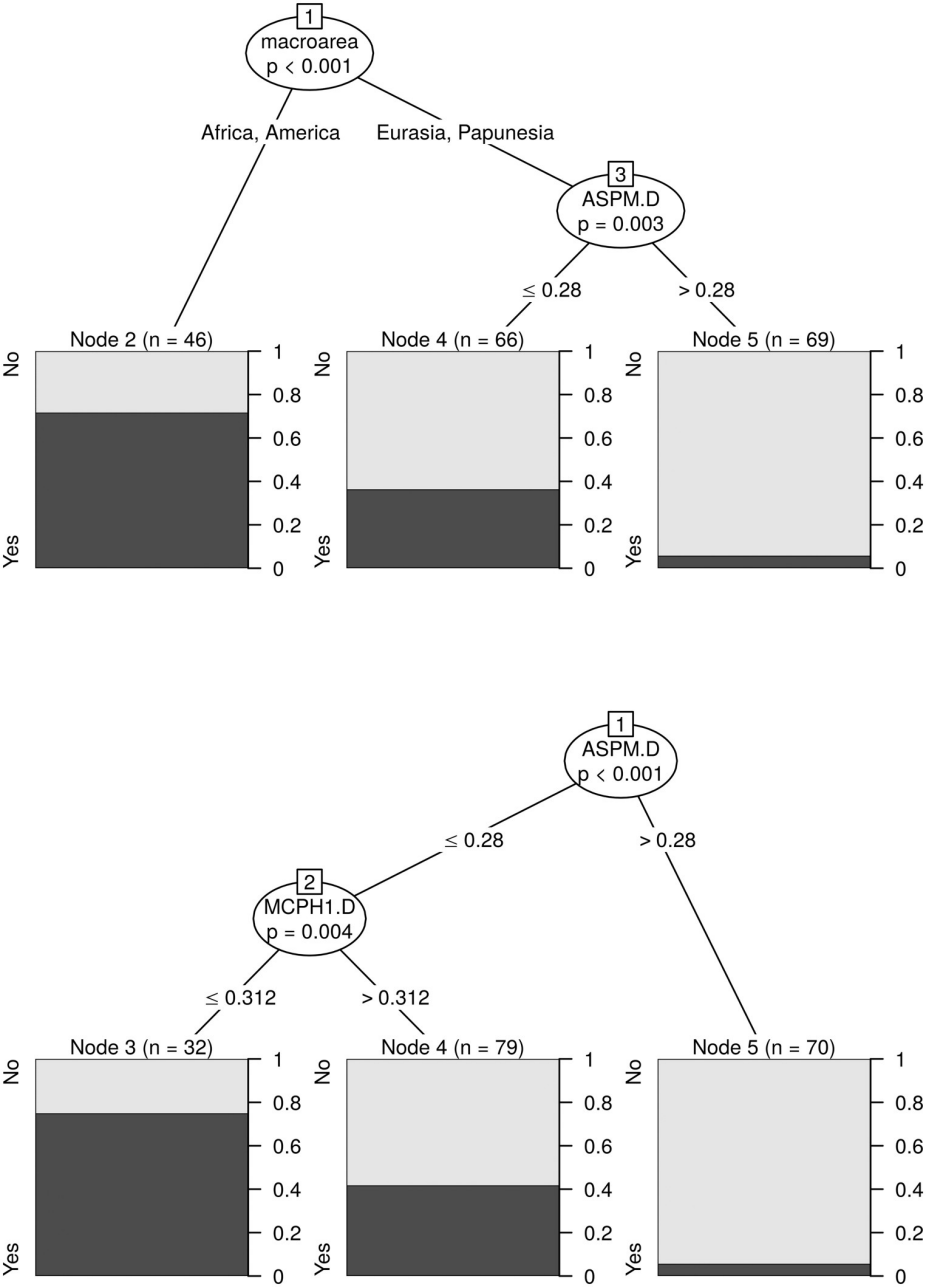
Decision trees for *tone1* including *macroarea* as a predictor (top) or not (bottom). Please note that in these decision tree plots “ASPM.D” and “MCPH1.D” stand for *ASPM*-D and *MCPH1*-D, respectively. Each split is a binary decision based on the values or conditions on the branches (with the *p*-value in parentheses). The bars at the bottom of the plots show the distribution of “No” (light gray’) and “Yes” (dark gray) values and the number of observations for that case (e.g., *n* = 69 in the top panel’s rightmost bar, means that 69 observations are in Eurasia and Papunesia with the population frequency of *ASPM*-D > 0.28, and for those only a minority of ≈6% have tone). It can be seen that in the bottom panel, a low frequency of *MCPH1*-D seems to be a proxy for Africa.

If, besides *ASPM*-D and *MCPH1*-D, *macroarea* is also included, the decision tree fits the full dataset well (accuracy = 77.3%, sensitivity = 71.7%, specificity = 79.3%, precision = 54.1%, and recall = 71.7%), and generalises across 100 training/testing sets (mean ± sd: accuracy = 77.1% ±6.6%, sensitivity = 71.6% ±15.2%, specificity = 79.3% ±6.8%, precision = 52.9% ±12.4%, recall = 71.6% ±15.2%), and suggests that within Eurasia and Papunesia, the frequency of *ASPM*-D is a good predictor of *tone1* (with *p* = 0.003). When *macroarea* is not included, then the fit for the whole dataset drops a bit (accuracy = 75.1%, sensitivity = 75.0%, specificity = 75.2%, precision = 39.3%, and recall = 75.0%) and generalises slightly less well (accuracy = 70.1% ±7.6%, sensitivity = 61.3% ±19.1%, specificity = 76.2% ±8.7%, precision = 44.0% ±23.0%, recall = 61.3% ±19.1%); now, *ASPM*-D is predictive overall (*p* < 0.001), and *MCPH1*-D is relevant (*p* = 0.004) only for low frequencies of *ASPM*-D (see Fig 15).

#### Random forests

If *macroarea* is included as a predictor, the random forests fit the data well (accuracy = 77.7% ±0.8%, sensitivity = 68.9% ±1.9%, specificity = 81.6% ±0.4%, precision = 62.0% ±1.0%, recall = 68.9% ±1.9%), and the conditional random forests are even better (accuracy = 84.3% ±0.7%, sensitivity = 78.8% ±0.5%, specificity = 86.8% ±0.9%, precision = 73.0% ±2.0%, recall = 78.8% ±0.5%). Of the 3 measures of variable importance, the mean decrease in accuracy for the random forests finds that *macroarea* > *ASPM*-D > *MCPH1*-D, the mean decrease in node impurity for the random forests finds that *ASPM*-D > *MCPH1*-D > *macroarea*, and the unconditional importance for the conditional random forests finds that *ASPM*-D > *macroarea* > *MCPH1*-D.

Leaving macroarea out slightly reduces the fit (random forests: accuracy = 70.7% ±1.0%, sensitivity = 56.3% ±1.4%, specificity = 78.6% ±0.8%, precision = 59.0% ±1.8%, recall = 56.3% ±1.4%; conditional random forests: accuracy = 82.1% ±0.5%, sensitivity = 81.8% ±0.7%, specificity = 82.3% ±0.6%, precision = 60.5% ±1.6%, recall = 81.8% ±0.7%), but now all three criteria agree that *ASPM*-D > *MCPH1*-D.

#### Summary

Thus, *tone1*, capturing the presence of any type of tone system, is negatively influenced by each of the two “derived” alleles by themselves (i.e., when not controlling for shared inheritance and contact), while controlling for these confounds still finds these negative influences but they are now much weaker. (Interestingly, when contact is modelled in a continuous manner, using a 2D Gaussian Process, then these effects are stronger than when modelling it as a standard random effect). The strengths of the effects of the two “derived” alleles are comparable and are affected in similar ways by controlling for the confounds. However, when properly modelling the difference between Africa and the rest of the world in allele frequencies and tone I found that only *ASPM*-D has a negative effect on tone above and beyond that due to macroarea and language family, suggesting that the frequency of *ASPM*-D partially mediates the influence of macroarea on tone. Thus, there seems to be a weak negative effect of *ASPM*-D on the presence/absence of tone above and beyond the confounding effects of contact and shared inheritance, but less convincingly for *MCPH1*-D.

### Is there complex tone? (*tone2*)

While the original hypothesis in [9] concerns strictly the presence of tone, here I capitalise on the availability of data allowing the comparison of languages with a complex tone system versus those without tone or with a simple system—this is embodied in the *tone2* variable, where “Yes” represents complex tone systems.

**Fig 16:**
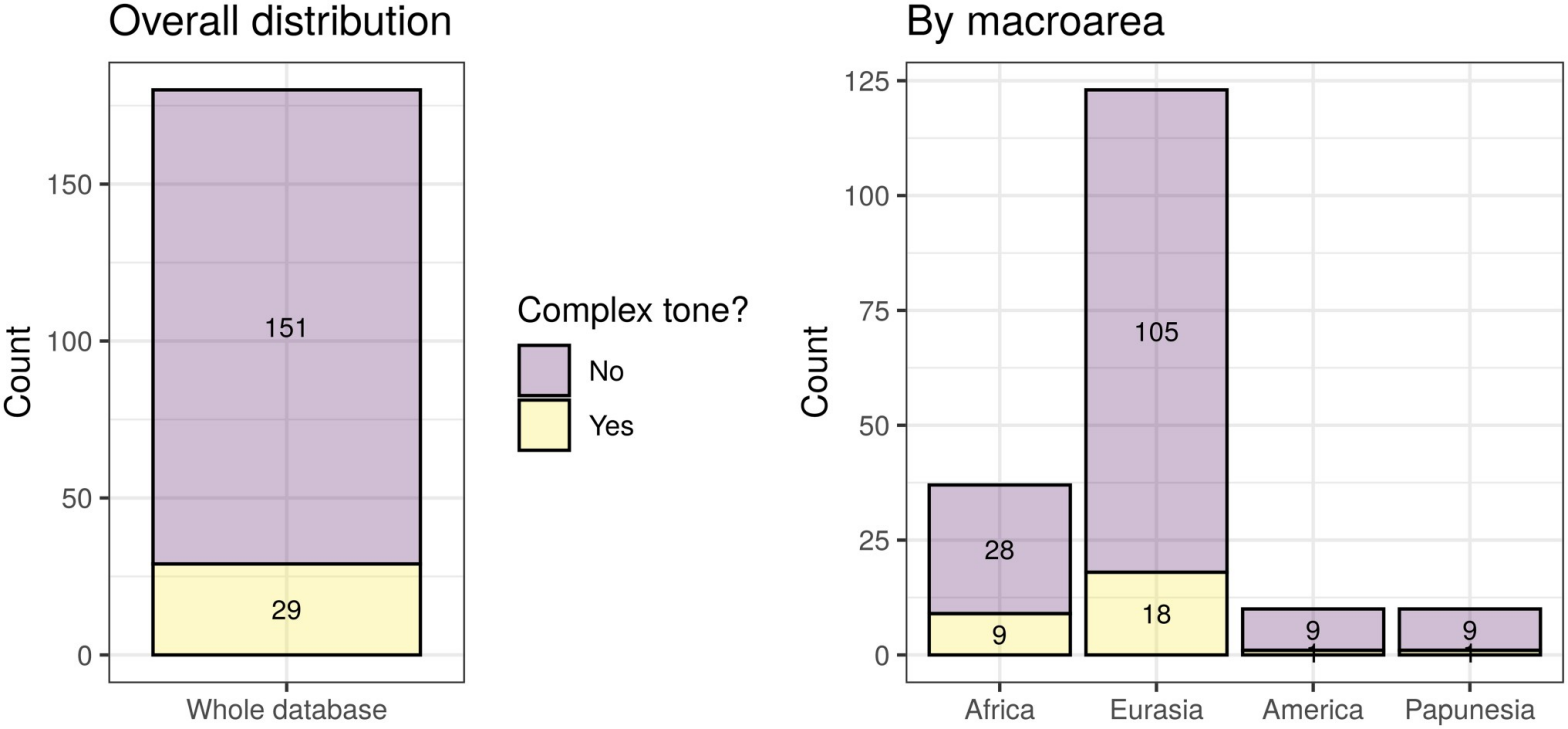
Distribution (as counts) of *tone2* (no and simple tone vs complex tone) across the full database (left) and by macroarea (right). Same conventions as in Fig 7.

When selecting all unique observations with non-missing data for *tone2*, *ASPM*-D and *MCPH1*-D, there are 180 observations, distributed among 118 unique Glottolog codes (languages) in 35 families (the number of languages per family ranges from 1 to 47, with mean 5.1 and median 2). There are 29 (16.1%) languages with complex tone (“Yes”) in the dataset ($\chi_{1}^{2}=82.7$, *p* < 2.2 ⋅ 10^−16^), and the distribution by macroarea is 37 (9 = 24.3% “Yes”) in Africa, 123 (18 = 14.6%) in Eurasia, 10 (1 = 10%) in America, and 10 (1 = 10%) in Papunesia (between macroareas $\chi_{3}^{2}=2.6$, *p* = 0.46); see Fig 16.

**Fig 17:**
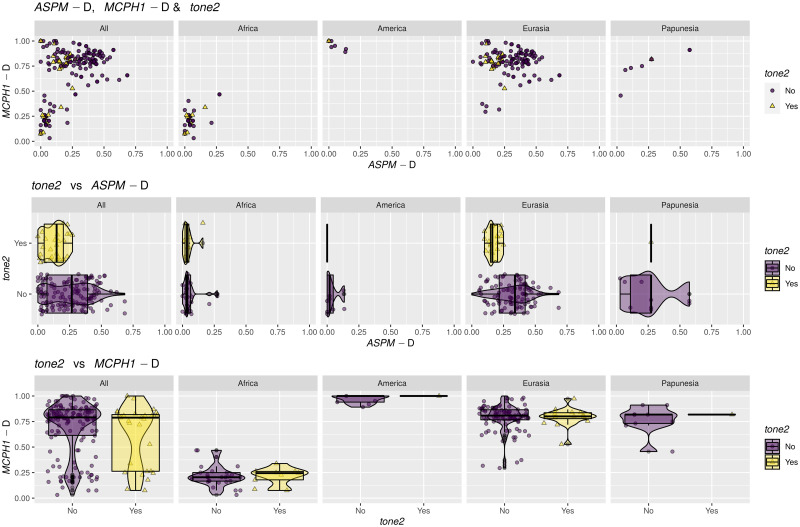
Relationship between tone2 and the population frequency of *ASPM*-D and *MCPH1*-D in the whole database and separately by macroarea. Same conventions as in Fig 8.

The relationship between *tone2* and the population frequency of the two “derived” alleles is shown in Fig 17. It can seen that, globally, it seems that languages with complex tone tend to be found when *ASPM*-D has a low frequency, which seems to be the case also in Eurasia.

#### Regressions

On the whole data and using the “classic” approach, *tone2* is almost completely clustered within families (*ICC*(*tone*2) = 95.6%). The interaction between *ASPM*-D and *MCPH*-D does not contribute (*p* = 0.76), *macroarea* does not predict *tone2* (*p* = 0.50, *R*^2^ = 2.0%), and neither do the two “derived” alleles individually (*ASPM*-D: *β* = −0.87 ± 0.69, *p* = 0.19, *R*^2^ = 1.3%; *MCPH1*-D *β* = −1.01 ± 0.73, *p* = 0.16, *R*^2^ = 1.5%).

**Fig 18:**
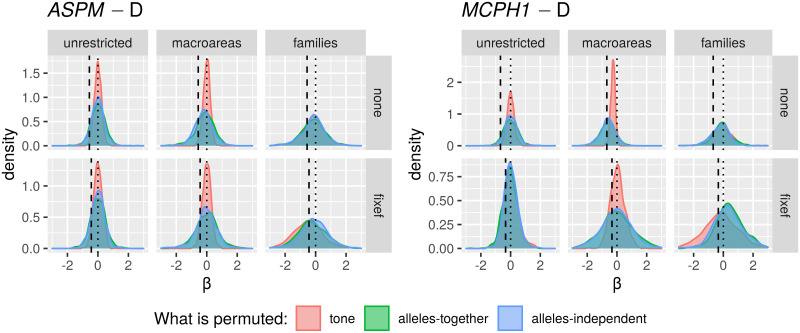
Distribution of the permutation regressions for *tone2*. Same conventions as in Fig 9.

Permuting the observations (Fig 18 and Table 8), shows that *ASPM*-D has a negative effect on *tone2* in all conditions, less clear when restricting the permutations within families, but *MCPH1*-D seems to have a negative effect only for unrestricted permutations when not controlling for *macroarea*.

**Fig 19:**
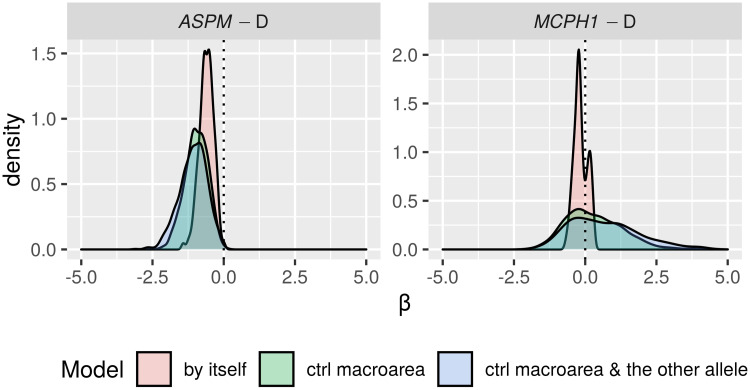
Distribution of the restricted sampling regressions for *tone2*. Same conventions as in Fig 10.

Repeated restricted sampling also shows a clear negative effect of *ASPM*-D on *tone2* even when controlling for *macroarea* and *MCPH1*-D (99.9% of the *β*’s <0, with a mean $\bar{\beta }=-0.96$), and arguably no effect for *MCPH1*-D (35.1% of the *β*’s <0, with a mean $\bar{\beta }=0.67$); see Fig 19.

Fitting a Bayesian mixed-effects logistic regression model of *tone2* on *ASPM*-D, *MCPH1*-D and their interaction as fixed effects, and *macroarea*, *family* and *(meta)population* (nested within family) as random effects, found that the interaction is not needed, but that each “derived” allele seems to have a negative effect on tone. For *ASPM*-D: *β* = −1.27, 89%*HDI* = [−2.73, 0.17], posterior probability *p*(*β* < 0) = 0.93, evidence ratio = 14, % HDI inside ROPE = 6.1%, *p*_*ROPE*_ = 0.055; model comparisons to the “null” model suggest that they are roughly equivalent (Bayes Factor = 1.46, LOO = 1.84 [SE = 1.14], WAIC = 0.10 [SE = 1.16], KFOLD = -0.80 [SE = 2.10]). For *MCPH1*-D: *β* = −0.91, 89%*HDI* = [−2.38, 0.58], posterior probability *p*(*β* < 0) = 0.85, evidence ratio = 5.5, % HDI inside ROPE = 10.9%, *p*_*ROPE*_ = 0.097; model comparisons to “null” suggest rough equivalence (Bayes Factor = 1.63, LOO = 0.25 [SE = 0.64], WAIC = -0.36 [SE = 0.73], KFOLD = -2.43 [SE = 1.67]). Modelling *macroarea* as a 2D Gaussian Processes in the Bayesian mixed-effects logistic regression model found that the interaction is not needed, but that each “derived” allele seems to have a negative effect on tone. For *ASPM*-D: *β* = −1.15, 89%*HDI* = [−2.12, −0.16], posterior probability *p*(*β* < 0) = 0.97, evidence ratio = 33, % HDI inside ROPE = 0.6%, *p*_*ROPE*_ = 0.044; model comparisons to the “null” model suggest anecdotal evidence for *ASPM*-D (Bayes Factor = 0.77, LOO = -0.34 [SE = 1.58], WAIC = -0.34 [SE = 1.59], KFOLD = -0.07 [SE = 1.64]). For *MCPH1*-D: *β* = −0.63, 89%*HDI* = [−1.27, −0.04], posterior probability *p*(*β* < 0) = 0.95, evidence ratio = 19, % HDI inside ROPE = 6.5%, *p*_*ROPE*_ = 0.099; model comparisons to “null” suggest that they are roughly equivalent (Bayes Factor = 2.14, LOO = 0.06 [SE = 1.58], WAIC = 0.19 [SE = 1.58], KFOLD = 0.88 [SE = 1.88]).

#### Mediation analysis

Using the full dataset and the “classic” approach, only for *ASPM*-D there is a hint of a *total effect* (*TE*) of macroarea on tone (0.14, 95%CI (−0.01, 0.30), *p* = 0.078), but not for *MCPH1*-D (0.11, 95%CI (−0.02, 0.27), *p* = 0.12). This positive *TE* for *ASPM*-D is decomposed non-significant *ADE* of *macroarea* on *tone2* (−0.05, 95%CI (−0.20, 0.10), *p* = 0.43) and a positive *ACME* (0.19, 95%CI (0.08, 0.31), *p* = 0.004), resulting from a significant negative effect of being in Africa on *ASPM*-D (−1.34 ± 0.16, *p* = 3.7 ⋅ 10^−15^) and a significant negative effect of *ASPM*-D on tone (−1.03 ± 0.31, *p* = 0.0011).

**Fig 20:**
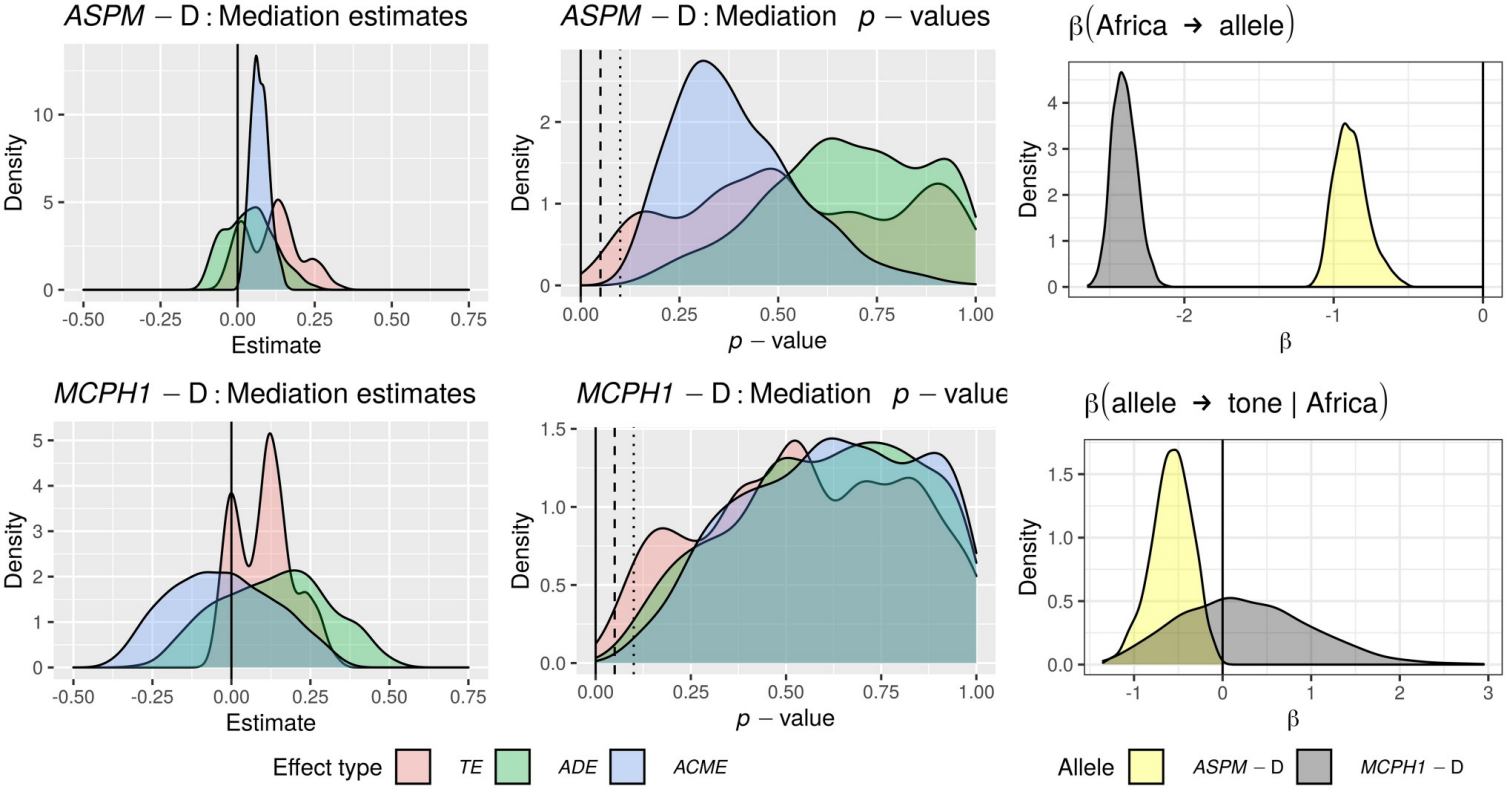
Mediation analysis for 1,000 restricted samples for *tone2*. Same conventions as in Fig 11. See Table 11 for the numeric summaries.

Restricted sampling (Fig 20 and Table 11), finds that while for both “derived” alleles there are positive and comparably strong total (*TE*) and direct (*ADE*) effects, only *ASPM*-D shows a positive indirect effect (*ACME*), but pretty much none of these effects are individually significant.

**Table 11:** Summaries of the mediation analysis for 1,000 restricted samples for *tone2*. Same conventions as in Table 9.

Allele	Estimate	Mean	Median	Compared to 0
*ASPM*-D	*TE*	0.11	0.12	> 0: 89.6%, *t* = 39.7, *p* < 2.2 ⋅ 10^−16^
	*ADE*	0.04	0.039	> 0: 67.3%, *t* = 16.0, *p* < 2.2 ⋅ 10^−16^
	*ACME*	0.072	0.07	> 0: 100.0%, *t* = 78.5, *p* < 2.2 ⋅ 10^−16^
	*β*(Africa → allele)	-0.88	-0.89	<0: 100.0%, *t* = −249.8, *p* < 2.2 ⋅ 10^−16^
	*β*(allele → tone ∣ Africa)	-0.58	-0.57	<0: 100.0%, *t* = −79.8, *p* < 2.2 ⋅ 10^−16^
*MCPH1*-D	*TE*	0.11	0.11	> 0: 82.1%, *t* = 37.1, *p* < 2.2 ⋅ 10^−16^
	*ADE*	0.13	0.14	> 0: 75.0%, *t* = 24.8, *p* < 2.2 ⋅ 10^−16^
	*ACME*	-0.028	-0.034	> 0: 43.0%, *t* = −5.4, *p* = 1
	*β*(Africa → allele)	-2.4	-2.4	<0: 100.0%, *t* = −927.9, *p* < 2.2 ⋅ 10^−16^
	*β*(allele → tone ∣ Africa)	0.26	0.21	<0: 38.0%, *t* = 11.3, *p* = 1

**Fig 21:**
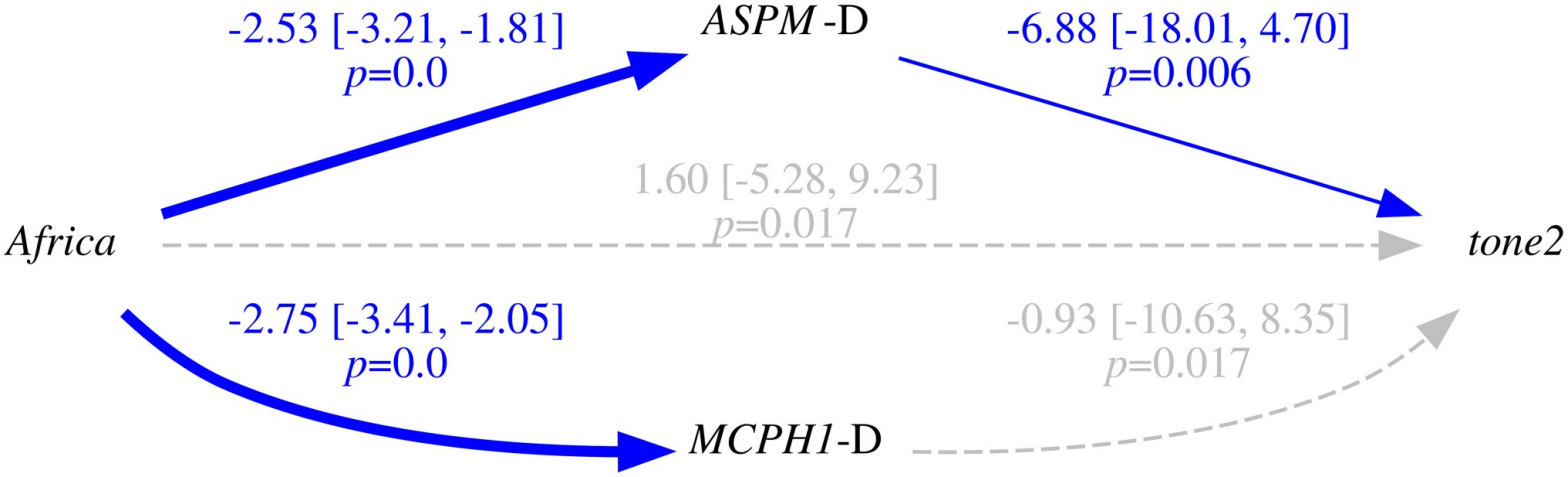
The simultaneous mediation model for *tone2* through both “derived” alleles. Same conventions as in Fig 12.

The Bayesian approach found a possible positive *TE* for *ASPM*-D (17.36, 89%HDI [−5.13, 44.06], *p*_*ROPE*_ = 0.0023) and less clear for *MCPH1*-D (6.72, 89%HDI [−11.75, 26.79], *p*_*ROPE*_ = 0.006), but arguably no *ADE*’s (*ASPM*-D: 1.86, 89%HDI [−2.03, 6.53], *p*_*ROPE*_ = 0.027; *MCPH1*-D: 2.37, 89%HDI [−3.92, 8.28], *p*_*ROPE*_ = 0.021). However, while there seem to be a positive *ACME* for *ASPM*-D (15.46, 89%HDI [−9.45, 42.77], *p*_*ROPE*_ = 0.0023) resulting from a negative effect of being in Africa on *ASPM*-D (−2.54, 89%HDI [−3.29, −1.87], *p*_*ROPE*_ = 0.0) and a negative effect of *ASPM*-D on tone (−6.25, 89%HDI [−16.86, 3.41], *p*_*ROPE*_ = 0.008), this is arguably absent for *MCPH1*-D (*ACME*: 4.56, 89%HDI [−18.69, 28.28], *p*_*ROPE*_ = 0.006; negative effect of being in Africa on *MCPH1*-D: −2.75, 89%HDI [−3.41, −2.09], *p*_*ROPE*_ = 0.0; null effect of *MCPH1*-D on tone: −1.70, 89%HDI [−10.10, 6.58], *p*_*ROPE*_ = 0.016). Simultaneously modelling the mediation of both “derived” alleles produces similar results (Fig 21): positive *TE* (21.02, 89%HDI [−9.08, 56.84], *p*_*ROPE*_ = 0.0027) and *ADE* (1.60, 89%HDI [−5.28, 9.23], *p*_*ROPE*_ = 0.017), with an arguably positive *ACME* for *ASPM*-D (17.13, 89%HDI [−11.56, 46.58], *p*_*ROPE*_ = 0.0023), resulting from a negative effect of being in Africa on *ASPM*-D (−2.53, 89%HDI [−3.21, −1.81], *p*_*ROPE*_ = 0.0) and a negative effect of *ASPM*-D on tone (−6.88, 89%HDI [−18.01, 4.70], *p*_*ROPE*_ = 0.006), but much less convincing evidence of an *ACME* for *MCPH1*-D (2.51, 89%HDI [−23.45, 28.80], *p*_*ROPE*_ = 0.006) resulting from a negative effect of being in Africa on *MCPH1*-D (−2.75, 89%HDI [−3.41, −2.05], *p*_*ROPE*_ = 0.0) and no effect of *MCPH1*-D on tone (−0.93, 89%HDI [−10.63, 8.35], *p*_*ROPE*_ = 0.017).

#### Path analysis

**Fig 22:**
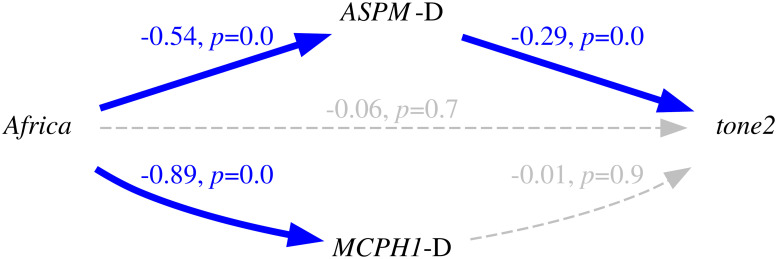
The path model for *tone2* fitted on all the data. Same conventions as in Fig 13.

**Fig 23:**
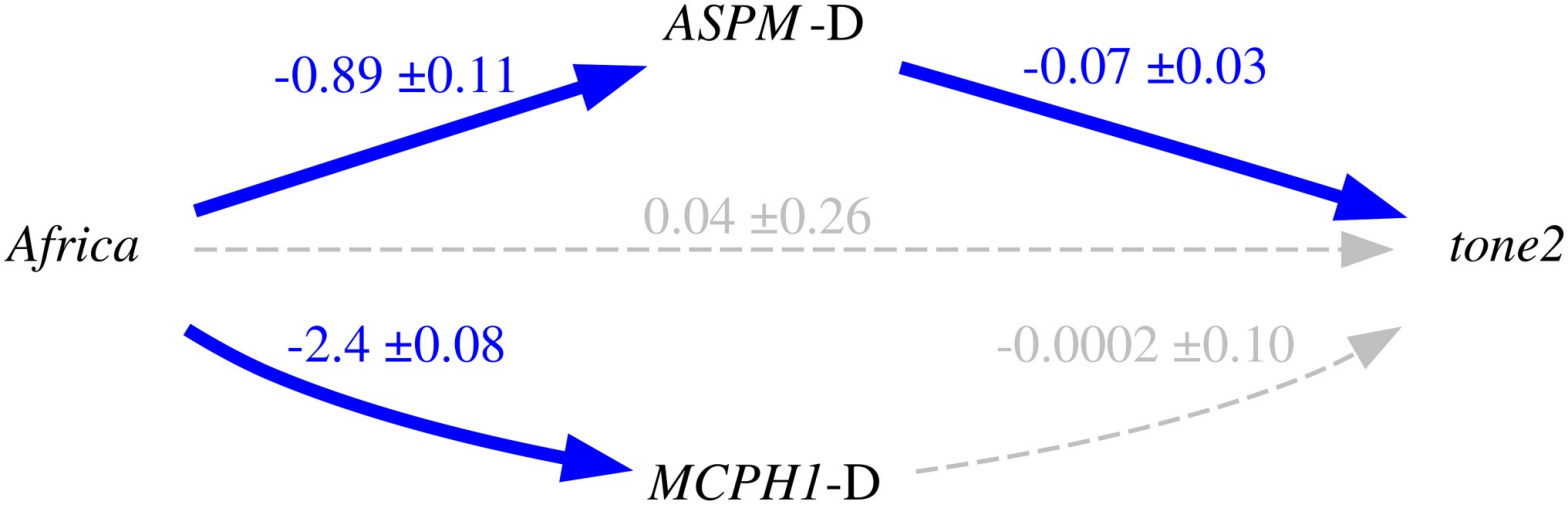
Summary of the 1,000 path models for *tone2* fitted to restricted samples. Same conventions as in Fig 14.

As above, I only report here the numeric coding. On the full dataset, the model fits the data very well ($\chi_{1}^{2}=0.36$, *p* = 0.55; *CFI* = 1.00, *TLI* = 1.01, *NNFI* = 1.01 and *RFI* = 0.99). As shown in Fig 22, being in Africa has no direct effect on complex tone, but has a significant negative effect on *ASPM*-D which has a negative significant effect on complex tone; however, while it has a stronger significant negative effect on *MCPH1*-D, this has no effect on complex tone. The same pattern is also found by restricted sampling, except that here there is also a hint of a negative effect of *MCPH1*-D on complex tone (see Fig 23 and Table 12).

**Table 12:** Summaries of the path analysis for 1,000 restricted samples for *tone2*. Similar conventions as for Table 9.

Type	Estimate	Mean (SD)	Median (IQR)	Compared to 0
**Fit**	*χ*^2^ test	97.6% n.s.	-	-
	*CFI*	0.99 (0.01)	1.00 (0.01)	-
	*TLI*	0.98 (0.10)	0.99 (0.15)	-
	*NNFI*	0.98 (0.10)	0.99 (0.15)	-
	*RFI*	0.90 (0.09)	0.92 (0.13)	-
**Path**	Africa → *tone2*	0.04 (0.26)	0.02 (0.37)	53.6% > 0
	Africa → *ASPM*-D	−0.89 (0.11)	−0.90 (0.15)	100.0% < 0
	*ASPM*-D → *tone2*	−0.07 (0.03)	−0.07 (0.04)	99.8% < 0
	Africa → *MCPH1*-D	−2.40 (0.08)	−2.40 (0.11)	100.0% < 0
	*MCPH1*-D → *tone2*	−0.0002 (0.10)	−0.0016 (0.14)	50.8% < 0

#### Decision trees

The decision trees including or excluding macroarea are the same and trivial in that they uniformly predict just the majority value “No” and have only one split depending on the frequency of *ASPM*-D (≤ or > ≈ 0.27, *p* < 0.001).

#### Random forests

If *macroarea* is included as a predictor, the random forests fit the data well (accuracy = 84.2% ±0.5%, sensitivity = 58.4% ±11.2%, specificity = 84.9% ±0.4%, precision = 8.8% ±2.6%, recall = 58.4% ±11.2%), and the conditional random forests are even better (accuracy = 87.2% ±0.7%, sensitivity = 97.5% ±4.8%, specificity = 86.9% ±0.7%, precision = 21.2% ±5.2%, recall = 97.5% ±4.8%), and two measures of variable importance (mean decrease in accuracy and mean decrease in the Gini index) find that *ASPM*-D > *MCPH1*-D > *macroarea*, while the third (unconditional importance) finds that *ASPM*-D > *macroarea* > *MCPH1*-D.

Leaving macroarea out has very little effect on the fit (random forests: accuracy = 82.2% ±1.0%, sensitivity = 42.8% ±4.4%, specificity = 87.3% ±0.6%, precision = 30.3% ±3.5%, recall = 42.8% ±4.4%; conditional random forests: accuracy = 87.3% ±0.2%, sensitivity = 81.2% ±3.1%, specificity = 87.7% ±0.0%, precision = 27.6% ±0.0%, recall = 81.2% ±3.1%), and all three criteria agree that *ASPM*-D > *MCPH1*-D.

#### Summary

Complex tone is much rarer than no tone and simple tone, so that the distribution of *tone2* is very skewed, and the results much less clear-cut than for *tone1*, but it seems that there is also a negative effect of *ASPM*-D on complex tone (while for *MCPH1*-D the evidence is much more ambiguous).

### Tone *counts*

**Fig 24:**
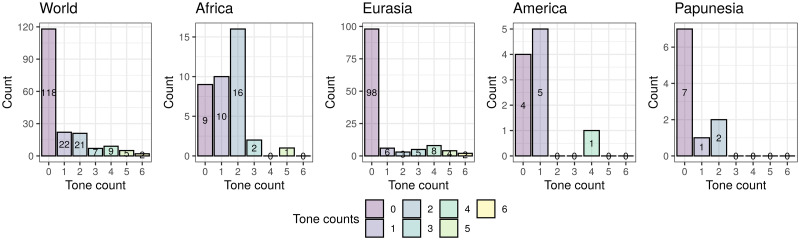
Distribution of tone *counts* across the full database (left-most panel) and by macroarea (the following 4 panels). Same conventions as in Fig 7.

There are 184 unique observations with non-missing data for tone *counts*, *ASPM*-D and *MCPH1*-D, distributed among 121 unique Glottolog codes (languages) in 35 families (the number of languages per family ranges from 1 to 47, with mean 5.3 and median 2); see Fig 24.

**Fig 25:**
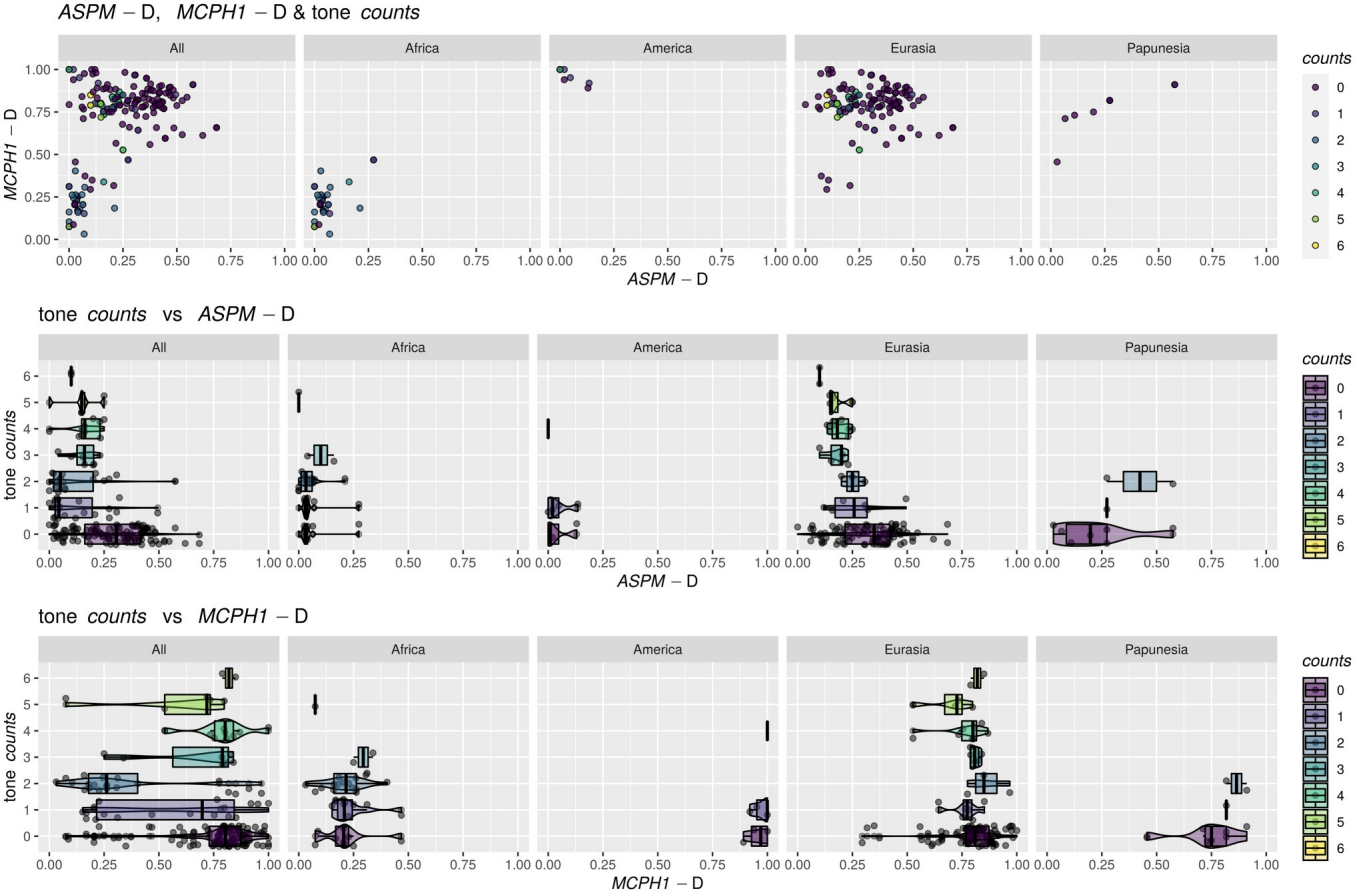
Relationship between tone *counts* and the population frequency of *ASPM*-D and *MCPH1*-D in the whole database and separately by macroarea. Same conventions as in Fig 8.

The relationship between the tone *counts* and the population frequency of the two “derived” alleles is shown in Fig 25. It can seen that, globally, languages with higher tone counts tend to be found when *ASPM*-D has a low frequency, but this does not seem to be a linear relationship.

#### Regressions

On the whole data and using the “classic” approach, the tone *counts* are strongly clustered within families (*ICC*(*tone*2) = 65.3%). The *macroarea* predicts the tone *counts* (*p* = 0.013, *R*^2^ = 23.8%), and while *ASPM*-D has no significant effect by itself (−0.37 ± 0.19, *p* = 0.061, *R*^2^ = 7.6%), *MCPH1*-D seemingly does (−0.46 ± 0.19, *p* = 0.016, *R*^2^ = 9.9%), but this seems to fully overlap with that of *macroarea*.

**Fig 26:**
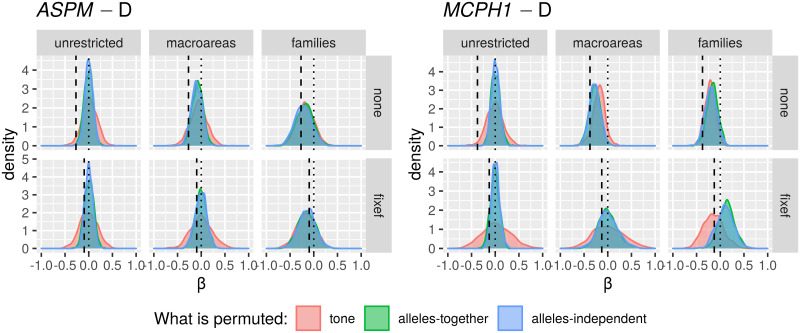
Distribution of the permutation regressions for tone *counts*. Same conventions as in Fig 9.

Permuting the observations (Fig 26 and Table 8), shows similar patterns for both alleles, with a negative effect on tone *counts* only when not controlling for *macroarea* and *family*.

**Fig 27:**
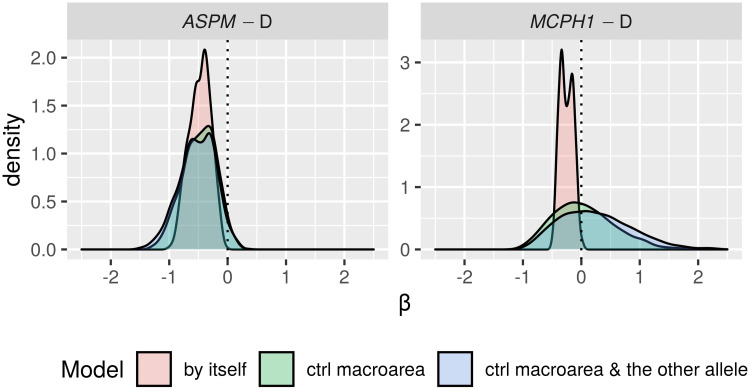
Distribution of the restricted sampling regressions for tone *counts*. Same conventions as in Fig 10.

Repeated restricted sampling shows a clear negative effect of *ASPM*-D on tone *counts* even when controlling for *macroarea* and *MCPH1*-D (96.7% of the *β*’s <0, with a mean $\bar{\beta }=-0.48$), and arguably no effect for *MCPH1*-D (38.2% of the *β*’s <0, with a mean $\bar{\beta }=0.05$); see Fig 27.

Fitting a Bayesian mixed-effects Poisson regression model of tone *counts* on *ASPM*-D, *MCPH1*-D and their interaction as fixed effects, and *macroarea*, *family* and *(meta)population* (nested within family) as random effects, found that the interaction is not needed, but that there might be a negative effect on tone for each “derived” allele. For *ASPM*-D: *β* = −0.25, 89%*HDI* = [−0.64, 0.17], posterior probability *p*(*β* < 0) = 0.93, evidence ratio = 5.2, % HDI inside ROPE = 21%, *p*_*ROPE*_ = 0.19; model comparisons to the “null” model suggest that they are roughly equivalent (Bayes Factor = 4.35, LOO = 1.00 [SE = 1.31], WAIC = 0.89 [SE = 0.89], KFOLD = 4.18 [SE = 3.39]). For *MCPH1*-D: *β* = −0.24, 89%*HDI* = [−0.65, 0.25], posterior probability *p*(*β* < 0) = 0.80, evidence ratio = 4, % HDI inside ROPE = 20.4%, *p*_*ROPE*_ = 0.18; model comparisons to “null” suggest rough equivalence (Bayes Factor = 6.59, LOO = 1.00 [SE = 0.83], WAIC = 0.63 [SE = 0.61], KFOLD = 1.49 [SE = 1.40]). Modelling *macroarea* as a 2D Gaussian Processes in the Bayesian mixed-effects logistic regression model found that the interaction is not needed, but that there might be a negative effect on tone for each “derived” allele, more convincing for *MCPH1*-D. For *ASPM*-D: *β* = −0.22, 89%*HDI* = [−0.66, 0.24], posterior probability *p*(*β* < 0) = 0.78, evidence ratio = 3.5, % HDI inside ROPE = 23.7%, *p*_*ROPE*_ = 0.21; model comparisons to the “null” model suggest rough equivalence (Bayes Factor = 9.37, LOO = 2.40 [SE = 1.07], WAIC = 2.20 [SE = 1.01], KFOLD = 1.54 [SE = 1.62]). For *MCPH1*-D: *β* = −0.41, 89%*HDI* = [−0.77, −0.08], posterior probability *p*(*β* < 0) = 0.95, evidence ratio = 21, % HDI inside ROPE = 1.2%, *p*_*ROPE*_ = 0.058; model comparisons to “null” suggest that they are roughly equivalent (Bayes Factor = 3.35, LOO = 2.30 [SE = 1.36], WAIC = 2.53 [SE = 1.30], KFOLD = 1.72 [SE = 1.79]).

#### Mediation analysis

Using the full dataset and the “classic” approach, both models find a significant positive *TE* of macroarea on tone *counts* (*ASPM*-D: 0.94, 95%CI (0.40, 1.72), *p* < 2 ⋅ 10^−16^; *MCPH1*-D: 0.69, 95%CI (0.32, 1.13), *p* < 2 ⋅ 10^−16^), confirming that African languages tend to be have higher tone counts in our database than languages outside Africa, a non-significant *ADE* of *macroarea* on tone *counts* (*ASPM*-D: replaced−0.16−0.17, 95%CI (−0.69, 0.30), *p* = 0.48; *MCPH1*-D: 0.44, 95%CI (−0.44, 1.38), *p* = 0.32). However, the *ACME*s differ strongly between the two alleles: for *ASPM*-D, the mediated effect is significant and positive (1.11, 95%CI (0.63, 1.79), *p* < 2 ⋅ 10^−16^), representing 100.0% of the total effect and resulting from a significant negative effect of being in Africa on *ASPM*-D (−1.34 ± 0.15, *p* = 1.6 ⋅ 10^−15^), and a significant negative effect of *ASPM*-D on tone counts (−0.73 ± 0.12, *p* = 4.2 ⋅ 10^−10^); however, for *MCPH1*-D, the mediated effect is not significant (0.25, 95%CI (−0.57, 1.06), *p* = 0.53). Taken together, this shows that there’s a positive effect of being in Africa on tone *counts*, but that this is fully mediated by the negative effect of *ASPM*-D, but not by *MCPH1*-D.

**Fig 28:**
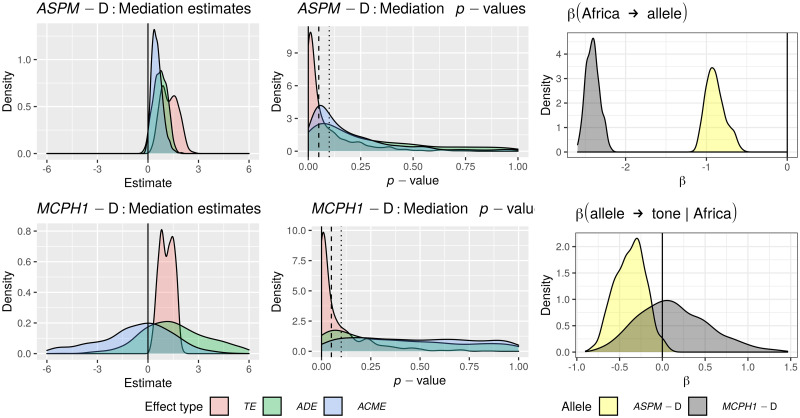
Mediation analysis for 1,000 restricted samples for tone *counts*. Same conventions as in Fig 11. See Table 11 for the numeric summaries.

Restricted sampling (Fig 28 and Table 13), finds that while for both “derived” alleles there are positive total (*TE*) and direct (*ADE*) effects, only *ASPM*-D shows a positive indirect effect (*ACME*), but very few of these effects are individually significant.

**Table 13:** Summaries of the mediation analysis for 1,000 restricted samples for tone *counts*. Same conventions as in Table 9.

Allele	Estimate	Mean	Median	Compared to 0
*ASPM*-D	*TE*	1.3	1.3	> 0: 100.0%, *t* = 79.3, *p* < 2.2 ⋅ 10^−16^
	*ADE*	0.73	0.74	> 0: 97.4%, *t* = 58.4, *p* < 2.2 ⋅ 10^−16^
	*ACME*	0.54	0.50	> 0: 98.4%, *t* = 53.7, *p* < 2.2 ⋅ 10^−16^
	*β*(Africa → allele)	-0.89	-0.90	<0: 100.0%, *t* = −237.7, *p* < 2.2 ⋅ 10^−16^
	*β*(allele → tone ∣ Africa)	-0.38	-0.36	<0: 98.2%, *t* = −66.8, *p* < 2.2 ⋅ 10^−16^
*MCPH1*-D	*TE*	1.1	1.1	> 0: 100.0%, *t* = 83.9, *p* < 2.2 ⋅ 10^−16^
	*ADE*	3.9	2.0	> 0: 83.2%, *t* = 17.2, *p* < 2.2 ⋅ 10^−16^
	*ACME*	-2.8	-0.89	> 0: 35.%, *t* = −12.3, *p* = 1
	*β*(Africa → allele)	-2.4	-2.4	<0: 100.0%, *t* = −915.3, *p* < 2.2 ⋅ 10^−16^
	*β*(allele → tone ∣ Africa)	0.13	0.10	<0: 40.439.8%, *t* = 10.3, *p* = 1

**Fig 29:**
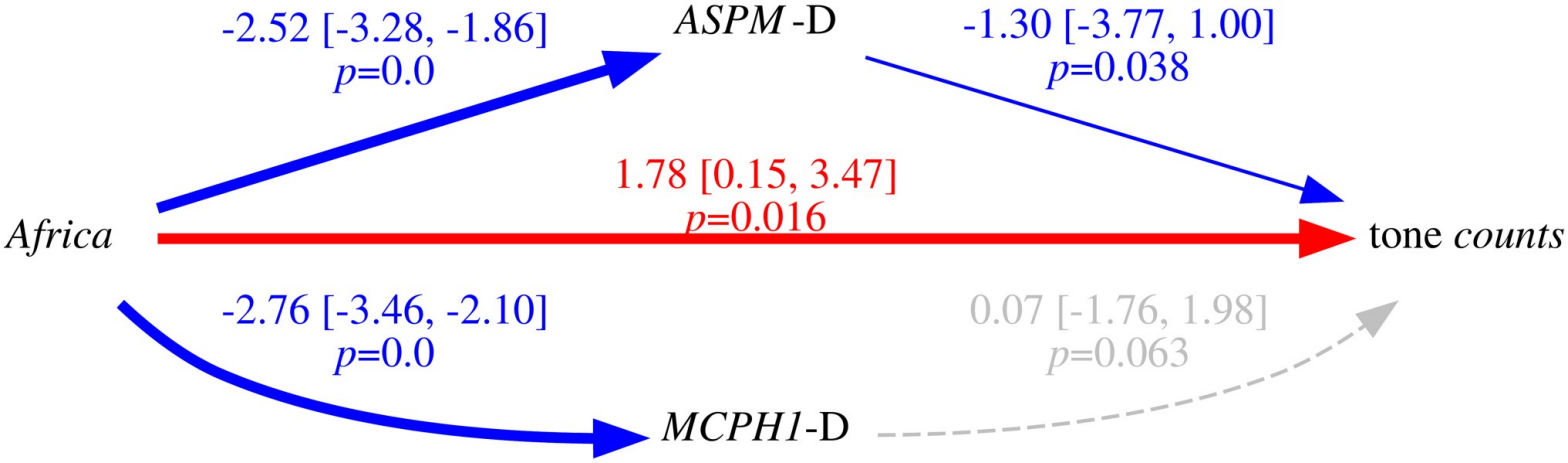
The simultaneous mediation model for tone *counts* through both “derived” alleles. Same conventions as in Fig 12.

The Bayesian approach found a possible positive *TE* for *ASPM*-D (4.89, 89%HDI [−1.29, 10.21], *p*_*ROPE*_ = 0.0063) and less clear for *MCPH1*-D (1.93, 89%HDI [−2.40, 6.14], *p*_*ROPE*_ = 0.026), clear positive *ADE*’s (*ASPM*-D: 1.69, 89%HDI [0.52, 2.97], *p*_*ROPE*_ = 0.007; *MCPH1*-D: 2.06, 89%HDI [0.59, 3.69], *p*_*ROPE*_ = 0.0058). However, while there seem to be a positive *ACME* for *ASPM*-D (3.19, 89%HDI [−2.98, 9.26], *p*_*ROPE*_ = 0.017) resulting from a negative effect of being in Africa on *ASPM*-D (−2.52, 89%HDI [−3.21, −1.79], *p*_*ROPE*_ = 0.0) and a possible negative effect of *ASPM*-D on tone (−1.29, 89%HDI [[−3.73, 1.04], *p*_*ROPE*_ = 0.036), this is arguably absent for *MCPH1*-D (*ACME*: −0.22, 89%HDI [−5.53, 4.78], *p*_*ROPE*_ = 0.02; negative effect of being in Africa on *MCPH1*-D: −2.75, 89%HDI [−3.37, −2.02], *p*_*ROPE*_ = 0.0; null effect of *MCPH1*-D on tone: 0.09, 89%HDI [−1.82, 1.89], *p*_*ROPE*_ = 0.062). Simultaneously modelling the mediation of both “derived” alleles produces similar results (Fig 29): positive *TE* (4.86, 89%HDI [−2.21, 11.80], *p*_*ROPE*_ = 0.012) and *ADE* (1.78, 89%HDI [0.15, 3.47], *p*_*ROPE*_ = 0.016), with an arguably positive *ACME* for *ASPM*-D (3.17, 89%HDI [−2.92, 9.28], *p*_*ROPE*_ = 0.013), resulting from a negative effect of being in Africa on *ASPM*-D (−2.52, 89%HDI [−3.28, −1.86], *p*_*ROPE*_ = 0.0) and a possible negative effect of *ASPM*-D on tone (−1.30, 89%HDI [−3.77, 1.00], *p*_*ROPE*_ = 0.038), but much less convincing evidence of an *ACME* for *MCPH1*-D (−0.18, 89%HDI [−5.49, 4.94], *p*_*ROPE*_ = 0.024) resulting from a negative effect of being in Africa on *MCPH1*-D (−2.76, 89%HDI [−3.46, −2.10], *p*_*ROPE*_ = 0.0) and no effect of *MCPH1*-D on tone (0.07, 89%HDI [−1.76, 1.98], *p*_*ROPE*_ = 0.063).

#### Path analysis

**Fig 30:**
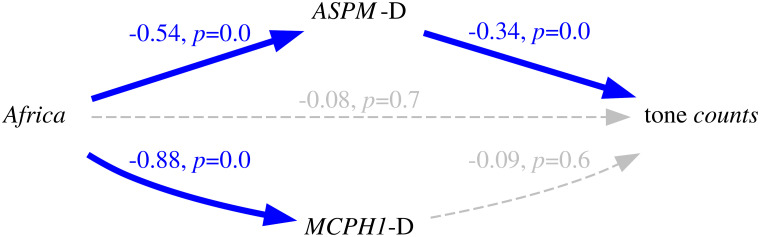
The path model for tone *counts* fitted on all the data. Same conventions as in Fig 13.

**Fig 31:**
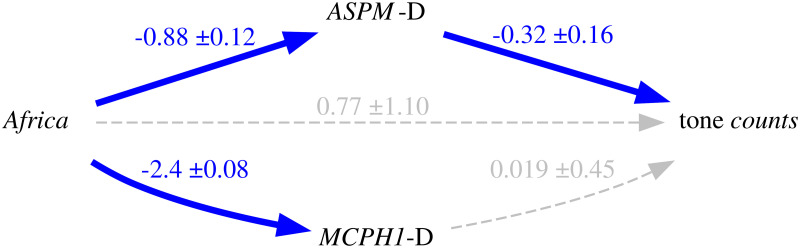
Summary of the 1,000 path models for tone *counts* fitted to restricted samples. Same conventions as in Fig 14.

On the full dataset, the model fits the data very well ($\chi_{1}^{2}=0.29$, *p* = 0.59; *CFI* = 1.00, *TLI* = 1.01, *NNFI* = 1.01 and *RFI* = 1.00), and, as shown in Fig 30, being in Africa has no direct effect on tone counts, but has a significant negative effect on *ASPM*-D which has a negative significant effect on tone counts, but while it has a stronger significant negative effect on *MCPH1*-D this has no effect on tone counts. The same pattern is also found by restricted sampling, except that here there is also a hint of a negative effect of *MCPH1*-D on complex tone (see Fig 31 and Table 14).

**Table 14:** Summaries of the path analysis for 1,000 restricted samples for tone *counts*. Similar conventions as for Table 9.

Type	Estimate	Mean (SD)	Median (IQR)	Compared to 0
**Fit**	*χ*^2^ test	97.9% n.s.	-	–
	*CFI*	0.99 (0.01)	1.00 (0.01)	-
	*TLI*	0.99 (0.09)	1.01 (0.15)	-
	*NNFI*	0.99 (0.09)	1.01 (0.15)	-
	*RFI*	0.91 (0.08)	0.93 (0.13)	-
**Path**	Africa → *counts*	0.77 (1.10)	0.93 (1.60)	74.9% > 0
	Africa → *ASPM*-D	−0.88 (0.12)	−0.89 (0.16)	100.0% < 0
	*ASPM*-D → *counts*	−0.32 (0.16)	−0.31 (0.22)	98.7% < 0
	Africa → *MCPH1*-D	−2.40 (0.08)	−2.40 (0.12)	100.0% < 0
	*MCPH1*-D → *counts*	0.02 (0.45)	0.03 (0.65)	47.4% < 0

#### Summary

The actual number of tones/tone symbols has a skewed distribution, and the results are less clear, but it seems that there is a negative effect of *ASPM*-D on tone counts above and beyond the confounding effects of macroarea and family, but not so for *MCPH1*-D (except when using a Gaussian Process to model contact).

### Bayesian regressions: Sensitivity to priors

Given the importance of the Bayesian models in this analysis, I checked if the Bayesian regressions of *tone1*, *tone2* and tone *counts* on the *z*-scored population frequency of each “derived” allele separately while controlling for *family*, *(meta)population* and *macroarea* as random effects (i.e., the models used above) are *sensitive* to the particular choice of prior distributions used. This is a standard technique in Bayesian data analysis (e.g., [30]) that, first, makes sure that the chosen prior distribution does not unduly affects the results (in effect, creating artefacts) and, second, is a different way of getting a “feeling” of how much information there is in the data. In the analyses presented above I systematically used a student’s *t*(*df* = 3, *μ* = 0, *σ* = 3) prior for the intercept *α* and the slopes *β*, where *μ* is the location parameter (the median) and *σ* is the scale parameter, resulting in a relatively wide spread around 0 for *z*-scored predictors (see also the prior recommendations for Stan at https://github.com/stan-dev/stan/wiki/Prior-Choice-Recommendations). To this, I compared a set of 13 other priors explicitly chosen for their properties and relevance (see Table 15) by fitting the exact same regression model to the exact same data (varying only the intercept and slope prior) separately for the *z*-scored population frequency of *ASPM*-D and of *MCPH1*-D, respectively. For each such model I focused on the slope *β* of the predictor, and I collected the point estimate with its 89%HDI (see Table 16; for full results please see *Appendix II* in the S1 File). First, it can be seen that the estimates are negative for all priors, including for the extreme positive one (except for *MCPH1*-D → *tone2*), suggesting that indeed, the “derived” alleles probably have a negative effect on tone after controlling for family and macroarea. Second, the “default”, the “flat” and the “default normal” priors produce similar estimates for *tone1* and tone *counts* (for both alleles) and slightly less so for *tone2*, suggesting that the “default” prior does not bias the results too much. Thus, there is enough information in the data to override even the extreme priors (and clearly the “default” prior used), and this information consistently points to a negative effect of the “derived” alleles on the three codings of tone.

**Table 15:** The priors used for the sensitivity analyses of the Bayesian regressions. The student’s *t*(*df*, *μ*, *σ*) distribution has parameters *df* = degrees of freedom, *μ* = location (the median) and *σ* = scale, while the *normal*(*μ*, *σ*) has parameters *μ* = mean and *σ* = standard deviation. Remember that these distributions refer to the intercept and slope of a *z*-scored predictor (i.e., one with mean 0 and standard deviation 1) that is expected to have a weak and possibly negative effect.

Name	Distribution	Description
default	*t*(3, 0, 3)	the distribution used in the analyses reported above; relatively wide spread around 0 following recommendations
flat	*normal*(0, 10)	very wide around 0 (i.e., “uninformative”)
default normal	*normal*(0, 5)	very similar to the “default” but using the normal distribution instead of student’s *t*
narrow zero	*t*(3, 0, 1)	relatively narrow around 0 (i.e., assuming the parameter is 0)
very narrow zero	*t*(3, 0, 0.1)	very narrow around 0 (i.e., strongly assuming the parameter is 0)
default negative	*t*(3, −1, 3)	wide around -1 (i.e., weakly assuming the parameter is negative)
narrow negative	*t*(3, −1, 1)	relatively narrow around -1 (i.e., assuming the parameter is negative)
default very negative	*t*(3, −3, 3)	wide around -3 (i.e., weakly assuming the parameter is very negative)
narrow very negative	*t*(3, −3, 1)	relatively narrow around -3 (i.e., assuming the parameter is very negative)
default positive	*t*(3, 1, 3)	wide around 1 (i.e., weakly assuming the parameter is positive)
narrow positive	*t*(3, 1, 1)	relatively narrow around 1 (i.e., assuming the parameter is positive)
default very positive	*t*(3, 3, 3)	wide around 3 (i.e., weakly assuming the parameter is very positive)
narrow very positive	*t*(3, 3, 1)	relatively narrow around 3 (i.e., assuming the parameter is very positive)
informative	*t*(3, *x*, 3)	relatively wide centred around the estimate *x* derived from the actual results using the default prior

**Table 16:** The results of the prior sensitivity analysis. Showing the point estimate and 89% HDI of the slope *β* for each of the three DVs (*tone1*, *tone2* and tone *counts*) and two IVs (*z*-scored population frequency of *ASPM*-D and *MCHP1*-D). See Table 15 for prior acronyms.

Prior	Allele	*tone1*	*tone2*	tone *counts*
default	*ASPM*-D	-0.70 [-1.52, 0.33]	-1.30 [-2.72, 0.17]	-0.24 [-0.65, 0.16]
flat	*ASPM*-D	-0.74 [-1.63, 0.24]	-1.76 [-3.44, 0.22]	-0.25 [-0.67, 0.16]
default normal	*ASPM*-D	-0.70 [-1.60, 0.24]	-1.50 [-3.04, 0.22]	-0.24 [-0.66, 0.16]
narrow zero	*ASPM*-D	-0.55 [-1.32, 0.23]	-0.67 [-1.73, 0.40]	-0.22 [-0.63, 0.18]
very narrow zero	*ASPM*-D	-0.05 [-0.28, 0.16]	-0.03 [-0.23, 0.20]	-0.04 [-0.22, 0.14]
default negative	*ASPM*-D	-0.75 [-1.71, 0.13]	-1.42 [-2.85, 0.07]	-0.26 [-0.69, 0.15]
narrow negative	*ASPM*-D	-0.82 [-1.60, -0.06]	-1.05 [-1.98, 0.02]	-0.32 [-0.74, 0.07]
default very negative	*ASPM*-D	-0.87 [-1.83, 0.10]	-1.76 [-3.33, -0.17]	-0.28 [-0.69, 0.14]
narrow very negative	*ASPM*-D	-1.33 [-2.44, -0.21]	-1.89 [-3.12, -0.61]	-0.34 [-0.77, 0.09]
default positive	*ASPM*-D	-0.62 [-1.52, 0.28]	-1.21 [-2.58, 0.36]	-0.23 [-0.67, 0.16]
narrow positive	*ASPM*-D	-0.34 [-1.13, 0.52]	-0.45 [-1.81, 0.77]	-0.15 [-0.55, 0.27]
default very positive	*ASPM*-D	-0.55 [-1.39, 0.44]	-1.14 [-2.60, 0.44]	-0.22 [-0.62, 0.21]
narrow very positive	*ASPM*-D	-0.23 [-1.12, 0.78]	-0.40 [-1.94, 1.51]	-0.14 [-0.57, 0.27]
informative	*ASPM*-D	-0.72 [-1.64, 0.21]	-1.48 [-2.94, 0.10]	-0.26 [-0.66, 0.14]
default	*MCPH1*-D	-0.65 [-1.69, 0.47]	-0.93 [-2.41, 0.53]	-0.23 [-0.70, 0.20]
flat	*MCPH1*-D	-0.68 [-1.76, 0.48]	-1.23 [-3.11, 0.51]	-0.24 [-0.66, 0.24]
default normal	*MCPH1*-D	-0.67 [-1.76, 0.47]	-1.07 [-2.60, 0.66]	-0.24 [-0.68, 0.24]
narrow zero	*MCPH1*-D	-0.49 [-1.35, 0.46]	-0.47 [-1.61, 0.68]	-0.21 [-0.65, 0.21]
very narrow zero	*MCPH1*-D	-0.04 [-0.25, 0.17]	-0.01 [-0.25, 0.19]	-0.04 [-0.22, 0.16]
default negative	*MCPH1*-D	-0.73 [-1.79, 0.35]	-1.05 [-2.58, 0.45]	-0.24 [-0.69, 0.22]
narrow negative	*MCPH1*-D	-0.80 [-1.74, 0.03]	-0.89 [-1.98, 0.15]	-0.30 [-0.73, 0.11]
default very negative	*MCPH1*-D	-0.85 [-1.93, 0.27]	-1.31 [-2.88, 0.30]	-0.25 [-0.71, 0.19]
narrow very negative	*MCPH1*-D	-1.31 [-2.51, -0.09]	-1.73 [-3.11, -0.32]	-0.34 [-0.77, 0.09]
default positive	*MCPH1*-D	-0.61 [-1.64, 0.52]	-0.81 [-2.25, 0.84]	-0.22 [-0.66, 0.23]
narrow positive	*MCPH1*-D	-0.22 [-1.26, 0.80]	-0.08 [-1.36, 1.31]	-0.13 [-0.59, 0.32]
default very positive	*MCPH1*-D	-0.47 [-1.56, 0.59]	-0.67 [-2.30, 0.85]	-0.20 [-0.65, 0.25]
narrow very positive	*MCPH1*-D	-0.06 [-1.39, 1.16]	0.40 [-1.51, 2.62]	-0.12 [-0.59, 0.37]
informative	*MCPH1*-D	-0.66 [-1.83, 0.42]	-1.01 [-2.44, 0.46]	-0.24 [-0.71, 0.17]

### Power analysis

I used a simulation approach (as implemented by the simr package [98]) to conduct a post-hoc power analysis of the effect of *ASPM*-D on *tone1*. More precisely, I fitted a “classic” maximum-likelihood mixed-effects logistic regression of *tone1* on *z*-scored *ASPM*-D while controlling for language *family* (as random effect) and *macroarea* (as fixed effect) using all the available data (*n* = 181 observations across 35 families), resulting in a non-significant observed effect of *ASPM*-D *β* = −0.4 with *p* = 0.41, and an (adjusted) intra-class correlation *ICC* = 68.4%. The achieved (observed) power for the *α*-level 0.05 is 1 − *β* = 15.1%, 95%CI = (12.9%, 17.5%), much lower than the usual 80%, showing that, indeed, with these data and method, there is a very low chance of detecting such a weak effect of *ASPM*-D if it were true.

When counterfactually changing the number of languages per family (while keeping everything else constant) suggests that the 80% power threshold would be achieved only if there were at least ≈700 languages per family, while changing the number of families (while keep everything else constant) suggests that we reach 80% power only at ≈350 families! Independently changing the number of families and languages suggests that the region with > 80% power needs either many languages per family or many families, with the best trade-off reached for ≈100 to ≈200 families with ≈250 to ≈100 languages each.

For comparison, currently there are ≈150 families in the Ethnologue and ≈420 in Glottolog, the latter listing a maximum of ≈1400 languages per family (Atlantic-Congo), with a mean of ≈20, and a median of 2 or of 5 (when excluding isolates). Of course, this analysis must be taken as only an indication of the rough order of magnitude but, given the distribution of linguistic diversity, it suggests that using such a model would require an almost exhaustive sampling and even then its success would not be guaranteed. Interestingly, for the rather different question of absolute universals (i.e., the non-existence of logically possible feature values), the bootstrapping and Bayesian analyses in [99] similarly suggest that at least hundreds of statistically independent languages might be required.

### Treating macroareas independently

The fact that the population frequencies of the two “derived” alleles is so different between Africa and the rest of the world, on the one hand, while being relatively uniform within each of these two geographic regions (particularly strong for *MCPH1*-D, raises the question of performing the analysis (a) excluding Africa, and (b) within each of the four macroareas separately. The full results can be found in the *Appendix IV: Macroareas as units of analysis* of the S1 File analysis report, but, briefly, as America and Papunesia have very few unique observations (each 10 in 6 families, respectively), they cannot be analysed independently, resulting in three analyses: (a) Eurasia + America + Papunesia (i.e., excluding Africa), (b1) just Africa, and (b2) just Eurasia. For these analyses, I did only the regression analyses (no Gaussian Process Bayesian regressions). In all these analyses, there is no detectable effect of the “derived” alleles on tone (except for restricted sampling and Bayesian repressions, which seems to find a negative effect of *ASPM*-D in certain cases). While these essentially null results at the level of the macroareas and when excluding Africa can be taken to show that the effect of *ASPM*-D on tone obtained using the full dataset are an artefact of the differences between the macroareas, and between Africa and non-Africa in particular (akin to Simpson’s paradox [30, 74]), I do not think that this interpretation is warranted. Instead, not finding a relationship within the individual macroareas and when excluding Africa is rather unsurprising given the small expected effect size, coupled with the very uniform population frequency of the “derived” alleles, and the small number of observations and their high clustering within families. Therefore, we should instead use all the data while modelling the family and macroarea structure, in particular that between Africa and non-Africa, as I have done in the regression, mediation and path analysis approaches.

### Summary of results

The three types of mixed-effects regression models using the full dataset, (a) the “classic” (maximum likelihood, considering family as a random effect but macroarea as a fixed effect), (b) the Bayesian (which models family, (meta)population and macroarea as random effects), and (c) the “experimental” Bayesian with Gaussian Process (which models family and (meta)population as random effects but contact as a continuous Gaussian Process split by macroarea), find effects of relatively similar sizes and of the same sign for the influence of each of the two “derived” alleles on each of the three measures of tone, but with different probabilities of being “significant” (for (a) they are uniformly n.s. when controlling for macroarea, for (b) the posterior probability *p*(*β* < 0) of a strictly negative effect is > 80%, the % HDI inside ROPE <21% and *p*_*ROPE*_ < 0.2, while for (c) *p*(*β* < 0) > 78%, the % HDI inside ROPE <24% and *p*_*ROPE*_ < 0.2 across both alleles and the three measures of tone). Thus, while all three suggest weak negative effects on tone of both “derived” alleles, the Bayesian approaches seem more powerful at detecting these effects above and beyond the confounds (especially macroarea); moreover, overall there seems to be a stronger effect of *ASPM*-D relative to *MCPH1*-D. Moreover, the Bayesian regression models not only are not very sensitive to the prior (including extremely biased ones), suggesting that there is enough signal in the data, but their results consistently point to a negative effect of the “derived” alleles on tone. Performing maximum likelihood mixed-effects regressions with family as a random effect on repeatedly permuted data (under various constraints) suggests that there is a weak negative effect of *ASPM*-D on *tone1* and *tone2* that is not entirely due to macroarea, but that there is strong similarity within language families (both confirming the high intra-class correlations for family and suggesting that a stratified sampling of one language per family is sufficiently appropriate). However, *MCPH1*-D has no “special” effect on neither of the three tone variables, nor does *ASPM*-D on tone *counts*, except when all structure is destroyed through unrestricted permutations (i.e., when each sample is considered as statistically independent of all the others). Likewise, repeatedly fitting maximum likelihood regressions on restricted samples (where only one language is randomly picked per family) while controlling for macroarea and the other “derived” allele as fixed effects, shows that there is a weak negative effect of *ASPM*-D on all three measures of tone, and possibly of *MCPH1*-D on *tone1*, but arguably not on *tone2* or tone *counts*.

Mediation analysis looks at the total influence of being an African language or not (split justified by the marked differences in the frequencies of the two “derived” alleles for populations within and outside Africa) on the three measures of tone mediated separately by the frequency of the two “derived” alleles. On the full dataset, I used both a “classic” maximum likelihood approach (which does not control for language families directly and are more restricted in terms of regression models available) and a Bayesian one (where family is modelled as a random effect and allows the use of more appropriate regression models, as well as the “experimental” mediation through both “derived” alleles simultaneously), and the two agree except in one case, where the Bayesian approach is more powerful at detecting a mediation. For *tone1*, both find a positive mediation through *ASPM*-D (composed of a negative effect of being in Africa on *ASPM*-D, and a negative effect of *ASPM*-D on *tone1*), and both fail to find mediation through *MCPH1*-D. For *tone2*, both fail to find mediation through *MCPH1*-D, but they disagree for *ASPM*-D: while the maximum-likelihood approach fails to find a significant mediation (even if both its components are each significant), the Bayesian one does find a positive one (composed of a two negative effects, as above). For tone *counts*, both find a positive mediation through *ASPM*-D (composed of a two negative effects, as above), and both fail to find mediation through *MCPH1*-D. Repeatedly conducting “classic” maximum likelihood mediation analyses when extracting only one language per family at random (restricted sampling, implementing a control for family), paints a very similar picture: there is a clear positive mediation effect of *ASPM*-D on all three measures of tone (composed of negative partial effects of being an African language on *ASPM*-D, and a negative effect of *ASPM*-D on tone), but much less so for *MCPH1*-D.

Path analysis has the added advantage that it models the mediation of the effect of within versus outside Africa on tone through both “derived” alleles simultaneously, but at the cost of less flexibility in the types of models that can be used, and of not directly controlling for language families (the “experimental” simultaneous mediation technique can satisfy both desiderata but it is not, at the moment, sufficiently well tested to be considered otherwise). On the full dataset and on repeated restricted samples of a single language per family, the results are very similar, in that there is a clear mediation through *ASPM*-D for all three measures of tone, composed of a negative effect of being in Africa on *ASPM*-D, and a negative effect of *ASPM*-D on tone, but not for *MCPH1*-D.

The decision trees and random forests suggest that *ASPM*-D and, to a lesser extent, *MCPH1*-D, contain information with regard to *tone1* and *tone2* above that provided by macroarea (but there is no control for language family at all).

The post-hoc power analysis of the results obtained from the maximum-likelihood mixed-effects regression of *tone1* on *ASPM*-D with family as a random effect and macroarea as a fixed effect suggest that this method would require too much data to be feasible.

Finally, the separate analyses of each macroarea independently and when excluding Africa, suggest that our interpretation should be based on the whole dataset at hand but modeling appropriately the areal and family structure of the data, as done in the mixed-effects regression, mediation and path analyses.

## Discussion and conclusions

Using updated data and methods relative to the original paper [9], I found, first, that the population frequencies of the two “derived” alleles (denoted *ASPM*-D and *MCPH1*-D), as well as the distribution of tone, coded as the presence/absence of any type of tone system (denoted *tone1*), of complex tone systems (*tone2*), or as the actual number of tones/tone symbols (tone *counts*), are strongly clustered within language families and macroareas. This is not surprising given that they are all shaped by similar processes, with a strong vertical transmission component (inheritance) coupled with various degrees of horizontal transmission (contact). However, this is a very important result, as it confirms the need to properly disentangle the confounding effects of inheritance (here, depending on the method, using the language families and the (meta)populations as proxies) and contact (also depending on the method, using the macroareas and the geographic distances within them as proxies), from the proposed causal effects of the two “derived” alleles on tone. On the other hand, we need to exercise care because we may risk “throwing the baby with the bathwater” in the sense that this type of causal effect is expected to act on comparable timescales to the processes affecting languages and the genetic structure of populations and, thus, to be similar to the confounding effects of inheritance and contact.

Therefore, I used multiple methods of controlling for these confounding effects, including the “standard” mixed-effects regression approach of modelling family as a random effect, and macroarea as a fixed effect, as a random effect, or through a bi-dimensional Gaussian Process of the geographic coordinates of the samples, but also by permuting the language and genetic data according to multiple types of constraints (none, within families, and within macroareas), and by repeatedly sampling only one data point from each family. Moreover, besides the regression approach which quantifies the “extra” effect of the alleles on tone left after removing the effects of macroarea and family, I also used mediation and path analysis, which allow the explicit modelling of the interplay between macroarea, tone and the alleles, as well as decision trees and random forests, which quantify the capacity and relative importance of macroarea and the two alleles for predicting tone.

With these, it is interesting to note that the results are largely consistent between the methods, and that, despite their differences, when considered together, they do paint a rather coherent picture. This overall picture is one of a *weak negative effect* of the population frequency of *ASPM*-D on tone above and beyond the effects of contact and inheritance, but these effects are largely overlapping in the sense that, on the one hand, samples from the same language family tend to be very similar to each other genetically and linguistically, while, on the other, being a sample from Africa or from outside Africa has a major effect on *ASPM*-D and (much less so) on tone. This “extra” weak negative effect of *ASPM*-D on tone means that languages spoken by populations with a high frequency of *ASPM*-D have, overall, a slight tendency to not use tone, and, if they do, to have a simpler tone system. It is interesting to note that the distribution of tone languages is clearly skewed relative to the high frequencies of *ASPM*-D, and that there seems to be a qualitative change around 25% from *Abau* (glottocode abau1245, Sepik; simple system; 57.5% *ASPM*-D), *Swedish* (swed1254, Indo-European; simple “pitch accent”; two samples with 49.5% and 43.8%), *Western Balochi* (west2368, Indo-European; simple; 32.0%), *Eastern Panjabi* (panj1256, Indo-European; simple system; 30.7%), *Burushaski* (buru1296, Burushaski; probably not?; 28.0%), *Awngi* (awng1244, Afro-Asiatic; simple; 27.4%), and a set of 7 Austronesian languages (cemu1238, fwai1237, kara1486, labu1248, kuma1276, xara1244 and yabe1254) ambiguously matching the “Micronesians” (*ALFRED* SA004382R) genetic sample with 27.3% *ASPM*-D with simple tone systems, to *Lü* and *Tai Nüa* (luuu1242 and tain1252, Tai-Kadai; complex tone systems; 25.0%), *Sichuan Yi* (sich1238, Sino-Tibetan; simple; 25.0%), *She* (shee1238, Hmong-Mien; complex; 23.4%) and *Mandarin Chinese* and *Yue Chinese* (mand1415 and yuec1235, Sino-Tibetan; complex; 23.1%). At the other extreme, a low frequency of *ASPM*-D clearly does not require the presence of tone. Concerning *MCPH1*-D, while it apparently also has a negative effect on tone on its own, this effect seems to be in large part driven by its very skewed distribution within versus outside Africa, and does not survive most of the correction techniques used here.

However, there are differences between methods that might give hints about the nature of the effects and confounding factors. One of the most important concerns the mixed-effects regressions on the full dataset, as they implement one of the currently *de facto* standard methods of controlling for the confounding effects of inheritance and contact [27, 28]. While the “classic” maximum-likelihood approach, with family as a random effect and macroarea as a fixed effect, fails to find any significant effect of *ASPM*-D on tone at the standard *α*-level of 0.05, the Bayesian ones, with family and (meta)population as random effects, and macroarea as random effect or as modelled by a bi-dimensional Gaussian Process, do find some evidence for a weak negative effect of both *ASPM*-D and *MCPH1*-D on all three measures of tone, but this evidence is far from overwhelming on its own. As the randomisation, restricted sampling, and the mediation and path analyses show, this failure very probably emerges, in large part, from, on the one hand, the very high similarity of the languages and of the genetic samples from the same family and macroarea (especially, for the genetic samples, Africa versus the rest of the world), and, on the other, from the inadequate modelling of the relationship between macroarea and the frequency of the two “derived” alleles. To end, the fact that modelling contact through a set of continuous 2D Gaussian Processes (one per macroarea) over the geographic coordinates of the samples, finds a stronger negative effect of *MCPH1*-D on *tone1* and tone *counts*, and comparable on *tone2*, than *ASPM*-D, suggests that the story might be even more complex, with either an actual effect of *MCPH*-D on tone in certain circumstances or pointing to issues with using a Gaussian Processes to control for contact in such a way.

Thus, the more (and, in some cases, more refined) data that became available since 2007 concerning both the population frequency of the two “derived” alleles (either directly, or inferred through proxies in high linkage disequilibrium that seem to induce minimal noise) and tone, and the newer methods that became popular since, support a *weak negative effect of ASPM-D but probably not of MCPH1-D* after removing the effects of contact and inheritance, modelled as macroareas and language families, respectively. Nevertheless, the work presented here shows the main limiting factor remains the availability of good quality genetic data with good geographic and linguistic coverage (hopefully, such data will become available from more populations across the globe, and maybe even from past groups using ancient DNA techniques), coupled with appropriate methods of analysis. For example, there are only 10 data points from the Americas and 10 from Papunesia, and none from Australia, the latter being a particularly interesting case [54]. If, indeed, *ASPM*-D has a weak negative effect on tone, and if *ASPM*-D has a low frequency among the Aboriginal Australian populations (as could be reasonably expected given the age of the allele and the apparently long genetic isolation of Australia [40, 100]), then we would expect to find at least some tone languages among its Aboriginal languages, but this is clearly not the case [34, 35]. However, there are at least two solutions to this (potential) paradox [54]: first, the small negative bias of *ASPM*-D on tone is inherently probabilistic and, moreover, does not place constraints on language change and evolution at (very) low frequencies of this allele, so that it is not entirely inconceivable that accidents of history and linguistic expansions and extinctions have resulted in a linguistic Australian landscape that does not include tone at this moment. The second is based on the suggested long-term effects of a high incidence of Chronic Otits Media (COM) among the Aborigine populations on the phonetics and phonology of Australian languages [101], in particular the loss of sensitivity in the low frequency range which, naturally, should explain the absence of tone distinctions (just as it explains a lack of voicing contrasts).

Interestingly, while the original data and analyses in [9] did not find anything that would suggest a qualitative difference between *ASPM*-D and *MCPH1*-D in how they affect tone, the results reported here do, with the effect of *MCPH1*-D apparently confounded, in large part, by its skewed distribution inside versus outside Africa. This is consonant with the experimental findings of [25] and [26], which supported a (negative) effect of *ASPM*-D on tone perception and/or processing, but failed to find any effect of *MCPH1*-D.

## Supporting information

S1 Fig The agreement between the 5 sources for tone. Each panel shows a pair of sources (e.g., the top-left panel shows *LAPSyD* on the vertical axis and *DL2007* on the horizontal axis); please note that the pairs are symmetric, so that the *DL2007* vs *LAPSyD* panel is not shown; likewise, the identity panels (e.g., *LAPSyD* vs *LAPSyD*) on the diagonal are also not shown. Each panel shows the number of languages with each possible combination of values from the two sources (e.g., for “None” vs “No”, there are 12 languages, but there’s no language for “None” vs “Yes”). The shade of blue varies between white (the lowest count) to light blue (highest count). A high agreement between two sources results in little discrepancy between corresponding values (e.g., all “None” in *LAPSyD* map to “No” in *DL2007* and vice-versa, while “Marginal”, “Simple”, “Moderately complex” and “Complex” map to “Yes”).
(TIF)

S2 Fig The agreement binary classification of tone versus the original sources *WALS*, *LAPSyD* and *DL2007*. The same conventions as for S1 Fig.
(TIF)

S3 Fig The agreement 3-way classification of tone versus the original sources *WALS*, *LAPSyD* and *DL2007*. The same conventions as for S1 Fig.
(TIF)

S4 Fig The agreement for tone counts versus the original sources *LAPSyD*, *PHOIBLE* and *WPHON*. The same conventions as for S1 Fig.
(TIF)

S1 File (HTML)
