# Supplementary figures and images for: Tone and genes: New cross-linguistic data and methods support the weak negative effect of the “derived” allele of ASPM on tone, but not of Microcephalin
Source: PLoS One. 2021 Jun 30;16(6):e0253546. doi: 10.1371/journal.pone.0253546 (PMC8244921; doi:10.1371/journal.pone.0253546)

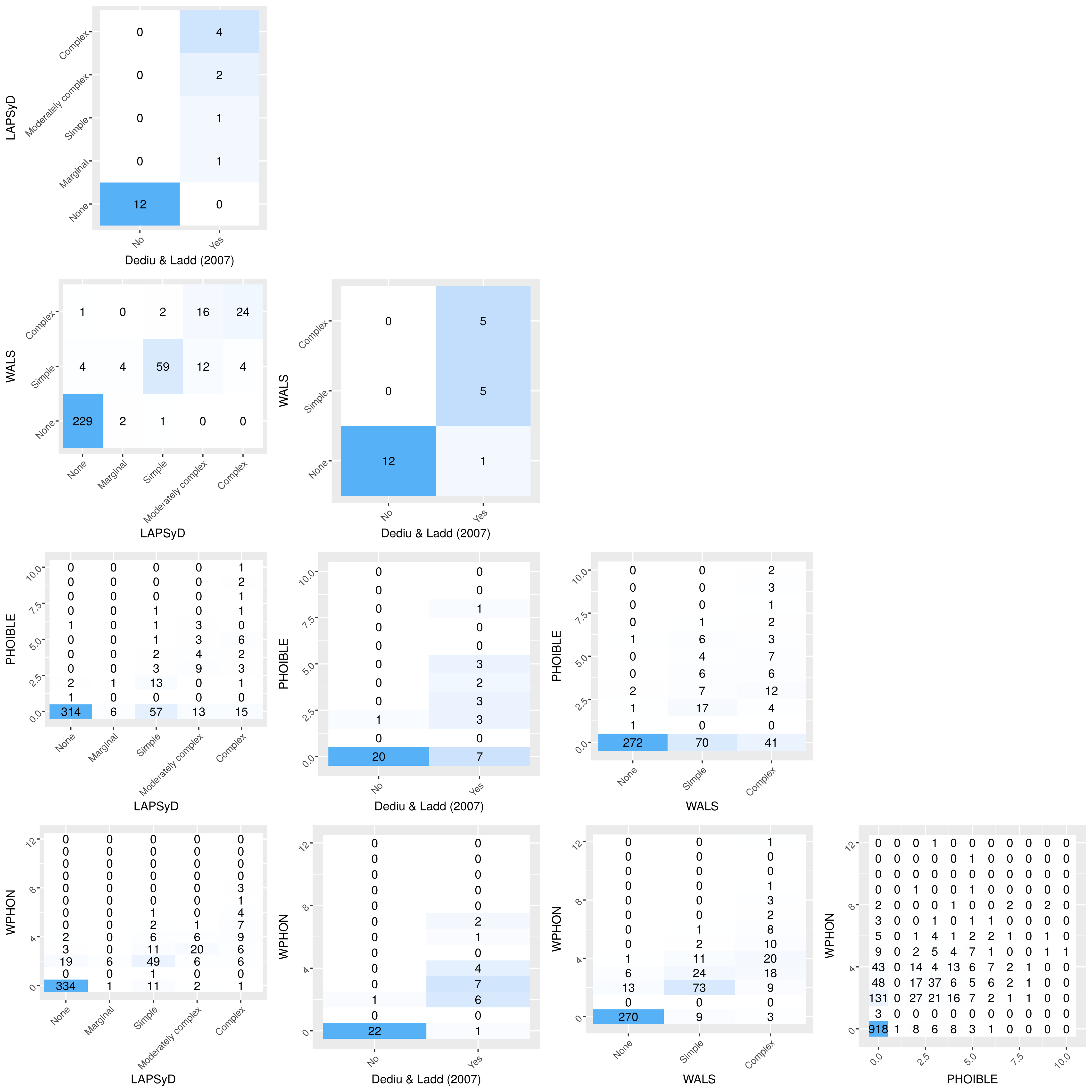

Supplement: S1 Fig — Each panel shows a pair of sources (e.g., the top-left panel shows LAPSyD on the vertical axis and DL2007 on the horizontal axis); please note that the pairs are symmetric, so that the DL2007 vs LAPSyD panel is not shown; likewise, the identity panels (e.g., LAPSyD vs LAPSyD) on the diagonal are also not shown. Each panel shows the number of languages with each possible combination of values from the two sources (e.g., for “None” vs “No”, there are 12 languages, but there’s no language for “None” vs “Yes”). The shade of blue varies between white (the lowest count) to light blue (highest count). A high agreement between two sources results in little discrepancy between corresponding values (e.g., all “None” in LAPSyD map to “No” in DL2007 and vice-versa, while “Marginal”, “Simple”, “Moderately complex” and “Complex” map to “Yes”). (TIF) [file pone.0253546.s001.tif]

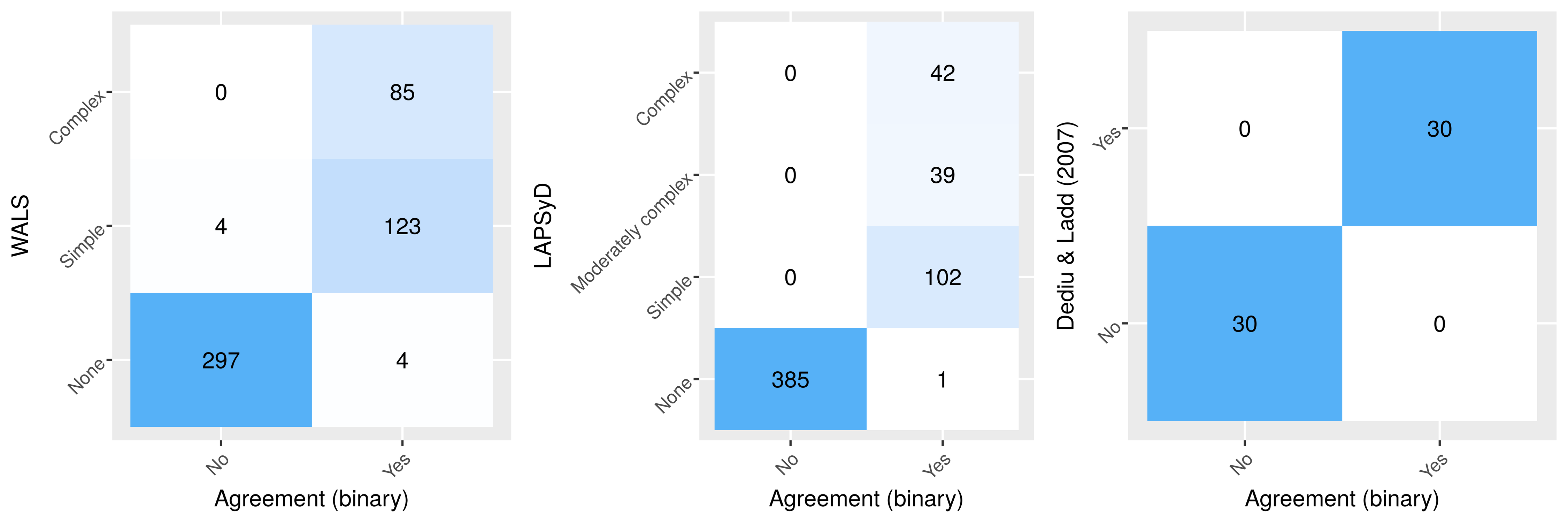

Supplement: S2 Fig — The same conventions as for S1 Fig. (TIF) [file pone.0253546.s002.tif]

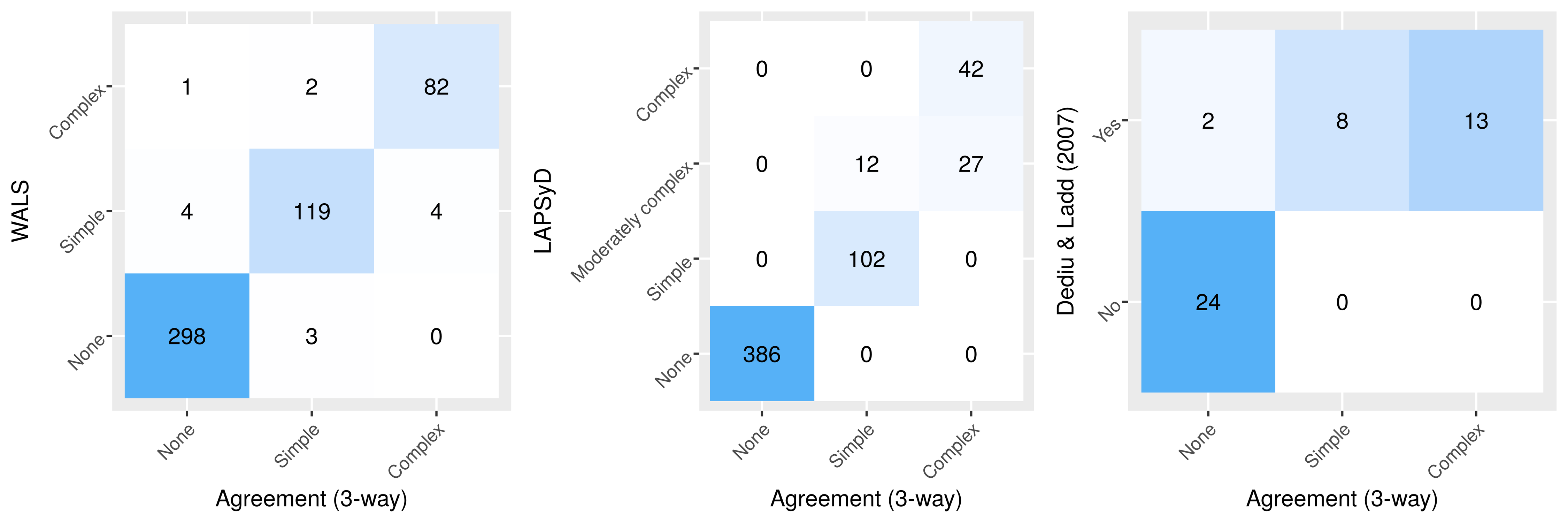

Supplement: S3 Fig — The same conventions as for S1 Fig. (TIF) [file pone.0253546.s003.tif]

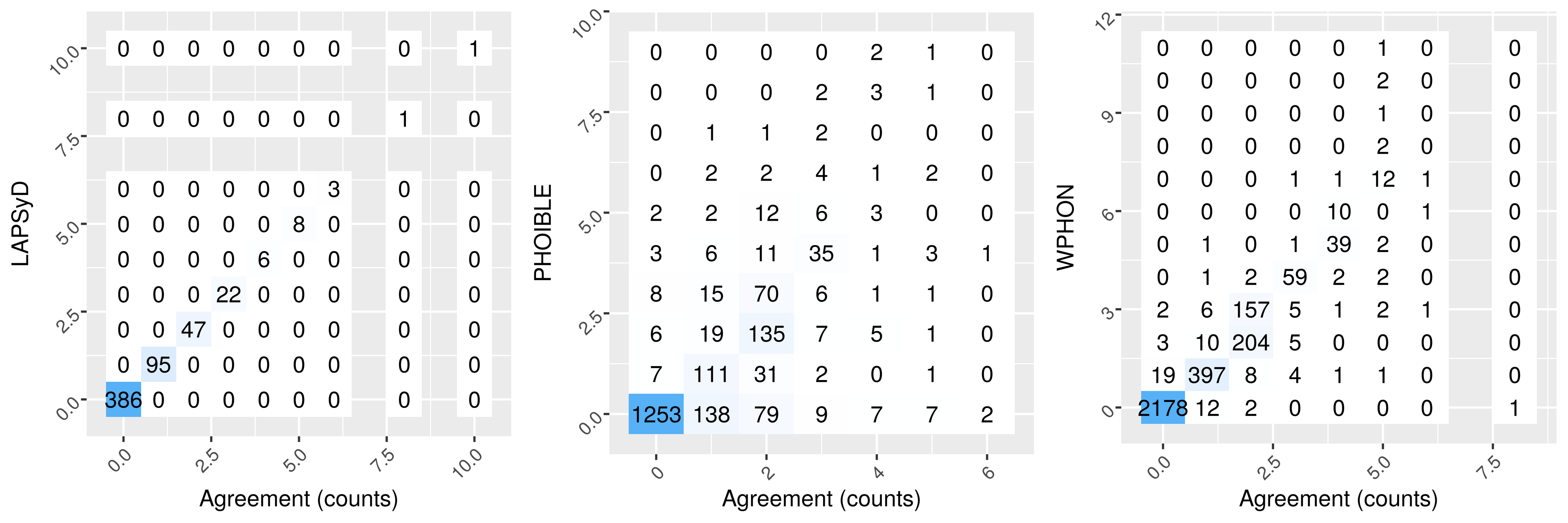

Supplement: S4 Fig — The same conventions as for S1 Fig. (TIF) [file pone.0253546.s004.tif]
