# Supplementary material for: Tone and genes: New cross-linguistic data and methods support the weak negative effect of the “derived” allele of ASPM on tone, but not of Microcephalin
Source: PLoS One. 2021 Jun 30;16(6):e0253546. doi: 10.1371/journal.pone.0253546 (PMC8244921; doi:10.1371/journal.pone.0253546)
Supplement: S1 File — (HTML) [file pone.0253546.s005.html]

 

 

 

 
 
 


 


 Tone and genes: new cross-linguistic data and methods support the weak negative effect of the “derived” allele of ASPM on tone, but not of Microcephalin 

 
 
 
 
 
 
 
 
 
 
 
 
 
 
 
 
 
 

 

 
 


 


 


 

 

 


 


 

 


 


 
 
 
 
 
 

 


 


 Tone and genes: new cross-linguistic data and methods support the weak negative effect of the “derived” allele of  ASPM  on tone, but not of  Microcephalin  
 Full analysis report 
 Dan Dediu ( dan.dediu@univ-lyon2.fr ) 
 Sun May 16 13:38:58 2021 

 


 
 Introduction 
 This  Rmarkdown  script contains the full results supporting the main paper (but very little interpretation). As described in detail in the  README.md  document, this script uses various types of input data (linguistic and genetic) and multiple methods of analysis. 
 
 
 Data 
 
 Tone 
 
 The data sources 
 
  WALS  
 WALS uses a  categorical  classification with 3 ordered categories ‘None’ < ‘Simple’ < ‘Complex.’ There are 513 languages with data. 
 
 
 
 
 
 
 
 
 None 
 Simple 
 Complex 
 
 
 
 
 301 
 127 
 85 
 
 
 
 
 
 
  Figure 1.    Distribution of tone in WALS. 
 
 
 
 
  LAPSyD  
 LAPSyD gives both a  categorical  classification with 5 ordered categories ‘None’ < ‘Simple’ < ‘Complex,’ and the actual  count  of tones. There are 569 languages with data. 
 
 
 
 
 
 
 
 
 
 
 None 
 Marginal 
 Simple 
 Moderately complex 
 Complex 
 
 
 
 
 386 
 8 
 94 
 39 
 42 
 
 
 
 
 
 
  Figure 2.    Distribution of tone in LAPSyD. 
 
 
 
 
 
  Figure 3.    Distribution of tone counts in LAPSyD. 
 
 
 
 
  Dediu & Ladd (2007)  
 This uses a  categorical  classification with 2 (presence/absence) categories ‘No’ and ‘Yes.’ There are 60 languages with data. 
 
 
 
 
 
 
 
 No 
 Yes 
 
 
 
 
 30 
 30 
 
 
 
 
 
 
  Figure 4.    Distribution of tone in Dediu & Ladd (2007)’s database. 
 
 
 
 
  PHOIBLE  
 PHOIBLE gives the actual  count  in 2030 languages with data. 
 
 
 
 
 
 
 
 
 
 
 
 
 
 
 
 
 0 
 1 
 2 
 3 
 4 
 5 
 6 
 7 
 8 
 9 
 10 
 
 
 
 
 1495 
 4 
 148 
 173 
 101 
 60 
 25 
 11 
 4 
 6 
 3 
 
 
 
 
 
 
  Figure 5.    Distribution of tone in PHOIBLE. 
 
 
 
 
  WPHON  
 WPHON gives the actual  count  in 3160 languages with data. 
 
 
 
 
 
 
 
 
 
 
 
 
 
 
 
 
 
 
 0 
 1 
 2 
 3 
 4 
 5 
 6 
 7 
 8 
 9 
 10 
 11 
 12 
 
 
 
 
 2193 
 3 
 427 
 222 
 174 
 66 
 43 
 11 
 15 
 2 
 1 
 2 
 1 
 
 
 
 
 
 
  Figure 6.    Distribution of tone in WPHON 
 
 
 
 
 
 Relationships between data sources 
 
  WALS  -  LAPSyD  
 
 languages with values in  at least one  classification: 724 
  shared  languages: 358 
 language with values  only in WALS : 155 
 language with values  only in LAPSyD : 211 
 
 
 
 
 
 
 
 
 
 
 
 
   
 None 
 Marginal 
 Simple 
 Moderately complex 
 Complex 
 
 
 
 
  None  
 229 
 2 
 1 
 0 
 0 
 
 
  Simple  
 4 
 4 
 59 
 12 
 4 
 
 
  Complex  
 1 
 0 
 2 
 16 
 24 
 
 
 
 
 
 
  Figure 7.    Relationship between tone in WALS and LAPSyD. 
 
 
 
 Pearson’s Chi-squared test:  cooc_tab  
 
 
 
 
 
 
 
 Test statistic 
 df 
 P value 
 
 
 
 
 515.4 
 8 
 3.407e-106 * * * 
 
 
 
 
 Pearson’s Chi-squared test with simulated p-value (based on 10000 replicates):  cooc_tab  
 
 
 
 
 
 
 
 Test statistic 
 df 
 P value 
 
 
 
 
 515.4 
 NA 
 9.999e-05 * * * 
 
 
 
 
 
  WALS  -  Dediu & Ladd (2007)  
 
 languages with values in  at least one  classification: 550 
  shared  languages: 23 
 language with values  only in WALS : 490 
 language with values  only in Dediu & Ladd (2007) : 37 
 
 
 
 
 
 
 
 
 
   
 No 
 Yes 
 
 
 
 
  None  
 12 
 1 
 
 
  Simple  
 0 
 5 
 
 
  Complex  
 0 
 5 
 
 
 
 
 
 
  Figure 8.    Relationship between tone in WALS and Dediu & Ladd (2007)’s database. 
 
 
 
 Pearson’s Chi-squared test:  cooc_tab  
 
 
 
 
 
 
 
 Test statistic 
 df 
 P value 
 
 
 
 
 19.3 
 2 
 6.44e-05 * * * 
 
 
 
 
 Pearson’s Chi-squared test with simulated p-value (based on 10000 replicates):  cooc_tab  
 
 
 
 
 
 
 
 Test statistic 
 df 
 P value 
 
 
 
 
 19.3 
 NA 
 9.999e-05 * * * 
 
 
 
 
 
  LAPSyD  -  Dediu & Ladd (2007)  
 
 languages with values in  at least one  classification: 609 
  shared  languages: 20 
 language with values  only in LAPSyD : 549 
 language with values  only in Dediu & Ladd (2007) : 40 
 
 
 
 
 
 
 
 
 
   
 No 
 Yes 
 
 
 
 
  None  
 12 
 0 
 
 
  Marginal  
 0 
 1 
 
 
  Simple  
 0 
 1 
 
 
  Moderately complex  
 0 
 2 
 
 
  Complex  
 0 
 4 
 
 
 
 
 
 
  Figure 9.    Relationship between tone in LAPSyD and Dediu & Ladd (2007)’s database. 
 
 
 
 Pearson’s Chi-squared test:  cooc_tab  
 
 
 
 
 
 
 
 Test statistic 
 df 
 P value 
 
 
 
 
 20 
 4 
 0.0004994 * * * 
 
 
 
 
 Pearson’s Chi-squared test with simulated p-value (based on 10000 replicates):  cooc_tab  
 
 
 
 
 
 
 
 Test statistic 
 df 
 P value 
 
 
 
 
 20 
 NA 
 9.999e-05 * * * 
 
 
 
 
 
  PHOIBLE  -  WALS  
 
 languages with values in  at least one  classification: 2074 
  shared  languages: 469 
 language with values  only in PHOIBLE : 1561 
 language with values  only in WALS : 44 
 
 
 
 
 
 
 
 
 
 
   
 None 
 Simple 
 Complex 
 
 
 
 
  0  
 272 
 70 
 41 
 
 
  1  
 1 
 0 
 0 
 
 
  2  
 1 
 17 
 4 
 
 
  3  
 2 
 7 
 12 
 
 
  4  
 0 
 6 
 6 
 
 
  5  
 0 
 4 
 7 
 
 
  6  
 1 
 6 
 3 
 
 
  7  
 0 
 1 
 2 
 
 
  8  
 0 
 0 
 1 
 
 
  9  
 0 
 0 
 3 
 
 
  10  
 0 
 0 
 2 
 
 
 
 
 
 
  Figure 10.    Relationship between tone in PHOIBLE and WALS (barplot). 
 
 
 
 
 
  Figure 11.    Relationship between tone in PHOIBLE and WALS (boxplots). 
 
 
 
 Analysis of Variance Model 
 
 
 
 
 
 
 
 
 
 
   
 Df 
 Sum Sq 
 Mean Sq 
 F value 
 Pr(>F) 
 
 
 
 
  wa_tone  
 2 
 371.4 
 185.7 
 76.53 
 1.826e-29 
 
 
  Residuals  
 466 
 1131 
 2.427 
 NA 
 NA 
 
 
 
 
 
 
 
 
 
 
 
 
 
   
 diff 
 lwr 
 upr 
 p adj 
 
 
 
 
  Simple-None  
 1.225 
 0.8137 
 1.637 
 3.976e-11 
 
 
  Complex-None  
 2.292 
 1.829 
 2.754 
 1.3e-11 
 
 
  Complex-Simple  
 1.066 
 0.5312 
 1.602 
 1.099e-05 
 
 
 
 
 
  PHOIBLE  -  LAPSyD  
 
 languages with values in  at least one  classification: 2132 
  shared  languages: 467 
 language with values  only in PHOIBLE : 1563 
 language with values  only in LAPSyD : 102 
 
 
 
 
 
 
 
 
 
 
 
 
   
 None 
 Marginal 
 Simple 
 Moderately complex 
 Complex 
 
 
 
 
  0  
 314 
 6 
 57 
 13 
 15 
 
 
  1  
 1 
 0 
 0 
 0 
 0 
 
 
  2  
 2 
 1 
 13 
 0 
 1 
 
 
  3  
 0 
 0 
 3 
 9 
 3 
 
 
  4  
 0 
 0 
 2 
 4 
 2 
 
 
  5  
 0 
 0 
 1 
 3 
 6 
 
 
  6  
 1 
 0 
 1 
 3 
 0 
 
 
  7  
 0 
 0 
 1 
 0 
 1 
 
 
  8  
 0 
 0 
 0 
 0 
 1 
 
 
  9  
 0 
 0 
 0 
 0 
 2 
 
 
  10  
 0 
 0 
 0 
 0 
 1 
 
 
 
 
 
 
  Figure 12.    Relationship between tone in PHOIBLE and LAPSyD (barplot). 
 
 
 
 
 
  Figure 13.    Relationship between tone in PHOIBLE and LAPSyD (boxplots). 
 
 
 
 Analysis of Variance Model 
 
 
 
 
 
 
 
 
 
 
   
 Df 
 Sum Sq 
 Mean Sq 
 F value 
 Pr(>F) 
 
 
 
 
  la_tone  
 4 
 368.3 
 92.06 
 61.43 
 1.324e-41 
 
 
  Residuals  
 462 
 692.3 
 1.499 
 NA 
 NA 
 
 
 
 
 
 
 
 
 
 
 
 
 
   
 diff 
 lwr 
 upr 
 p adj 
 
 
 
 
  Marginal-None  
 0.2511 
 -1.03 
 1.532 
 0.9835 
 
 
  Simple-None  
 0.7475 
 0.3239 
 1.171 
 1.812e-05 
 
 
  Moderately complex-None  
 2.34 
 1.719 
 2.962 
 4.919e-12 
 
 
  Complex-None  
 2.84 
 2.219 
 3.462 
 4.874e-12 
 
 
  Simple-Marginal  
 0.4963 
 -0.8264 
 1.819 
 0.8425 
 
 
  Moderately complex-Marginal  
 2.089 
 0.6904 
 3.488 
 0.0004863 
 
 
  Complex-Marginal  
 2.589 
 1.19 
 3.988 
 5.735e-06 
 
 
  Moderately complex-Simple  
 1.593 
 0.8892 
 2.297 
 1.263e-08 
 
 
  Complex-Simple  
 2.093 
 1.389 
 2.797 
 4.965e-12 
 
 
  Complex-Moderately complex  
 0.5 
 -0.3381 
 1.338 
 0.4765 
 
 
 
 
 
 
  Figure 14.    Relationship between tone in PHOIBLE and number of tones in LAPSyD (jittered for increased visibility). 
 
 
 
 Pearson’s product-moment correlation:  la_n_tones  and  ph_n_tones  
 
 
 
 
 
 
 
 
 
 Test statistic 
 df 
 P value 
 Alternative hypothesis 
 cor 
 
 
 
 
 13.86 
 465 
 8.444e-37 * * * 
 two.sided 
 0.5406 
 
 
 
 
 Spearman’s rank correlation rho:  la_n_tones  and  ph_n_tones  
 
 
 
 
 
 
 
 
 Test statistic 
 P value 
 Alternative hypothesis 
 rho 
 
 
 
 
 7516233 
 1.916e-39 * * * 
 two.sided 
 0.5572 
 
 
 
 
 
  PHOIBLE  -  Dediu & Ladd (2007)  
 
 languages with values in  at least one  classification: 2050 
  shared  languages: 40 
 language with values  only in PHOIBLE : 1990 
 language with values  only in Dediu & Ladd (2007) : 20 
 
 
 
 
 
 
 
 
 
   
 No 
 Yes 
 
 
 
 
  0  
 20 
 7 
 
 
  1  
 0 
 0 
 
 
  2  
 1 
 3 
 
 
  3  
 0 
 3 
 
 
  4  
 0 
 2 
 
 
  5  
 0 
 3 
 
 
  6  
 0 
 0 
 
 
  7  
 0 
 0 
 
 
  8  
 0 
 1 
 
 
  9  
 0 
 0 
 
 
  10  
 0 
 0 
 
 
 
 
 
 
  Figure 15.    Relationship between tone in PHOIBLE and Dediu & Ladd (2007)’s database (barplot). 
 
 
 
 
 
  Figure 16.    Relationship between tone in PHOIBLE and Dediu & Ladd (2007)’s database (boxplots). 
 
 
 
 Welch Two Sample t-test:  ph_n_tones  by  dl_tone  (continued below) 
 
 
 
 
 
 
 
 
 Test statistic 
 df 
 P value 
 Alternative hypothesis 
 
 
 
 
 -4.264 
 19.13 
 0.0004133 * * * 
 two.sided 
 
 
 
 
 
 
 
 
 
 
 mean in group No 
 mean in group Yes 
 
 
 
 
 0.09524 
 2.421 
 
 
 
 
 
  WPHON  -  WALS  
 
 languages with values in  at least one  classification: 3188 
  shared  languages: 485 
 language with values  only in WPHON : 2675 
 language with values  only in WALS : 28 
 
 
 
 
 
 
 
 
 
 
   
 None 
 Simple 
 Complex 
 
 
 
 
  0  
 270 
 9 
 3 
 
 
  1  
 0 
 0 
 0 
 
 
  2  
 13 
 73 
 9 
 
 
  3  
 6 
 24 
 18 
 
 
  4  
 1 
 11 
 20 
 
 
  5  
 0 
 2 
 10 
 
 
  6  
 0 
 1 
 8 
 
 
  7  
 0 
 0 
 2 
 
 
  8  
 0 
 0 
 3 
 
 
  9  
 0 
 0 
 1 
 
 
  10  
 0 
 0 
 0 
 
 
  11  
 0 
 0 
 0 
 
 
  12  
 0 
 0 
 1 
 
 
 
 
 
 
  Figure 17.    Relationship between tone in WPHON and WALS (barplot). 
 
 
 
 
 
  Figure 18.    Relationship between tone in WPHON and WALS (boxplots). 
 
 
 
 Analysis of Variance Model 
 
 
 
 
 
 
 
 
 
 
   
 Df 
 Sum Sq 
 Mean Sq 
 F value 
 Pr(>F) 
 
 
 
 
  wa_tone  
 2 
 1094 
 546.8 
 490 
 7.358e-117 
 
 
  Residuals  
 482 
 537.9 
 1.116 
 NA 
 NA 
 
 
 
 
 
 
 
 
 
 
 
 
 
   
 diff 
 lwr 
 upr 
 p adj 
 
 
 
 
  Simple-None  
 2.151 
 1.882 
 2.421 
 5.838e-11 
 
 
  Complex-None  
 3.954 
 3.633 
 4.276 
 5.838e-11 
 
 
  Complex-Simple  
 1.803 
 1.438 
 2.169 
 5.838e-11 
 
 
 
 
 
  WPHON  -  LAPSyD  
 
 languages with values in  at least one  classification: 3211 
  shared  languages: 518 
 language with values  only in WPHON : 2642 
 language with values  only in LAPSyD : 51 
 
 
 
 
 
 
 
 
 
 
 
 
   
 None 
 Marginal 
 Simple 
 Moderately complex 
 Complex 
 
 
 
 
  0  
 334 
 1 
 11 
 2 
 1 
 
 
  1  
 0 
 0 
 1 
 0 
 0 
 
 
  2  
 19 
 6 
 49 
 6 
 6 
 
 
  3  
 3 
 0 
 11 
 20 
 6 
 
 
  4  
 2 
 0 
 6 
 6 
 9 
 
 
  5  
 0 
 0 
 2 
 1 
 7 
 
 
  6  
 0 
 0 
 1 
 0 
 4 
 
 
  7  
 0 
 0 
 0 
 0 
 1 
 
 
  8  
 0 
 0 
 0 
 0 
 3 
 
 
  9  
 0 
 0 
 0 
 0 
 0 
 
 
  10  
 0 
 0 
 0 
 0 
 0 
 
 
  11  
 0 
 0 
 0 
 0 
 0 
 
 
  12  
 0 
 0 
 0 
 0 
 0 
 
 
 
 
 
 
  Figure 19.    Relationship between tone in WPHON and LAPSyD (barplot). 
 
 
 
 
 
  Figure 20.    Relationship between tone in WPHON and LAPSyD (boxplots). 
 
 
 
 Analysis of Variance Model 
 
 
 
 
 
 
 
 
 
 
   
 Df 
 Sum Sq 
 Mean Sq 
 F value 
 Pr(>F) 
 
 
 
 
  la_tone  
 4 
 868.5 
 217.1 
 278.1 
 6.119e-127 
 
 
  Residuals  
 513 
 400.6 
 0.7808 
 NA 
 NA 
 
 
 
 
 
 
 
 
 
 
 
 
 
   
 diff 
 lwr 
 upr 
 p adj 
 
 
 
 
  Marginal-None  
 1.561 
 0.6375 
 2.484 
 4.595e-05 
 
 
  Simple-None  
 1.97 
 1.672 
 2.267 
 2.014e-10 
 
 
  Moderately complex-None  
 2.732 
 2.304 
 3.16 
 2.014e-10 
 
 
  Complex-None  
 4.063 
 3.645 
 4.48 
 2.014e-10 
 
 
  Simple-Marginal  
 0.4092 
 -0.5438 
 1.362 
 0.7655 
 
 
  Moderately complex-Marginal  
 1.171 
 0.1699 
 2.173 
 0.01257 
 
 
  Complex-Marginal  
 2.502 
 1.505 
 3.499 
 3.885e-10 
 
 
  Moderately complex-Simple  
 0.7623 
 0.273 
 1.252 
 0.0002305 
 
 
  Complex-Simple  
 2.093 
 1.613 
 2.573 
 2.014e-10 
 
 
  Complex-Moderately complex  
 1.331 
 0.7601 
 1.901 
 4.023e-09 
 
 
 
 
 
 
  Figure 21.    Relationship between tone in WPHON and number of tones in LAPSyD (jittered for increased visibility). 
 
 
 
 Pearson’s product-moment correlation:  la_n_tones  and  wp_tone  
 
 
 
 
 
 
 
 
 
 Test statistic 
 df 
 P value 
 Alternative hypothesis 
 cor 
 
 
 
 
 31.51 
 516 
 2.475e-122 * * * 
 two.sided 
 0.8112 
 
 
 
 
 Spearman’s rank correlation rho:  la_n_tones  and  wp_tone  
 
 
 
 
 
 
 
 
 Test statistic 
 P value 
 Alternative hypothesis 
 rho 
 
 
 
 
 3591376 
 2.359e-142 * * * 
 two.sided 
 0.845 
 
 
 
 
 
  WPHON  -  Dediu & Ladd (2007)  
 
 languages with values in  at least one  classification: 3176 
  shared  languages: 44 
 language with values  only in WPHON : 3116 
 language with values  only in Dediu & Ladd (2007) : 16 
 
 
 
 
 
 
 
 
 
   
 No 
 Yes 
 
 
 
 
  0  
 22 
 1 
 
 
  1  
 0 
 0 
 
 
  2  
 1 
 6 
 
 
  3  
 0 
 7 
 
 
  4  
 0 
 4 
 
 
  5  
 0 
 0 
 
 
  6  
 0 
 1 
 
 
  7  
 0 
 2 
 
 
  8  
 0 
 0 
 
 
  9  
 0 
 0 
 
 
  10  
 0 
 0 
 
 
  11  
 0 
 0 
 
 
  12  
 0 
 0 
 
 
 
 
 
 
  Figure 22.    Relationship between tone in WPHON and Dediu & Ladd (2007)’s database (barplot). 
 
 
 
 
 
  Figure 23.    Relationship between tone in WPHON and Dediu & Ladd (2007)’s database (boxplots). 
 
 
 
 Welch Two Sample t-test:  wp_tone  by  dl_tone  (continued below) 
 
 
 
 
 
 
 
 
 Test statistic 
 df 
 P value 
 Alternative hypothesis 
 
 
 
 
 -8.362 
 22.18 
 2.64e-08 * * * 
 two.sided 
 
 
 
 
 
 
 
 
 
 
 mean in group No 
 mean in group Yes 
 
 
 
 
 0.08696 
 3.286 
 
 
 
 
 
  WPHON  -  PHOIBLE  
 
 languages with values in  at least one  classification: 3760 
  shared  languages: 1430 
 language with values  only in WPHON : 1730 
 language with values  only in PHOIBLE : 600 
 
 
 
 
 
 
 
 
 
 
 
 
 
 
 
 
 
 
   
 0 
 1 
 2 
 3 
 4 
 5 
 6 
 7 
 8 
 9 
 10 
 
 
 
 
  0  
 918 
 1 
 8 
 6 
 8 
 3 
 1 
 0 
 0 
 0 
 0 
 
 
  1  
 3 
 0 
 0 
 0 
 0 
 0 
 0 
 0 
 0 
 0 
 0 
 
 
  2  
 131 
 0 
 27 
 21 
 16 
 7 
 2 
 1 
 1 
 0 
 0 
 
 
  3  
 48 
 0 
 17 
 37 
 6 
 5 
 6 
 2 
 1 
 0 
 0 
 
 
  4  
 43 
 0 
 14 
 4 
 13 
 6 
 7 
 2 
 1 
 0 
 0 
 
 
  5  
 9 
 0 
 2 
 5 
 4 
 7 
 1 
 0 
 0 
 1 
 1 
 
 
  6  
 5 
 0 
 1 
 4 
 1 
 2 
 2 
 1 
 0 
 1 
 0 
 
 
  7  
 3 
 0 
 0 
 1 
 0 
 1 
 1 
 0 
 0 
 0 
 0 
 
 
  8  
 2 
 0 
 0 
 0 
 1 
 0 
 0 
 2 
 0 
 2 
 0 
 
 
  9  
 0 
 0 
 1 
 0 
 0 
 1 
 0 
 0 
 0 
 0 
 0 
 
 
  10  
 0 
 0 
 0 
 0 
 0 
 0 
 0 
 0 
 0 
 0 
 0 
 
 
  11  
 0 
 0 
 0 
 0 
 0 
 1 
 0 
 0 
 0 
 0 
 0 
 
 
  12  
 0 
 0 
 0 
 1 
 0 
 0 
 0 
 0 
 0 
 0 
 0 
 
 
 
 
 
 
  Figure 24.    Relationship between tone in WPHON and PHOIBLE (barplot). 
 
 
 
 
 
  Figure 25.    Relationship between tone in WPHON and PHOIBLE (scatterplot). 
 
 
 
 Pearson’s product-moment correlation:  wp_tone  and  ph_n_tones  
 
 
 
 
 
 
 
 
 
 Test statistic 
 df 
 P value 
 Alternative hypothesis 
 cor 
 
 
 
 
 26.83 
 1428 
 9.157e-129 * * * 
 two.sided 
 0.579 
 
 
 
 
 Spearman’s rank correlation rho:  wp_tone  and  ph_n_tones  
 
 
 
 
 
 
 
 
 Test statistic 
 P value 
 Alternative hypothesis 
 rho 
 
 
 
 
 1.97e+08 
 3.554e-138 * * * 
 two.sided 
 0.5959 
 
 
 
 
 
 
 Reconciliating the sources 
 
 Collapse  LAPSyD  4-way 
 The 5-level coding in  LAPSyD  is too fine-grained, especially “Marginal” is very rare, and seemingly quite similar with “Simple” (rather than “None”) in its behaviour in the other data sets. On the other hand, “Moderately complex,” while quite similar with “Complex” (but not “Simple”), seems to have an identity of its own. Thus, I collapsed “Marginal” into “Simple,” resulting in a 4-way classification: “None” < “Simple” < “Moderately complex” < “Complex.” 
 
 
 The content of the sources 
 With this (and as a reminder), the sources contain the following information: 
 
  LAPSyD :
 
  4-way classification  for 569 languages: “None” (386), “Simple” (102), “Moderately complex” (39), “Complex” (42) 
  counts  for 569 languages: 0 (386), 1 (6), 2 (89), 3 (47), 4 (22), 5 (6), 6 (8), 7 (3), 9 (1), 11 (1) 
  
  WALS :
 
  3-way classification  for 513 languages: “None” (301), “Simple” (127), “Complex” (85) 
  
  Dediu & Ladd (2007) :
 
  binary classification  for 60 languages: “No” (30), “Yes” (30) 
  
  WPHON :
 
  counts  for 3160 languages: 0 (2193), 1 (3), 2 (427), 3 (222), 4 (174), 5 (66), 6 (43), 7 (11), 8 (15), 9 (2), 10 (1), 11 (2), 12 (1) 
  
  PHOIBLE :
 
  counts  for 2030 languages: 0 (1495), 1 (4), 2 (148), 3 (173), 4 (101), 5 (60), 6 (25), 7 (11), 8 (4), 9 (6), 10 (3) 
  
 
 
 
 The reconciliation rules 
 I designed a set of rules for deciding on a set of two “agreement”  categorical  classifications, based on a precedence of the sources and the patterns of (dis)agreement between them: 
 
 a  binary  classification: no tone (“No”) vs any form of tone (“Yes”), and 
 a  3-way  classification: “None” < “Simple” < “Complex.” 
 
 More precisely, I preferred to use manually-curated categorical classifications to count sources, resulting in the following (rough) ordering:  LAPSyD  >  WALS  >  Dediu & Ladd (2007)  >  WPHON  >  PHOIBLE . 
 For the sources that give actual numbers (i.e.,  counts  of tones or tone symbols), we observe that  1  is very rare, probably signalling coding errors, marginal systems (“pitch-accent”) or theoretical arguments, so they can probably be safely collapsed it into  2 , and then move everything “one step down” (i.e., 2 → 1, 3 → 2, etc) so we have a continuum of counts from 0 onward. With this, the pairwise correlations between the count sources become: 
 
 
 
  Figure 26.    Relationships between counts after merging 1 into 2 and moving everything down by 1. 
 
 
 Thus, the main idea is to use  LAPSyD  wherever these data exists, followed by  WPHON  and finally  PHOIBLE  (thus with precedence  LAPSyD  >  WPHON  >  PHOIBLE ). Please note that the counts in  WPHON  and  PHOIBLE  are “corrected” to better map on those in  LAPSyD  and to “predict” missing data, using quadratic regression (i.e., the “corrected” counts are computed as  WPHON  corr  = 0.079 +0.919 WPHON  -0.04 WPHON  2 , and  PHOIBLE  corr  = 0.394 +0.68 PHOIBLE  -0.037 PHOIBLE  2 , respectively). 
 
 
 The “agreement” classifications 
 
 Distributions 
 
 Binary classification 
 # languages with data: 3798: 
 
 
 
 
 
 
 
 No 
 Yes 
 
 
 
 
 2541 
 1257 
 
 
 
 
 
 
  Figure 27.    Distribution of the binary agreement classification of tone. 
 
 
 
 
 3-way classification 
 # languages with data: 3785: 
 
 
 
 
 
 
 
 
 None 
 Simple 
 Complex 
 
 
 
 
 2538 
 936 
 311 
 
 
 
 
 
 
  Figure 28.    Distribution of the 3-way agreement classification of tone. 
 
 
 
 
 Counts 
  Rounded  
 # languages with data: 3785: 
 
 
 
 
 
 
 
 
 
 
 
 
 
 
 0 
 1 
 2 
 3 
 4 
 5 
 6 
 8 
 10 
 
 
 
 
 2544 
 516 
 524 
 114 
 56 
 26 
 3 
 1 
 1 
 
 
 
 
 
 
  Figure 29.    Distribution of the agreement counts of tone. 
 
 
  Unrounded  
 # languages with data: 3785: 
 
 
 
  Figure 30.    Distribution of the agreement counts of tone (unrounded). 
 
 
 
 
 
 Relationships with original sources 
 
 
 With  WALS  
 
 Binary classification 
 
 
 
 
 
 
 
 
   
 No 
 Yes 
 
 
 
 
  None  
 297 
 4 
 
 
  Simple  
 4 
 123 
 
 
  Complex  
 0 
 85 
 
 
 
 
 
 
  Figure 31.    Relationship between tone in WALS and the agreement binary classification. 
 
 
 
 Pearson’s Chi-squared test:  cooc_tab  
 
 
 
 
 
 
 
 Test statistic 
 df 
 P value 
 
 
 
 
 480.7 
 2 
 4.049e-105 * * * 
 
 
 
 
 Pearson’s Chi-squared test with simulated p-value (based on 10000 replicates):  cooc_tab  
 
 
 
 
 
 
 
 Test statistic 
 df 
 P value 
 
 
 
 
 480.7 
 NA 
 9.999e-05 * * * 
 
 
 
 
 
 3-way classification 
 
 
 
 
 
 
 
 
 
   
 None 
 Simple 
 Complex 
 
 
 
 
  None  
 298 
 3 
 0 
 
 
  Simple  
 4 
 119 
 4 
 
 
  Complex  
 1 
 2 
 82 
 
 
 
 
 
 
  Figure 32.    Relationship between tone in WALS and the agreement 3-way classification. 
 
 
 
 Pearson’s Chi-squared test:  cooc_tab  
 
 
 
 
 
 
 
 Test statistic 
 df 
 P value 
 
 
 
 
 921 
 4 
 4.723e-198 * * * 
 
 
 
 
 Pearson’s Chi-squared test with simulated p-value (based on 10000 replicates):  cooc_tab  
 
 
 
 
 
 
 
 Test statistic 
 df 
 P value 
 
 
 
 
 921 
 NA 
 9.999e-05 * * * 
 
 
 
 
 
 
 With  LAPSyD  
 
 Binary classification 
 
 
 
 
 
 
 
 
   
 No 
 Yes 
 
 
 
 
  None  
 385 
 1 
 
 
  Simple  
 0 
 102 
 
 
  Moderately complex  
 0 
 39 
 
 
  Complex  
 0 
 42 
 
 
 
 
 
 
  Figure 33.    Relationship between tone in LAPSyD and the agreement binary classification. 
 
 
 
 Pearson’s Chi-squared test:  cooc_tab  
 
 
 
 
 
 
 
 Test statistic 
 df 
 P value 
 
 
 
 
 564.4 
 3 
 5.148e-122 * * * 
 
 
 
 
 Pearson’s Chi-squared test with simulated p-value (based on 10000 replicates):  cooc_tab  
 
 
 
 
 
 
 
 Test statistic 
 df 
 P value 
 
 
 
 
 564.4 
 NA 
 9.999e-05 * * * 
 
 
 
 
 
 3-way classification 
 
 
 
 
 
 
 
 
 
   
 None 
 Simple 
 Complex 
 
 
 
 
  None  
 386 
 0 
 0 
 
 
  Simple  
 0 
 102 
 0 
 
 
  Moderately complex  
 0 
 12 
 27 
 
 
  Complex  
 0 
 0 
 42 
 
 
 
 
 
 
  Figure 34.    Relationship between tone in LAPSyD and the agreement 3-way classification. 
 
 
 
 Pearson’s Chi-squared test:  cooc_tab  
 
 
 
 
 
 
 
 Test statistic 
 df 
 P value 
 
 
 
 
 1028 
 6 
 7.755e-219 * * * 
 
 
 
 
 Pearson’s Chi-squared test with simulated p-value (based on 10000 replicates):  cooc_tab  
 
 
 
 
 
 
 
 Test statistic 
 df 
 P value 
 
 
 
 
 1028 
 NA 
 9.999e-05 * * * 
 
 
 
 
 
 Counts 
  Rounded  
 
 
 
  Figure 35.    Relationship between tone in LAPSyD and the agreement counts. 
 
 
 
 Pearson’s product-moment correlation:  la_n_tones  and  n_tones  
 
 
 
 
 
 
 
 
 
 Test statistic 
 df 
 P value 
 Alternative hypothesis 
 cor 
 
 
 
 
 Inf 
 567 
 0 * * * 
 two.sided 
 1 
 
 
 
 
 Spearman’s rank correlation rho:  la_n_tones  and  n_tones  
 
 
 
 
 
 
 
 
 Test statistic 
 P value 
 Alternative hypothesis 
 rho 
 
 
 
 
 0 
 0 * * * 
 two.sided 
 1 
 
 
 
  Unrounded  
 
 
 
  Figure 36.    Relationship between tone in LAPSyD and the agreement counts (unrounded). 
 
 
 
 Pearson’s product-moment correlation:  la_n_tones  and  n_tones_raw  
 
 
 
 
 
 
 
 
 
 Test statistic 
 df 
 P value 
 Alternative hypothesis 
 cor 
 
 
 
 
 Inf 
 567 
 0 * * * 
 two.sided 
 1 
 
 
 
 
 Spearman’s rank correlation rho:  la_n_tones  and  n_tones_raw  
 
 
 
 
 
 
 
 
 Test statistic 
 P value 
 Alternative hypothesis 
 rho 
 
 
 
 
 0 
 0 * * * 
 two.sided 
 1 
 
 
 
 
 
 
 With  Dediu & Ladd (2007)  
 
 Binary classification 
 
 
 
 
 
 
 
 
   
 No 
 Yes 
 
 
 
 
  No  
 30 
 0 
 
 
  Yes  
 0 
 30 
 
 
 
 
 
 
  Figure 37.    Relationship between tone in Dediu & Ladd (2007) and the agreement binary classification. 
 
 
 
 Pearson’s Chi-squared test with Yates’ continuity correction:  cooc_tab  
 
 
 
 
 
 
 
 Test statistic 
 df 
 P value 
 
 
 
 
 56.07 
 1 
 7.005e-14 * * * 
 
 
 
 
 Pearson’s Chi-squared test with simulated p-value (based on 10000 replicates):  cooc_tab  
 
 
 
 
 
 
 
 Test statistic 
 df 
 P value 
 
 
 
 
 60 
 NA 
 9.999e-05 * * * 
 
 
 
 
 
 3-way classification 
 
 
 
 
 
 
 
 
 
   
 None 
 Simple 
 Complex 
 
 
 
 
  No  
 24 
 0 
 0 
 
 
  Yes  
 2 
 8 
 13 
 
 
 
 
 
 
  Figure 38.    Relationship between tone in Dediu & Ladd (2007) and the agreement 3-way classification. 
 
 
 
 Pearson’s Chi-squared test:  cooc_tab  
 
 
 
 
 
 
 
 Test statistic 
 df 
 P value 
 
 
 
 
 39.61 
 2 
 2.502e-09 * * * 
 
 
 
 
 Pearson’s Chi-squared test with simulated p-value (based on 10000 replicates):  cooc_tab  
 
 
 
 
 
 
 
 Test statistic 
 df 
 P value 
 
 
 
 
 39.61 
 NA 
 9.999e-05 * * * 
 
 
 
 
 
 
 With  PHOIBLE  
 
 Counts 
  Rounded  
 
 
 
  Figure 39.    Relationship between tone in PHOIBLE and the agreement counts. 
 
 
 
 Pearson’s product-moment correlation:  ph_n_tones  and  n_tones  
 
 
 
 
 
 
 
 
 
 Test statistic 
 df 
 P value 
 Alternative hypothesis 
 cor 
 
 
 
 
 41.42 
 2028 
 2.854e-272 * * * 
 two.sided 
 0.677 
 
 
 
 
 Spearman’s rank correlation rho:  ph_n_tones  and  n_tones  
 
 
 
 
 
 
 
 
 Test statistic 
 P value 
 Alternative hypothesis 
 rho 
 
 
 
 
 358843117 
 0 * * * 
 two.sided 
 0.7426 
 
 
 
  Unrounded  
 
 
 
  Figure 40.    Relationship between tone in PHOIBLE and the agreement counts (unrounded). 
 
 
 
 Pearson’s product-moment correlation:  ph_n_tones  and  n_tones_raw  
 
 
 
 
 
 
 
 
 
 Test statistic 
 df 
 P value 
 Alternative hypothesis 
 cor 
 
 
 
 
 38.8 
 2028 
 9.067e-247 * * * 
 two.sided 
 0.6527 
 
 
 
 
 Spearman’s rank correlation rho:  ph_n_tones  and  n_tones_raw  
 
 
 
 
 
 
 
 
 Test statistic 
 P value 
 Alternative hypothesis 
 rho 
 
 
 
 
 488625970 
 1.276e-243 * * * 
 two.sided 
 0.6495 
 
 
 
 
 
 
 With  WPHON  
 
 Counts 
  Rounded  
 
 
 
  Figure 41.    Relationship between tone in WPHON and the agreement counts. 
 
 
 
 Pearson’s product-moment correlation:  wp_tone  and  n_tones  
 
 
 
 
 
 
 
 
 
 Test statistic 
 df 
 P value 
 Alternative hypothesis 
 cor 
 
 
 
 
 156 
 3158 
 0 * * * 
 two.sided 
 0.9408 
 
 
 
 
 Spearman’s rank correlation rho:  wp_tone  and  n_tones  
 
 
 
 
 
 
 
 
 Test statistic 
 P value 
 Alternative hypothesis 
 rho 
 
 
 
 
 136411420 
 0 * * * 
 two.sided 
 0.9741 
 
 
 
  Unrounded  
 
 
 
  Figure 42.    Relationship between tone in WPHON and the agreement counts (unrounded). 
 
 
 
 Pearson’s product-moment correlation:  wp_tone  and  n_tones_raw  
 
 
 
 
 
 
 
 
 
 Test statistic 
 df 
 P value 
 Alternative hypothesis 
 cor 
 
 
 
 
 168.4 
 3158 
 0 * * * 
 two.sided 
 0.9486 
 
 
 
 
 Spearman’s rank correlation rho:  wp_tone  and  n_tones_raw  
 
 
 
 
 
 
 
 
 Test statistic 
 P value 
 Alternative hypothesis 
 rho 
 
 
 
 
 689289938 
 0 * * * 
 two.sided 
 0.8689 
 
 
 
 
 
 
 
 
 Conclusions about tone 
 These three agreement tone codings were obtain using the  full  information from the 5 sources, but, of course, we have information about much fewer languages for this study, so that we end up using fewer languages here. 
 After this sub-setting, in summary, I used 5 primary sources: 
 
  WALS : categorical with 3 ordered categories ‘None’ < ‘Simple’ < ‘Complex,’ 
  LAPSYD : categorical, recoded with 4 ordered categories ‘None’ < ‘Simple’ < ‘Moderately complex’ < ‘Complex’ by collapsing ‘Marginal’ into ‘Simple,’ and count, from 0 to 10 tones (mean 0.62 and median 0), by collapsing the original  1  tone into the original  2  tones and moving all tones one step down (i.e., original  2  tones become  1  tone), 
  Dediu & Ladd (2007) : categorical with 2 (presence/absence) categories ‘No’ and ‘Yes,’ 
  WPHON : count, from 0 to 11 tones (mean 0.67 and median 0), by collapsing the original  1  tone into the original  2  tones and moving all tones one step down (i.e., original  2  tones become  1  tone), and 
  PHOIBLE : count, from 0 to 9 tones (mean 0.66 and median 0), by collapsing the original  1  tone into the original  2  tones and moving all tones one step down (i.e., original  2  tones become  1  tone), 
 
 From these, I built 3 “agreement” combined and reconciled measures: 
 
  tone_binary : a binary (presence/absence) variable with categories ‘No’ and ‘Yes,’ 
  tone_3way : a categorical variable with 3 ordered categories ‘None’ < ‘Simple’ < ‘Complex,’ and 
  n_tones : count, from 0 to 10 tones (mean 0.61 and median 0). 
 
 However, for the analyses reported here, I used the following variables: 
 
  tone1 : this represents directly  tone_binary  and encapsulates the question “does the language use tone?” contrasting no tone (“No”) versus any type of tone system (“Yes”), 
  tone2 : this is the dichotomisation of  tone_3way  into the question “does the language use a complex tone system?” contrasting complex tone systems (“Yes”) versus no tone and simple tone systems (“No”), and 
 tone  counts : this is the  n_tones , counting the number of tones/tone symbols in the language. 
 
 For  counts , I will also use the  unrounded  (i.e., raw) “counts,”  n_tones_raw , varying between 0 to 10 tones (mean 0.67 and median 0.0793991), to avoid any biases induced by numerically rounding to integer counts. 
 
 Distribution of retained tone data 
 
 Binary 
 # languages with data: 321: 
 
 
 
 
 
 
 
 No 
 Yes 
 
 
 
 
 251 
 70 
 
 
 
 
 
 
  Figure 43.    Distribution of binary tone. 
 
 
 
 
 3-way 
 # languages with data: 314: 
 
 
 
 
 
 
 
 
 None 
 Simple 
 Complex 
 
 
 
 
 248 
 39 
 27 
 
 
 
 
 
 
  Figure 44.    Distribution of 3-way tone. 
 
 
 
 
 Counts 
 # languages with data: 314: 
 
 
 
 
 
 
 
 
 
 
 
 
 0 
 1 
 2 
 3 
 4 
 5 
 6 
 
 
 
 
 249 
 26 
 23 
 6 
 5 
 3 
 2 
 
 
 
 
 
 
  Figure 45.    Distribution of tone counts. 
 
 
 
 
 Counts (unrounded) 
 # languages with data: 314: 
 
 
 
  Figure 46.    Distribution of tone counts (unrounded). 
 
 
 
 
 Intersection 
 There are 314 languages with data for  binary ,  3-way  and  counts . 
 
 
 
 
 
  ASPM -D and  MCPH1 -D population frequencies 
 I will denote the “derived” alleles of  ASPM  and  MCPH1  ( Microcephalin ) as  ASPM -D and  MCPH1 -D, respectively. 
 
  ASPM -D 
  ASPM -D this was originally defined in relation to “haplotype 63” and two of its polymorphic nonsynonymous sites in exon 18 in an open reading frame (ORF),  A44871G  and  C45126A  with the ancestral alleles, respectively, A and C, and the derived ones, G and A  (Mekel-Bobrov et al., 2005, p. 1720) . Later relevant publications  (Patrick C. M. Wong, Chandrasekaran, & Zheng, 2012; Patrick C. M. Wong et al., 2020)  however, use SNP   rs41310927   with ancestral allele T and derived allele C. While most databases do contain info about this SNP, others do not, such that I also collected data about SNPs in very tight LD with it:  rs41308365 ,  rs3762271 ,  rs41304071 ,  rs147068597  and  rs61819087  (the LD data was obtained from  LDlink’s “LDproxy Tool”  using all populations in that database). 
 Thus, I collected the following data: 
 
 
 
 Locus/SNP 
 “derived” allele 
 Datatbases 
 Position and LD to target 
 
 
 
 
 “haplotype 63” 
 “haplogroup D” 
  MB2005  
 the target 
 
 
  rs41310927  
 C 
  WONG2020 ,  LDLink ,  gnomAD ,  dbSNP  
 the target 
 
 
  rs41308365  
 A 
  LDLink ,  gnomAD ,  dbSNP  
 chr1:197070707; D’=1.00, R 2 =1.00 
 
 
  rs3762271  
 T 
  LDLink ,  gnomAD ,  dbSNP ,  ALFRED  
 chr1:197070442; D’=1.00, R 2 =1.00 
 
 
  rs41304071  
 T 
  LDLink ,  dbSNP  
 chr1:197063352; D’=1.00, R 2 =1.00 
 
 
  rs147068597  
 A 
  LDLink  
 chr1:197058136; D’=1.00, R 2 =1.00 
 
 
  rs61819087  
 G 
  LDLink ,  dbSNP  
 chr1:197084857; D’=1.00, R 2 =1.00 
 
 
 
 where the databases are identified as: 
 
 
 
 
 
 
 
 
 
 Database 
 URL 
 Info 
 ID 
 
 
 
 
  Mekel-Bobrov et al. (2005)  
  https://science.sciencemag.org/content/309/5741/1720  
 The original source; 59 populations 
  MB2005  
 
 
  Patrick C. M. Wong et al. (2020)  
  https://advances.sciencemag.org/content/6/22/eaba5090  
 Massive experimental study in Cantonese speakers; 1 population 
  WONG2020  
 
 
 LDLink 
  https://ldlink.nci.nih.gov/?tab=home  
 “[…] a suite of web-based applications designed to easily and efficiently interrogate linkage disequilibrium in population groups”; 1000 genomes data in 32 individual and grouped populations 
  LDLink  
 
 
 gnomAD 
  https://gnomad.broadinstitute.org/  
 Genome Aggregation Database v2.1.1; very broad populations 
  gnomAD  
 
 
 dbSNP 
  https://www.ncbi.nlm.nih.gov/snp/  
 aggregation of info form multiple databases, mostly using very broad populations 
  dbSNP  
 
 
 1000 genomes 
  https://www.internationalgenome.org/  
 this info is included in other databases (gnomAD) so is not specifically used here 
  1KG  
 
 
 ALFRED 
  https://alfred.med.yale.edu/alfred/index.asp  
 The ALlele FREquency Database; lots of info in many populations; unfortunately, for ASPM only one SNP in strong LD with the target rs41310927 (rs3762271) is available 
  ALFRED  
 
 
 
 
 All SNPs 
 I ended up with frequency data about these loci in 170 unique samples coming from 127 unique  meta-populations  (such as “Han Chinese,” “Italians” or “Finnish”). After making sure the frequencies of these SNPs are very highly correlated (in those samples where they do co-occur), I computed their weighted average frequency (weighed by the number of sampled individuals). 
 
 
 
 
 
 
 
 
 
 
 
 Min. 
 1st Qu. 
 Median 
 Mean 
 3rd Qu. 
 Max. 
 
 
 
 
 0 
 0.1012 
 0.2291 
 0.2416 
 0.3886 
 0.684 
 
 
 
 
 
 
  Figure 48.    Distribution of the frequency of the “derived” allele of  ASPM  across the world. 
 
 
 
 
 Excluding “proxy” SNPs 
 Of these 7 SNPs, 5 are “proxy” SNPs ( rs147068597 ,  rs3762271 ,  rs41304071 ,  rs41308365 ,  rs61819087 ), representing 289 unique samples (and 233237 total alleles) out of 396 (73%) unique samples (and 367519 total alleles; 63.5%) available for  ASPM -D. 
 
 
 
 
 
 
 
 
 
 
 
 Min. 
 1st Qu. 
 Median 
 Mean 
 3rd Qu. 
 Max. 
 
 
 
 
 0 
 0.0995 
 0.2073 
 0.2275 
 0.38 
 0.6 
 
 
 
 
 
 
  Figure 50.    Distribution of the frequency of the “derived” allele of  ASPM  across the world, excluding the “proxy” SNPs. 
 
 
 Due to this high proportion of the data being represented by “proxy” SNPs, I also conducted separate analyses excluding these SNPs. 
 
 
 New samples 
 Moreover, 111 are new samples from 84 unique (meta)populations, compared to the 59 samples in 56 (meta)populations in the original  Mekel-Bobrov et al. (2005) . These new samples are distributed as: 
 
 
 
 
 
 
 
 
 
 Africa 
 Eurasia 
 America 
 Papunesia 
 
 
 
 
 12 
 90 
 5 
 4 
 
 
 
 and the corresponding new (meta)populations as: 
 
 
 
 
 
 
 
 
 
 Africa 
 Eurasia 
 America 
 Papunesia 
 
 
 
 
 12 
 63 
 5 
 4 
 
 
 
 
 
 
  Figure 51.    Distribution of the “original” and “new” samples of  ASPM -D across the world. 
 
 
 
 
 
  MCPH1 -D 
  MCPH1 -D was originally defined in relation to  G37995C  in exon 8 in an open reading frame (ORF) with the ancestral allele G, and the derived one C  (Evans et al., 2005, p. 1717) . Later relevant publications  (Patrick C. M. Wong et al., 2020)  however, use SNP   rs930557   with ancestral allele G and derived allele C. While most databases do contain info about this SNP, others do not, such that I also collected info about the SNP  rs1129706  which is in very tight LD with it (the linkage data was obtained from  LDlink’s “LDproxy Tool”  using all populations in that database). 
 Thus, I obtained the following data: 
 
 
 
 Locus/SNP 
 “derived” allele 
 Datatbases 
 Position and LD to target 
 
 
 
 
  G37995C  
 C 
  MB2005  
 the target 
 
 
  rs930557  
 C 
  WONG2020 ,  LDLink ,  dbSNP  
 the target 
 
 
  rs1129706  
 G 
  ALFRED  
 chr8:6304814; D’=0.995, R 2 =0.936 
 
 
 
 
 All SNPs 
 I ended up with frequency data about these loci in 166 unique samples coming from 128 unique meta-populations. After making sure the frequencies of these SNPs are very highly correlated (in those samples where they do co-occur), I computed their weighted average frequency (weighted by the number of sampled individuals). 
 
 
 
 
 
 
 
 
 
 
 
 Min. 
 1st Qu. 
 Median 
 Mean 
 3rd Qu. 
 Max. 
 
 
 
 
 0.0315 
 0.658 
 0.7986 
 0.7125 
 0.8652 
 1 
 
 
 
 
 
 
  Figure 53.    Distribution of the frequency of the “derived” allele of  MCPH1  across the world. 
 
 
 
 
 Excluding “proxy” SNPs 
 Of these 3 SNPs, 1 are “proxy” SNPs ( rs1129706 ), representing 141 unique samples (and 13028 total alleles) out of 245 (57.6%) unique samples (and 107258 total alleles; 12.1%) available for  MCPH1 -D. 
 
 
 
 
 
 
 
 
 
 
 
 Min. 
 1st Qu. 
 Median 
 Mean 
 3rd Qu. 
 Max. 
 
 
 
 
 0.033 
 0.5634 
 0.7737 
 0.6729 
 0.8357 
 1 
 
 
 
 
 
 
  Figure 55.    Distribution of the frequency of the “derived” allele of  MCPH1  across the world, excluding the “proxy” SNPs. 
 
 
 Due to this high proportion of the data being represented by “proxy” SNPs, I also conducted separate analyses excluding these SNPs. 
 
 
 New samples 
 Moreover, 107 are new samples from 85 unique (meta)populations, compared to the 59 samples in 56 (meta)populations in the original  Evans et al. (2005) . These new samples are distributed as: 
 
 
 
 
 
 
 
 
 
 Africa 
 Eurasia 
 America 
 Papunesia 
 
 
 
 
 12 
 86 
 5 
 4 
 
 
 
 and the corresponding new (meta)populations as: 
 
 
 
 
 
 
 
 
 
 Africa 
 Eurasia 
 America 
 Papunesia 
 
 
 
 
 12 
 64 
 5 
 4 
 
 
 
 
 
 
  Figure 56.    Distribution of the “original” and “new” samples of  MCPH1 -D across the world. 
 
 
 
 
 
 The original  Dediu & Ladd (2007)  samples 
 These are the same for  ASPM -D and  MCPH1 -D: 
 
 
 
 
 
 
 
 
 
 Africa 
 Eurasia 
 America 
 Papunesia 
 
 
 
 
 15 
 37 
 5 
 2 
 
 
 
 and the corresponding new (meta)populations as: 
 
 
 
 
 
 
 
 
 
 Africa 
 Eurasia 
 America 
 Papunesia 
 
 
 
 
 14 
 35 
 5 
 2 
 
 
 
 
 
 
  Figure 57.    Distribution of the “original” and “new” samples of  MCPH1 -D across the world. 
 
 
 
 
 
 Putting tone and genes together 
 When combining the linguistic and genetic data, we are left with 175 unique samples in 129 unique (meta)populations speaking 321 unique “languages” (i.e., Glottolog codes) (from now on, denoted as 175:129:321), of which: 
 
 
 
 
 
 
 
 
 Information for 
 Number of samples:(meta)pops:languages 
 Missing samples:(meta)pops:languages 
 
 
 
 
 tone  binary  
 175:129:321 
 0:0:0 = {} : {} : {} 
 
 
 tone  3-way  
 170:124:314 
 5:5:7 = {SA001471N, SA001477T, SA001487U, SA001491P, SA001681Q} : {Burunge, Hazara, Mozabite, Oroqen, Xibe} : {buru1320, efee1239, gyel1242, haza1239, oroq1238, tumz1238, xibe1242} 
 
 
 tone  counts  
 170:124:314 
 5:5:7 = {SA001471N, SA001477T, SA001487U, SA001491P, SA001681Q} : {Burunge, Hazara, Mozabite, Oroqen, Xibe} : {buru1320, efee1239, gyel1242, haza1239, oroq1238, tumz1238, xibe1242} 
 
 
  ASPM -D 
 170:127:319 
 5:2:2 = {FINRISK, GenDan, GenNed5, KRGDB, Qatari} : {Dutch, Qatari} : {dutc1256, gulf1241} 
 
 
  MCPH1 -D 
 166:128:320 
 9:1:1 = {gnomAD_asj, gnomAD_bgr, gnomAD_est, gnomAD_fin, gnomAD_jpn, gnomAD_kor, gnomAD_swe, gnomADexomes_AshkenaziJewish, gnomADgenomes_AshkenaziJewish} : {Bulgarian} : {bulg1262} 
 
 
  ASPM -D &  MCPH1 -D 
 161:126:318 
 14:3:3 = {FINRISK, GenDan, GenNed5, gnomAD_asj, gnomAD_bgr, gnomAD_est, gnomAD_fin, gnomAD_jpn, gnomAD_kor, gnomAD_swe, gnomADexomes_AshkenaziJewish, gnomADgenomes_AshkenaziJewish, KRGDB, Qatari} : {Bulgarian, Dutch, Qatari} : {bulg1262, dutc1256, gulf1241} 
 
 
 tone  binary  &  ASPM -D &  MCPH1 -D 
 161:126:318 
 14:3:3 = {FINRISK, GenDan, GenNed5, gnomAD_asj, gnomAD_bgr, gnomAD_est, gnomAD_fin, gnomAD_jpn, gnomAD_kor, gnomAD_swe, gnomADexomes_AshkenaziJewish, gnomADgenomes_AshkenaziJewish, KRGDB, Qatari} : {Bulgarian, Dutch, Qatari} : {bulg1262, dutc1256, gulf1241} 
 
 
 tone  3-way  &  ASPM -D &  MCPH1 -D 
 156:121:311 
 19:8:10 = {FINRISK, GenDan, GenNed5, gnomAD_asj, gnomAD_bgr, gnomAD_est, gnomAD_fin, gnomAD_jpn, gnomAD_kor, gnomAD_swe, gnomADexomes_AshkenaziJewish, gnomADgenomes_AshkenaziJewish, KRGDB, Qatari, SA001471N, SA001477T, SA001487U, SA001491P, SA001681Q} : {Bulgarian, Burunge, Dutch, Hazara, Mozabite, Oroqen, Qatari, Xibe} : {bulg1262, buru1320, dutc1256, efee1239, gulf1241, gyel1242, haza1239, oroq1238, tumz1238, xibe1242} 
 
 
 tone  counts  &  ASPM -D &  MCPH1 -D 
 156:121:311 
 19:8:10 = {FINRISK, GenDan, GenNed5, gnomAD_asj, gnomAD_bgr, gnomAD_est, gnomAD_fin, gnomAD_jpn, gnomAD_kor, gnomAD_swe, gnomADexomes_AshkenaziJewish, gnomADgenomes_AshkenaziJewish, KRGDB, Qatari, SA001471N, SA001477T, SA001487U, SA001491P, SA001681Q} : {Bulgarian, Burunge, Dutch, Hazara, Mozabite, Oroqen, Qatari, Xibe} : {bulg1262, buru1320, dutc1256, efee1239, gulf1241, gyel1242, haza1239, oroq1238, tumz1238, xibe1242} 
 
 
 
 Some pair-wise differences in terms of samples:(meta)populations:languages with data: 
 
 
 
 
 
 
 
 
 Present in… 
 … but absent from 
 samples:(meta)pops:languages 
 
 
 
 
 tone  binary  
 tone  3-way  (and  counts ) 
 5:5:7 = {SA001471N, SA001477T, SA001487U, SA001491P, SA001681Q} : {Burunge, Hazara, Mozabite, Oroqen, Xibe} : {buru1320, efee1239, gyel1242, haza1239, oroq1238, tumz1238, xibe1242} 
 
 
 tone  binary  
  ASPM -D 
 5:2:2 = {FINRISK, GenDan, GenNed5, KRGDB, Qatari} : {Dutch, Qatari} : {dutc1256, gulf1241} 
 
 
 tone  binary  
  MCPH1 -D 
 9:1:1 = {gnomAD_asj, gnomAD_bgr, gnomAD_est, gnomAD_fin, gnomAD_jpn, gnomAD_kor, gnomAD_swe, gnomADexomes_AshkenaziJewish, gnomADgenomes_AshkenaziJewish} : {Bulgarian} : {bulg1262} 
 
 
 tone  binary  
  ASPM -D &  MCPH1 -D 
 14:3:3 = {FINRISK, GenDan, GenNed5, gnomAD_asj, gnomAD_bgr, gnomAD_est, gnomAD_fin, gnomAD_jpn, gnomAD_kor, gnomAD_swe, gnomADexomes_AshkenaziJewish, gnomADgenomes_AshkenaziJewish, KRGDB, Qatari} : {Bulgarian, Dutch, Qatari} : {bulg1262, dutc1256, gulf1241} 
 
 
 tone  3-way  (and  counts ) 
  ASPM -D 
 5:2:2 = {FINRISK, GenDan, GenNed5, KRGDB, Qatari} : {Dutch, Qatari} : {dutc1256, gulf1241} 
 
 
 tone  3-way  (and  counts ) 
  MCPH1 -D 
 9:1:1 = {gnomAD_asj, gnomAD_bgr, gnomAD_est, gnomAD_fin, gnomAD_jpn, gnomAD_kor, gnomAD_swe, gnomADexomes_AshkenaziJewish, gnomADgenomes_AshkenaziJewish} : {Bulgarian} : {bulg1262} 
 
 
 tone  3-way  (and  counts ) 
  ASPM -D &  MCPH1 -D 
 14:3:3 = {FINRISK, GenDan, GenNed5, gnomAD_asj, gnomAD_bgr, gnomAD_est, gnomAD_fin, gnomAD_jpn, gnomAD_kor, gnomAD_swe, gnomADexomes_AshkenaziJewish, gnomADgenomes_AshkenaziJewish, KRGDB, Qatari} : {Bulgarian, Dutch, Qatari} : {bulg1262, dutc1256, gulf1241} 
 
 
 
 
 
 
 Stats 
 
  tone1  (is there tone?) 
 I kept only the entries with non-missing data for the  tone1 ,  ASPM -D and  MCPH1 -D, and if there are more than one possible languages or allele frequencies for a given sample, I only kept those entries that have different tone or allele data. The resulting dataset has 181 observations, distributed among 119 unique Glottolg codes in 35 families (ranging from a minimum of 1 language per family to a maximum of 48, with a mean 5.2 and median 2 languages per family) and 4 macroareas. 
 There are 161:126:119 unique samples:(meta)populations:languages retained, dropping 14:3:202 = {FINRISK, GenDan, GenNed5, gnomAD_asj, gnomAD_bgr, gnomAD_est, gnomAD_fin, gnomAD_jpn, gnomAD_kor, gnomAD_swe, gnomADexomes_AshkenaziJewish, gnomADgenomes_AshkenaziJewish, KRGDB, Qatari} : {Bulgarian, Dutch, Qatari} : {adze1240, ajie1238, amar1272, ambu1247, anei1239, apma1241, arak1252, arib1241, arop1243, aros1241, aulu1238, awtu1239, ayiw1239, baba1268, bahi1254, bann1247, bign1238, bili1260, boik1241, bulg1262, caro1242, cham1313, chek1238, chuu1238, dehu1237, dumb1241, dutc1256, east2443, east2447, fiji1243, futu1245, fwai1237, gapa1238, geez1241, gela1263, gilb1244, gulf1241, guma1254, hali1244, hang1263, hano1246, hmon1264, hoav1238, iaai1238, iatm1242, idak1243, idun1242, iris1253, iwam1256, juho1239, kaia1245, kair1263, kamb1297, kapi1249, kara1486, kaul1240, kela1255, kele1258, kiku1240, kili1267, kire1240, koko1269, kosr1238, kuan1247, kuan1248, kuma1276, kung1261, kwai1243, kwam1251, kwam1252, kwas1243, kwom1262, labu1248, lala1268, lame1260, lauu1247, lena1238, lese1243, lewo1242, long1395, loni1238, lonw1238, louu1245, lusi1240, maee1241, mais1250, male1289, malo1243, mana1295, mana1298, maor1246, mars1254, masa1299, matu1261, mbal1255, mbul1263, mehe1243, meke1243, mele1250, mina1269, ming1252, moch1256, moki1238, moks1248, mono1273, motl1237, motu1246, mudu1242, muri1260, muso1238, muss1246, muyu1244, naka1262, nali1244, nama1264, nami1256, natu1246, naur1243, ndon1254, neha1247, neng1238, ngan1300, niua1240, niue1239, nort2646, nort2836, nort2845, nuku1260, onto1237, paam1238, pate1247, patp1243, pile1238, ping1243, pohn1238, port1285, pulu1242, qima1242, raoo1244, rapa1244, renn1242, rotu1241, rovi1238, russ1264, saaa1240, saam1283, saka1289, sali1295, samo1305, sapo1253, scot1243, siar1238, siee1239, sina1266, sioo1240, sobe1238, sons1242, sout2642, sout2679, sout2807, sout2856, sout2866, sout2869, stan1318, sude1239, surs1246, tahi1242, tain1252, taki1248, tawa1275, tean1237, teop1238, tiga1245, tigr1271, tiri1258, toab1237, toba1266, toke1240, tong1325, tsot1241, tswa1253, tuam1242, tuml1238, tung1290, tuva1244, ulit1238, urav1235, urip1239, vinm1237, waim1251, wall1257, wata1253, west2500, west2519, woga1249, wole1240, xamt1239, xara1244, yabe1254, yess1239, yima1243, zulu1248}. 
 
 
 
 
 
 
 
 
 
 
 
   
 Africa 
 Eurasia 
 America 
 Papunesia 
 Sum 
 
 
 
 
  No  
 9 
 100 
 4 
 7 
 120 
 
 
  Yes  
 27 
 26 
 6 
 2 
 61 
 
 
  Sum  
 36 
 126 
 10 
 9 
 181 
 
 
 
 
 
 
  Figure 58.    Distribution of  tone1 . 
 
 
 
 
 
  Figure 59.    Map of  tone1 . 
 
 
 
 
 
  Figure 60.    Relationship between  tone1 ,  ASPM -D and  MCPH1 -D. 
 
 
 
 Regressions 
 
  glmer  
 
 All data 
 
  null model : R 2  = 0.0%  1  , ICC = 70.4%  2   (but generates warnings:  Model is nearly unidentifiable: very large eigenvalue ) 
  macroarea : R 2  = 23.3%,  p  macroarea/null  = 0.00082  3   
  ASPM :
 
 by itself: R 2  = 10.0%,  β  = -1.00 ± 0.37,  p  ASPM/null  = 0.0041 
 quadratic: R 2  = 10.7%,  β ASPM2   = -0.97 ± 0.38,  p  ASPM2/ASPM  = 0.6 
 with  macroarea : R 2  = 24.4%,  β  = -0.37 ± 0.45,  p  macroarea/ASPM  = 0.028,  p  ASPM/macroarea  = 0.42 
  
  MCPH1 :
 
 by itself: R 2  = 9.4%,  β  = -1.04 ± 0.39,  p  MCPH1/null  = 0.0064 
 quadratic: R 2  = 9.3%,  β MCPH12   = -1.01 ± 0.39,  p  MCPH12/MCPH1  = 0.21 
 with  macroarea : R 2  = 24.1%,  β  = -0.39 ± 0.57,  p  macroarea/MCPH1  = 0.021,  p  MCPH1/macroarea  = 0.5 
  
  both alleles  (no  macroarea ):
 
  ASPM  +  MCPH1 : R 2  = 13.4%,  β  ASPM  = -0.72 ± 0.40,  p  ASPM/MCPH1  = 0.074,  β  MCPH1  = -0.62 ± 0.40,  p  MCPH1/ASPM  = 0.12,  p  ASPM+MCPH1/null  = 0.0049, 
 interaction: R 2  = 13.2%,  p  ASPM:MCPH1/ASPM+MCPH1  = 0.86 
  
 
 
 
 Alleles on macroarea 
 To better understand this overlap between family, macroarea and the two “derived” alleles, I regressed (separately) the  ASPM -D and  MCPH1 -D on the  macroarea , using mixed-effects  beta regression  (after replacing all  \(0.0\)  values by  \(10^{-7}\)  and all  \(1.0\)  by  \(1.0-10^{-7}\) , respectively) with language family as random effect: 
 
 the alleles are very strongly clustered within  families :
 
  ASPM : ICC = 100.0% 
  MCPH1 : ICC = 100.0% 
  
  macroarea  predicts their distribution very strongly:
 
  ASPM :  p  = 3.4e-16, R 2  = 57.9% 
  MCPH1 :  p  = 3.1e-12, R 2  = 70.3% 
  
 separating Africa vs the rest of the world seems to drive most of this effect (both alleles have lower frequencies in Africa):
 
  ASPM :  p  = 2.3e-14, R 2  = 39.2% 
  MCPH1 :  p  = 3.2e-09, R 2  = 32.6% 
  
 
 
 
 Randomization 
 For these randomization analyses there are several important parameters: 
 
 
 
 
 
 
 
 
 Parameter 
 Meaning 
 Values 
 
 
 
 
  permute  
 what to permute? 
  nothing  = the original data 
 
 
  
  
  tone  = permute the tone variable 
 
 
  
  
  alleles-together  = permute the two alleles together 
 
 
  
  
  alleles-independent  = permute the two alleles separately, i.e., each is independently permuted 
 
 
  within  
 how are the permutations constrained? 
  unrestricted  = all the observations are freely permuted (i.e., there are no constraints, no structure in the data is preserved) 
 
 
  
  
  families  = only observations within the same language family are permuted (i.e., the structure of the families is preserved) 
 
 
  
  
  macroareas  = only observations within the same macroarea are permuted (i.e., the structure of the macroareas is preserved) 
 
 
  macroarea  
 how do we control for macroareas? 
  none  = no control for macroareas at all 
 
 
  
  
  fixef  = as fixed effects 
 
 
 
 I performed 1000 independent replications of each of these parameter combinations, and below are the distributions of the permuted values versus the original ones (i.e., those obtained on the original, non-permuted data). 
 
 Regressions on 1000 permuted data. The first 3 columns show the permutation constraints (if any), how the  macroarea  is considered (if at all), and what is permuted. The next columns show the percent of the permutations that, in order, have a better AIC compared to the original fit, are significantly better than the null model (thus testing the effect of both alleles simultaneously), have a significant effect of  ASPM -D, have a smaller effect ( β ) of  ASPM -D than the original fit, and the same for  MCPH1 -D. 
 
 
 Permute within 
 Macroarea 
 Permute 
 AIC 
 Signif. 
  p   ASPM -D  
  β   ASPM -D  
  p   MCPH1 -D  
  β   MCPH1 -D  
 
 
 
 
 unrestricted 
 none 
 tone 
 0% 
 4% 
 6% 
 0% 
 4% 
 0% 
 
 
 unrestricted 
 none 
 alleles-together 
 0% 
 5% 
 4% 
 0% 
 5% 
 2% 
 
 
 unrestricted 
 none 
 alleles-independent 
 1% 
 6% 
 6% 
 0% 
 6% 
 1% 
 
 
 unrestricted 
 fixef 
 tone 
 0% 
 5% 
 5% 
 8% 
 5% 
 25% 
 
 
 unrestricted 
 fixef 
 alleles-together 
 68% 
 6% 
 7% 
 15% 
 4% 
 23% 
 
 
 unrestricted 
 fixef 
 alleles-independent 
 68% 
 5% 
 6% 
 16% 
 6% 
 20% 
 
 
 macroareas 
 none 
 tone 
 0% 
 95% 
 42% 
 4% 
 86% 
 28% 
 
 
 macroareas 
 none 
 alleles-together 
 26% 
 76% 
 10% 
 7% 
 59% 
 73% 
 
 
 macroareas 
 none 
 alleles-independent 
 32% 
 83% 
 20% 
 15% 
 66% 
 78% 
 
 
 macroareas 
 fixef 
 tone 
 0% 
 7% 
 7% 
 11% 
 6% 
 29% 
 
 
 macroareas 
 fixef 
 alleles-together 
 65% 
 5% 
 5% 
 19% 
 5% 
 35% 
 
 
 macroareas 
 fixef 
 alleles-independent 
 66% 
 4% 
 5% 
 20% 
 4% 
 35% 
 
 
 families 
 none 
 tone 
 2% 
 16% 
 2% 
 3% 
 14% 
 36% 
 
 
 families 
 none 
 alleles-together 
 2% 
 11% 
 3% 
 5% 
 5% 
 16% 
 
 
 families 
 none 
 alleles-independent 
 2% 
 16% 
 13% 
 10% 
 12% 
 20% 
 
 
 families 
 fixef 
 tone 
 1% 
 8% 
 3% 
 16% 
 11% 
 74% 
 
 
 families 
 fixef 
 alleles-together 
 66% 
 4% 
 5% 
 46% 
 2% 
 16% 
 
 
 families 
 fixef 
 alleles-independent 
 66% 
 3% 
 5% 
 37% 
 3% 
 22% 
 
 
 
 
 
 
Regressions on 1000 permuted data. Each plot shows the original result (vertical dashed black line) and the distribution of the permutations for the three possible things to be permuted (colored curves) for each combination of permutation constraints (horizontal panels) and control for  macroarea  (vertical panels) in terms of the effect size  β ;  ASPM -D is on the left and  MCPH1 -D on the right. The vertical dotted black thin line is at 0.0.
 
 
 
 
 Restricted sampling 
 
 
 
  Figure 61.    Results for 1000 restricted samplings. For  ASPM -D (left): 100% of βs are negative when regressing tone on  ASPM  alone (one-sided  t -test < 0:  t (999) = -81.1, mean = -0.81,  p  = 0), 82.7%, when controlling for the macroarea ( t (999) = -32.0, mean = -0.45,  p  = 2.2e-155), and 82.2% when controlling for both macroarea and  MCPH1  ( t (999) = -30.5, mean = -0.45,  p  = 4.9e-145). For  MCPH1 -D (right): 100% of βs are negative when regressing tone on  MCPH1  alone (one-sided  t -test < 0:  t (999) = -107.4, mean = -0.59,  p  = 0), 68.7% when controlling for the macroarea ( t (999) = -15.5, mean = -0.38,  p  = 3.9e-49), and 67.6% when controlling for both macroarea and  ASPM  ( t (999) = -14.3, mean = -0.37,  p  = 1.7e-42). 
 
 
 
 
 
  brms  
  tone1  on  ASPM -D and  MCPH1 -D in a mixed-effects Bayesian framework (using  brms ) with  macroarea , language  family  and  (meta)population  as (nested) random effects. The  ROPE  is the region of practical equivalence around 0.0, usually [-0.1, 0.1] but may vary by regression type" the idea is that the HDI should have an as small intersection as possible with the ROPE. Another take is represented by the  p  ROPE  which is the proportion of the  whole  posterior distribution (i.e., 100%HDI) inside the ROPE; so, it can be interpreted like a “classic”  p-value . 
 
  ASPM  only:
 
  β  = -0.69, 89%HDI = [-1.59, 0.25] 
 posterior probability  p ( β <0) = 0.89 (evidence ratio = 7.9),  p ( β =0) = 0.73 (evidence ratio = 2.8) 
 ROPE  4   = [-0.18, 0.18], % HDI inside ROPE = 13.3%;  p  ROPE  = 0.118 
 comparison ‘null’ vs ‘ASPM’: [B> L= W=(71%:29%) K>]: moderate evidence for null against ASPM (BF=3.83), LOO=0.70 [SE=1.43], WAIC=0.92 [SE=1.19], KFOLD=4.48 [SE=3.46]  5   
  
  MCPH1  only:
 
  β  = -0.63, 89%HDI = [-1.66, 0.46] 
 posterior probability  p ( β <0) = 0.83 (evidence ratio = 5),  p ( β =0) = 0.76 (evidence ratio = 3.2) 
 ROPE = [-0.18, 0.18], % HDI inside ROPE = 15.2%;  p  ROPE  = 0.135 
 comparison ‘null’ vs ‘MCPH1’: [B> L= W=(61%:39%) K>]: moderate evidence for null against MCPH1 (BF=3.17), LOO=-0.01 [SE=1.03], WAIC=0.45 [SE=0.77], KFOLD=2.33 [SE=2.31] 
  
  both alleles :
 
 comparison ‘null’ vs ‘both’: [B> L= W=(79%:21%) K>]: moderate evidence for null against both (BF=9.56), LOO=1.30 [SE=1.53], WAIC=1.35 [SE=1.44], KFOLD=4.67 [SE=3.70] 
 interaction:
 
 posterior probability  p (=0) = 0.82 (evidence ratio = 4.5) 
 ROPE = [-0.18, 0.18], % HDI inside ROPE = 21.5%;  p  ROPE  = 0.134 
 comparison ‘no interaction’ vs ‘with interaction’: [B> L> W=(46%:54%) K>]: moderate evidence for no interaction against with interaction (BF=3.06), LOO=0.88 [SE=0.81], WAIC=-0.14 [SE=0.46], KFOLD=6.05 [SE=3.43] 
  
  ASPM  (partial):
 
  β  = -0.61, 89%HDI = [-1.62, 0.32] 
 posterior probability  p ( β <0) = 0.84 (evidence ratio = 5.3),  p ( β =0) = 0.78 (evidence ratio = 3.6) 
 ROPE = [-0.18, 0.18], % HDI inside ROPE = 17.6%;  p  ROPE  = 0.156 
  
  MCPH1  (partial):
 
  β  = -0.46, 89%HDI = [-1.55, 0.78] 
 posterior probability  p ( β <0) = 0.75 (evidence ratio = 2.9),  p ( β =0) = 0.8 (evidence ratio = 4) 
 ROPE = [-0.18, 0.18], % HDI inside ROPE = 19.4%;  p  ROPE  = 0.173 
  
  
 
 
  
 
  Figure 62.    Posterior distributions (with 50% probability mass highlighted) versus 0.0 (the vertical line) for  ASPM -D (left) and  MCPH1 -D (right). 
 
 
 
  
 
  Figure 63.    Conditional effects of  ASPM -D (left) and  MCPH1 -D (right). 
 
 
 
  
 
  Figure 64.    Posterior predictive checks for  ASPM -D (left) and  MCPH1 -D (right). 
 
 
 
  
 
  Figure 65.    Confusion matrices for  ASPM -D (left) and  MCPH1 -D (right). 
 
 
 
 
 
 Mediation and path analysis 
 Here, I try to disentangle the fact that  macroarea  is a very good predictor of  tone1 , but also of the frequency of the two alleles, from any effect that the alleles might have on  tone1 . For this, I conducted mediation analysis and path analysis, where I model the effect of  macroarea  on  tone1  as partially mediated by the two alleles. 
 Please note that there are several technical issues with these approaches: 
 
  for mediation analysis, the method used (as implemented by function  mediate  in package  mediation ): 
 
 cannot deal with a factor with several levels → I focused on the contrast between Africa and the rest of the world; 
 cannot deal with language  family  as random effect → I use “flat” regressions throughout, but I did perform restricted sampling as well as a method to control for  family . 
  
  to adress these issues, I also conducted Bayesian mediation analysis (using  brms ) with logistic regression for the outcome, beta regression for the “derived” allele frequencies, and  family  and  (meta)population  as random effects (the  macroarea  cannot be a random effect as it is the treatment as Africa vs the rest of the world).  
  for path analysis, the method used (as implemented by function  sem  with robust estimators in package  lavaan ): 
 
 cannot deal with binary variables unless they are either converted to numeric (0 vs 1) or ordered (i.e., assume that there is an intrinsic ordering between the two values), affecting both the binary contrast between Africa and the rest of the world (coded as Africa=1, or ordered as “rest of the world” < “Africa”) and  tone1  (coded as Yes=1, or No < Yes); I tested both codings separately; 
 cannot deal with language  family  as random effect, but I did perform restricted sampling as well as a method to control for  family . 
  
 
 
 Mediation analysis 
 
  
 
 
  Figure 66.    Graphical representation of the mediation model for the two alleles considered separately. Blue =  direct effect  of  macroarea  on  tone1 ; red =  indirect effect  mediatated by the alleles. 
 
 
 
  (g)lm  
 
 All data 
 For  ASPM -D: 
 
   total effect  (TE) of being in Africa on tone: 0.49 (0.33, 0.63),  p =0, decomposed into:  
   average direct effect  (ADE): 0.27 (0.08, 0.47),  p =0.008, and  
   average indirect effect  (ACME) mediated by  ASPM -D: 0.22 (0.11, 0.34),  p =0, mediating 44.9% (19.1%, 79.5%),  p =0 of the effect, resulting from: 
 
 effect of being in Africa on  ASPM -D: -1.25 ±0.16,  p =7.7e-13, and 
 effect of  ASPM -D on tone: -0.90 ±0.24,  p =0.00015. 
  
 
 For  MCPH1 -D: 
 
   TE : 0.50 (0.34, 0.65),  p =0, decomposed into:  
   ADE : 0.55 (0.19, 0.75),  p =0.002, and  
   ACME : -0.05 (-0.22, 0.25),  p =0.49, mediating -14.7% (-51.3%, 56.8%),  p =0.49 of the effect, resulting from: 
 
 effect of being in Africa on  MCPH1 -D: -2.19 ±0.09,  p =9.9e-59, and 
 effect of  MCPH1 -D on tone: 0.20 ±0.38,  p =0.6. 
  
 
 
 
 Restricted sampling 
 
 
 
  Figure 67.    Mediation analysis for 1000 restricted samples (i.e., picking one random language per family). The leftmost panels show the distribution of point estimates of the Total Effect (TE), the Direct Effect (ADE) and the Indirect Effect (ACME) for  ASPM -D and  MCPH1 -D; the middle panels show the distribution of the  p -values for the same effects, while the rightmost panels show the distribution of the regression slopes ( β ) for the two alleles, top: for the regression of the allele frequency on within vs outside Africa, and bottom: for the regression of tone on the allele while controlling for within vs outside Africa. The black vertical lines show: 0.0 (solid), 0.05 (dashed) and 0.10 (dotted). 
 
 
 For  ASPM -D: 
 
   TE : mean = 0.38, median = 0.38; 44.5% significant at  α -level 0.05 and 72.8% significant at  α -level 0.10; 100.0% > 0.0; one-sample one-sided t-test vs 0:  t (999) = 134.2,  p  = 0;  
   ADE : mean = 0.28, median = 0.28; 8.2% significant at  α -level 0.05 and 29.6% significant at  α -level 0.10; 99.6% > 0.0; one-sample one-sided t-test vs 0:  t (999) = 87.2,  p  = 0;  
   ACME : mean = 0.094, median = 0.091; 3.6% significant at  α -level 0.05 and 20.1% significant at  α -level 0.10; 99.5% > 0.0; one-sample one-sided t-test vs 0:  t (999) = 61.4,  p  = 0;  
   β(Africa → allele) : mean = -0.86, median = -0.87; 79.8% significant at  α -level 0.05 and 96.0% significant at  α -level 0.10; 100.0% < 0.0; one-sample one-sided t-test vs 0:  t (999) = -211.9,  p  = 0;  
   β(allele → tone | Africa) : mean = -0.61, median = -0.6; 10.2% significant at  α -level 0.05 and 27.7% significant at  α -level 0.10; 99.1% < 0.0; one-sample one-sided t-test vs 0:  t (999) = -60.0,  p  = 0.  
 
 For  MCPH1 -D: 
 
   TE : mean = 0.38, median = 0.39; 44.3% significant at  α -level 0.05 and 72.6% significant at  α -level 0.10; 100.0% > 0.0; one-sample one-sided t-test vs 0:  t (999) = 133.1,  p  = 0;  
   ADE : mean = 0.41, median = 0.44; 6.0% significant at  α -level 0.05 and 18.9% significant at  α -level 0.10; 96.9% > 0.0; one-sample one-sided t-test vs 0:  t (999) = 74.0,  p  = 0;  
   ACME : mean = -0.029, median = -0.052; 0.1% significant at  α -level 0.05 and 1.4% significant at  α -level 0.10; 35.7% > 0.0; one-sample one-sided t-test vs 0:  t (999) = -6.2,  p  = 1;  
   β(Africa → allele) : mean = -2.5, median = -2.5; 100.0% significant at  α -level 0.05 and 100.0% significant at  α -level 0.10; 100.0% < 0.0; one-sample one-sided t-test vs 0:  t (999) = -884.5,  p  = 0;  
   β(allele → tone | Africa) : mean = 0.42, median = 0.42; 0.2% significant at  α -level 0.05 and 1.5% significant at  α -level 0.10; 25.5% < 0.0; one-sample one-sided t-test vs 0:  t (999) = 21.4,  p  = 1.  
 
 Given the low sample size  N  = 35 unique families, relatively few effect sizes are big enough to be significant for each individual analysis; however, there are many more significant ACMEs for  ASPM -D than for  MCPH1 -D: 10.2% vs 0.2% (51.0 times) for  α -level 0.05, and 27.7% vs 1.5% (18.5 times) for  α -level 0.10. 
 
 
 
  brms  
 
  
 
 
  Figure 68.    Graphical representation of the Bayesian mediation analysis for  ASPM -D showing the means of the effects and the actual partial regression coefficients, with their 89% HDIs and p-ROPEs. The colors reflect the sign of the mean estimate (blue=negative, red=positive, gray=(p-ROPE >= 0.05)); solid=(0 not in the HDI), dashed=(0 is in the HDI). 
 
 
 
  
 
 
  Figure 69.    Graphical representation of the Bayesian mediation analysis for  MCPH1 -D showing the means of the effects and the actual partial regression coefficients, with their 89% HDIs and p-ROPEs. The colors reflect the sign of the mean estimate (blue=negative, red=positive, gray=(p-ROPE >= 0.05)); solid=(0 not in the HDI), dashed=(0 is in the HDI). 
 
 
 
  
 
 
  Figure 70.    Graphical representation of the Bayesian mediation analysis for both  ASPM -D and  MCPH1 -D showing the means of the effects and the actual partial regression coefficients, with their 89% HDIs and p-ROPEs. The colors reflect the sign of the mean estimate (blue=negative, red=positive, gray=(p-ROPE >= 0.05)); solid=(0 not in the HDI), dashed=(0 is in the HDI). 
 
 
 
 
 
 Path analysis 
 
 All data 
 With Africa and  tone1  coded numerically, the model fits the data very well  6   ( χ  2 (1)=0.22,  p =0.64; CFI=1.00, TLI=1.01, NNFI=1.01 and RFI=1.00): 
 
  
 
 
  Figure 71.    Path analysis model with standardised coefficients and significance stars.  tone1  and  macroarea  (Africa vs non-Africa) are coded as numeric binary ( tone_bin_num  with Yes=1 and  Africa_num  with in Africa=1);  ASPM_z  is  ASPM -D and  MCPH1_z  is  MCPH1 -D. 
 
 
  ## lavaan 0.6-8 ended normally after 25 iterations
## 
##   Estimator                                         ML
##   Optimization method                           NLMINB
##   Number of model parameters                         8
##                                                       
##   Number of observations                           181
##                                                       
## Model Test User Model:
##                                                       
##   Test statistic                                 0.225
##   Degrees of freedom                                 1
##   P-value (Chi-square)                           0.635
## 
## Model Test Baseline Model:
## 
##   Test statistic                               371.522
##   Degrees of freedom                                 6
##   P-value                                        0.000
## 
## User Model versus Baseline Model:
## 
##   Comparative Fit Index (CFI)                    1.000
##   Tucker-Lewis Index (TLI)                       1.013
## 
## Loglikelihood and Information Criteria:
## 
##   Loglikelihood user model (H0)               -448.206
##   Loglikelihood unrestricted model (H1)       -448.094
##                                                       
##   Akaike (AIC)                                 912.413
##   Bayesian (BIC)                               938.000
##   Sample-size adjusted Bayesian (BIC)          912.664
## 
## Root Mean Square Error of Approximation:
## 
##   RMSEA                                          0.000
##   90 Percent confidence interval - lower         0.000
##   90 Percent confidence interval - upper         0.154
##   P-value RMSEA <= 0.05                          0.704
## 
## Standardized Root Mean Square Residual:
## 
##   SRMR                                           0.005
## 
## Parameter Estimates:
## 
##   Standard errors                           Robust.sem
##   Information                                 Expected
##   Information saturated (h1) model          Structured
## 
## Regressions:
##                    Estimate  Std.Err  z-value  P(>|z|) ci.lower ci.upper
##   tone_bin_num ~                                                        
##     Africa_num        0.390    0.166    2.351    0.019    0.065    0.716
##     ASPM_z           -0.144    0.035   -4.163    0.000   -0.212   -0.076
##     MCPH1_z           0.025    0.061    0.410    0.682   -0.095    0.145
##   ASPM_z ~                                                              
##     Africa_num       -1.249    0.111  -11.254    0.000   -1.467   -1.032
##   MCPH1_z ~                                                             
##     Africa_num       -2.190    0.081  -27.039    0.000   -2.349   -2.031
##    Std.lv  Std.all
##                   
##     0.390    0.330
##    -0.144   -0.305
##     0.025    0.053
##                   
##    -1.249   -0.500
##                   
##    -2.190   -0.877
## 
## Variances:
##                    Estimate  Std.Err  z-value  P(>|z|) ci.lower ci.upper
##    .tone_bin_num      0.165    0.015   10.844    0.000    0.135    0.195
##    .ASPM_z            0.746    0.074   10.144    0.000    0.602    0.890
##    .MCPH1_z           0.230    0.039    5.899    0.000    0.154    0.307
##    Std.lv  Std.all
##     0.165    0.740
##     0.746    0.750
##     0.230    0.232
## 
## R-Square:
##                    Estimate
##     tone_bin_num      0.260
##     ASPM_z            0.250
##     MCPH1_z           0.768  
 Likewise, with Africa and  tone1  coded as ordered binary factors, the model also fits the data very well ( χ  2 (1)=0.57,  p =0.45; CFI=1.00, TLI=1.07, NNFI=1.07 and RFI=0.92): 
 
  
 
 
  Figure 72.    Path analysis model with standardised coefficients and significance stars.  tone1  and  macroarea  (Africa vs non-Africa) are coded as ordered binary factors ( tone_bin_ord  with No < Yes, and  Africa_ord  with outside Africa < in Africa);  ASPM_z  is  ASPM -D and  MCPH1_z  is  MCPH1 -D. 
 
 
 
 
 Restricted sampling 
 Here I use only the numerical coding. 
 
 
 
  Figure 73.    Path analysis for 1000 restricted samples (i.e., picking one random language per family). The leftmost row of two plots shows the coefficient estimates and the  p -values, respectively, for the five paths in the model (see the path plots above). The rightmost plot shows the various fit indices. The black horiontal lines show: 0.0 (solid), 0.05 (dashed) and 1.0 (dotted). 
 
 
 
  models fits: 
 
 94.7% of the  p -values are not significant 
 mean(CFI) = 0.99, median(CFI) = 1, sd(CFI) = 0.01, IQR(CFI) = 0.02 
 mean(TLI) = 0.97, median(TLI) = 0.99, sd(TLI) = 0.1, IQR(TLI) = 0.16 
 mean(NNFI) = 0.97, median(NNFI) = 0.99, sd(NNFI) = 0.1, IQR(NNFI) = 0.16 
 mean(RFI) = 0.9, median(RFI) = 0.92, sd(RFI) = 0.09, IQR(RFI) = 0.14 
  
  Africa →  ASPM -D: mean = -0.87, median = -0.89, sd = 0.12, IQR = 0.17, 100.0% < 0; 98.7% significant at  α -level 0.05; one-sample one-sided  t -test vs 0:  t (999) = -2.2e+02,  p  = 0;  
  Africa →  MCPH1 -D: mean = -2.5, median = -2.5, sd = 0.086, IQR = 0.12, 100.0% < 0; 100.0% significant at  α -level 0.05; one-sample one-sided  t -test vs 0:  t (999) = -9e+02,  p  = 0;  
  Africa →  tone1 : mean = 0.43, median = 0.44, sd = 0.32, IQR = 0.46, 89.5% > 0; 12.8% significant at  α -level 0.05; one-sample one-sided  t -test vs 0:  t (999) = 41,  p  = 3.2e-219;  
   ASPM -D →  tone1 : mean = -0.11, median = -0.11, sd = 0.058, IQR = 0.089, 98.6% < 0; 36.2% significant at  α -level 0.05; one-sample one-sided  t -test vs 0:  t (999) = -62,  p  = 0;  
   MCPH1 -D →  tone1 : mean = 0.041, median = 0.041, as = 0.11, IQR = 0.17, 36.7% < 0; 0.9% significant at  α -level 0.05; one-sample one-sided  t -test vs 0:  t (999) = 11,  p  = 1.  
 
 
 
 
 
 Machine Learning techniques 
 Here I apply various “machine learning” techniques to explore how well the  macroarea  and the two alleles predict  tone1 . For these techniques, in general I: 
 
 fit the model to the  full  data and estimate how well these modes fit, but also 
 repeatedly split the data into a  training  set the complementary  test  set; the first usually contains a random subset of 80% of the data and is used to fit the model, while the second, containing the remaining 20% of the data, is used to check how well the model generalizes to new data. 
 
 Thus, these techniques can: 
 
 quantify the  amount of information  about tone contained by macroarea and the alleles, 
 but also give an estimate of the  relative importance  of these variables as predictors. 
 
 
 Decision trees 
 
 Including macroarea 
 Using the frequency of the two alleles and the  macroarea  as predictors, the fit to the data is: accuracy = 77.3%, sensitivity = 71.7%, specificity = 79.3%, precision = 54.1%, and recall = 71.7%. 
 
 
 
  Figure 74.    Decision tree on the full data using the two alleles and  macroarea .  ASPM.D  =  ASPM -D. 
 
 
 On the 100 training/testing sets, the fit is: accuracy = 77.1% ±6.6%, sensitivity = 71.6% ±15.2%, specificity = 79.3% ±6.8%, precision = 52.9% ±12.4%, recall = 71.6% ±15.2%. 
 
 
 
  Figure 75.    The success of generalising to the testing sets from the training sets (yellow boxplots) compared to the success on the full data (red segments). 
 
 
 
 
 Excluding macroarea 
 When using the frequency of the two alleles only as predictors, the fit to the data is: accuracy = 75.1%, sensitivity = 75.0%, specificity = 75.2%, precision = 39.3%, and recall = 75.0%: 
 
 
 
  Figure 76.    Decision tree on the full data using the two alleles only.  ASPM.D  =  ASPM -D,  MCPH1.D  =  MCPH1 -D. 
 
 
 On the 100 training/testing sets,the fit is: accuracy = 70.1% ±7.6%, sensitivity = 61.3% ±19.1%, specificity = 76.2% ±8.7%, precision = 44.0% ±23.0%, recall = 61.3% ±19.1%. 
 
 
 
  Figure 77.    The success of generalising to the testing sets from the training sets (yellow boxplots) compared to the success on the full data (red segments). 
 
 
 
 
 
 Random forests 
 I use two methods: random forests as implemented by  randomForest()  in package  randomForest , and conditional random forests as implemented by  cforest()  in package  partykit . As (conditional) random forests do internal bootstrapping, there is no need for the explicit training/testing set repeated refitting. 
 
 Including macroarea 
 When using the frequency of the two alleles and the  macroarea  as predictors, the models fit to the full data is: 
 
 random forests: accuracy = 77.7% ±0.8%, sensitivity = 68.9% ±1.9%, specificity = 81.6% ±0.4%, precision = 62.0% ±1.0%, recall = 68.9% ±1.9%, 
 conditional random forests: accuracy = 84.3% ±0.7%, sensitivity = 78.8% ±0.5%, specificity = 86.8% ±0.9%, precision = 73.0% ±2.0%, recall = 78.8% ±0.5%. 
 
 
 
 
  Figure 78.    The success of the two random forest methods on the full data. 
 
 
 
 
 
  Figure 79.    Variable importance using three methods: mean decrease in accuracy, mean decrease of the Gini coeficient, and unconditional importance.  ASPM_freq_wavg  =  ASPM -D,  MCPH1_freq_wavg  =  MCPH1 -D. 
 
 
 
 
 Excluding macroarea 
 When using the frequency of the two alleles only, the models fit the full as: 
 
 random forests: accuracy = 70.7% ±1.0%, sensitivity = 56.3% ±1.4%, specificity = 78.6% ±0.8%, precision = 59.0% ±1.8%, recall = 56.3% ±1.4%, 
 conditional random forests: accuracy = 82.1% ±0.5%, sensitivity = 81.8% ±0.7%, specificity = 82.3% ±0.6%, precision = 60.5% ±1.6%, recall = 81.8% ±0.7%. 
 
 
 
 
  Figure 80.    The success of the two random forest methods on the full data. 
 
 
 
 
 
  Figure 81.    Variable importance using three methods: mean decrease in accuracy, mean decrease of the Gini coeficient, and unconditional importance.  ASPM_freq_wavg  =  ASPM -D,  MCPH1_freq_wavg  =  MCPH1 -D. 
 
 
 
 
 
 
 Diachronic analyses 
 Here I try various analyses that explicitly take into account the diachronic nature of the processes. 
 The families with more than 2 tips are: 
 
 
 
  Figure 82.    Phylogenies with  tone1  (0=“No,” 1=“Yes,”  ASPM -D and  MCHP1 -D for the families with at least 2 languages. 
 
 
 It can be seen that, unfortunately, there are very few families with more than 2 languages with data (17), and even for those with relatively many languages, there is very little variation in  tone1  and in the frequencies of the two “derived” alleles. Unfortunately, combined with the issues concerning branch length for language family trees, this precludes the estimation of correlated evolution or phylogenetic regression methods. 
 
 
 
  tone2  (is there  complex  tone?) 
 I kept only the entries with non-missing data for the  tone2 ,  ASPM -D and  MCPH1 -D, and if there are more than one possible languages or allele frequencies for a given sample, I only kept those entries that have different tone or allele data. The resulting dataset has 180 observations, distributed among 118 unique Glottolg codes in 35 families (ranging from a minimum of 1 language per family to a maximum of 47, with a mean 5.1 and median 2 languages per family) and 4 macroareas. 
 There are 156:121:118 unique samples:(meta)populations:languages retained, dropping 19:8:203 = {FINRISK, GenDan, GenNed5, gnomAD_asj, gnomAD_bgr, gnomAD_est, gnomAD_fin, gnomAD_jpn, gnomAD_kor, gnomAD_swe, gnomADexomes_AshkenaziJewish, gnomADgenomes_AshkenaziJewish, KRGDB, Qatari, SA001471N, SA001477T, SA001487U, SA001491P, SA001681Q} : {Bulgarian, Burunge, Dutch, Hazara, Mozabite, Oroqen, Qatari, Xibe} : {adze1240, ajie1238, amar1272, ambu1247, anei1239, apma1241, arak1252, arib1241, arop1243, aros1241, aulu1238, awtu1239, ayiw1239, baba1268, bahi1254, bann1247, bign1238, bili1260, boik1241, bulg1262, buru1320, caro1242, cham1313, chek1238, chuu1238, dehu1237, dumb1241, dutc1256, east2443, east2447, efee1239, fiji1243, futu1245, gapa1238, geez1241, gela1263, gilb1244, gulf1241, guma1254, gyel1242, hali1244, hang1263, hano1246, haza1239, hmon1264, hoav1238, iaai1238, iatm1242, idak1243, idun1242, iris1253, iwam1256, juho1239, kaia1245, kair1263, kamb1297, kapi1249, kara1486, kaul1240, kela1255, kele1258, kili1267, kire1240, koko1269, kosr1238, kuan1247, kuan1248, kuma1276, kung1261, kwai1243, kwam1251, kwam1252, kwom1262, labu1248, lala1268, lame1260, lauu1247, lena1238, lewo1242, long1395, loni1238, lonw1238, louu1245, lusi1240, maee1241, mais1250, male1289, malo1243, mana1295, mana1298, maor1246, mars1254, masa1299, matu1261, mbal1255, mbul1263, mehe1243, meke1243, mele1250, mina1269, ming1252, moch1256, moki1238, moks1248, mono1273, motl1237, motu1246, mudu1242, muri1260, muso1238, muss1246, muyu1244, naka1262, nali1244, nami1256, natu1246, naur1243, ndon1254, neha1247, neng1238, ngan1300, niua1240, niue1239, nort2646, nort2836, nort2845, nuku1260, onto1237, oroq1238, paam1238, pate1247, patp1243, pile1238, ping1243, pohn1238, port1285, pulu1242, qima1242, raoo1244, rapa1244, renn1242, rotu1241, rovi1238, russ1264, saaa1240, saam1283, saka1289, sali1295, samo1305, sapo1253, scot1243, siar1238, siee1239, sina1266, sioo1240, sobe1238, sons1242, sout2642, sout2679, sout2807, sout2856, sout2866, sout2869, stan1318, sude1239, surs1246, tahi1242, tain1252, taki1248, tawa1275, tean1237, teop1238, tiga1245, tigr1271, tiri1258, toab1237, toba1266, toke1240, tong1325, tswa1253, tuam1242, tuml1238, tumz1238, tung1290, tuva1244, ulit1238, urav1235, urip1239, vinm1237, waim1251, wall1257, wata1253, west2500, west2519, woga1249, wole1240, xamt1239, xara1244, xibe1242, yabe1254, yess1239, yima1243, zulu1248}. 
 
 
 
 
 
 
 
 
 
 
 
   
 Africa 
 Eurasia 
 America 
 Papunesia 
 Sum 
 
 
 
 
  No  
 28 
 105 
 9 
 9 
 151 
 
 
  Yes  
 9 
 18 
 1 
 1 
 29 
 
 
  Sum  
 37 
 123 
 10 
 10 
 180 
 
 
 
 
 
 
  Figure 83.    Distribution of  tone2 . 
 
 
 
 
 
  Figure 84.    Map of  tone2 . 
 
 
 
 
 
  Figure 85.    Relationship between  tone2 ,  ASPM -D and  MCPH1 -D. 
 
 
 Please note that the distribution of this variable is very skewed, so the results might not be very solid… 
 
 Regressions 
 
  glmer  
 
 All data 
 
  null model : R 2  = 0.0%, ICC = 95.6% 
  macroarea : R 2  = 2.0%,  p  macroarea/null  = 0.5 
  ASPM :
 
 by itself: R 2  = 1.3%,  β  = -0.87 ± 0.69,  p  ASPM/null  = 0.19 
 quadratic: R 2  = 36.6%,  β ASPM2   = -3.46 ± 2.31,  p  ASPM2/ASPM  = 0.049 
 with  macroarea : R 2  = 2.6%,  p  macroarea/ASPM  = 0.8,  p  ASPM/macroarea  = 0.55 
  
  MCPH1 :
 
 by itself: R 2  = 1.5%,  β  = -1.01 ± 0.73,  p  MCPH1/null  = 0.16 
 quadratic: R 2  = 3.0%,  β MCPH12   = -1.17 ± 0.81,  p  MCPH12/MCPH1  = 0.22 
 with  macroarea : R 2  = 2.4%,  p  macroarea/MCPH1  = 0.89,  p  MCPH1/macroarea  = 0.62 
  
  both alleles  (no  macroarea ):
 
  ASPM  +  MCPH1 : R 2  = 2.0%,  β  ASPM  = -0.56 ± 0.78,  p  ASPM/MCPH1  = 0.47,  β  MCPH1  = -0.68 ± 0.81,  p  MCPH1/ASPM  = 0.39,  p  ASPM+MCPH1/null  = 0.29, 
 interaction: R 2  = 1.5%,  p  ASPM:MCPH1/ASPM+MCPH1  = 0.76 
  
 
 
 
 Randomization 
 
 Regressions with randomizations for  tone2 . 
 
 
 Permute within 
 Macroarea 
 Permute 
 AIC 
 Signif. 
  p   ASPM -D  
  β   ASPM -D  
  p   MCPH1 -D  
  β   MCPH1 -D  
 
 
 
 
 unrestricted 
 none 
 tone 
 0% 
 4% 
 4% 
 1% 
 6% 
 0% 
 
 
 unrestricted 
 none 
 alleles-together 
 31% 
 7% 
 6% 
 10% 
 6% 
 6% 
 
 
 unrestricted 
 none 
 alleles-independent 
 32% 
 7% 
 7% 
 9% 
 6% 
 4% 
 
 
 unrestricted 
 fixef 
 tone 
 0% 
 6% 
 6% 
 6% 
 7% 
 23% 
 
 
 unrestricted 
 fixef 
 alleles-together 
 84% 
 7% 
 7% 
 17% 
 7% 
 25% 
 
 
 unrestricted 
 fixef 
 alleles-independent 
 83% 
 9% 
 8% 
 16% 
 7% 
 21% 
 
 
 macroareas 
 none 
 tone 
 0% 
 4% 
 3% 
 1% 
 10% 
 1% 
 
 
 macroareas 
 none 
 alleles-together 
 40% 
 7% 
 5% 
 21% 
 6% 
 42% 
 
 
 macroareas 
 none 
 alleles-independent 
 44% 
 8% 
 6% 
 28% 
 10% 
 45% 
 
 
 macroareas 
 fixef 
 tone 
 0% 
 4% 
 4% 
 7% 
 4% 
 22% 
 
 
 macroareas 
 fixef 
 alleles-together 
 80% 
 8% 
 8% 
 28% 
 7% 
 38% 
 
 
 macroareas 
 fixef 
 alleles-independent 
 80% 
 9% 
 8% 
 25% 
 8% 
 37% 
 
 
 families 
 none 
 tone 
 31% 
 4% 
 4% 
 28% 
 1% 
 16% 
 
 
 families 
 none 
 alleles-together 
 20% 
 4% 
 5% 
 29% 
 1% 
 15% 
 
 
 families 
 none 
 alleles-independent 
 24% 
 4% 
 6% 
 25% 
 3% 
 22% 
 
 
 families 
 fixef 
 tone 
 45% 
 8% 
 9% 
 54% 
 4% 
 43% 
 
 
 families 
 fixef 
 alleles-together 
 80% 
 4% 
 6% 
 43% 
 3% 
 18% 
 
 
 families 
 fixef 
 alleles-independent 
 80% 
 5% 
 7% 
 34% 
 4% 
 24% 
 
 
 
 
 
 
Regressions on 1000 permuted data. Each plot shows the original result (vertical dashed black line) and the distribution of the permutations for the three possible things to be permuted (colored curves) for each combination of permutation constraints (horizontal panels) and control for  macroarea  (vertical panels) in terms of the effect size  β ;  ASPM -D is on the left and  MCPH1 -D on the right. The vertical dotted black thin line is at 0.0.
 
 
 
 
 Restricted sampling 
 
 
 
  Figure 86.    Results for 1000 restricted samplings. For  ASPM -D (left): 99.6% of βs are negative when regressing tone on  ASPM  alone (one-sided  t -test < 0:  t (999) = -77.3, mean = -0.61,  p  = 0), 99.8%, when controlling for the macroarea ( t (999) = -75.6, mean = -0.96,  p  = 0), and 99.9% when controlling for both macroarea and  MCPH1  ( t (999) = -70.9, mean = -1.08,  p  = 0). For  MCPH1 -D (right): 72.9% of βs are negative when regressing tone on  MCPH1  alone (one-sided  t -test < 0:  t (999) = -21.7, mean = -0.16,  p  = 7.8e-86), 42.9% when controlling for the macroarea ( t (999) = 9.6, mean = 0.30,  p  = 1), and 35.1% when controlling for both macroarea and  ASPM  ( t (999) = 17.1, mean = 0.67,  p  = 1). 
 
 
 
 
 
  brms  
 
  ASPM  only:
 
  β  = -1.27, 89%HDI = [-2.73, 0.17] 
 posterior probability  p ( β <0) = 0.93 (evidence ratio = 14),  p ( β =0) = 0.57 (evidence ratio = 1.4) 
 ROPE = [-0.18, 0.18], % HDI inside ROPE = 6.1%;  p  ROPE  = 0.055 
 comparison ‘null’ vs ‘ASPM’: [B= L> W=(53%:47%) K=]: anecdotal evidence for null against ASPM (BF=1.46), LOO=1.84 [SE=1.14], WAIC=0.10 [SE=1.16], KFOLD=-0.80 [SE=2.10] 
  
  MCPH1  only:
 
  β  = -0.91, 89%HDI = [-2.38, 0.58] 
 posterior probability  p ( β <0) = 0.85 (evidence ratio = 5.5),  p ( β =0) = 0.7 (evidence ratio = 2.4) 
 ROPE = [-0.18, 0.18], % HDI inside ROPE = 10.9%;  p  ROPE  = 0.097 
 comparison ‘null’ vs ‘MCPH1’: [B= L= W=(41%:59%) K<]: anecdotal evidence for null against MCPH1 (BF=1.63), LOO=0.25 [SE=0.64], WAIC=-0.36 [SE=0.73], KFOLD=-2.43 [SE=1.67] 
  
  both alleles :
 
 comparison ‘null’ vs ‘both’: [B> L= W=(30%:70%) K=]: moderate evidence for null against both (BF=3.66), LOO=0.13 [SE=1.39], WAIC=-0.87 [SE=1.44], KFOLD=-1.33 [SE=2.11] 
 interaction:
 
 posterior probability  p (=0) = 0.75 (evidence ratio = 3) 
 ROPE = [-0.18, 0.18], % HDI inside ROPE = 15.1%;  p  ROPE  = 0.134 
 comparison ‘no interaction’ vs ‘with interaction’: [B> L> W=(57%:43%) K>]: moderate evidence for no interaction against with interaction (BF=3.65), LOO=1.16 [SE=1.00], WAIC=0.29 [SE=0.39], KFOLD=2.31 [SE=1.26] 
  
  ASPM  (partial):
 
  β  = -1.13, 89%HDI = [-2.71, 0.60] 
 posterior probability  p ( β <0) = 0.87 (evidence ratio = 6.8),  p ( β =0) = 0.66 (evidence ratio = 1.9) 
 ROPE = [-0.18, 0.18], % HDI inside ROPE = 9%;  p  ROPE  = 0.08 
  
  MCPH1  (partial):
 
  β  = -0.58, 89%HDI = [-2.21, 0.99] 
 posterior probability  p ( β <0) = 0.73 (evidence ratio = 2.7),  p ( β =0) = 0.75 (evidence ratio = 3) 
 ROPE = [-0.18, 0.18], % HDI inside ROPE = 13.7%;  p  ROPE  = 0.122 
  
  
 
 
  
 
  Figure 87.    Posterior distributions (with 50% probability mass highlighted) versus 0.0 (the vertical line) for  ASPM -D (left) and  MCPH1 -D (right). 
 
 
 
  
 
  Figure 88.    Conditional effects of  ASPM -D (left) and  MCPH1 -D (right). 
 
 
 
  
 
  Figure 89.    Posterior predictive checks for  ASPM -D (left) and  MCPH1 -D (right). 
 
 
 
  
 
  Figure 90.    Confusion matrices for  ASPM -D (left) and  MCPH1 -D (right). 
 
 
 
 
 
 Mediation and path analysis 
 
 Mediation analysis 
 
  (g)lm  
 
 All data 
 For  ASPM -D: 
 
   total effect  (TE) of being in Africa on tone: 0.14 (-0.01, 0.30),  p =0.078, decomposed into:  
   average direct effect  (ADE): -0.05 (-0.20, 0.10),  p =0.43, and  
   average indirect effect  (ACME) mediated by  ASPM -D: 0.19 (0.08, 0.31),  p =0.004, mediating 133.8% (-419.6%, 802.0%),  p =0.082 of the effect, resulting from: 
 
 effect of being in Africa on  ASPM -D: -1.34 ±0.16,  p =3.7e-15, and 
 effect of  ASPM -D on tone: -1.03 ±0.31,  p =0.0011. 
  
 
 For  MCPH1 -D: 
 
   TE : 0.11 (-0.02, 0.27),  p =0.12, decomposed into:  
   ADE : 0.12 (-0.21, 0.45),  p =0.47, and  
   ACME : -0.01 (-0.29, 0.29),  p =0.9, mediating -11.4% (-804.5%, 1112.4%),  p =0.93 of the effect, resulting from: 
 
 effect of being in Africa on  MCPH1 -D: -2.19 ±0.09,  p =1.2e-61, and 
 effect of  MCPH1 -D on tone: 0.03 ±0.45,  p =0.94. 
  
 
 
 
 Restricted sampling 
 
 
 
  Figure 91.    Mediation analysis for 1000 restricted samples (i.e., picking one random language per family). The leftmost panels show the distribution of point estimates of the Total Effect (TE), the Direct Effect (ADE) and the Indirect Effect (ACME) for  ASPM  and  MCPH1 ; the middle panels show the distribution of the  p -values for the same effects, while the rightmost panels show the distribution of the regression slopes ( β ) for the two alleles, top: for the regression of the allele frequency on within vs outside Africa, and bottom: for the regression of tone on the allele while controlling for within vs outside Africa. The black vertical lines show: 0.0 (solid), 0.05 (dashed) and 0.10 (dotted). 
 
 
 For  ASPM -D: 
 
   TE : mean = 0.11, median = 0.12; 0.4% significant at  α -level 0.05 and 2.3% significant at  α -level 0.10; 89.6% > 0.0; one-sample one-sided t-test vs 0:  t (999) = 39.7,  p  = 5.9e-208;  
   ADE : mean = 0.04, median = 0.039; 0.0% significant at  α -level 0.05 and 0.0% significant at  α -level 0.10; 67.3% > 0.0; one-sample one-sided t-test vs 0:  t (999) = 16.0,  p  = 7.9e-52;  
   ACME : mean = 0.072, median = 0.07; 0.0% significant at  α -level 0.05 and 0.1% significant at  α -level 0.10; 100.0% > 0.0; one-sample one-sided t-test vs 0:  t (999) = 78.5,  p  = 0;  
   β(Africa → allele) : mean = -0.88, median = -0.89; 88.6% significant at  α -level 0.05 and 98.7% significant at  α -level 0.10; 100.0% < 0.0; one-sample one-sided t-test vs 0:  t (999) = -249.8,  p  = 0;  
   β(allele → tone | Africa) : mean = -0.58, median = -0.57; 0.0% significant at  α -level 0.05 and 0.4% significant at  α -level 0.10; 100.0% < 0.0; one-sample one-sided t-test vs 0:  t (999) = -79.8,  p  = 0.  
 
 For  MCPH1 -D: 
 
   TE : mean = 0.11, median = 0.11; 0.2% significant at  α -level 0.05 and 2.3% significant at  α -level 0.10; 82.1% > 0.0; one-sample one-sided t-test vs 0:  t (999) = 37.1,  p  = 1.3e-190;  
   ADE : mean = 0.13, median = 0.14; 0.0% significant at  α -level 0.05 and 0.7% significant at  α -level 0.10; 75.0% > 0.0; one-sample one-sided t-test vs 0:  t (999) = 24.8,  p  = 2.3e-106;  
   ACME : mean = -0.028, median = -0.034; 0.0% significant at  α -level 0.05 and 0.3% significant at  α -level 0.10; 43.0% > 0.0; one-sample one-sided t-test vs 0:  t (999) = -5.4,  p  = 1;  
   β(Africa → allele) : mean = -2.4, median = -2.4; 100.0% significant at  α -level 0.05 and 100.0% significant at  α -level 0.10; 100.0% < 0.0; one-sample one-sided t-test vs 0:  t (999) = -927.9,  p  = 0;  
   β(allele → tone | Africa) : mean = 0.26, median = 0.21; 0.0% significant at  α -level 0.05 and 0.1% significant at  α -level 0.10; 38.0% < 0.0; one-sample one-sided t-test vs 0:  t (999) = 11.3,  p  = 1.  
 
 
 
 
  brms  
 
  
 
 
  Figure 92.    Graphical representation of the Bayesian mediation analysis for  ASPM -D showing the means of the effects and the actual partial regression coefficients, with their 89% HDIs and p-ROPEs. The colors reflect the sign of the mean estimate (blue=negative, red=positive, gray=(p-ROPE >= 0.05)); solid=(0 not in the HDI), dashed=(0 is in the HDI). 
 
 
 
  
 
 
  Figure 93.    Graphical representation of the Bayesian mediation analysis for  MCPH1 -D showing the means of the effects and the actual partial regression coefficients, with their 89% HDIs and p-ROPEs. The colors reflect the sign of the mean estimate (blue=negative, red=positive, gray=(p-ROPE >= 0.05)); solid=(0 not in the HDI), dashed=(0 is in the HDI). 
 
 
 
  
 
 
  Figure 94.    Graphical representation of the Bayesian mediation analysis for both  ASPM -D and  MCPH1 -D showing the means of the effects and the actual partial regression coefficients, with their 89% HDIs and p-ROPEs. The colors reflect the sign of the mean estimate (blue=negative, red=positive, gray=(p-ROPE >= 0.05)); solid=(0 not in the HDI), dashed=(0 is in the HDI). 
 
 
 
 
 
 Path analysis 
 
 All data 
 Coding Africa and  tone2  numerically, the model fit is:  χ  2 (1)=0.36,  p =0.55; CFI=1.00, TLI=1.01, NNFI=1.01 and RFI=0.99. 
 
  
 
 
  Figure 95.    Path analysis model with standardised coefficients and significance stars. Here, we coded tone and macroarea (Africa vs non-Africa) as numeric binary ( tone_complex_num  with Yes=1 and  Africa_num  with in Africa=1);  ASPM_z  is  ASPM -D and  MCPH1_z  is  MCPH1 -D. 
 
 
  ## lavaan 0.6-8 ended normally after 25 iterations
## 
##   Estimator                                         ML
##   Optimization method                           NLMINB
##   Number of model parameters                         8
##                                                       
##   Number of observations                           180
##                                                       
## Model Test User Model:
##                                                       
##   Test statistic                                 0.361
##   Degrees of freedom                                 1
##   P-value (Chi-square)                           0.548
## 
## Model Test Baseline Model:
## 
##   Test statistic                               354.897
##   Degrees of freedom                                 6
##   P-value                                        0.000
## 
## User Model versus Baseline Model:
## 
##   Comparative Fit Index (CFI)                    1.000
##   Tucker-Lewis Index (TLI)                       1.011
## 
## Loglikelihood and Information Criteria:
## 
##   Loglikelihood user model (H0)               -407.836
##   Loglikelihood unrestricted model (H1)       -407.655
##                                                       
##   Akaike (AIC)                                 831.671
##   Bayesian (BIC)                               857.215
##   Sample-size adjusted Bayesian (BIC)          831.879
## 
## Root Mean Square Error of Approximation:
## 
##   RMSEA                                          0.000
##   90 Percent confidence interval - lower         0.000
##   90 Percent confidence interval - upper         0.166
##   P-value RMSEA <= 0.05                          0.630
## 
## Standardized Root Mean Square Residual:
## 
##   SRMR                                           0.006
## 
## Parameter Estimates:
## 
##   Standard errors                           Robust.sem
##   Information                                 Expected
##   Information saturated (h1) model          Structured
## 
## Regressions:
##                      Estimate  Std.Err  z-value  P(>|z|) ci.lower ci.upper
##   tone_complex_num ~                                                      
##     Africa_num         -0.051    0.139   -0.366    0.714   -0.322    0.221
##     ASPM_z             -0.108    0.026   -4.124    0.000   -0.159   -0.056
##     MCPH1_z            -0.005    0.050   -0.093    0.926   -0.102    0.093
##   ASPM_z ~                                                                
##     Africa_num         -1.338    0.101  -13.315    0.000   -1.535   -1.141
##   MCPH1_z ~                                                               
##     Africa_num         -2.189    0.072  -30.443    0.000   -2.330   -2.048
##    Std.lv  Std.all
##                   
##    -0.051   -0.056
##    -0.108   -0.292
##    -0.005   -0.013
##                   
##    -1.338   -0.542
##                   
##    -2.189   -0.887
## 
## Variances:
##                    Estimate  Std.Err  z-value  P(>|z|) ci.lower ci.upper
##    .tone_complx_nm    0.125    0.016    7.848    0.000    0.094    0.157
##    .ASPM_z            0.702    0.072    9.767    0.000    0.561    0.843
##    .MCPH1_z           0.212    0.037    5.748    0.000    0.140    0.284
##    Std.lv  Std.all
##     0.125    0.927
##     0.702    0.706
##     0.212    0.213
## 
## R-Square:
##                    Estimate
##     tone_complx_nm    0.073
##     ASPM_z            0.294
##     MCPH1_z           0.787  
 Coding Africa and  tone2  as ordered binary factors, the model fit is:  χ  2 (1)=0.98,  p =0.32; CFI=1.00, TLI=1.01, NNFI=1.01 and RFI=0.79. 
 
  
 
 
  Figure 96.    Path analysis model with standardised coefficients and significance stars. Here, we coded tone and macroarea (Africa vs non-Africa) as ordered binary factors ( tone_complex_ord  with No < Yes, and  Africa_ord  with outside Africa < in Africa);  ASPM_z  is  ASPM -D and  MCPH1_z  is  MCPH1 -D. 
 
 
 
 
 Restricted sampling 
 Here I use here only the numerically-coded model. 
 
 
 
  Figure 97.    Path analysis for 1000 restricted samples (i.e., picking one random language per family). The leftmost row of two plots shows the coefficient estimates and the  p -values, respectively, for the five paths in the model (see the path plots above). The rightmost plot shows the various fit indices. The black horiontal lines show: 0.0 (solid), 0.05 (dashed) and 1.0 (dotted). 
 
 
 It can be seen that: 
 
  the models fit are: 
 
 97.6% of the  p -values are not significant 
 mean(CFI) = 0.99, median(CFI) = 1, sd(CFI) = 0.01, IQR(CFI) = 0.01 
 mean(TLI) = 0.98, median(TLI) = 0.99, sd(TLI) = 0.1, IQR(TLI) = 0.15 
 mean(NNFI) = 0.98, median(NNFI) = 0.99, sd(NNFI) = 0.1, IQR(NNFI) = 0.15 
 mean(RFI) = 0.9, median(RFI) = 0.92, sd(RFI) = 0.09, IQR(RFI) = 0.13 
  
  Africa →  ASPM -D: mean = -0.89, median = -0.9, sd = 0.11, IQR = 0.15, 100.0% < 0; 99.8% significant at  α -level 0.05; one-sample one-sided  t -test vs 0:  t (999) = -2.5e+02,  p  = 0;  
  Africa →  MCPH1 -D: mean = -2.4, median = -2.4, sd = 0.079, IQR = 0.11, 100.0% < 0; 100.0% significant at  α -level 0.05; one-sample one-sided  t -test vs 0:  t (999) = -9.6e+02,  p  = 0;  
  Africa →  tone2 : mean = 0.039, median = 0.021, sd = 0.26, IQR = 0.37, 53.6% > 0; 0.0% significant at  α -level 0.05; one-sample one-sided  t -test vs 0:  t (999) = 4.7,  p  = 1.2e-06;  
   ASPM -D →  tone2 : mean = -0.071, median = -0.071, sd = 0.027, IQR = 0.037, 99.8% < 0; 5.9% significant at  α -level 0.05; one-sample one-sided  t -test vs 0:  t (999) = -83,  p  = 0;  
   MCPH1 -D →  tone2 : mean = -0.00016, median = -0.0016, as = 0.1, IQR = 0.14, 50.8% < 0; 0.0% significant at  α -level 0.05; one-sample one-sided  t -test vs 0:  t (999) = -0.051,  p  = 0.48.  
 
 
 
 
 
 Machine Learning techniques 
 
 Decision trees 
 When using the frequency of the two alleles and the  macroarea  as predictors, the decision tree is trivial: it uniformly predicts just the majority value “No.” 
 
 
 
  Figure 98.    Decision tree on the full data using the two alleles and macroarea. 
 
 
 accuracy = 83.9%, sensitivity = NA%, specificity = 83.9%, precision = 0.0%, and recall = NA%. 
 On the 100 training/testing sets: accuracy = 82.9% ±5.8%, sensitivity = 15.8% ±9.0%, specificity = 83.5% ±5.3%, precision = 0.8% ±4.4%, recall = 15.8% ±9.0%. 
 
 
 
  Figure 99.    The success of generalising to the testing sets from the training sets (yellow boxplots) compared to the success on the full data (red segments). 
 
 
 
 
 Random forests 
 
 Including macroarea 
 
 random forests: accuracy = 84.2% ±0.5%, sensitivity = 58.4% ±11.2%, specificity = 84.9% ±0.4%, precision = 8.8% ±2.6%, recall = 58.4% ±11.2% 
 conditional random forests: accuracy = 87.2% ±0.7%, sensitivity = 97.5% ±4.8%, specificity = 86.9% ±0.7%, precision = 21.2% ±5.2%, recall = 97.5% ±4.8% 
 
 
 
 
  Figure 100.    The success of the two random forest methods on the full data. 
 
 
 
 
 
  Figure 101.    Variable importance using three methods: mean decrease in accuracy, mean decrease of the Gini coeficient, and unconditional importance. 
 
 
 
 
 Excluding macroarea 
 
 random forests: accuracy = 82.2% ±1.0%, sensitivity = 42.8% ±4.4%, specificity = 87.3% ±0.6%, precision = 30.3% ±3.5%, recall = 42.8% ±4.4% 
 conditional random forests: accuracy = 87.3% ±0.2%, sensitivity = 81.2% ±3.1%, specificity = 87.7% ±0.0%, precision = 27.6% ±0.0%, recall = 81.2% ±3.1% 
 
 
 
 
  Figure 102.    The success of the two random forest methods on the full data. 
 
 
 
 
 
  Figure 103.    Variable importance using three methods: mean decrease in accuracy, mean decrease of the Gini coeficient, and unconditional importance. 
 
 
 
 
 
 
 
 Tone  counts  
 Here the imputed counts are  rounded  to the nearest integer; please see below for using the actually predicted values. 
 I kept only the entries with non-missing data for the tone  counts ,  ASPM -D and  MCPH1 -D, and if there are more than one possible languages or allele frequencies for a given sample, I only kept those entries that have different tone or allele data. The resulting dataset has 184 observations, distributed among 121 unique Glottolg codes in 35 families (ranging from a minimum of 1 language per family to a maximum of 47, with a mean 5.3 and median 2 languages per family) and 4 macroareas. 
 There are 156:121:121 unique samples:(meta)populations:languages retained, dropping 19:8:200 = {FINRISK, GenDan, GenNed5, gnomAD_asj, gnomAD_bgr, gnomAD_est, gnomAD_fin, gnomAD_jpn, gnomAD_kor, gnomAD_swe, gnomADexomes_AshkenaziJewish, gnomADgenomes_AshkenaziJewish, KRGDB, Qatari, SA001471N, SA001477T, SA001487U, SA001491P, SA001681Q} : {Bulgarian, Burunge, Dutch, Hazara, Mozabite, Oroqen, Qatari, Xibe} : {adze1240, ajie1238, amar1272, ambu1247, anei1239, apma1241, arak1252, arib1241, arop1243, aros1241, aulu1238, awtu1239, ayiw1239, baba1268, bahi1254, bann1247, bign1238, bili1260, boik1241, bulg1262, buru1320, caro1242, cham1313, chek1238, chuu1238, dehu1237, dumb1241, dutc1256, east2443, east2447, efee1239, fiji1243, futu1245, gapa1238, geez1241, gela1263, gilb1244, gulf1241, guma1254, gyel1242, hali1244, hang1263, hano1246, haza1239, hoav1238, iaai1238, iatm1242, idak1243, idun1242, iris1253, iwam1256, juho1239, kaia1245, kair1263, kamb1297, kapi1249, kara1486, kaul1240, kela1255, kele1258, kili1267, kire1240, koko1269, kosr1238, kuan1248, kuma1276, kung1261, kwai1243, kwam1251, kwam1252, kwom1262, labu1248, lala1268, lame1260, lauu1247, lena1238, lewo1242, long1395, loni1238, lonw1238, louu1245, lusi1240, maee1241, mais1250, male1289, malo1243, mana1295, mana1298, maor1246, mars1254, masa1299, matu1261, mbal1255, mbul1263, mehe1243, meke1243, mele1250, mina1269, ming1252, moch1256, moki1238, moks1248, mono1273, motl1237, motu1246, mudu1242, muri1260, muso1238, muss1246, muyu1244, naka1262, nali1244, nami1256, natu1246, naur1243, ndon1254, neha1247, neng1238, ngan1300, niua1240, niue1239, nort2646, nort2836, nort2845, nuku1260, onto1237, oroq1238, paam1238, pate1247, patp1243, pile1238, ping1243, pohn1238, port1285, pulu1242, qima1242, raoo1244, rapa1244, renn1242, rotu1241, rovi1238, russ1264, saaa1240, saam1283, saka1289, sali1295, samo1305, sapo1253, scot1243, siar1238, siee1239, sina1266, sioo1240, sobe1238, sons1242, sout2642, sout2679, sout2807, sout2856, sout2866, sout2869, stan1318, sude1239, surs1246, tahi1242, taki1248, tawa1275, tean1237, teop1238, tiga1245, tigr1271, tiri1258, toab1237, toba1266, toke1240, tong1325, tswa1253, tuam1242, tuml1238, tumz1238, tung1290, tuva1244, ulit1238, urav1235, urip1239, vinm1237, waim1251, wall1257, wata1253, west2500, west2519, woga1249, wole1240, xamt1239, xara1244, xibe1242, yabe1254, yess1239, yima1243, zulu1248}. 
 
 
 
 
 
 
 
 
 
 
 
   
 Africa 
 Eurasia 
 America 
 Papunesia 
 Sum 
 
 
 
 
  0  
 9 
 98 
 4 
 7 
 118 
 
 
  1  
 10 
 6 
 5 
 1 
 22 
 
 
  2  
 16 
 3 
 0 
 2 
 21 
 
 
  3  
 2 
 5 
 0 
 0 
 7 
 
 
  4  
 0 
 8 
 1 
 0 
 9 
 
 
  5  
 1 
 4 
 0 
 0 
 5 
 
 
  6  
 0 
 2 
 0 
 0 
 2 
 
 
  Sum  
 38 
 126 
 10 
 10 
 184 
 
 
 
 
 
 
  Figure 104.    Distribution of tone  counts . 
 
 
 
 
 
  Figure 105.    Distribution of tone  counts  across the world. 
 
 
 
 
 
  Figure 106.    Relationship between tone  counts  (colors) and the two alleles (frequency) by macroarea. 
 
 
 
 Regressions 
 I used a mixed-effects  Poisson model . 
 
  glmer  
 
 All data 
 
  null model : R 2  = 0.0%, ICC = 100.0% 
 the Poisson model is  not  overdispersed:  χ  2 (182) = 112.6,  p  = 1 
  macroarea : R 2  = 23.8%,  p  macroarea/null  = 0.013 
  ASPM :
 
 by itself: R 2  = 7.6%,  β  = -0.37 ± 0.19,  p  ASPM/null  = 0.061 
 quadratic: R 2  = 17.9%,  β ASPM2   = -0.42 ± 0.21,  p  ASPM2/ASPM  = 0.12 
 with  macroarea : R 2  = 24.8%,  p  macroarea/ASPM  = 0.058,  p  ASPM/macroarea  = 0.66 
  
  MCPH1 :
 
 by itself: R 2  = 9.9%,  β  = -0.46 ± 0.19,  p  MCPH1/null  = 0.016 
 quadratic: R 2  = 10.9%,  β MCPH12   = -0.45 ± 0.19,  p  MCPH12/MCPH1  = 0.19 
 with  macroarea : R 2  = 24.1%,  p  macroarea/MCPH1  = 0.15,  p  MCPH1/macroarea  = 0.64 
  
  both alleles  (no  macroarea ):
 
  ASPM  +  MCPH1 : R 2  = 15.7%,  β  ASPM  = -0.27 ± 0.20,  p  ASPM/MCPH1  = 0.18,  β  MCPH1  = -0.37 ± 0.19,  p  MCPH1/ASPM  = 0.043,  p  ASPM+MCPH1/null  = 0.022, 
 interaction: R 2  = 15.4%,  p  ASPM:MCPH1/ASPM+MCPH1  = 0.86 
  
 
 
 
 Randomization 
 We performed 1000 independent replications: 
 
 Regressions with randomizations for tone  counts . 
 
 
 Permute within 
 Macroarea 
 Permute 
 AIC 
 Signif. 
  p   ASPM -D  
  β   ASPM -D  
  p   MCPH1 -D  
  β   MCPH1 -D  
 
 
 
 
 unrestricted 
 none 
 tone 
 0% 
 18% 
 14% 
 4% 
 13% 
 2% 
 
 
 unrestricted 
 none 
 alleles-together 
 1% 
 2% 
 3% 
 0% 
 3% 
 0% 
 
 
 unrestricted 
 none 
 alleles-independent 
 1% 
 3% 
 4% 
 0% 
 3% 
 0% 
 
 
 unrestricted 
 fixef 
 tone 
 0% 
 23% 
 16% 
 33% 
 19% 
 30% 
 
 
 unrestricted 
 fixef 
 alleles-together 
 81% 
 2% 
 3% 
 16% 
 3% 
 12% 
 
 
 unrestricted 
 fixef 
 alleles-independent 
 81% 
 4% 
 3% 
 13% 
 4% 
 6% 
 
 
 macroareas 
 none 
 tone 
 0% 
 44% 
 19% 
 12% 
 31% 
 7% 
 
 
 macroareas 
 none 
 alleles-together 
 18% 
 36% 
 8% 
 6% 
 34% 
 20% 
 
 
 macroareas 
 none 
 alleles-independent 
 20% 
 34% 
 12% 
 7% 
 37% 
 19% 
 
 
 macroareas 
 fixef 
 tone 
 0% 
 31% 
 23% 
 33% 
 21% 
 35% 
 
 
 macroareas 
 fixef 
 alleles-together 
 79% 
 3% 
 4% 
 23% 
 4% 
 26% 
 
 
 macroareas 
 fixef 
 alleles-independent 
 81% 
 4% 
 4% 
 24% 
 4% 
 26% 
 
 
 families 
 none 
 tone 
 24% 
 19% 
 14% 
 28% 
 8% 
 8% 
 
 
 families 
 none 
 alleles-together 
 9% 
 16% 
 12% 
 32% 
 4% 
 4% 
 
 
 families 
 none 
 alleles-independent 
 10% 
 20% 
 20% 
 40% 
 8% 
 8% 
 
 
 families 
 fixef 
 tone 
 18% 
 7% 
 9% 
 63% 
 2% 
 54% 
 
 
 families 
 fixef 
 alleles-together 
 83% 
 4% 
 8% 
 61% 
 2% 
 5% 
 
 
 families 
 fixef 
 alleles-independent 
 82% 
 5% 
 7% 
 59% 
 2% 
 11% 
 
 
 
 
 
 
Regressions on 1000 permuted data. Each plot shows the original result (vertical dashed black line) and the distribution of the permutations for the three possible things to be permuted (colored curves) for each combination of permutation constraints (horizontal panels) and control for  macroarea  (vertical panels) in terms of the effect size  β ;  ASPM -D is on the left and  MCPH1 -D on the right. The vertical dotted black thin line is at 0.0.
 
 
 
 
 Restricted sampling 
 
 
 
  Figure 107.    Results for 1000 restricted samplings. For  ASPM -D (left): 100% of βs are negative when regressing tone on  ASPM  alone (one-sided  t -test < 0:  t (999) = -81.5, mean = -0.48,  p  = 0), 96.7%, when controlling for the macroarea ( t (999) = -52.9, mean = -0.48,  p  = 4.6e-292), and 96.7% when controlling for both macroarea and  MCPH1  ( t (999) = -53.5, mean = -0.52,  p  = 5e-296). For  MCPH1 -D (right): 99.2% of βs are negative when regressing tone on  MCPH1  alone (one-sided  t -test < 0:  t (999) = -70.7, mean = -0.25,  p  = 0), 50.5% when controlling for the macroarea ( t (999) = 2.8, mean = 0.05,  p  = 1), and 38.2% when controlling for both macroarea and  ASPM  ( t (999) = 12.6, mean = 0.24,  p  = 1). 
 
 
 
 
 
  brms  
 
  ASPM  only:
 
  β  = -0.25, 89%HDI = [-0.64, 0.17] 
 posterior probability  p ( β <0) = 0.84 (evidence ratio = 5.2),  p ( β =0) = 0.88 (evidence ratio = 7.5) 
 ROPE = [-0.10, 0.10], % HDI inside ROPE = 21%;  p  ROPE  = 0.187 
 comparison ‘null’ vs ‘ASPM’: [B> L= W>(71%:29%) K>]: moderate evidence for null against ASPM (BF=4.35), LOO=1.00 [SE=1.31], WAIC=0.89 [SE=0.89], KFOLD=4.18 [SE=3.39] 
  
  MCPH1  only:
 
  β  = -0.24, 89%HDI = [-0.65, 0.25] 
 posterior probability  p ( β <0) = 0.8 (evidence ratio = 4),  p ( β =0) = 0.89 (evidence ratio = 8.1) 
 ROPE = [-0.10, 0.10], % HDI inside ROPE = 20.4%;  p  ROPE  = 0.182 
 comparison ‘null’ vs ‘MCPH1’: [B> L> W>(65%:35%) K>]: moderate evidence for null against MCPH1 (BF=6.59), LOO=1.00 [SE=0.83], WAIC=0.63 [SE=0.61], KFOLD=1.49 [SE=1.40] 
  
  both alleles :
 
 comparison ‘null’ vs ‘both’: [B>> L> W>(83%:17%) K>>]: very strong evidence for null against both (BF=46.8), LOO=1.99 [SE=1.23], WAIC=1.58 [SE=1.08], KFOLD=9.05 [SE=3.68] 
 interaction:
 
 posterior probability  p (=0) = 0.93 (evidence ratio = 13) 
 ROPE = [-0.10, 0.10], % HDI inside ROPE = 33.8%;  p  ROPE  = 0.301 
 comparison ‘no interaction’ vs ‘with interaction’: [B>> L> W>>(68%:32%) K>]: strong evidence for no interaction against with interaction (BF=17.1), LOO=0.63 [SE=0.59], WAIC=0.77 [SE=0.30], KFOLD=2.09 [SE=1.95] 
  
  ASPM  (partial):
 
  β  = -0.22, 89%HDI = [-0.66, 0.16] 
 posterior probability  p ( β <0) = 0.81 (evidence ratio = 4.2),  p ( β =0) = 0.9 (evidence ratio = 8.6) 
 ROPE = [-0.10, 0.10], % HDI inside ROPE = 24.2%;  p  ROPE  = 0.216 
  
  MCPH1  (partial):
 
  β  = -0.21, 89%HDI = [-0.64, 0.23] 
 posterior probability  p ( β <0) = 0.78 (evidence ratio = 3.5),  p ( β =0) = 0.89 (evidence ratio = 8.1) 
 ROPE = [-0.10, 0.10], % HDI inside ROPE = 22.4%;  p  ROPE  = 0.199 
  
  
 
 
  
 
  Figure 108.    Posterior distributions (with 50% probability mass highlighted) versus 0.0 (the vertical line) for  ASPM -D (left) and  MCPH1 -D (right). 
 
 
 
  
 
  Figure 109.    Conditional effects of  ASPM -D (left) and  MCPH1 -D (right). 
 
 
 
  
 
  Figure 110.    Posterior predictive checks for  ASPM -D (left) and  MCPH1 -D (right). 
 
 
 
 
 
 Mediation and path analysis 
 
 Mediation analysis 
 
  (g)lm  
 
 All data 
 For  ASPM -D: 
 
   total effect  (TE) of being in Africa on tone: 0.94 (0.40, 1.72),  p =0, decomposed into:  
   average direct effect  (ADE): -0.16 (-0.69, 0.30),  p =0.48, and  
   average indirect effect  (ACME) mediated by  ASPM -D: 1.11 (0.63, 1.79),  p =0, mediating 117.0% (76.3%, 223.9%),  p =0 of the effect, resulting from: 
 
 effect of being in Africa on  ASPM -D: -1.34 ±0.15,  p =1.6e-15, and 
 effect of  ASPM -D on tone: -0.73 ±0.12,  p =4.2e-10. 
  
 
 For  MCPH1 -D: 
 
   TE : 0.69 (0.32, 1.13),  p =0, decomposed into:  
   ADE : 0.44 (-0.44, 1.38),  p =0.32, and  
   ACME : 0.25 (-0.57, 1.06),  p =0.53, mediating 36.6% (-95.5%, 197.0%),  p =0.53 of the effect, resulting from: 
 
 effect of being in Africa on  MCPH1 -D: -2.18 ±0.09,  p =3e-62, and 
 effect of  MCPH1 -D on tone: -0.12 ±0.17,  p =0.5. 
  
 
 
 
 Restricted sampling 
 
 
 
  Figure 111.    Mediation analysis for 1000 restricted samples (i.e., picking one random language per family). The leftmost panels show the distribution of point estimates of the Total Effect (TE), the Direct Effect (ADE) and the Indirect Effect (ACME) for  ASPM  and  MCPH1 ; the middle panels show the distribution of the  p -values for the same effects, while the rightmost panels show the distribution of the regression slopes ( β ) for the two alleles, top: for the regression of the allele frequency on within vs outside Africa, and bottom: for the regression of tone on the allele while controlling for within vs outside Africa. The black vertical lines show: 0.0 (dotted), 0.05 (solid) and 0.10 (dashed). 
 
 
 For  ASPM -D: 
 
   TE : mean = 1.3, median = 1.3; 59.2% significant at  α -level 0.05 and 72.5% significant at  α -level 0.10; 100.0% > 0.0; one-sample one-sided t-test vs 0:  t (999) = 79.3,  p  = 0;  
   ADE : mean = 0.73, median = 0.74; 19.4% significant at  α -level 0.05 and 33.9% significant at  α -level 0.10; 97.4% > 0.0; one-sample one-sided t-test vs 0:  t (999) = 58.4,  p  = 0;  
   ACME : mean = 0.54, median = 0.5; 21.5% significant at  α -level 0.05 and 44.6% significant at  α -level 0.10; 98.4% > 0.0; one-sample one-sided t-test vs 0:  t (999) = 53.7,  p  = 2.6e-297;  
   β(Africa → allele) : mean = -0.89, median = -0.9; 87.0% significant at  α -level 0.05 and 98.9% significant at  α -level 0.10; 100.0% < 0.0; one-sample one-sided t-test vs 0:  t (999) = -237.7,  p  = 0;  
   β(allele → tone | Africa) : mean = -0.38, median = -0.36; 34.1% significant at  α -level 0.05 and 51.0% significant at  α -level 0.10; 98.2% < 0.0; one-sample one-sided t-test vs 0:  t (999) = -66.8,  p  = 0.  
 
 For  MCPH1 -D: 
 
   TE : mean = 1.1, median = 1.1; 57.6% significant at  α -level 0.05 and 70.0% significant at  α -level 0.10; 100.0% > 0.0; one-sample one-sided t-test vs 0:  t (999) = 83.9,  p  = 0;  
   ADE : mean = 3.9, median = 2; 16.4% significant at  α -level 0.05 and 26.1% significant at  α -level 0.10; 83.2% > 0.0; one-sample one-sided t-test vs 0:  t (999) = 17.2,  p  = 1.3e-58;  
   ACME : mean = -2.8, median = -0.89; 5.2% significant at  α -level 0.05 and 11.9% significant at  α -level 0.10; 35.0% > 0.0; one-sample one-sided t-test vs 0:  t (999) = -12.3,  p  = 1;  
   β(Africa → allele) : mean = -2.4, median = -2.4; 100.0% significant at  α -level 0.05 and 100.0% significant at  α -level 0.10; 100.0% < 0.0; one-sample one-sided t-test vs 0:  t (999) = -915.3,  p  = 0;  
   β(allele → tone | Africa) : mean = 0.13, median = 0.1; 5.7% significant at  α -level 0.05 and 11.9% significant at  α -level 0.10; 40.4% < 0.0; one-sample one-sided t-test vs 0:  t (999) = 10.3,  p  = 1.  
 
 Given the low sample size  N  = 35 unique families, relatively few effect sizes are big enough to be significant; however, there are many more significant indirect effects (ACME) for  ASPM -D than for  MCPH1 -D: 34.1% vs 5.7% (6.0 times) for  α -level 0.05, and 51.0% vs 11.9% (4.3 times) for  α -level 0.10. 
 
 
 
  brms  
 
  
 
 
  Figure 112.    Graphical representation of the Bayesian mediation analysis for  ASPM -D showing the means of the effects and the actual partial regression coefficients, with their 89% HDIs and p-ROPEs. The colors reflect the sign of the mean estimate (blue=negative, red=positive, gray=(p-ROPE >= 0.05)); solid=(0 not in the HDI), dashed=(0 is in the HDI). 
 
 
 
  
 
 
  Figure 113.    Graphical representation of the Bayesian mediation analysis for  MCPH1 -D showing the means of the effects and the actual partial regression coefficients, with their 89% HDIs and p-ROPEs. The colors reflect the sign of the mean estimate (blue=negative, red=positive, gray=(p-ROPE >= 0.05)); solid=(0 not in the HDI), dashed=(0 is in the HDI). 
 
 
 
  
 
 
  Figure 114.    Graphical representation of the Bayesian mediation analysis for both  ASPM -D and  MCPH1 -D showing the means of the effects and the actual partial regression coefficients, with their 89% HDIs and p-ROPEs. The colors reflect the sign of the mean estimate (blue=negative, red=positive, gray=(p-ROPE >= 0.05)); solid=(0 not in the HDI), dashed=(0 is in the HDI). 
 
 
 
 
 
 Path analysis 
 Please note that path analysis uses a linear model (so not a Poisson one) for the tone  counts ; also I only use the numeric coding for Africa. 
 
 All data 
 Coding Africa numerically, the model fits the data very well ( χ  2 (1)=0.29,  p =0.59; CFI=1.00, TLI=1.01, NNFI=1.01 and RFI=1.00): 
 
  
 
 
  Figure 115.    Path analysis model with standardised coefficients and significance stars. Here, macroarea (Africa vs non-Africa) is coded as numeric binary ( Africa_num  with in Africa=1);  ASPM_z  is  ASPM -D and  MCPH1_z  is  MCPH1 -D.. 
 
 
  ## lavaan 0.6-8 ended normally after 28 iterations
## 
##   Estimator                                         ML
##   Optimization method                           NLMINB
##   Number of model parameters                         8
##                                                       
##   Number of observations                           184
##                                                       
## Model Test User Model:
##                                                       
##   Test statistic                                 0.292
##   Degrees of freedom                                 1
##   P-value (Chi-square)                           0.589
## 
## Model Test Baseline Model:
## 
##   Test statistic                               369.194
##   Degrees of freedom                                 6
##   P-value                                        0.000
## 
## User Model versus Baseline Model:
## 
##   Comparative Fit Index (CFI)                    1.000
##   Tucker-Lewis Index (TLI)                       1.012
## 
## Loglikelihood and Information Criteria:
## 
##   Loglikelihood user model (H0)               -663.138
##   Loglikelihood unrestricted model (H1)       -662.992
##                                                       
##   Akaike (AIC)                                1342.276
##   Bayesian (BIC)                              1367.995
##   Sample-size adjusted Bayesian (BIC)         1342.657
## 
## Root Mean Square Error of Approximation:
## 
##   RMSEA                                          0.000
##   90 Percent confidence interval - lower         0.000
##   90 Percent confidence interval - upper         0.159
##   P-value RMSEA <= 0.05                          0.666
## 
## Standardized Root Mean Square Residual:
## 
##   SRMR                                           0.005
## 
## Parameter Estimates:
## 
##   Standard errors                           Robust.sem
##   Information                                 Expected
##   Information saturated (h1) model          Structured
## 
## Regressions:
##                    Estimate  Std.Err  z-value  P(>|z|) ci.lower ci.upper
##   n_tones ~                                                             
##     Africa_num       -0.265    0.601   -0.441    0.659   -1.443    0.913
##     ASPM_z           -0.490    0.114   -4.291    0.000   -0.713   -0.266
##     MCPH1_z          -0.131    0.229   -0.571    0.568   -0.580    0.319
##   ASPM_z ~                                                              
##     Africa_num       -1.338    0.099  -13.477    0.000   -1.533   -1.144
##   MCPH1_z ~                                                             
##     Africa_num       -2.180    0.071  -30.517    0.000   -2.320   -2.040
##    Std.lv  Std.all
##                   
##    -0.265   -0.075
##    -0.490   -0.342
##    -0.131   -0.091
##                   
##    -1.338   -0.543
##                   
##    -2.180   -0.885
## 
## Variances:
##                    Estimate  Std.Err  z-value  P(>|z|) ci.lower ci.upper
##    .n_tones           1.790    0.273    6.566    0.000    1.256    2.324
##    .ASPM_z            0.701    0.071    9.837    0.000    0.561    0.841
##    .MCPH1_z           0.216    0.036    5.943    0.000    0.145    0.287
##    Std.lv  Std.all
##     1.790    0.879
##     0.701    0.705
##     0.216    0.217
## 
## R-Square:
##                    Estimate
##     n_tones           0.121
##     ASPM_z            0.295
##     MCPH1_z           0.783  
 
 
 Restricted sampling 
 
 
 
  Figure 116.    Path analysis for 1000 restricted samples (i.e., picking one random language per family). The leftmost row of two plots shows the coefficient estimates and the  p -values, respectively, for the five paths in the model (see the path plots above). The rightmost plot shows the various fit indices. The black horiontal lines show: 0.0 (solid), 0.05 (dashed) and 1.0 (dotted). 
 
 
 It can be seen that: 
 
  the models fits: 
 
 97.9% of the  p -values are not significant 
 mean(CFI) = 0.99, median(CFI) = 1, sd(CFI) = 0.01, IQR(CFI) = 0.01 
 mean(TLI) = 0.99, median(TLI) = 1.01, sd(TLI) = 0.09, IQR(TLI) = 0.15 
 mean(NNFI) = 0.99, median(NNFI) = 1.01, sd(NNFI) = 0.09, IQR(NNFI) = 0.15 
 mean(RFI) = 0.91, median(RFI) = 0.93, sd(RFI) = 0.08, IQR(RFI) = 0.13 
  
  Africa →  ASPM -D: mean = -0.88, median = -0.89, sd = 0.12, IQR = 0.16, 100.0% < 0; 99.9% significant at  α -level 0.05; one-sample one-sided  t -test vs 0:  t (999) = -2.3e+02,  p  = 0  
  Africa →  MCPH1 -D: mean = -2.4, median = -2.4, sd = 0.083, IQR = 0.12, 100.0% < 0; 100.0% significant at  α -level 0.05; one-sample one-sided  t -test vs 0:  t (999) = -9.1e+02,  p  = 0  
  Africa → tone  counts : mean = 0.77, median = 0.83, sd = 1.1, IQR = 1.6, 74.9% > 0; 10.0% significant at  α -level 0.05; one-sample one-sided  t -test vs 0:  t (999) = 22,  p  = 2e-86  
   ASPM -D → tone  counts : mean = -0.32, median = -0.31, sd = 0.16, IQR = 0.22, 98.7% < 0; 22.5% significant at  α -level 0.05; one-sample one-sided  t -test vs 0:  t (999) = -63,  p  = 0  
   MCPH1 -D → tone  counts : mean = 0.019, median = 0.034, as = 0.45, IQR = 0.65, 47.4% < 0; 3.3% significant at  α -level 0.05; one-sample one-sided  t -test vs 0:  t (999) = 1.3,  p  = 0.91  
 
 
 
 
 
 Unrounded (raw) imputed counts 
 I kept only the entries with non-missing data for the tone  counts ,  ASPM -D and  MCPH1 -D, and if there are more than one possible languages or allele frequencies for a given sample, I only kept those entries that have different tone or allele data. The resulting dataset has 200 observations, distributed among 136 unique Glottolg codes in 37 families (ranging from a minimum of 1 language per family to a maximum of 51, with a mean 5.4 and median 2 languages per family) and 4 macroareas. 
 
 
 
  Figure 117.    Distribution of tone  counts  (unrounded). 
 
 
 
 
 
  Figure 118.    Distribution of tone  counts  (unrounded) across the world. 
 
 
 
 
 
  Figure 119.    Relationship between tone  counts  (unrounded; colors) and the two alleles (frequency) by macroarea. 
 
 
 
 
 
 Power analysis 
 I use simulations for power analysis (as implemented by package  simr ), focusing on the effect of  ASPM -D on  tone1  using  glmer , i.e. logistic regression with  ASPM -D as fixed effect and controlling for language  family  (as random effect) and  macroarea  as fixed effect. 
 
 Observed power 
 The observed effect size of  ASPM -D is  β   ASPM -D  = -0.4,  p   ASPM -D  = 0.41, with an ICC = 68.4% on 35 level-2 groups (families) and 181 observations (languages/samples). The  observed  (post-hoc) power 1 -  β  = %, 95%CI = . 
 
 
 Changing the number of languages 
 If we keep the families but change the number of languages per family: 
 
 
 
  Figure 120.    Estimated power (with 95%) when changing the number of languages but keeping everything else constant. 
 
 
 
 
 Changing the number of families 
 If we change the number of families: 
 
 
 
  Figure 121.    Estimated power (with 95%) when changing the number of language families but keeping everything else constant. 
 
 
 
 
 Changing the number of families and languages 
 If we change the number of families and the number of languages per family: 
 
 
 
  Figure 122.    Estimated power when changing the number of language families and the number of languages per family, but keeping everything else constant. Color is proportional to power and the shape shows if the power is > 80%. The two vertical dotted lines are the approximate number of families in Ethnologue (blue, ~150) and Glottolog (black, ~420). The horizontal dotted lines are summaries of the number of languages in Glottolog: the mean (red, ~20), the median (black, 2) and the median excluding isolates (blue, 5); not shown is the maxmimum (~1400 in Atlantic-Congo). 
 
 
 
 
 
 
 Appendix I: Gaussian Process 
 Here I model language contact with a  2D Gaussian Process  as suggested in, for example,  McElreath (2020) , using  brms ’s  gp() .  tone  is regressed on  ASPM -D and  MCPH1 -D with language  family  and  (meta)population  as (nested) random effects, and a 2D Gaussian process separately for each  macroarea . 
 
  tone1  
 
  ASPM  only:
 
  β  = -0.88, 89%HDI = [-1.63, -0.06] 
 posterior probability  p ( β <0) = 0.96 (evidence ratio = 25),  p ( β =0) = 0.59 (evidence ratio = 1.4) 
 ROPE = [-0.18, 0.18], % HDI inside ROPE = 3.5%;  p  ROPE  = 0.065 
 comparison ‘null’ vs ‘ASPM’: [B= L= W=(33%:67%) K=]: anecdotal evidence for null against ASPM (BF=1.3), LOO=-0.05 [SE=2.69], WAIC=-0.69 [SE=2.74], KFOLD=-1.21 [SE=3.41] 
  
  MCPH1  only:
 
  β  = -1.03, 89%HDI = [-1.63, -0.45] 
 posterior probability  p ( β <0) = 0.99 (evidence ratio = 1.7e+02),  p ( β =0) = 0.15 (evidence ratio = 0.17) 
 ROPE = [-0.18, 0.18], % HDI inside ROPE = 0%;  p  ROPE  = 0.011 
 comparison ‘null’ vs ‘MCPH1’: [B< L< W<(10%:90%) K=]: moderate evidence for MCPH1 against null (BF=0.301), LOO=-2.13 [SE=1.73], WAIC=-2.22 [SE=1.73], KFOLD=-2.02 [SE=2.70] 
  
  both alleles :
 
 comparison ‘null’ vs ‘both’: [B= L= W=(9%:91%) K=]: anecdotal evidence for both against null (BF=0.704), LOO=-1.87 [SE=2.93], WAIC=-2.34 [SE=2.95], KFOLD=-2.34 [SE=3.50] 
 interaction:
 
 posterior probability  p (=0) = 0.87 (evidence ratio = 6.6) 
 ROPE = [-0.18, 0.18], % HDI inside ROPE = 31.5%;  p  ROPE  = 0.28 
 comparison ‘no interaction’ vs ‘with interaction’: [B> L>> W>(76%:24%) K>]: moderate evidence for no interaction against with interaction (BF=5.75), LOO=1.49 [SE=0.73], WAIC=1.16 [SE=0.66], KFOLD=2.70 [SE=2.09] 
  
  ASPM  (partial):
 
  β  = -0.58, 89%HDI = [-1.35, 0.20] 
 posterior probability  p ( β <0) = 0.88 (evidence ratio = 7.4),  p ( β =0) = 0.76 (evidence ratio = 3.2) 
 ROPE = [-0.18, 0.18], % HDI inside ROPE = 15.7%;  p  ROPE  = 0.14 
  
  MCPH1  (partial):
 
  β  = -0.85, 89%HDI = [-1.47, -0.25] 
 posterior probability  p ( β <0) = 0.99 (evidence ratio = 77),  p ( β =0) = 0.41 (evidence ratio = 0.69) 
 ROPE = [-0.18, 0.18], % HDI inside ROPE = 0%;  p  ROPE  = 0.032 
  
  
 
 
  
 
  Figure 123.    Posterior distributions (with 50% probability mass highlighted) versus 0.0 (the vertical line) for  ASPM -D (left) and  MCPH1 -D (right). Please note that I have cut the x-axis at 2.5 as the distributions of the sdgp and lscale have a few extreme outliers which would make the plots impossible to see. 
 
 
 
  
 
  Figure 124.    Conditional effects of  ASPM -D (left) and  MCPH1 -D (right). 
 
 
 
  
 
  Figure 125.    Posterior predictive checks for  ASPM -D (left) and  MCPH1 -D (right). 
 
 
 
  
 
  Figure 126.    Confusion matrices for  ASPM -D (left) and  MCPH1 -D (right). 
 
 
 
 
  tone2  
 
  ASPM  only:
 
  β  = -1.15, 89%HDI = [-2.12, -0.16] 
 posterior probability  p ( β <0) = 0.97 (evidence ratio = 33),  p ( β =0) = 0.5 (evidence ratio = 1) 
 ROPE = [-0.18, 0.18], % HDI inside ROPE = 0.6%;  p  ROPE  = 0.044 
 comparison ‘null’ vs ‘ASPM’: [B= L= W=(41%:59%) K=]: anecdotal evidence for ASPM against null (BF=0.767), LOO=-0.34 [SE=1.58], WAIC=-0.34 [SE=1.59], KFOLD=-0.07 [SE=1.64] 
  
  MCPH1  only:
 
  β  = -0.63, 89%HDI = [-1.27, -0.04] 
 posterior probability  p ( β <0) = 0.95 (evidence ratio = 19),  p ( β =0) = 0.68 (evidence ratio = 2.1) 
 ROPE = [-0.18, 0.18], % HDI inside ROPE = 6.5%;  p  ROPE  = 0.099 
 comparison ‘null’ vs ‘MCPH1’: [B= L= W=(55%:45%) K=]: anecdotal evidence for null against MCPH1 (BF=2.14), LOO=0.06 [SE=1.58], WAIC=0.19 [SE=1.58], KFOLD=0.88 [SE=1.88] 
  
  both alleles :
 
 comparison ‘null’ vs ‘both’: [B> L= W=(45%:55%) K=]: moderate evidence for null against both (BF=4.38), LOO=-0.23 [SE=1.82], WAIC=-0.19 [SE=1.81], KFOLD=0.17 [SE=1.92] 
 interaction:
 
 posterior probability  p (=0) = 0.83 (evidence ratio = 4.7) 
 ROPE = [-0.18, 0.18], % HDI inside ROPE = 22.4%;  p  ROPE  = 0.2 
 comparison ‘no interaction’ vs ‘with interaction’: [B> L> W>(62%:38%) K>>]: moderate evidence for no interaction against with interaction (BF=6.35), LOO=0.53 [SE=0.37], WAIC=0.48 [SE=0.37], KFOLD=2.63 [SE=1.03] 
  
  ASPM  (partial):
 
  β  = -0.88, 89%HDI = [-1.96, 0.15] 
 posterior probability  p ( β <0) = 0.91 (evidence ratio = 10),  p ( β =0) = 0.68 (evidence ratio = 2.1) 
 ROPE = [-0.18, 0.18], % HDI inside ROPE = 9.9%;  p  ROPE  = 0.092 
  
  MCPH1  (partial):
 
  β  = -0.42, 89%HDI = [-1.04, 0.21] 
 posterior probability  p ( β <0) = 0.86 (evidence ratio = 6.3),  p ( β =0) = 0.83 (evidence ratio = 4.7) 
 ROPE = [-0.18, 0.18], % HDI inside ROPE = 24.1%;  p  ROPE  = 0.214 
  
  
 
 
  
 
  Figure 127.    Posterior distributions (with 50% probability mass highlighted) versus 0.0 (the vertical line) for  ASPM -D (left) and  MCPH1 -D (right). Please note that I have cut the x-axis at 2.5 as the distributions of the sdgp and lscale have a few extreme outliers which would make the plots impossible to see. 
 
 
 
  
 
  Figure 128.    Conditional effects of  ASPM -D (left) and  MCPH1 -D (right). 
 
 
 
  
 
  Figure 129.    Posterior predictive checks for  ASPM -D (left) and  MCPH1 -D (right). 
 
 
 
  
 
  Figure 130.    Confusion matrices for  ASPM -D (left) and  MCPH1 -D (right). 
 
 
 
 
 Tone  counts  
 
  ASPM  only:
 
  β  = -0.22, 89%HDI = [-0.66, 0.24] 
 posterior probability  p ( β <0) = 0.78 (evidence ratio = 3.5),  p ( β =0) = 0.9 (evidence ratio = 8.9) 
 ROPE = [-0.10, 0.10], % HDI inside ROPE = 23.7%;  p  ROPE  = 0.211 
 comparison ‘null’ vs ‘ASPM’: [B> L>> W>>(90%:10%) K=]: moderate evidence for null against ASPM (BF=9.37), LOO=2.40 [SE=1.07], WAIC=2.20 [SE=1.01], KFOLD=1.54 [SE=1.62] 
  
  MCPH1  only:
 
  β  = -0.41, 89%HDI = [-0.77, -0.08] 
 posterior probability  p ( β <0) = 0.95 (evidence ratio = 21),  p ( β =0) = 0.7 (evidence ratio = 2.3) 
 ROPE = [-0.10, 0.10], % HDI inside ROPE = 1.2%;  p  ROPE  = 0.058 
 comparison ‘null’ vs ‘MCPH1’: [B> L> W>(93%:7%) K=]: moderate evidence for null against MCPH1 (BF=3.35), LOO=2.30 [SE=1.36], WAIC=2.53 [SE=1.30], KFOLD=1.72 [SE=1.79] 
  
  both alleles :
 
 comparison ‘null’ vs ‘both’: [B>> L> W>(97%:3%) K>]: strong evidence for null against both (BF=14), LOO=3.61 [SE=2.03], WAIC=3.47 [SE=1.89], KFOLD=3.27 [SE=2.59] 
 interaction:
 
 posterior probability  p (=0) = 0.93 (evidence ratio = 12) 
 ROPE = [-0.10, 0.10], % HDI inside ROPE = 33.7%;  p  ROPE  = 0.3 
 comparison ‘no interaction’ vs ‘with interaction’: [B> L>> W>>(68%:32%) K>>]: moderate evidence for no interaction against with interaction (BF=6.73), LOO=1.03 [SE=0.44], WAIC=0.74 [SE=0.37], KFOLD=4.04 [SE=1.72] 
  
  ASPM  (partial):
 
  β  = -0.27, 89%HDI = [-0.67, 0.17] 
 posterior probability  p ( β <0) = 0.84 (evidence ratio = 5.4),  p ( β =0) = 0.88 (evidence ratio = 7.5) 
 ROPE = [-0.10, 0.10], % HDI inside ROPE = 19.5%;  p  ROPE  = 0.174 
  
  MCPH1  (partial):
 
  β  = -0.4, 89%HDI = [-0.72, -0.11] 
 posterior probability  p ( β <0) = 0.97 (evidence ratio = 29),  p ( β =0) = 0.67 (evidence ratio = 2) 
 ROPE = [-0.10, 0.10], % HDI inside ROPE = 0%;  p  ROPE  = 0.049 
  
  
 
 
  
 
  Figure 131.    Posterior distributions (with 50% probability mass highlighted) versus 0.0 (the vertical line) for  ASPM -D (left) and  MCPH1 -D (right). Please note that I have cut the x-axis at 2.5 as the distributions of the sdgp and lscale have a few extreme outliers which would make the plots impossible to see. 
 
 
 
  
 
  Figure 132.    Conditional effects of  ASPM -D (left) and  MCPH1 -D (right). 
 
 
 
  
 
  Figure 133.    Posterior predictive checks for  ASPM -D (left) and  MCPH1 -D (right). 
 
 
 
 
 
 Appendix II: Sensitivity to the prior 
 Here I explore the sensitivity to the prior of the  brms  models, focusing on each “derived” allele independently. 
 
  tone1  
 
  ASPM -D 
 All models show good mixing and convergence (not shown). 
 
 Comparing the posterior distribution for  ASPM -D frequency (z-scored) for various prior distributions. The HDI is a 89%HDI;  p ( β <0) and  p ( β =0) show the posterior probability (and the evidence ratio, in parantheses); the ROPE is [-0.18, 0.18]. 
 
 
 
 
 
 
 
 
 
 
 
 
 Prior name 
 Prior distribution 
  β  
 HDI 
  p ( β <0) 
  p ( β =0) 
 %HDI in ROPE 
  p  ROPE  
 
 
 
 
 default 
 student_t(3, 0, 3) 
 -0.70 
 [-1.52, 0.33] 
 0.89 (8.0) 
 0.73 (2.7) 
 13.3% 
 0.12 
 
 
 flat 
 normal(0, 10) 
 -0.74 
 [-1.63, 0.24] 
 0.9 (9.0) 
 0.89 (8.0) 
 13.1% 
 0.12 
 
 
 default_normal 
 normal(0, 5) 
 -0.70 
 [-1.60, 0.24] 
 0.89 (8.5) 
 0.8 (4.0) 
 13.8% 
 0.12 
 
 
 narrow_0 
 student_t(3, 0, 1) 
 -0.55 
 [-1.32, 0.23] 
 0.87 (7.0) 
 0.55 (1.2) 
 18.7% 
 0.17 
 
 
 verynarrow_0 
 student_t(3, 0, 0.1) 
 -0.05 
 [-0.28, 0.16] 
 0.62 (1.6) 
 0.49 (1.0) 
 91.1% 
 0.82 
 
 
 negative_default 
 student_t(3, -1, 3) 
 -0.75 
 [-1.71, 0.13] 
 0.91 (10.0) 
 0.73 (2.7) 
 11.4% 
 0.11 
 
 
 negative_narrow 
 student_t(3, -1, 1) 
 -0.82 
 [-1.60, -0.06] 
 0.96 (25.1) 
 0.47 (0.9) 
 4.0% 
 0.07 
 
 
 verynegative_default 
 student_t(3, -3, 3) 
 -0.87 
 [-1.83, 0.10] 
 0.94 (14.5) 
 0.79 (3.7) 
 8.3% 
 0.08 
 
 
 verynegative_narrow 
 student_t(3, -3, 1) 
 -1.33 
 [-2.44, -0.21] 
 0.98 (51.6) 
 0.8 (3.9) 
 0.0% 
 0.03 
 
 
 positive_default 
 student_t(3, 1, 3) 
 -0.62 
 [-1.52, 0.28] 
 0.87 (6.8) 
 0.76 (3.2) 
 14.0% 
 0.12 
 
 
 positive_narrow 
 student_t(3, 1, 1) 
 -0.34 
 [-1.13, 0.52] 
 0.74 (2.9) 
 0.74 (2.9) 
 25.7% 
 0.23 
 
 
 verypositive_default 
 student_t(3, 3, 3) 
 -0.55 
 [-1.39, 0.44] 
 0.84 (5.2) 
 0.86 (6.4) 
 17.8% 
 0.16 
 
 
 verypositive_narrow 
 student_t(3, 3, 1) 
 -0.23 
 [-1.12, 0.78] 
 0.66 (1.9) 
 0.97 (27.8) 
 25.0% 
 0.22 
 
 
 informative 
 student_t(3, -0.7, 3) 
 -0.72 
 [-1.64, 0.21] 
 0.9 (9.2) 
 0.73 (2.7) 
 13.0% 
 0.12 
 
 
 
 
 
  MCPH1 -D 
 All models show good mixing and convergence (not shown). 
 
 Comparing the posterior distribution for  MCPH1 -D frequency (z-scored) for various prior distributions. The HDI is a 89%HDI;  p ( β <0) and  p ( β =0) show the posterior probability (and the evidence ratio, in parantheses); the ROPE is [-0.18, 0.18]. 
 
 
 
 
 
 
 
 
 
 
 
 
 Prior name 
 Prior distribution 
  β  
 HDI 
  p ( β <0) 
  p ( β =0) 
 %HDI in ROPE 
  p  ROPE  
 
 
 
 
 default 
 student_t(3, 0, 3) 
 -0.65 
 [-1.69, 0.47] 
 0.84 (5.2) 
 0.75 (3.0) 
 14.3% 
 0.13 
 
 
 flat 
 normal(0, 10) 
 -0.68 
 [-1.76, 0.48] 
 0.85 (5.5) 
 0.9 (8.6) 
 13.6% 
 0.12 
 
 
 default_normal 
 normal(0, 5) 
 -0.67 
 [-1.76, 0.47] 
 0.84 (5.1) 
 0.83 (4.8) 
 14.9% 
 0.13 
 
 
 narrow_0 
 student_t(3, 0, 1) 
 -0.49 
 [-1.35, 0.46] 
 0.82 (4.4) 
 0.56 (1.3) 
 18.6% 
 0.17 
 
 
 verynarrow_0 
 student_t(3, 0, 0.1) 
 -0.04 
 [-0.25, 0.17] 
 0.59 (1.4) 
 0.52 (1.1) 
 94.0% 
 0.84 
 
 
 negative_default 
 student_t(3, -1, 3) 
 -0.73 
 [-1.79, 0.35] 
 0.87 (6.6) 
 0.75 (3.0) 
 12.6% 
 0.11 
 
 
 negative_narrow 
 student_t(3, -1, 1) 
 -0.80 
 [-1.74, 0.03] 
 0.93 (12.7) 
 0.57 (1.3) 
 7.5% 
 0.09 
 
 
 verynegative_default 
 student_t(3, -3, 3) 
 -0.85 
 [-1.93, 0.27] 
 0.9 (8.7) 
 0.8 (3.9) 
 11.5% 
 0.10 
 
 
 verynegative_narrow 
 student_t(3, -3, 1) 
 -1.31 
 [-2.51, -0.09] 
 0.97 (27.8) 
 0.84 (5.3) 
 2.1% 
 0.04 
 
 
 positive_default 
 student_t(3, 1, 3) 
 -0.61 
 [-1.64, 0.52] 
 0.81 (4.3) 
 0.77 (3.3) 
 15.6% 
 0.14 
 
 
 positive_narrow 
 student_t(3, 1, 1) 
 -0.22 
 [-1.26, 0.80] 
 0.63 (1.7) 
 0.75 (2.9) 
 23.6% 
 0.21 
 
 
 verypositive_default 
 student_t(3, 3, 3) 
 -0.47 
 [-1.56, 0.59] 
 0.77 (3.3) 
 0.87 (6.4) 
 18.1% 
 0.16 
 
 
 verypositive_narrow 
 student_t(3, 3, 1) 
 -0.06 
 [-1.39, 1.16] 
 0.55 (1.2) 
 0.96 (22.6) 
 19.9% 
 0.18 
 
 
 informative 
 student_t(3, -0.6, 3) 
 -0.66 
 [-1.83, 0.42] 
 0.83 (4.9) 
 0.74 (2.8) 
 13.0% 
 0.12 
 
 
 
 
 
 
  tone2  
 
  ASPM -D 
 All models show good mixing and convergence (not shown). 
 
 Comparing the posterior distribution for  ASPM -D frequency (z-scored) for various prior distributions. The HDI is a 89%HDI;  p ( β <0) and  p ( β =0) show the posterior probability (and the evidence ratio, in parantheses); the ROPE is [-0.18, 0.18]. 
 
 
 
 
 
 
 
 
 
 
 
 
 Prior name 
 Prior distribution 
  β  
 HDI 
  p ( β <0) 
  p ( β =0) 
 %HDI in ROPE 
  p  ROPE  
 
 
 
 
 default 
 student_t(3, 0, 3) 
 -1.30 
 [-2.72, 0.17] 
 0.93 (13.7) 
 0.56 (1.3) 
 6.3% 
 0.06 
 
 
 flat 
 normal(0, 10) 
 -1.76 
 [-3.44, 0.22] 
 0.95 (20.4) 
 0.71 (2.5) 
 3.8% 
 0.03 
 
 
 default_normal 
 normal(0, 5) 
 -1.50 
 [-3.04, 0.22] 
 0.94 (17.0) 
 0.63 (1.7) 
 5.3% 
 0.05 
 
 
 narrow_0 
 student_t(3, 0, 1) 
 -0.67 
 [-1.73, 0.40] 
 0.84 (5.4) 
 0.52 (1.1) 
 15.6% 
 0.14 
 
 
 verynarrow_0 
 student_t(3, 0, 0.1) 
 -0.03 
 [-0.23, 0.20] 
 0.55 (1.2) 
 0.5 (1.0) 
 94.4% 
 0.84 
 
 
 negative_default 
 student_t(3, -1, 3) 
 -1.42 
 [-2.85, 0.07] 
 0.95 (17.7) 
 0.55 (1.2) 
 4.9% 
 0.05 
 
 
 negative_narrow 
 student_t(3, -1, 1) 
 -1.05 
 [-1.98, 0.02] 
 0.96 (23.1) 
 0.44 (0.8) 
 4.3% 
 0.06 
 
 
 verynegative_default 
 student_t(3, -3, 3) 
 -1.76 
 [-3.33, -0.17] 
 0.98 (40.2) 
 0.54 (1.2) 
 0.2% 
 0.03 
 
 
 verynegative_narrow 
 student_t(3, -3, 1) 
 -1.89 
 [-3.12, -0.61] 
 0.99 (120.2) 
 0.57 (1.4) 
 0.0% 
 0.01 
 
 
 positive_default 
 student_t(3, 1, 3) 
 -1.21 
 [-2.58, 0.36] 
 0.91 (10.1) 
 0.64 (1.8) 
 7.9% 
 0.07 
 
 
 positive_narrow 
 student_t(3, 1, 1) 
 -0.45 
 [-1.81, 0.77] 
 0.7 (2.3) 
 0.7 (2.3) 
 19.6% 
 0.17 
 
 
 verypositive_default 
 student_t(3, 3, 3) 
 -1.14 
 [-2.60, 0.44] 
 0.89 (7.8) 
 0.77 (3.3) 
 9.1% 
 0.08 
 
 
 verypositive_narrow 
 student_t(3, 3, 1) 
 -0.40 
 [-1.94, 1.51] 
 0.68 (2.1) 
 0.94 (16.3) 
 14.2% 
 0.13 
 
 
 informative 
 student_t(3, -1.3, 3) 
 -1.48 
 [-2.94, 0.10] 
 0.95 (20.5) 
 0.53 (1.1) 
 3.9% 
 0.04 
 
 
 
 
 
  MCPH1 -D 
 All models show good mixing and convergence (not shown). 
 
 Comparing the posterior distribution for  MCPH1 -D frequency (z-scored) for various prior distributions. The HDI is a 89%HDI;  p ( β <0) and  p ( β =0) show the posterior probability (and the evidence ratio, in parantheses); the ROPE is [-0.18, 0.18]. 
 
 
 
 
 
 
 
 
 
 
 
 
 Prior name 
 Prior distribution 
  β  
 HDI 
  p ( β <0) 
  p ( β =0) 
 %HDI in ROPE 
  p  ROPE  
 
 
 
 
 default 
 student_t(3, 0, 3) 
 -0.93 
 [-2.41, 0.53] 
 0.85 (5.6) 
 0.68 (2.2) 
 10.6% 
 0.09 
 
 
 flat 
 normal(0, 10) 
 -1.23 
 [-3.11, 0.51] 
 0.88 (7.0) 
 0.83 (5.0) 
 8.2% 
 0.07 
 
 
 default_normal 
 normal(0, 5) 
 -1.07 
 [-2.60, 0.66] 
 0.86 (6.2) 
 0.75 (2.9) 
 9.2% 
 0.08 
 
 
 narrow_0 
 student_t(3, 0, 1) 
 -0.47 
 [-1.61, 0.68] 
 0.75 (3.0) 
 0.57 (1.3) 
 18.7% 
 0.17 
 
 
 verynarrow_0 
 student_t(3, 0, 0.1) 
 -0.01 
 [-0.25, 0.19] 
 0.53 (1.1) 
 0.5 (1.0) 
 93.8% 
 0.84 
 
 
 negative_default 
 student_t(3, -1, 3) 
 -1.05 
 [-2.58, 0.45] 
 0.88 (7.0) 
 0.66 (2.0) 
 8.8% 
 0.08 
 
 
 negative_narrow 
 student_t(3, -1, 1) 
 -0.89 
 [-1.98, 0.15] 
 0.92 (10.9) 
 0.5 (1.0) 
 8.1% 
 0.07 
 
 
 verynegative_default 
 student_t(3, -3, 3) 
 -1.31 
 [-2.88, 0.30] 
 0.92 (10.8) 
 0.73 (2.7) 
 6.7% 
 0.06 
 
 
 verynegative_narrow 
 student_t(3, -3, 1) 
 -1.73 
 [-3.11, -0.32] 
 0.98 (39.4) 
 0.75 (2.9) 
 0.0% 
 0.02 
 
 
 positive_default 
 student_t(3, 1, 3) 
 -0.81 
 [-2.25, 0.84] 
 0.81 (4.4) 
 0.72 (2.6) 
 12.2% 
 0.11 
 
 
 positive_narrow 
 student_t(3, 1, 1) 
 -0.08 
 [-1.36, 1.31] 
 0.53 (1.1) 
 0.69 (2.2) 
 19.5% 
 0.17 
 
 
 verypositive_default 
 student_t(3, 3, 3) 
 -0.67 
 [-2.30, 0.85] 
 0.77 (3.4) 
 0.82 (4.5) 
 13.8% 
 0.12 
 
 
 verypositive_narrow 
 student_t(3, 3, 1) 
 0.40 
 [-1.51, 2.62] 
 0.42 (0.7) 
 0.94 (14.7) 
 11.8% 
 0.10 
 
 
 informative 
 student_t(3, -0.9, 3) 
 -1.01 
 [-2.44, 0.46] 
 0.88 (7.5) 
 0.69 (2.2) 
 10.1% 
 0.09 
 
 
 
 
 
 
 Tone  counts  
 
  ASPM -D 
 All models show good mixing and convergence (not shown). 
 
 Comparing the posterior distribution for  ASPM -D frequency (z-scored) for various prior distributions. The HDI is a 89%HDI;  p ( β <0) and  p ( β =0) show the posterior probability (and the evidence ratio, in parantheses); the ROPE is [-0.10, 0.10]. 
 
 
 
 
 
 
 
 
 
 
 
 
 Prior name 
 Prior distribution 
  β  
 HDI 
  p ( β <0) 
  p ( β =0) 
 %HDI in ROPE 
  p  ROPE  
 
 
 
 
 default 
 student_t(3, 0, 3) 
 -0.24 
 [-0.65, 0.16] 
 0.82 (4.7) 
 0.89 (8.1) 
 22.2% 
 0.20 
 
 
 flat 
 normal(0, 10) 
 -0.25 
 [-0.67, 0.16] 
 0.83 (5.0) 
 0.96 (23.4) 
 21.1% 
 0.19 
 
 
 default_normal 
 normal(0, 5) 
 -0.24 
 [-0.66, 0.16] 
 0.82 (4.7) 
 0.92 (12.1) 
 21.8% 
 0.19 
 
 
 narrow_0 
 student_t(3, 0, 1) 
 -0.22 
 [-0.63, 0.18] 
 0.81 (4.4) 
 0.75 (2.9) 
 23.4% 
 0.21 
 
 
 verynarrow_0 
 student_t(3, 0, 0.1) 
 -0.04 
 [-0.22, 0.14] 
 0.63 (1.7) 
 0.53 (1.1) 
 72.9% 
 0.65 
 
 
 negative_default 
 student_t(3, -1, 3) 
 -0.26 
 [-0.69, 0.15] 
 0.83 (5.0) 
 0.9 (8.5) 
 21.6% 
 0.19 
 
 
 negative_narrow 
 student_t(3, -1, 1) 
 -0.32 
 [-0.74, 0.07] 
 0.9 (8.9) 
 0.76 (3.2) 
 14.3% 
 0.14 
 
 
 verynegative_default 
 student_t(3, -3, 3) 
 -0.28 
 [-0.69, 0.14] 
 0.86 (6.2) 
 0.93 (12.3) 
 18.6% 
 0.17 
 
 
 verynegative_narrow 
 student_t(3, -3, 1) 
 -0.34 
 [-0.77, 0.09] 
 0.89 (8.4) 
 0.96 (27.0) 
 14.3% 
 0.13 
 
 
 positive_default 
 student_t(3, 1, 3) 
 -0.23 
 [-0.67, 0.16] 
 0.82 (4.6) 
 0.9 (9.1) 
 22.4% 
 0.20 
 
 
 positive_narrow 
 student_t(3, 1, 1) 
 -0.15 
 [-0.55, 0.27] 
 0.72 (2.6) 
 0.87 (6.5) 
 28.6% 
 0.25 
 
 
 verypositive_default 
 student_t(3, 3, 3) 
 -0.22 
 [-0.62, 0.21] 
 0.79 (3.9) 
 0.94 (15.3) 
 23.0% 
 0.20 
 
 
 verypositive_narrow 
 student_t(3, 3, 1) 
 -0.14 
 [-0.57, 0.27] 
 0.7 (2.3) 
 0.98 (58.0) 
 30.0% 
 0.27 
 
 
 informative 
 student_t(3, -0.2, 3) 
 -0.26 
 [-0.66, 0.14] 
 0.85 (5.6) 
 0.89 (7.8) 
 20.2% 
 0.18 
 
 
 
 
 
  MCPH1 -D 
 All models show good mixing and convergence (not shown). 
 
 Comparing the posterior distribution for  MCPH1 -D frequency (z-scored) for various prior distributions. The HDI is a 89%HDI;  p ( β <0) and  p ( β =0) show the posterior probability (and the evidence ratio, in parantheses); the ROPE is [-0.10, 0.10]. 
 
 
 
 
 
 
 
 
 
 
 
 
 Prior name 
 Prior distribution 
  β  
 HDI 
  p ( β <0) 
  p ( β =0) 
 %HDI in ROPE 
  p  ROPE  
 
 
 
 
 default 
 student_t(3, 0, 3) 
 -0.23 
 [-0.70, 0.20] 
 0.79 (3.7) 
 0.89 (7.9) 
 21.8% 
 0.19 
 
 
 flat 
 normal(0, 10) 
 -0.24 
 [-0.66, 0.24] 
 0.81 (4.3) 
 0.96 (22.7) 
 20.4% 
 0.18 
 
 
 default_normal 
 normal(0, 5) 
 -0.24 
 [-0.68, 0.24] 
 0.8 (4.0) 
 0.92 (11.4) 
 19.6% 
 0.17 
 
 
 narrow_0 
 student_t(3, 0, 1) 
 -0.21 
 [-0.65, 0.21] 
 0.79 (3.7) 
 0.72 (2.6) 
 21.9% 
 0.20 
 
 
 verynarrow_0 
 student_t(3, 0, 0.1) 
 -0.04 
 [-0.22, 0.16] 
 0.62 (1.6) 
 0.53 (1.1) 
 70.3% 
 0.63 
 
 
 negative_default 
 student_t(3, -1, 3) 
 -0.24 
 [-0.69, 0.22] 
 0.81 (4.3) 
 0.89 (7.8) 
 19.5% 
 0.17 
 
 
 negative_narrow 
 student_t(3, -1, 1) 
 -0.30 
 [-0.73, 0.11] 
 0.87 (6.8) 
 0.79 (3.8) 
 16.6% 
 0.15 
 
 
 verynegative_default 
 student_t(3, -3, 3) 
 -0.25 
 [-0.71, 0.19] 
 0.81 (4.4) 
 0.93 (12.7) 
 20.2% 
 0.18 
 
 
 verynegative_narrow 
 student_t(3, -3, 1) 
 -0.31 
 [-0.77, 0.14] 
 0.86 (5.9) 
 0.97 (31.5) 
 15.4% 
 0.14 
 
 
 positive_default 
 student_t(3, 1, 3) 
 -0.22 
 [-0.66, 0.23] 
 0.79 (3.8) 
 0.9 (8.8) 
 21.3% 
 0.19 
 
 
 positive_narrow 
 student_t(3, 1, 1) 
 -0.13 
 [-0.59, 0.32] 
 0.67 (2.1) 
 0.85 (5.6) 
 27.5% 
 0.24 
 
 
 verypositive_default 
 student_t(3, 3, 3) 
 -0.20 
 [-0.65, 0.25] 
 0.77 (3.3) 
 0.93 (13.8) 
 22.3% 
 0.20 
 
 
 verypositive_narrow 
 student_t(3, 3, 1) 
 -0.12 
 [-0.59, 0.37] 
 0.66 (1.9) 
 0.98 (52.4) 
 25.9% 
 0.23 
 
 
 informative 
 student_t(3, -0.2, 3) 
 -0.24 
 [-0.71, 0.17] 
 0.82 (4.5) 
 0.89 (7.9) 
 20.6% 
 0.18 
 
 
 
 
 
 
 
 Appendix III: Excluding the “proxy” SNPs 
 Here I conduct some of the analyses using only the actual “derived” loci for the two genes (i.e., excluding all the “proxy” SNPs used in the full analysis). 
 
  tone1  
 There are 108 observations, distributed among 75 unique Glottolg codes in 29 families (ranging from a minimum of 1 language per family to a maximum of 38, with a mean 3.7 and median 2 languages per family) and 4 macroareas. 
 There are 98:83:75 unique samples:(meta)populations:languages retained. 
 
 
 
 
 
 
 
 
 
 
 
   
 Africa 
 Eurasia 
 America 
 Papunesia 
 Sum 
 
 
 
 
  No  
 6 
 53 
 2 
 3 
 64 
 
 
  Yes  
 19 
 21 
 3 
 1 
 44 
 
 
  Sum  
 25 
 74 
 5 
 4 
 108 
 
 
 
 
 
 
  Figure 134.    Distribution of  tone1 . 
 
 
 
 
 
  Figure 135.    Map of  tone1 . 
 
 
 
 
 
  Figure 136.    Relationship between  tone1 ,  ASPM -D and  MCPH1 -D. 
 
 
 
 Regressions 
 
  glmer  
 
 All data 
 
  null model : R 2  = 0.0%, ICC = 53.2% 
  macroarea :  p  macroarea/null  = 0.046 
  ASPM :
 
 by itself: R 2  = 6.8%,  β  = -0.68 ± 0.38,  p  ASPM/null  = 0.065 
 quadratic: R 2  = 7.0%,  β ASPM2   = -0.61 ± 0.43,  p  ASPM2/ASPM  = 0.71 
 with  macroarea : R 2  = 18.2%,  p  macroarea/ASPM  = 0.19,  p  ASPM/macroarea  = 0.64 
  
  MCPH1 :
 
 by itself: R 2  = 8.5%,  β  = -0.79 ± 0.40,  p  MCPH1/null  = 0.042 
 quadratic: R 2  = 9.5%,  β MCPH12   = -0.74 ± 0.39,  p  MCPH12/MCPH1  = 0.28 
 with  macroarea : R 2  = 18.2%,  p  macroarea/MCPH1  = 0.26,  p  MCPH1/macroarea  = 0.68 
  
  both alleles  (no  macroarea ):
 
  ASPM  +  MCPH1 : R 2  = 11.0%,  β  ASPM  = -0.45 ± 0.40,  p  ASPM/MCPH1  = 0.26,  β  MCPH1  = -0.57 ± 0.42,  p  MCPH1/ASPM  = 0.16,  p  ASPM+MCPH1/null  = 0.068, 
 interaction: R 2  = 11.4%,  p  ASPM:MCPH1/ASPM+MCPH1  = 0.62 
  
 
 
 Alleles on macroarea 
 To better understand this overlap between family, macroarea and the two “derived” alleles, I regressed (separately) the  ASPM -D and  MCPH1 -D on the  macroarea , using mixed-effects  beta regression  (after replacing all  \(0.0\)  values by  \(10^{-7}\)  and all  \(1.0\)  by  \(1.0-10^{-7}\) , respectively) with language family as random effect: 
 
 the alleles are very strongly clustered within  families :
 
  ASPM : ICC = 100.0% 
  MCPH1 : ICC = 100.0% 
  
  macroarea  predicts their distribution very strongly:
 
  ASPM :  p  = 3.3e-05, R 2  = 39.1% 
  MCPH1 :  p  = 2.3e-09, R 2  = 74.1% 
  
 separating Africa vs the rest of the world seems to drive most of this effect (both alleles have lower frequencies in Africa):
 
  ASPM :  p  = 8.1e-05, R 2  = 24.1% 
  MCPH1 :  p  = 1.5e-07, R 2  = 49.5% 
  
 
 
 
 
 Randomization 
 I performed 1000 independent replications of each of these parameter combinations, and below are the distributions of the permuted values versus the original ones (i.e., those obtained on the original, non-permuted data). 
 
 Regressions on 1000 permuted data. The first 3 columns show the permutation constraints (if any), how the  macroarea  is considered (if at all), and what is permuted. The next columns show the percent of the permutations that, in order, have a better AIC compared to the original fit, are significantly better than the null model (thus testing the effect of both alleles simultaneously), have a significant effect of  ASPM -D, have a smaller effect ( β ) of  ASPM -D than the original fit, and the same for  MCPH1 -D. 
 
 
 Permute within 
 Macroarea 
 Permute 
 AIC 
 Signif. 
  p   ASPM -D  
  β   ASPM -D  
  p   MCPH1 -D  
  β   MCPH1 -D  
 
 
 
 
 unrestricted 
 none 
 tone 
 0% 
 5% 
 4% 
 3% 
 5% 
 1% 
 
 
 unrestricted 
 none 
 alleles-together 
 8% 
 6% 
 6% 
 9% 
 6% 
 5% 
 
 
 unrestricted 
 none 
 alleles-independent 
 8% 
 5% 
 6% 
 9% 
 5% 
 2% 
 
 
 unrestricted 
 fixef 
 tone 
 0% 
 7% 
 7% 
 29% 
 5% 
 33% 
 
 
 unrestricted 
 fixef 
 alleles-together 
 89% 
 6% 
 6% 
 34% 
 6% 
 28% 
 
 
 unrestricted 
 fixef 
 alleles-independent 
 86% 
 5% 
 5% 
 30% 
 5% 
 28% 
 
 
 macroareas 
 none 
 tone 
 0% 
 82% 
 14% 
 19% 
 71% 
 51% 
 
 
 macroareas 
 none 
 alleles-together 
 39% 
 30% 
 8% 
 27% 
 22% 
 56% 
 
 
 macroareas 
 none 
 alleles-independent 
 43% 
 35% 
 12% 
 34% 
 25% 
 59% 
 
 
 macroareas 
 fixef 
 tone 
 0% 
 5% 
 4% 
 26% 
 5% 
 33% 
 
 
 macroareas 
 fixef 
 alleles-together 
 86% 
 5% 
 5% 
 32% 
 5% 
 38% 
 
 
 macroareas 
 fixef 
 alleles-independent 
 86% 
 4% 
 5% 
 36% 
 4% 
 38% 
 
 
 families 
 none 
 tone 
 20% 
 13% 
 2% 
 10% 
 17% 
 46% 
 
 
 families 
 none 
 alleles-together 
 9% 
 6% 
 2% 
 16% 
 4% 
 21% 
 
 
 families 
 none 
 alleles-independent 
 13% 
 9% 
 7% 
 25% 
 8% 
 26% 
 
 
 families 
 fixef 
 tone 
 20% 
 6% 
 2% 
 33% 
 7% 
 65% 
 
 
 families 
 fixef 
 alleles-together 
 79% 
 1% 
 2% 
 35% 
 1% 
 19% 
 
 
 families 
 fixef 
 alleles-independent 
 84% 
 2% 
 3% 
 32% 
 1% 
 19% 
 
 
 
 
 
 
Regressions on 1000 permuted data. Each plot shows the original result (vertical dashed black line) and the distribution of the permutations for the three possible things to be permuted (colored curves) for each combination of permutation constraints (horizontal panels) and control for  macroarea  (vertical panels) in terms of the effect size  β ;  ASPM -D is on the left and  MCPH1 -D on the right. The vertical dotted black thin line is at 0.0.
 
 
 
 
 Restricted sampling 
 
 
 
  Figure 137.    Results for 1000 restricted samplings. For  ASPM -D (left): 98.2% of βs are negative when regressing tone on  ASPM  alone (one-sided  t -test < 0:  t (999) = -56.0, mean = -0.55,  p  = 1.2e-310), 56.3%, when controlling for the macroarea ( t (999) = -7.6, mean = -0.10,  p  = 4e-14), and 54.3% when controlling for both macroarea and  MCPH1  ( t (999) = -7.3, mean = -0.10,  p  = 2.2e-13). For  MCPH1 -D (right): 100% of βs are negative when regressing tone on  MCPH1  alone (one-sided  t -test < 0:  t (999) = -88.1, mean = -0.54,  p  = 0), 58.4% when controlling for the macroarea ( t (999) = -7.1, mean = -0.15,  p  = 9.9e-13), and 60% when controlling for both macroarea and  ASPM  ( t (999) = -8.8, mean = -0.19,  p  = 3.8e-18). 
 
 
 
 
 
  brms  
 
  ASPM  only:
 
  β  = -0.53, 89%HDI = [-1.46, 0.31] 
 posterior probability  p ( β <0) = 0.83 (evidence ratio = 5),  p ( β =0) = 0.8 (evidence ratio = 4.1) 
 ROPE = [-0.18, 0.18], % HDI inside ROPE = 18.8%;  p  ROPE  = 0.168 
 comparison ‘null’ vs ‘ASPM’: [B> L= W=(61%:39%) K>]: moderate evidence for null against ASPM (BF=3.2), LOO=0.42 [SE=1.20], WAIC=0.46 [SE=1.07], KFOLD=1.90 [SE=1.88] 
 comparison ‘null’ vs ‘ASPM’: [B> L= W=(61%:39%) K>]: moderate evidence for null against ASPM (BF=3.2), LOO=0.42 [SE=1.20], WAIC=0.46 [SE=1.07], KFOLD=1.90 [SE=1.88] 
  
  MCPH1  only:
 
  β  = -0.7, 89%HDI = [-1.91, 0.43] 
 posterior probability  p ( β <0) = 0.84 (evidence ratio = 5.2),  p ( β =0) = 0.75 (evidence ratio = 3.1) 
 ROPE = [-0.18, 0.18], % HDI inside ROPE = 13.2%;  p  ROPE  = 0.117 
 comparison ‘null’ vs ‘MCPH1’: [B= L= W=(52%:48%) K>]: anecdotal evidence for null against MCPH1 (BF=2.89), LOO=0.27 [SE=0.87], WAIC=0.07 [SE=0.75], KFOLD=1.68 [SE=1.60] 
 comparison ‘null’ vs ‘MCPH1’: [B= L= W=(52%:48%) K>]: anecdotal evidence for null against MCPH1 (BF=2.89), LOO=0.27 [SE=0.87], WAIC=0.07 [SE=0.75], KFOLD=1.68 [SE=1.60] 
  
  both alleles :
 
 comparison ‘null’ vs ‘both’: [B>> L> W=(69%:31%) K>]: strong evidence for null against both (BF=15.6), LOO=1.80 [SE=1.38], WAIC=0.82 [SE=1.28], KFOLD=2.78 [SE=1.76] 
 interaction:
 
 posterior probability  p (=0) = 0.77 (evidence ratio = 3.3) 
 ROPE = [-0.18, 0.18], % HDI inside ROPE = 14.9%;  p  ROPE  = 0.132 
 comparison ‘no interaction’ vs ‘with interaction’: [B> L= W=(44%:56%) K>>]: moderate evidence for no interaction against with interaction (BF=3.55), LOO=0.25 [SE=0.82], WAIC=-0.25 [SE=0.68], KFOLD=3.69 [SE=1.62] 
  
  ASPM  (partial):
 
  β  = -0.41, 89%HDI = [-1.33, 0.55] 
 posterior probability  p ( β <0) = 0.76 (evidence ratio = 3.1),  p ( β =0) = 0.82 (evidence ratio = 4.5) 
 ROPE = [-0.18, 0.18], % HDI inside ROPE = 22.1%;  p  ROPE  = 0.197 
  
  MCPH1  (partial):
 
  β  = -0.6, 89%HDI = [-1.74, 0.63] 
 posterior probability  p ( β <0) = 0.8 (evidence ratio = 4),  p ( β =0) = 0.77 (evidence ratio = 3.3) 
 ROPE = [-0.18, 0.18], % HDI inside ROPE = 15.7%;  p  ROPE  = 0.14 
  
  
 
 
  
 
  Figure 138.    Posterior distributions (with 50% probability mass highlighted) versus 0.0 (the vertical line) for  ASPM -D (left) and  MCPH1 -D (right). 
 
 
 
  
 
  Figure 139.    Conditional effects of  ASPM -D (left) and  MCPH1 -D (right). 
 
 
 
  
 
  Figure 140.    Posterior predictive checks for  ASPM -D (left) and  MCPH1 -D (right). 
 
 
 
  
 
  Figure 141.    Confusion matrices for  ASPM -D (left) and  MCPH1 -D (right). 
 
 
 
 
 
 Mediation and path analysis 
 
 Mediation analysis 
 
  (g)lm  
 
 All data 
 For  ASPM -D: 
 
   total effect  (TE) of being in Africa on tone: 0.44 (0.24, 0.61),  p =0, decomposed into:  
   average direct effect  (ADE): 0.28 (0.04, 0.51),  p =0.02, and  
   average indirect effect  (ACME) mediated by  ASPM -D: 0.16 (0.03, 0.30),  p =0.022, mediating 35.7% (5.6%, 85.0%),  p =0.022 of the effect, resulting from: 
 
 effect of being in Africa on  ASPM -D: -1.22 ±0.20,  p =1.2e-08, and 
 effect of  ASPM -D on tone: -0.63 ±0.27,  p =0.022. 
  
 
 For  MCPH1 -D: 
 
   TE : 0.44 (0.24, 0.61),  p =0, decomposed into:  
   ADE : 0.53 (0.10, 0.73),  p =0.012, and  
   ACME : -0.09 (-0.29, 0.28),  p =0.43, mediating -25.9% (-104.5%, 70.5%),  p =0.43 of the effect, resulting from: 
 
 effect of being in Africa on  MCPH1 -D: -2.08 ±0.11,  p =1.7e-36, and 
 effect of  MCPH1 -D on tone: 0.38 ±0.48,  p =0.42. 
  
 
 
 
 Restricted sampling 
 
 
 
  Figure 142.    Mediation analysis for 1000 restricted samples (i.e., picking one random language per family). The leftmost panels show the distribution of point estimates of the Total Effect (TE), the Direct Effect (ADE) and the Indirect Effect (ACME) for  ASPM -D and  MCPH1 -D; the middle panels show the distribution of the  p -values for the same effects, while the rightmost panels show the distribution of the regression slopes ( β ) for the two alleles, top: for the regression of the allele frequency on within vs outside Africa, and bottom: for the regression of tone on the allele while controlling for within vs outside Africa. The black vertical lines show: 0.0 (solid), 0.05 (dashed) and 0.10 (dotted). 
 
 
 For  ASPM -D: 
 
   TE : mean = 0.32, median = 0.33; 15.6% significant at  α -level 0.05 and 49.1% significant at  α -level 0.10; 99.9% > 0.0; one-sample one-sided t-test vs 0:  t (999) = 115.5,  p  = 0;  
   ADE : mean = 0.28, median = 0.28; 6.4% significant at  α -level 0.05 and 24.7% significant at  α -level 0.10; 99.7% > 0.0; one-sample one-sided t-test vs 0:  t (999) = 87.8,  p  = 0;  
   ACME : mean = 0.046, median = 0.044; 0.0% significant at  α -level 0.05 and 1.8% significant at  α -level 0.10; 79.3% > 0.0; one-sample one-sided t-test vs 0:  t (999) = 30.0,  p  = 1.1e-141;  
   β(Africa → allele) : mean = -0.82, median = -0.84; 69.4% significant at  α -level 0.05 and 89.8% significant at  α -level 0.10; 100.0% < 0.0; one-sample one-sided t-test vs 0:  t (999) = -228.0,  p  = 0;  
   β(allele → tone | Africa) : mean = -0.31, median = -0.29; 0.1% significant at  α -level 0.05 and 4.3% significant at  α -level 0.10; 78.7% < 0.0; one-sample one-sided t-test vs 0:  t (999) = -28.3,  p  = 5.8e-130.  
 
 For  MCPH1 -D: 
 
   TE : mean = 0.33, median = 0.33; 15.8% significant at  α -level 0.05 and 50.0% significant at  α -level 0.10; 99.9% > 0.0; one-sample one-sided t-test vs 0:  t (999) = 116.1,  p  = 0;  
   ADE : mean = 0.35, median = 0.39; 2.1% significant at  α -level 0.05 and 12.3% significant at  α -level 0.10; 95.6% > 0.0; one-sample one-sided t-test vs 0:  t (999) = 65.5,  p  = 0;  
   ACME : mean = -0.029, median = -0.048; 0.1% significant at  α -level 0.05 and 0.8% significant at  α -level 0.10; 38.0% > 0.0; one-sample one-sided t-test vs 0:  t (999) = -6.5,  p  = 1;  
   β(Africa → allele) : mean = -2.3, median = -2.3; 100.0% significant at  α -level 0.05 and 100.0% significant at  α -level 0.10; 100.0% < 0.0; one-sample one-sided t-test vs 0:  t (999) = -953.8,  p  = 0;  
   β(allele → tone | Africa) : mean = 0.34, median = 0.33; 0.2% significant at  α -level 0.05 and 0.5% significant at  α -level 0.10; 27.6% < 0.0; one-sample one-sided t-test vs 0:  t (999) = 18.9,  p  = 1.  
 
 Given the low sample size  N  = 29 unique families, relatively few effect sizes are big enough to be significant for each individual analysis; however, there are many more significant ACMEs for  ASPM -D than for  MCPH1 -D: 0.1% vs 0.2% (0.5 times) for  α -level 0.05, and 4.3% vs 0.5% (8.6 times) for  α -level 0.10. 
 
 
 
  brms  
 
  
 
 
  Figure 143.    Graphical representation of the Bayesian mediation analysis for  ASPM -D showing the means of the effects and the actual partial regression coefficients, with their 89% HDIs and p-ROPEs. The colors reflect the sign of the mean estimate (blue=negative, red=positive, gray=(p-ROPE >= 0.05)); solid=(0 not in the HDI), dashed=(0 is in the HDI). 
 
 
 
  
 
 
  Figure 144.    Graphical representation of the Bayesian mediation analysis for  MCPH1 -D showing the means of the effects and the actual partial regression coefficients, with their 89% HDIs and p-ROPEs. The colors reflect the sign of the mean estimate (blue=negative, red=positive, gray=(p-ROPE >= 0.05)); solid=(0 not in the HDI), dashed=(0 is in the HDI). 
 
 
 
  
 
 
  Figure 145.    Graphical representation of the Bayesian mediation analysis for both  ASPM -D and  MCPH1 -D showing the means of the effects and the actual partial regression coefficients, with their 89% HDIs and p-ROPEs. The colors reflect the sign of the mean estimate (blue=negative, red=positive, gray=(p-ROPE >= 0.05)); solid=(0 not in the HDI), dashed=(0 is in the HDI). 
 
 
 
 
 
 Path analysis 
 
 All data 
 With Africa and  tone1  coded numerically, the model fit is:  χ  2 (1)=0.00,  p =0.96; CFI=1.00, TLI=1.03, NNFI=1.03 and RFI=1.00: 
 
  
 
 
  Figure 146.    Path analysis model with standardised coefficients and significance stars.  tone1  and  macroarea  (Africa vs non-Africa) are coded as numeric binary ( tone_bin_num  with Yes=1 and  Africa_num  with in Africa=1);  ASPM_z  is  ASPM -D and  MCPH1_z  is  MCPH1 -D. 
 
 
 Likewise, with Africa and  tone1  coded as ordered binary factors, the model fit is:  χ  2 (1)=0.01,  p =0.94; CFI=1.00, TLI=1.66, NNFI=1.66 and RFI=1.00: 
 
  
 
 
  Figure 147.    Path analysis model with standardised coefficients and significance stars.  tone1  and  macroarea  (Africa vs non-Africa) are coded as ordered binary factors ( tone_bin_ord  with No < Yes, and  Africa_ord  with outside Africa < in Africa);  ASPM_z  is  ASPM -D and  MCPH1_z  is  MCPH1 -D. 
 
 
 
 
 Restricted sampling 
 
 
 
  Figure 148.    Path analysis for 1000 restricted samples (i.e., picking one random language per family). The leftmost row of two plots shows the coefficient estimates and the  p -values, respectively, for the five paths in the model (see the path plots above). The rightmost plot shows the various fit indices. The black horiontal lines show: 0.0 (solid), 0.05 (dashed) and 1.0 (dotted). 
 
 
 
  models fits: 
 
 100% of the  p -values are not significant 
 mean(CFI) = 1, median(CFI) = 1, sd(CFI) = 0.01, IQR(CFI) = 0 
 mean(TLI) = 1.04, median(TLI) = 1.06, sd(TLI) = 0.07, IQR(TLI) = 0.08 
 mean(NNFI) = 1.04, median(NNFI) = 1.06, sd(NNFI) = 0.07, IQR(NNFI) = 0.08 
 mean(RFI) = 0.94, median(RFI) = 0.96, sd(RFI) = 0.06, IQR(RFI) = 0.08 
  
  Africa →  ASPM -D: mean = -0.83, median = -0.85, sd = 0.11, IQR = 0.14, 100.0% < 0; 95.6% significant at  α -level 0.05; one-sample one-sided  t -test vs 0:  t (999) = -2.4e+02,  p  = 0;  
  Africa →  MCPH1 -D: mean = -2.3, median = -2.3, sd = 0.077, IQR = 0.099, 100.0% < 0; 100.0% significant at  α -level 0.05; one-sample one-sided  t -test vs 0:  t (999) = -9.5e+02,  p  = 0;  
  Africa →  tone1 : mean = 0.45, median = 0.47, sd = 0.3, IQR = 0.44, 92.2% > 0; 12.4% significant at  α -level 0.05; one-sample one-sided  t -test vs 0:  t (999) = 47,  p  = 5.6e-253;  
   ASPM -D →  tone1 : mean = -0.057, median = -0.057, sd = 0.071, IQR = 0.11, 76.0% < 0; 10.8% significant at  α -level 0.05; one-sample one-sided  t -test vs 0:  t (999) = -25,  p  = 2.3e-110;  
   MCPH1 -D →  tone1 : mean = 0.057, median = 0.064, as = 0.11, IQR = 0.16, 30.4% < 0; 0.9% significant at  α -level 0.05; one-sample one-sided  t -test vs 0:  t (999) = 16,  p  = 1.  
 
 
 
 
 
 
  tone2  
 The resulting dataset has 106 observations, distributed among 73 unique Glottolg codes in 29 families (ranging from a minimum of 1 language per family to a maximum of 37, with a mean 3.7 and median 2 languages per family) and 4 macroareas. 
 There are 93:78:73 unique samples:(meta)populations:languages retained. 
 
 
 
 
 
 
 
 
 
 
 
   
 Africa 
 Eurasia 
 America 
 Papunesia 
 Sum 
 
 
 
 
  No  
 17 
 58 
 5 
 4 
 84 
 
 
  Yes  
 9 
 13 
 0 
 0 
 22 
 
 
  Sum  
 26 
 71 
 5 
 4 
 106 
 
 
 
 
 
 
  Figure 149.    Distribution of  tone2 . 
 
 
 
 
 
  Figure 150.    Map of  tone2 . 
 
 
 
 
 
  Figure 151.    Relationship between  tone2 ,  ASPM -D and  MCPH1 -D. 
 
 
 
 Regressions 
 
  glmer  
 
 All data 
 
  null model : R 2  = 0.0%, ICC = 94.9% 
  macroarea :  p  macroarea/null  = 0.21 
  ASPM :
 
 by itself: R 2  = 3.7%,  β  = -1.78 ± 1.25,  p  ASPM/null  = 0.072 
 quadratic: R 2  = 50.0%,  β ASPM2   = -8.21 ± 6.07,  p  ASPM2/ASPM  = 0.0044 
 with  macroarea : R 2  = 52.7%,  p  macroarea/ASPM  = 0.52,  p  ASPM/macroarea  = 0.33 
  
  MCPH1 :
 
 by itself: R 2  = 3.3%,  β  = -1.30 ± 0.94,  p  MCPH1/null  = 0.13 
 quadratic: R 2  = 24.3%,  β MCPH12   = -1.39 ± 0.93,  p  MCPH12/MCPH1  = 0.06 
 with  macroarea : R 2  = 47.0%,  p  macroarea/MCPH1  = 0.43,  p  MCPH1/macroarea  = 0.52 
  
  both alleles  (no  macroarea ):
 
  ASPM  +  MCPH1 : R 2  = 5.1%,  β  ASPM  = -1.38 ± 1.32,  p  ASPM/MCPH1  = 0.22,  β  MCPH1  = -0.72 ± 1.03,  p  MCPH1/ASPM  = 0.48,  p  ASPM+MCPH1/null  = 0.15, 
 interaction: R 2  = 6.9%,  p  ASPM:MCPH1/ASPM+MCPH1  = 0.69 
  
 
 
 
 Randomization 
 I performed 1000 independent replications of each of these parameter combinations, and below are the distributions of the permuted values versus the original ones (i.e., those obtained on the original, non-permuted data). 
 
 Regressions on 1000 permuted data. The first 3 columns show the permutation constraints (if any), how the  macroarea  is considered (if at all), and what is permuted. The next columns show the percent of the permutations that, in order, have a better AIC compared to the original fit, are significantly better than the null model (thus testing the effect of both alleles simultaneously), have a significant effect of  ASPM -D, have a smaller effect ( β ) of  ASPM -D than the original fit, and the same for  MCPH1 -D. 
 
 
 Permute within 
 Macroarea 
 Permute 
 AIC 
 Signif. 
  p   ASPM -D  
  β   ASPM -D  
  p   MCPH1 -D  
  β   MCPH1 -D  
 
 
 
 
 unrestricted 
 none 
 tone 
 0% 
 5% 
 6% 
 0% 
 6% 
 1% 
 
 
 unrestricted 
 none 
 alleles-together 
 20% 
 9% 
 8% 
 3% 
 9% 
 13% 
 
 
 unrestricted 
 none 
 alleles-independent 
 23% 
 9% 
 8% 
 2% 
 8% 
 9% 
 
 
 unrestricted 
 fixef 
 tone 
 0% 
 6% 
 6% 
 0% 
 6% 
 91% 
 
 
 unrestricted 
 fixef 
 alleles-together 
 60% 
 9% 
 9% 
 5% 
 8% 
 92% 
 
 
 unrestricted 
 fixef 
 alleles-independent 
 62% 
 9% 
 7% 
 3% 
 9% 
 94% 
 
 
 macroareas 
 none 
 tone 
 0% 
 19% 
 3% 
 0% 
 28% 
 13% 
 
 
 macroareas 
 none 
 alleles-together 
 46% 
 19% 
 4% 
 10% 
 24% 
 78% 
 
 
 macroareas 
 none 
 alleles-independent 
 48% 
 23% 
 8% 
 15% 
 25% 
 79% 
 
 
 macroareas 
 fixef 
 tone 
 0% 
 6% 
 6% 
 0% 
 5% 
 90% 
 
 
 macroareas 
 fixef 
 alleles-together 
 54% 
 6% 
 6% 
 13% 
 7% 
 73% 
 
 
 macroareas 
 fixef 
 alleles-independent 
 55% 
 7% 
 8% 
 15% 
 7% 
 79% 
 
 
 families 
 none 
 tone 
 6% 
 10% 
 5% 
 13% 
 3% 
 26% 
 
 
 families 
 none 
 alleles-together 
 18% 
 8% 
 8% 
 18% 
 0% 
 18% 
 
 
 families 
 none 
 alleles-independent 
 18% 
 8% 
 12% 
 19% 
 3% 
 23% 
 
 
 families 
 fixef 
 tone 
 6% 
 6% 
 9% 
 28% 
 3% 
 90% 
 
 
 families 
 fixef 
 alleles-together 
 70% 
 8% 
 6% 
 25% 
 10% 
 31% 
 
 
 families 
 fixef 
 alleles-independent 
 66% 
 10% 
 8% 
 17% 
 11% 
 35% 
 
 
 
 
 
 
Regressions on 1000 permuted data. Each plot shows the original result (vertical dashed black line) and the distribution of the permutations for the three possible things to be permuted (colored curves) for each combination of permutation constraints (horizontal panels) and control for  macroarea  (vertical panels) in terms of the effect size  β ;  ASPM -D is on the left and  MCPH1 -D on the right. The vertical dotted black thin line is at 0.0.
 
 
 
 
 Restricted sampling 
 
 
 
  Figure 152.    Results for 1000 restricted samplings. For  ASPM -D (left): 87.8% of βs are negative when regressing tone on  ASPM  alone (one-sided  t -test < 0:  t (999) = -38.2, mean = -0.27,  p  = 5.6e-198), 93%, when controlling for the macroarea ( t (999) = -45.5, mean = -0.48,  p  = 9.2e-246), and 94.3% when controlling for both macroarea and  MCPH1  ( t (999) = -44.1, mean = -0.60,  p  = 9.5e-237). For  MCPH1 -D (right): 82.5% of βs are negative when regressing tone on  MCPH1  alone (one-sided  t -test < 0:  t (999) = -35.5, mean = -0.29,  p  = 2.7e-179), 13.3% when controlling for the macroarea ( t (999) = 32.3, mean = 0.86,  p  = 1), and 12.6% when controlling for both macroarea and  ASPM  ( t (999) = 30.9, mean = 1.08,  p  = 1). 
 
 
 
 
 
  brms  
 
  ASPM  only:
 
  β  = -1.33, 89%HDI = [-2.91, 0.32] 
 posterior probability  p ( β <0) = 0.92 (evidence ratio = 11),  p ( β =0) = 0.57 (evidence ratio = 1.3) 
 ROPE = [-0.18, 0.18], % HDI inside ROPE = 6.9%;  p  ROPE  = 0.061 
 comparison ‘null’ vs ‘ASPM’: [B= L= W=(45%:55%) K=]: anecdotal evidence for null against ASPM (BF=1.26), LOO=0.33 [SE=1.05], WAIC=-0.20 [SE=0.94], KFOLD=-0.02 [SE=2.27] 
 comparison ‘null’ vs ‘ASPM’: [B= L= W=(45%:55%) K=]: anecdotal evidence for null against ASPM (BF=1.26), LOO=0.33 [SE=1.05], WAIC=-0.20 [SE=0.94], KFOLD=-0.02 [SE=2.27] 
  
  MCPH1  only:
 
  β  = -0.9, 89%HDI = [-2.89, 0.76] 
 posterior probability  p ( β <0) = 0.8 (evidence ratio = 3.9),  p ( β =0) = 0.66 (evidence ratio = 2) 
 ROPE = [-0.18, 0.18], % HDI inside ROPE = 10.1%;  p  ROPE  = 0.09 
 comparison ‘null’ vs ‘MCPH1’: [B= L= W<(37%:63%) K=]: anecdotal evidence for null against MCPH1 (BF=1.69), LOO=0.55 [SE=0.68], WAIC=-0.51 [SE=0.47], KFOLD=-0.29 [SE=2.45] 
 comparison ‘null’ vs ‘MCPH1’: [B= L= W<(37%:63%) K=]: anecdotal evidence for null against MCPH1 (BF=1.69), LOO=0.55 [SE=0.68], WAIC=-0.51 [SE=0.47], KFOLD=-0.29 [SE=2.45] 
  
  both alleles :
 
 comparison ‘null’ vs ‘both’: [B> L= W=(30%:70%) K=]: moderate evidence for null against both (BF=3.62), LOO=-0.09 [SE=1.10], WAIC=-0.86 [SE=1.02], KFOLD=0.92 [SE=1.18] 
 interaction:
 
 posterior probability  p (=0) = 0.7 (evidence ratio = 2.4) 
 ROPE = [-0.18, 0.18], % HDI inside ROPE = 11.7%;  p  ROPE  = 0.104 
 comparison ‘no interaction’ vs ‘with interaction’: [B= L> W=(58%:42%) K>]: anecdotal evidence for no interaction against with interaction (BF=2.54), LOO=1.33 [SE=0.81], WAIC=0.34 [SE=0.41], KFOLD=3.94 [SE=2.20] 
  
  ASPM  (partial):
 
  β  = -1.22, 89%HDI = [-2.99, 0.53] 
 posterior probability  p ( β <0) = 0.88 (evidence ratio = 7),  p ( β =0) = 0.64 (evidence ratio = 1.7) 
 ROPE = [-0.18, 0.18], % HDI inside ROPE = 8.6%;  p  ROPE  = 0.077 
  
  MCPH1  (partial):
 
  β  = -0.59, 89%HDI = [-2.52, 1.43] 
 posterior probability  p ( β <0) = 0.7 (evidence ratio = 2.3),  p ( β =0) = 0.72 (evidence ratio = 2.5) 
 ROPE = [-0.18, 0.18], % HDI inside ROPE = 12.7%;  p  ROPE  = 0.114 
  
  
 
 
  
 
  Figure 153.    Posterior distributions (with 50% probability mass highlighted) versus 0.0 (the vertical line) for  ASPM -D (left) and  MCPH1 -D (right). 
 
 
 
  
 
  Figure 154.    Conditional effects of  ASPM -D (left) and  MCPH1 -D (right). 
 
 
 
  
 
  Figure 155.    Posterior predictive checks for  ASPM -D (left) and  MCPH1 -D (right). 
 
 
 
  
 
  Figure 156.    Confusion matrices for  ASPM -D (left) and  MCPH1 -D (right). 
 
 
 
 
 
 Mediation and path analysis 
 
 Mediation analysis 
 
  (g)lm  
 
 All data 
 For  ASPM -D: 
 
   total effect  (TE) of being in Africa on tone: 0.20 (0.01, 0.40),  p =0.036, decomposed into:  
   average direct effect  (ADE): 0.01 (-0.18, 0.25),  p =0.96, and  
   average indirect effect  (ACME) mediated by  ASPM -D: 0.19 (0.02, 0.34),  p =0.024, mediating 95.9% (-1.7%, 470.4%),  p =0.052 of the effect, resulting from: 
 
 effect of being in Africa on  ASPM -D: -1.34 ±0.18,  p =7.4e-11, and 
 effect of  ASPM -D on tone: -0.86 ±0.39,  p =0.028. 
  
 
 For  MCPH1 -D: 
 
   TE : 0.18 (-0.01, 0.39),  p =0.064, decomposed into:  
   ADE : 0.30 (-0.15, 0.63),  p =0.17, and  
   ACME : -0.12 (-0.39, 0.28),  p =0.46, mediating -72.3% (-674.6%, 386.6%),  p =0.5 of the effect, resulting from: 
 
 effect of being in Africa on  MCPH1 -D: -2.08 ±0.10,  p =7.6e-39, and 
 effect of  MCPH1 -D on tone: 0.47 ±0.63,  p =0.46. 
  
 
 
 
 Restricted sampling 
 
 
 
  Figure 157.    Mediation analysis for 1000 restricted samples (i.e., picking one random language per family). The leftmost panels show the distribution of point estimates of the Total Effect (TE), the Direct Effect (ADE) and the Indirect Effect (ACME) for  ASPM -D and  MCPH1 -D; the middle panels show the distribution of the  p -values for the same effects, while the rightmost panels show the distribution of the regression slopes ( β ) for the two alleles, top: for the regression of the allele frequency on within vs outside Africa, and bottom: for the regression of tone on the allele while controlling for within vs outside Africa. The black vertical lines show: 0.0 (solid), 0.05 (dashed) and 0.10 (dotted). 
 
 
 For  ASPM -D: 
 
   TE : mean = 0.15, median = 0.15; 0.7% significant at  α -level 0.05 and 4.8% significant at  α -level 0.10; 90.1% > 0.0; one-sample one-sided t-test vs 0:  t (999) = 50.2,  p  = 7.1e-276;  
   ADE : mean = 0.13, median = 0.13; 0.1% significant at  α -level 0.05 and 2.4% significant at  α -level 0.10; 90.7% > 0.0; one-sample one-sided t-test vs 0:  t (999) = 46.1,  p  = 1.3e-249;  
   ACME : mean = 0.016, median = 0.016; 0.0% significant at  α -level 0.05 and 0.0% significant at  α -level 0.10; 71.4% > 0.0; one-sample one-sided t-test vs 0:  t (999) = 19.0,  p  = 5e-69;  
   β(Africa → allele) : mean = -0.88, median = -0.88; 93.2% significant at  α -level 0.05 and 100.0% significant at  α -level 0.10; 100.0% < 0.0; one-sample one-sided t-test vs 0:  t (999) = -433.7,  p  = 0;  
   β(allele → tone | Africa) : mean = -0.097, median = -0.11; 0.0% significant at  α -level 0.05 and 0.0% significant at  α -level 0.10; 69.5% < 0.0; one-sample one-sided t-test vs 0:  t (999) = -14.6,  p  = 3.1e-44.  
 
 For  MCPH1 -D: 
 
   TE : mean = 0.15, median = 0.15; 0.9% significant at  α -level 0.05 and 4.8% significant at  α -level 0.10; 90.1% > 0.0; one-sample one-sided t-test vs 0:  t (999) = 49.6,  p  = 3.8e-272;  
   ADE : mean = 0.12, median = 0.12; 0.0% significant at  α -level 0.05 and 0.1% significant at  α -level 0.10; 81.0% > 0.0; one-sample one-sided t-test vs 0:  t (999) = 28.7,  p  = 7e-133;  
   ACME : mean = 0.024, median = 0.023; 0.0% significant at  α -level 0.05 and 0.0% significant at  α -level 0.10; 55.0% > 0.0; one-sample one-sided t-test vs 0:  t (999) = 5.7,  p  = 7.3e-09;  
   β(Africa → allele) : mean = -2.3, median = -2.3; 100.0% significant at  α -level 0.05 and 100.0% significant at  α -level 0.10; 100.0% < 0.0; one-sample one-sided t-test vs 0:  t (999) = -1219.2,  p  = 0;  
   β(allele → tone | Africa) : mean = 0.033, median = 0.021; 0.0% significant at  α -level 0.05 and 0.0% significant at  α -level 0.10; 48.5% < 0.0; one-sample one-sided t-test vs 0:  t (999) = 1.9,  p  = 0.97.  
 
 Given the low sample size  N  = 29 unique families, relatively few effect sizes are big enough to be significant for each individual analysis; however, there are many more significant ACMEs for  ASPM -D than for  MCPH1 -D: 0.0% vs 0.0% (NaN times) for  α -level 0.05, and 0.0% vs 0.0% (NaN times) for  α -level 0.10. 
 
 
 
  brms  
 
  
 
 
  Figure 158.    Graphical representation of the Bayesian mediation analysis for  ASPM -D showing the means of the effects and the actual partial regression coefficients, with their 89% HDIs and p-ROPEs. The colors reflect the sign of the mean estimate (blue=negative, red=positive, gray=(p-ROPE >= 0.05)); solid=(0 not in the HDI), dashed=(0 is in the HDI). 
 
 
 
  
 
 
  Figure 159.    Graphical representation of the Bayesian mediation analysis for  MCPH1 -D showing the means of the effects and the actual partial regression coefficients, with their 89% HDIs and p-ROPEs. The colors reflect the sign of the mean estimate (blue=negative, red=positive, gray=(p-ROPE >= 0.05)); solid=(0 not in the HDI), dashed=(0 is in the HDI). 
 
 
 
  
 
 
  Figure 160.    Graphical representation of the Bayesian mediation analysis for both  ASPM -D and  MCPH1 -D showing the means of the effects and the actual partial regression coefficients, with their 89% HDIs and p-ROPEs. The colors reflect the sign of the mean estimate (blue=negative, red=positive, gray=(p-ROPE >= 0.05)); solid=(0 not in the HDI), dashed=(0 is in the HDI). 
 
 
 
 
 
 Path analysis 
 
 All data 
 With Africa and  tone1  coded numerically, the model fit is:  χ  2 (1)=0.03,  p =0.86; CFI=1.00, TLI=1.03, NNFI=1.03 and RFI=1.00: 
 
  
 
 
  Figure 161.    Path analysis model with standardised coefficients and significance stars.  tone1  and  macroarea  (Africa vs non-Africa) are coded as numeric binary ( tone_complex_num  with Yes=1 and  Africa_num  with in Africa=1);  ASPM_z  is  ASPM -D and  MCPH1_z  is  MCPH1 -D. 
 
 
 Likewise, with Africa and  tone1  coded as ordered binary factors, the model fit is:  χ  2 (1)=0.07,  p =0.79; CFI=1.00, TLI=1.61, NNFI=1.61 and RFI=0.97: 
 
  
 
 
  Figure 162.    Path analysis model with standardised coefficients and significance stars.  tone1  and  macroarea  (Africa vs non-Africa) are coded as ordered binary factors ( tone_complex_ord  with No < Yes, and  Africa_ord  with outside Africa < in Africa);  ASPM_z  is  ASPM -D and  MCPH1_z  is  MCPH1 -D. 
 
 
 
 
 Restricted sampling 
 
 
 
  Figure 163.    Path analysis for 1000 restricted samples (i.e., picking one random language per family). The leftmost row of two plots shows the coefficient estimates and the  p -values, respectively, for the five paths in the model (see the path plots above). The rightmost plot shows the various fit indices. The black horiontal lines show: 0.0 (solid), 0.05 (dashed) and 1.0 (dotted). 
 
 
 
  models fits: 
 
 100% of the  p -values are not significant 
 mean(CFI) = 1, median(CFI) = 1, sd(CFI) = 0.01, IQR(CFI) = 0 
 mean(TLI) = 1.04, median(TLI) = 1.05, sd(TLI) = 0.06, IQR(TLI) = 0.08 
 mean(NNFI) = 1.04, median(NNFI) = 1.05, sd(NNFI) = 0.06, IQR(NNFI) = 0.08 
 mean(RFI) = 0.94, median(RFI) = 0.95, sd(RFI) = 0.06, IQR(RFI) = 0.07 
  
  Africa →  ASPM -D: mean = -0.88, median = -0.89, sd = 0.062, IQR = 0.084, 100.0% < 0; 100.0% significant at  α -level 0.05; one-sample one-sided  t -test vs 0:  t (999) = -4.5e+02,  p  = 0;  
  Africa →  MCPH1 -D: mean = -2.3, median = -2.3, sd = 0.056, IQR = 0.074, 100.0% < 0; 100.0% significant at  α -level 0.05; one-sample one-sided  t -test vs 0:  t (999) = -1.3e+03,  p  = 0;  
  Africa →  tone1 : mean = 0.12, median = 0.11, sd = 0.21, IQR = 0.3, 69.9% > 0; 0.0% significant at  α -level 0.05; one-sample one-sided  t -test vs 0:  t (999) = 18,  p  = 5.9e-64;  
   ASPM -D →  tone1 : mean = -0.013, median = -0.013, sd = 0.028, IQR = 0.039, 67.2% < 0; 0.0% significant at  α -level 0.05; one-sample one-sided  t -test vs 0:  t (999) = -14,  p  = 3.9e-41;  
   MCPH1 -D →  tone1 : mean = -0.004, median = -0.0039, as = 0.09, IQR = 0.13, 51.1% < 0; 0.0% significant at  α -level 0.05; one-sample one-sided  t -test vs 0:  t (999) = -1.4,  p  = 0.08.  
 
 
 
 
 
 
 Tone  counts  
 The resulting dataset has 110 observations, distributed among 76 unique Glottolg codes in 29 families (ranging from a minimum of 1 language per family to a maximum of 37, with a mean 3.8 and median 2 languages per family) and 4 macroareas. 
 There are 93:78:76 unique samples:(meta)populations:languages retained. 
 
 
 
 
 
 
 
 
 
 
 
   
 Africa 
 Eurasia 
 America 
 Papunesia 
 Sum 
 
 
 
 
  0  
 6 
 52 
 2 
 3 
 63 
 
 
  1  
 6 
 6 
 3 
 0 
 15 
 
 
  2  
 12 
 2 
 0 
 1 
 15 
 
 
  3  
 2 
 3 
 0 
 0 
 5 
 
 
  4  
 0 
 6 
 0 
 0 
 6 
 
 
  5  
 1 
 3 
 0 
 0 
 4 
 
 
  6  
 0 
 2 
 0 
 0 
 2 
 
 
  Sum  
 27 
 74 
 5 
 4 
 110 
 
 
 
 
 
 
  Figure 164.    Distribution of tone  counts . 
 
 
 
 
 
  Figure 165.    Distribution of tone  counts  across the world. 
 
 
 
 
 
  Figure 166.    Relationship between tone  counts  (colors) and the two alleles (frequency) by macroarea. 
 
 
 
 Regressions 
 
  glmer  
 
 All data 
 
  null model : R 2  = 0.0%, ICC = 100.0% 
 the Poisson model is  not  overdispersed:  χ  2 (108) = 68.4,  p  = 1 
  macroarea :  p  macroarea/null  = 0.22 
  ASPM :
 
 by itself: R 2  = 5.7%,  β  = -0.28 ± 0.23,  p  ASPM/null  = 0.22 
 quadratic: R 2  = 9.6%,  β ASPM2   = -0.24 ± 0.23,  p  ASPM2/ASPM  = 0.41 
 with  macroarea : R 2  = 17.6%,  p  macroarea/ASPM  = 0.37,  p  ASPM/macroarea  = 0.62 
  
  MCPH1 :
 
 by itself: R 2  = 10.2%,  β  = -0.40 ± 0.21,  p  MCPH1/null  = 0.064 
 quadratic: R 2  = 12.8%,  β MCPH12   = -0.39 ± 0.21,  p  MCPH12/MCPH1  = 0.31 
 with  macroarea : R 2  = 15.8%,  p  macroarea/MCPH1  = 0.78,  p  MCPH1/macroarea  = 0.76 
  
  both alleles  (no  macroarea ):
 
  ASPM  +  MCPH1 : R 2  = 14.0%,  β  ASPM  = -0.18 ± 0.24,  p  ASPM/MCPH1  = 0.44,  β  MCPH1  = -0.34 ± 0.22,  p  MCPH1/ASPM  = 0.11,  p  ASPM+MCPH1/null  = 0.13, 
 interaction: R 2  = 14.0%,  p  ASPM:MCPH1/ASPM+MCPH1  = 0.9 
  
 
 
 
 Randomization 
 We performed 1000 independent replications: 
 
 Regressions with randomizations for tone  counts . 
 
 
 Permute within 
 Macroarea 
 Permute 
 AIC 
 Signif. 
  p   ASPM -D  
  β   ASPM -D  
  p   MCPH1 -D  
  β   MCPH1 -D  
 
 
 
 
 unrestricted 
 none 
 tone 
 0% 
 18% 
 16% 
 15% 
 13% 
 4% 
 
 
 unrestricted 
 none 
 alleles-together 
 11% 
 4% 
 3% 
 6% 
 4% 
 0% 
 
 
 unrestricted 
 none 
 alleles-independent 
 10% 
 3% 
 4% 
 5% 
 3% 
 0% 
 
 
 unrestricted 
 fixef 
 tone 
 0% 
 24% 
 18% 
 26% 
 18% 
 34% 
 
 
 unrestricted 
 fixef 
 alleles-together 
 86% 
 3% 
 3% 
 15% 
 4% 
 19% 
 
 
 unrestricted 
 fixef 
 alleles-independent 
 84% 
 3% 
 4% 
 12% 
 3% 
 15% 
 
 
 macroareas 
 none 
 tone 
 0% 
 40% 
 20% 
 18% 
 33% 
 22% 
 
 
 macroareas 
 none 
 alleles-together 
 32% 
 15% 
 4% 
 16% 
 17% 
 25% 
 
 
 macroareas 
 none 
 alleles-independent 
 30% 
 12% 
 4% 
 18% 
 16% 
 23% 
 
 
 macroareas 
 fixef 
 tone 
 0% 
 34% 
 24% 
 33% 
 22% 
 38% 
 
 
 macroareas 
 fixef 
 alleles-together 
 85% 
 4% 
 5% 
 21% 
 4% 
 35% 
 
 
 macroareas 
 fixef 
 alleles-independent 
 86% 
 3% 
 4% 
 19% 
 4% 
 33% 
 
 
 families 
 none 
 tone 
 11% 
 6% 
 8% 
 49% 
 2% 
 6% 
 
 
 families 
 none 
 alleles-together 
 24% 
 10% 
 14% 
 66% 
 0% 
 3% 
 
 
 families 
 none 
 alleles-independent 
 26% 
 9% 
 15% 
 65% 
 0% 
 5% 
 
 
 families 
 fixef 
 tone 
 17% 
 7% 
 12% 
 67% 
 1% 
 49% 
 
 
 families 
 fixef 
 alleles-together 
 86% 
 2% 
 6% 
 58% 
 1% 
 3% 
 
 
 families 
 fixef 
 alleles-independent 
 83% 
 2% 
 6% 
 53% 
 1% 
 6% 
 
 
 
 
 
 
Regressions on 1000 permuted data. Each plot shows the original result (vertical dashed black line) and the distribution of the permutations for the three possible things to be permuted (colored curves) for each combination of permutation constraints (horizontal panels) and control for  macroarea  (vertical panels) in terms of the effect size  β ;  ASPM -D is on the left and  MCPH1 -D on the right. The vertical dotted black thin line is at 0.0.
 
 
 
 
 Restricted sampling 
 
 
 
  Figure 167.    Results for 1000 restricted samplings. For  ASPM -D (left): 97.7% of βs are negative when regressing tone on  ASPM  alone (one-sided  t -test < 0:  t (999) = -57.2, mean = -0.35,  p  = 8.7e-318), 88.4%, when controlling for the macroarea ( t (999) = -35.2, mean = -0.31,  p  = 2.6e-177), and 88.5% when controlling for both macroarea and  MCPH1  ( t (999) = -36.1, mean = -0.33,  p  = 2.6e-183). For  MCPH1 -D (right): 99.9% of βs are negative when regressing tone on  MCPH1  alone (one-sided  t -test < 0:  t (999) = -77.4, mean = -0.31,  p  = 0), 50.8% when controlling for the macroarea ( t (999) = 1.5, mean = 0.02,  p  = 0.93), and 44.1% when controlling for both macroarea and  ASPM  ( t (999) = 7.5, mean = 0.10,  p  = 1). 
 
 
 
 
 
  brms  
 
  ASPM  only:
 
  β  = -0.2, 89%HDI = [-0.65, 0.24] 
 posterior probability  p ( β <0) = 0.77 (evidence ratio = 3.3),  p ( β =0) = 0.9 (evidence ratio = 9.1) 
 ROPE = [-0.10, 0.10], % HDI inside ROPE = 24.7%;  p  ROPE  = 0.22 
 comparison ‘null’ vs ‘ASPM’: [B> L> W>(83%:17%) K>]: moderate evidence for null against ASPM (BF=6.54), LOO=1.59 [SE=0.82], WAIC=1.60 [SE=0.85], KFOLD=2.27 [SE=1.55] 
  
  MCPH1  only:
 
  β  = -0.3, 89%HDI = [-0.79, 0.18] 
 posterior probability  p ( β <0) = 0.84 (evidence ratio = 5.5),  p ( β =0) = 0.86 (evidence ratio = 6.2) 
 ROPE = [-0.10, 0.10], % HDI inside ROPE = 15.8%;  p  ROPE  = 0.141 
 comparison ‘null’ vs ‘MCPH1’: [B> L= W=(58%:42%) K>]: moderate evidence for null against MCPH1 (BF=5.03), LOO=0.07 [SE=0.70], WAIC=0.32 [SE=0.59], KFOLD=1.38 [SE=0.83] 
  
  both alleles :
 
 comparison ‘null’ vs ‘both’: [B>> L> W>(81%:19%) K>>]: very strong evidence for null against both (BF=67.8), LOO=1.39 [SE=0.84], WAIC=1.42 [SE=1.02], KFOLD=4.66 [SE=1.61] 
 interaction:
 
 posterior probability  p (=0) = 0.9 (evidence ratio = 8.7) 
 ROPE = [-0.10, 0.10], % HDI inside ROPE = 24%;  p  ROPE  = 0.214 
 comparison ‘no interaction’ vs ‘with interaction’: [B> L= W=(58%:42%) K<]: moderate evidence for no interaction against with interaction (BF=8.92), LOO=0.36 [SE=0.71], WAIC=0.31 [SE=0.37], KFOLD=-6.87 [SE=3.51] 
  
  ASPM  (partial):
 
  β  = -0.17, 89%HDI = [-0.61, 0.24] 
 posterior probability  p ( β <0) = 0.74 (evidence ratio = 2.9),  p ( β =0) = 0.91 (evidence ratio = 9.7) 
 ROPE = [-0.10, 0.10], % HDI inside ROPE = 27%;  p  ROPE  = 0.24 
  
  MCPH1  (partial):
 
  β  = -0.27, 89%HDI = [-0.77, 0.20] 
 posterior probability  p ( β <0) = 0.82 (evidence ratio = 4.7),  p ( β =0) = 0.86 (evidence ratio = 6.3) 
 ROPE = [-0.10, 0.10], % HDI inside ROPE = 17.3%;  p  ROPE  = 0.154 
  
  
 
 
  
 
  Figure 168.    Posterior distributions (with 50% probability mass highlighted) versus 0.0 (the vertical line) for  ASPM -D (left) and  MCPH1 -D (right). 
 
 
 
  
 
  Figure 169.    Conditional effects of  ASPM -D (left) and  MCPH1 -D (right). 
 
 
 
  
 
  Figure 170.    Posterior predictive checks for  ASPM -D (left) and  MCPH1 -D (right). 
 
 
 
 
 Mediation analysis 
 
  (g)lm  
 
 All data 
 For  ASPM -D: 
 
   total effect  (TE) of being in Africa on tone: 0.81 (0.19, 1.64),  p =0.006, decomposed into:  
   average direct effect  (ADE): -0.20 (-0.91, 0.42),  p =0.53, and  
   average indirect effect  (ACME) mediated by  ASPM -D: 1.01 (0.49, 1.89),  p =0, mediating 124.1% (61.9%, 380.1%),  p =0.006 of the effect, resulting from: 
 
 effect of being in Africa on  ASPM -D: -1.34 ±0.18,  p =3.1e-11, and 
 effect of  ASPM -D on tone: -0.58 ±0.14,  p =3.9e-05. 
  
 
 For  MCPH1 -D: 
 
   TE : 0.62 (0.13, 1.17),  p =0.018, decomposed into:  
   ADE : 0.62 (-0.69, 2.13),  p =0.35, and  
   ACME : 0.00 (-1.26, 1.27),  p =1, mediating 0.3% (-277.8%, 294.1%),  p =1 of the effect, resulting from: 
 
 effect of being in Africa on  MCPH1 -D: -2.07 ±0.10,  p =1e-39, and 
 effect of  MCPH1 -D on tone: -0.01 ±0.22,  p =0.96. 
  
 
 
 
 Restricted sampling 
 
 
 
  Figure 171.    Mediation analysis for 1000 restricted samples (i.e., picking one random language per family). The leftmost panels show the distribution of point estimates of the Total Effect (TE), the Direct Effect (ADE) and the Indirect Effect (ACME) for  ASPM  and  MCPH1 ; the middle panels show the distribution of the  p -values for the same effects, while the rightmost panels show the distribution of the regression slopes ( β ) for the two alleles, top: for the regression of the allele frequency on within vs outside Africa, and bottom: for the regression of tone on the allele while controlling for within vs outside Africa. The black vertical lines show: 0.0 (dotted), 0.05 (solid) and 0.10 (dashed). 
 
 
 For  ASPM -D: 
 
   TE : mean = 1.2, median = 1.1; 55.1% significant at  α -level 0.05 and 65.0% significant at  α -level 0.10; 100.0% > 0.0; one-sample one-sided t-test vs 0:  t (999) = 72.9,  p  = 0;  
   ADE : mean = 0.91, median = 0.9; 32.4% significant at  α -level 0.05 and 46.1% significant at  α -level 0.10; 98.3% > 0.0; one-sample one-sided t-test vs 0:  t (999) = 60.8,  p  = 0;  
   ACME : mean = 0.26, median = 0.22; 3.0% significant at  α -level 0.05 and 11.2% significant at  α -level 0.10; 78.0% > 0.0; one-sample one-sided t-test vs 0:  t (999) = 25.7,  p  = 8.1e-113;  
   β(Africa → allele) : mean = -0.89, median = -0.89; 92.1% significant at  α -level 0.05 and 100.0% significant at  α -level 0.10; 100.0% < 0.0; one-sample one-sided t-test vs 0:  t (999) = -447.7,  p  = 0;  
   β(allele → tone | Africa) : mean = -0.18, median = -0.17; 6.5% significant at  α -level 0.05 and 14.2% significant at  α -level 0.10; 78.7% < 0.0; one-sample one-sided t-test vs 0:  t (999) = -27.1,  p  = 3.3e-122.  
 
 For  MCPH1 -D: 
 
   TE : mean = 1.1, median = 1.1; 55.6% significant at  α -level 0.05 and 64.9% significant at  α -level 0.10; 100.0% > 0.0; one-sample one-sided t-test vs 0:  t (999) = 74.9,  p  = 0;  
   ADE : mean = 0.79, median = 0.74; 1.1% significant at  α -level 0.05 and 3.7% significant at  α -level 0.10; 70.7% > 0.0; one-sample one-sided t-test vs 0:  t (999) = 15.0,  p  = 1.6e-46;  
   ACME : mean = 0.31, median = 0.36; 0.2% significant at  α -level 0.05 and 2.9% significant at  α -level 0.10; 60.7% > 0.0; one-sample one-sided t-test vs 0:  t (999) = 5.7,  p  = 8.9e-09;  
   β(Africa → allele) : mean = -2.3, median = -2.3; 100.0% significant at  α -level 0.05 and 100.0% significant at  α -level 0.10; 100.0% < 0.0; one-sample one-sided t-test vs 0:  t (999) = -1254.2,  p  = 0;  
   β(allele → tone | Africa) : mean = -0.12, median = -0.13; 0.4% significant at  α -level 0.05 and 2.7% significant at  α -level 0.10; 67.5% < 0.0; one-sample one-sided t-test vs 0:  t (999) = -13.5,  p  = 1.3e-38.  
 
 Given the low sample size  N  = 35 unique families, relatively few effect sizes are big enough to be significant; however, there are many more significant indirect effects (ACME) for  ASPM -D than for  MCPH1 -D: 6.5% vs 0.4% (16.2 times) for  α -level 0.05, and 14.2% vs 2.7% (5.3 times) for  α -level 0.10. 
 
 
 
  brms  
 
  
 
 
  Figure 172.    Graphical representation of the Bayesian mediation analysis for  ASPM -D showing the means of the effects and the actual partial regression coefficients, with their 89% HDIs and p-ROPEs. The colors reflect the sign of the mean estimate (blue=negative, red=positive, gray=(p-ROPE >= 0.05)); solid=(0 not in the HDI), dashed=(0 is in the HDI). 
 
 
 
  
 
 
  Figure 173.    Graphical representation of the Bayesian mediation analysis for  MCPH1 -D showing the means of the effects and the actual partial regression coefficients, with their 89% HDIs and p-ROPEs. The colors reflect the sign of the mean estimate (blue=negative, red=positive, gray=(p-ROPE >= 0.05)); solid=(0 not in the HDI), dashed=(0 is in the HDI). 
 
 
 
  
 
 
  Figure 174.    Graphical representation of the Bayesian mediation analysis for both  ASPM -D and  MCPH1 -D showing the means of the effects and the actual partial regression coefficients, with their 89% HDIs and p-ROPEs. The colors reflect the sign of the mean estimate (blue=negative, red=positive, gray=(p-ROPE >= 0.05)); solid=(0 not in the HDI), dashed=(0 is in the HDI). 
 
 
 
 
 
 Path analysis 
 Please note that path analysis uses a linear model (so not a Poisson one) for the tone  counts ; also I only use the numeric coding for Africa. 
 
 All data 
 Coding Africa numerically, the model fits the data very well ( χ  2 (1)=0.02,  p =0.88; CFI=1.00, TLI=1.03, NNFI=1.03 and RFI=1.00): 
 
  
 
 
  Figure 175.    Path analysis model with standardised coefficients and significance stars. Here, macroarea (Africa vs non-Africa) is coded as numeric binary ( Africa_num  with in Africa=1);  ASPM_z  is  ASPM -D and  MCPH1_z  is  MCPH1 -D.. 
 
 
 
 
 Restricted sampling 
 
 
 
  Figure 176.    Path analysis for 1000 restricted samples (i.e., picking one random language per family). The leftmost row of two plots shows the coefficient estimates and the  p -values, respectively, for the five paths in the model (see the path plots above). The rightmost plot shows the various fit indices. The black horiontal lines show: 0.0 (solid), 0.05 (dashed) and 1.0 (dotted). 
 
 
 It can be seen that: 
 
  the models fits: 
 
 100% of the  p -values are not significant 
 mean(CFI) = 1, median(CFI) = 1, sd(CFI) = 0, IQR(CFI) = 0 
 mean(TLI) = 1.04, median(TLI) = 1.05, sd(TLI) = 0.06, IQR(TLI) = 0.07 
 mean(NNFI) = 1.04, median(NNFI) = 1.05, sd(NNFI) = 0.06, IQR(NNFI) = 0.07 
 mean(RFI) = 0.94, median(RFI) = 0.95, sd(RFI) = 0.05, IQR(RFI) = 0.07 
  
  Africa →  ASPM -D: mean = -0.89, median = -0.89, sd = 0.064, IQR = 0.084, 100.0% < 0; 100.0% significant at  α -level 0.05; one-sample one-sided  t -test vs 0:  t (999) = -4.4e+02,  p  = 0  
  Africa →  MCPH1 -D: mean = -2.3, median = -2.3, sd = 0.057, IQR = 0.078, 100.0% < 0; 100.0% significant at  α -level 0.05; one-sample one-sided  t -test vs 0:  t (999) = -1.3e+03,  p  = 0  
  Africa → tone  counts : mean = 0.45, median = 0.49, sd = 0.89, IQR = 1.2, 70.2% > 0; 3.0% significant at  α -level 0.05; one-sample one-sided  t -test vs 0:  t (999) = 16,  p  = 1.1e-51  
   ASPM -D → tone  counts : mean = -0.16, median = -0.16, sd = 0.19, IQR = 0.26, 79.9% < 0; 7.3% significant at  α -level 0.05; one-sample one-sided  t -test vs 0:  t (999) = -27,  p  = 2.7e-122  
   MCPH1 -D → tone  counts : mean = -0.18, median = -0.18, as = 0.37, IQR = 0.51, 69.2% < 0; 0.1% significant at  α -level 0.05; one-sample one-sided  t -test vs 0:  t (999) = -16,  p  = 4.7e-49  
 
 
 
 
 
 
 
 Appendix IV: Excluding ambiguous samples 
 The correspondence between genetic samples and languages is given below (excluding the 139 samples that unambiguously correspond to a single language): 
 
 Genetic samples corresponding to more than 1 language. 
 
 
 
 
 
 
 
 
 
 pop_ID 
 n_languages 
 n_families 
 languages 
 families 
 
 
 
 
 SA004382R 
 144 
 1 
 ’Are’are, Adzera, Äiwoo, Ajië, Amara, Aneityum, Araki, Aribwatsa, Arop-Lokep, Arosi, Aulua, Babatana, Bannoni, Big Nambas, Carolinian, Cemuhî, Cheke Holo, Chuukese, Dehu, Dumbea, East Ambae, East Futuna, East Uvean, Fijian, Futuna-Aniwa, Fwâi, Gapapaiwa, Gela, Gilbertese, Gumawana, Halia, Hano, Hawaiian, Hoava, Iaai, Iduna, Kairiru, Kapingamarangi, Kara (Papua New Guinea), Kaulong, Kela (Papua New Guinea), Kele (Papua New Guinea), Kilivila, Kokota, Kosraean, Kuanua, Kwaio, Kwamera, Labu, Lala, Lamenu, Lau, Lenakel, Lewo, Longgu, Loniu, Lonwolwol, Lou, Luangiua, Lusi, Maisin, Maleu-Kilenge, Manam, Maori, Marshallese, Matukar, Mbula, Mekeo, Mele-Fila, Minaveha, Mokilese, Mono-Alu, Motu, Muduapa, Musom, Mussau-Emira, Muyuw, Mwotlap, Nakanai, Nalik, Natügu, Nauru, Nehan, Nêlêmwa-Nixumwak, Nengone, Neve’ei, Niuafo’ou, Niuean, North Efate, North Marquesan, Nukuoro, Paama, Patep, Patpatar, Pingelapese, Pohnpeian, Port Sandwich, Puluwatese, Rapanui, Rennell-Bellona, Rotuman, Roviana, Sa’a, Saliba, Samoan, Saposa, Siar-Lak, Sie, Sinaugoro, Sio, Sobei, Sonsorol, South Efate, South Marquesan, Southwest Tanna, Sudest, Sursurunga, Tahitian, Takia, Tamambo, Tawala, Teanu, Teop, Tigak, Tirax, Tiri-Mea, To’abaita, Tobati, Tokelau, Tonga (Tonga Islands), Tuamotuan, Tumleo, Tungag, Tuvalu, Ulithian, Ura (Vanuatu), Uripiv-Wala-Rano-Atchin, Vaeakau-Taumako, Waima, Wanohe, Western Fijian, Woleaian, Xârâcùù, Yabem 
 Austronesian 
 
 
 SA001501H 
 23 
 4 
 Abau, Alamblak, Ambulas, Ap Ma, Awtuw, Bahinemo, Boikin, Chambri, Hanga Hundi, Iatmul, Iwam, Kaian, Kire, Kwoma, Manambu, Mehek, Murik (Papua New Guinea), Namia, Rao, Watam, Wogamusin, Yessan-Mayo, Yimas 
 Ap Ma, Lower Sepik-Ramu, Ndu, Sepik 
 
 
 SA001818S 
 9 
 1 
 Herero, Kuanyama, Kwambi, Mbalanhu, Ndonga, Ngandyera, Southern Sotho, Tswana, Zulu 
 Atlantic-Congo 
 
 
 SA004368V 
 7 
 1 
 Amharic, Awngi, Bilin, Geez, Qimant, Tigrinya, Xamtanga 
 Afro-Asiatic 
 
 
 SA004046O 
 6 
 1 
 Bukusu, Idakho-Isukha-Tiriki, Kisa, Masaaba, Saamia, Tsotso 
 Atlantic-Congo 
 
 
 SA001469U 
 5 
 3 
 East Taa, Hai//om-Akhoe, Nama (Namibia), North-Central Ju, South-Eastern Ju 
 Khoe-Kwadi, Kxa, Tuu 
 
 
 SA001467S 
 4 
 1 
 Eastern Maninkakan, Kita Maninkakan, Mandinka, Western Maninkakan 
 Mande 
 
 
 SA001819T 
 4 
 1 
 Gusii, Kamba (Kenya), Kikuyu, Meru 
 Atlantic-Congo 
 
 
 SA003646T 
 4 
 1 
 Eastern Maninkakan, Kita Maninkakan, Mandinka, Western Maninkakan 
 Mande 
 
 
 SA001476S 
 3 
 1 
 Eastern Balochi, Southern Balochi, Western Balochi 
 Indo-European 
 
 
 SA001478U 
 3 
 1 
 Eastern Balochi, Southern Balochi, Western Balochi 
 Indo-European 
 
 
 SA004365S 
 3 
 1 
 Kahe, Machame, Mochi 
 Atlantic-Congo 
 
 
 SA004371P 
 3 
 1 
 Modern Hebrew, South Levantine Arabic, Standard Arabic 
 Afro-Asiatic 
 
 
 ESTONIAN_VAR 
 2 
 1 
 Estonian, South Estonian 
 Uralic 
 
 
 MB2005_BakolaPygmy 
 2 
 1 
 Gyele, Kwasio 
 Atlantic-Congo 
 
 
 Qatari 
 2 
 1 
 Gulf Arabic, Standard Arabic 
 Afro-Asiatic 
 
 
 SA001466R 
 2 
 1 
 Efe, Lese 
 Central Sudanic 
 
 
 SA001474Q 
 2 
 1 
 South Levantine Arabic, Standard Arabic 
 Afro-Asiatic 
 
 
 SA001483Q 
 2 
 1 
 Mandarin Chinese, Yue Chinese 
 Sino-Tibetan 
 
 
 SA001486T 
 2 
 1 
 Central Mashan Hmong, Hmong Njua 
 Hmong-Mien 
 
 
 SA001493R 
 2 
 1 
 Lü, Tai Nüa 
 Tai-Kadai 
 
 
 SA001508O 
 2 
 1 
 English, Scots 
 Indo-European 
 
 
 SA002254N 
 2 
 1 
 South Levantine Arabic, Standard Arabic 
 Afro-Asiatic 
 
 
 SA002257Q 
 2 
 1 
 South Levantine Arabic, Standard Arabic 
 Afro-Asiatic 
 
 
 SA002262M 
 2 
 1 
 Central Pashto, Northern Pashto 
 Indo-European 
 
 
 SA003028N 
 2 
 1 
 Estonian, South Estonian 
 Uralic 
 
 
 SA004111H 
 2 
 1 
 English, Spanish 
 Indo-European 
 
 
 SA004238R 
 2 
 1 
 Lü, Tai Nüa 
 Tai-Kadai 
 
 
 SA004361O 
 2 
 1 
 Efe, Lese 
 Central Sudanic 
 
 
 SA004370O 
 2 
 1 
 Judeo-Yemeni Arabic, Modern Hebrew 
 Afro-Asiatic 
 
 
 SA004378W 
 2 
 1 
 English, Irish 
 Indo-European 
 
 
 SA004587Y 
 2 
 1 
 Mongolia Buriat, Russia Buriat 
 Mongolic-Khitan 
 
 
 SA004592U 
 2 
 1 
 Mandarin Chinese, Yue Chinese 
 Sino-Tibetan 
 
 
 SA004599B 
 2 
 1 
 Erzya, Moksha 
 Uralic 
 
 
 SA004603N 
 2 
 1 
 Church Slavic, Russian 
 Indo-European 
 
 
 SA004623P 
 2 
 1 
 Georgian, Mingrelian 
 Kartvelian 
 
 
 
 Even if it is very conservative (e.g.,  Church Slavic  and  Russian  corresponding to  SA004603N  are very similar for tone, as are  Modern Hebrew ,  South Levantine Arabic  and  Standard Arabic  corresponding to  SA004371P ), I removed from this analysis all genetic samples that map to more than 1 language. 
 This results in 139 unique genetic samples corresponding to 103 (meta)populations, each mapping to a single language, distributed across 91 unique languages (i.e., it is still the case that more than one sample maps to the same language) in 4 macroareas: 
 
 
 
 
 
 
 
 
 
 Africa 
 Eurasia 
 America 
 Papunesia 
 
 
 
 
 16 
 109 
 10 
 4 
 
 
 
 
  tone1  
 There are 126 observations, distributed among 88 unique Glottolg codes in 30 families (ranging from a minimum of 1 language per family to a maximum of 37, with a mean 4.2 and median 2 languages per family) and 4 macroareas. 
 There are 126:100:88 unique samples:(meta)populations:languages retained. 
 
 
 
 
 
 
 
 
 
 
 
   
 Africa 
 Eurasia 
 America 
 Papunesia 
 Sum 
 
 
 
 
  No  
 2 
 79 
 4 
 4 
 89 
 
 
  Yes  
 14 
 17 
 6 
 0 
 37 
 
 
  Sum  
 16 
 96 
 10 
 4 
 126 
 
 
 
 
 
 
  Figure 177.    Distribution of  tone1 . 
 
 
 
 
 
  Figure 178.    Map of  tone1 . 
 
 
 
 
 
  Figure 179.    Relationship between  tone1 ,  ASPM -D and  MCPH1 -D. 
 
 
 
 Regressions 
 
  glmer  
 
 All data 
 
  null model : R 2  = 0.0%, ICC = 79.9% 
  macroarea :  p  macroarea/null  = 0.0027 
  ASPM :
 
 by itself: R 2  = 6.9%,  β  = -0.92 ± 0.54,  p  ASPM/null  = 0.094 
 quadratic: R 2  = 6.3%,  β ASPM2   = -0.91 ± 0.52,  p  ASPM2/ASPM  = 0.77 
 with  macroarea : R 2  = 54.9%,  p  macroarea/ASPM  = 0.008,  p  ASPM/macroarea  = 0.48 
  
  MCPH1 :
 
 by itself: R 2  = 7.1%,  β  = -1.01 ± 0.47,  p  MCPH1/null  = 0.032 
 quadratic: R 2  = 7.2%,  β MCPH12   = -1.02 ± 0.47,  p  MCPH12/MCPH1  = 0.9 
 with  macroarea : R 2  = 53.3%,  p  macroarea/MCPH1  = 0.018,  p  MCPH1/macroarea  = 0.45 
  
  both alleles  (no  macroarea ):
 
  ASPM  +  MCPH1 : R 2  = 10.6%,  β  ASPM  = -0.58 ± 0.60,  p  ASPM/MCPH1  = 0.36,  β  MCPH1  = -0.77 ± 0.48,  p  MCPH1/ASPM  = 0.1,  p  ASPM+MCPH1/null  = 0.065, 
 interaction: R 2  = 10.0%,  p  ASPM:MCPH1/ASPM+MCPH1  = 0.56 
  
 
 
 Alleles on macroarea 
 To better understand this overlap between family, macroarea and the two “derived” alleles, I regressed (separately) the  ASPM -D and  MCPH1 -D on the  macroarea , using mixed-effects  beta regression  (after replacing all  \(0.0\)  values by  \(10^{-7}\)  and all  \(1.0\)  by  \(1.0-10^{-7}\) , respectively) with language family as random effect: 
 
 the alleles are very strongly clustered within  families :
 
  ASPM : ICC = 100.0% 
  MCPH1 : ICC = 100.0% 
  
  macroarea  predicts their distribution very strongly:
 
  ASPM :  p  = 8.6e-05, R 2  = 48.8% 
  MCPH1 :  p  = 6.2e-10, R 2  = 77.6% 
  
 separating Africa vs the rest of the world seems to drive most of this effect (both alleles have lower frequencies in Africa):
 
  ASPM :  p  = 0.023, R 2  = 15.8% 
  MCPH1 :  p  = 3.1e-07, R 2  = 47.5% 
  
 
 
 
 
 Randomization 
 I performed 1000 independent replications of each of these parameter combinations, and below are the distributions of the permuted values versus the original ones (i.e., those obtained on the original, non-permuted data). 
 
 Regressions on 1000 permuted data. The first 3 columns show the permutation constraints (if any), how the  macroarea  is considered (if at all), and what is permuted. The next columns show the percent of the permutations that, in order, have a better AIC compared to the original fit, are significantly better than the null model (thus testing the effect of both alleles simultaneously), have a significant effect of  ASPM -D, have a smaller effect ( β ) of  ASPM -D than the original fit, and the same for  MCPH1 -D. 
 
 
 Permute within 
 Macroarea 
 Permute 
 AIC 
 Signif. 
  p   ASPM -D  
  β   ASPM -D  
  p   MCPH1 -D  
  β   MCPH1 -D  
 
 
 
 
 unrestricted 
 none 
 tone 
 0% 
 4% 
 4% 
 0% 
 5% 
 0% 
 
 
 unrestricted 
 none 
 alleles-together 
 6% 
 5% 
 5% 
 7% 
 6% 
 4% 
 
 
 unrestricted 
 none 
 alleles-independent 
 7% 
 6% 
 5% 
 7% 
 5% 
 2% 
 
 
 unrestricted 
 fixef 
 tone 
 0% 
 6% 
 5% 
 13% 
 7% 
 15% 
 
 
 unrestricted 
 fixef 
 alleles-together 
 70% 
 6% 
 5% 
 25% 
 6% 
 19% 
 
 
 unrestricted 
 fixef 
 alleles-independent 
 67% 
 6% 
 6% 
 24% 
 5% 
 16% 
 
 
 macroareas 
 none 
 tone 
 0% 
 72% 
 28% 
 24% 
 67% 
 7% 
 
 
 macroareas 
 none 
 alleles-together 
 30% 
 24% 
 8% 
 28% 
 20% 
 40% 
 
 
 macroareas 
 none 
 alleles-independent 
 35% 
 29% 
 14% 
 37% 
 25% 
 46% 
 
 
 macroareas 
 fixef 
 tone 
 0% 
 5% 
 5% 
 14% 
 6% 
 21% 
 
 
 macroareas 
 fixef 
 alleles-together 
 64% 
 4% 
 4% 
 24% 
 5% 
 28% 
 
 
 macroareas 
 fixef 
 alleles-independent 
 64% 
 5% 
 4% 
 27% 
 4% 
 26% 
 
 
 families 
 none 
 tone 
 44% 
 21% 
 5% 
 24% 
 16% 
 38% 
 
 
 families 
 none 
 alleles-together 
 18% 
 15% 
 6% 
 32% 
 6% 
 17% 
 
 
 families 
 none 
 alleles-independent 
 30% 
 27% 
 20% 
 49% 
 19% 
 27% 
 
 
 families 
 fixef 
 tone 
 27% 
 6% 
 4% 
 37% 
 4% 
 38% 
 
 
 families 
 fixef 
 alleles-together 
 70% 
 8% 
 6% 
 50% 
 3% 
 24% 
 
 
 families 
 fixef 
 alleles-independent 
 74% 
 8% 
 10% 
 52% 
 5% 
 35% 
 
 
 
 
 
 
Regressions on 1000 permuted data. Each plot shows the original result (vertical dashed black line) and the distribution of the permutations for the three possible things to be permuted (colored curves) for each combination of permutation constraints (horizontal panels) and control for  macroarea  (vertical panels) in terms of the effect size  β ;  ASPM -D is on the left and  MCPH1 -D on the right. The vertical dotted black thin line is at 0.0.
 
 
 
 
 Restricted sampling 
 
 
 
  Figure 180.    Results for 1000 restricted samplings. For  ASPM -D (left): 100% of βs are negative when regressing tone on  ASPM  alone (one-sided  t -test < 0:  t (999) = -137.3, mean = -1.04,  p  = 0), 99.5%, when controlling for the macroarea ( t (999) = -76.0, mean = -0.95,  p  = 0), and 99.5% when controlling for both macroarea and  MCPH1  ( t (999) = -67.1, mean = -1.13,  p  = 0). For  MCPH1 -D (right): 100% of βs are negative when regressing tone on  MCPH1  alone (one-sided  t -test < 0:  t (999) = -100.4, mean = -0.78,  p  = 0), 92.4% when controlling for the macroarea ( t (999) = -45.7, mean = -1.42,  p  = 1.4e-247), and 92.5% when controlling for both macroarea and  ASPM  ( t (999) = -47.9, mean = -1.75,  p  = 3.3e-261). 
 
 
 
 
 
  brms  
 
  ASPM  only:
 
  β  = -1.17, 89%HDI = [-3.06, 0.82] 
 posterior probability  p ( β <0) = 0.84 (evidence ratio = 5.2),  p ( β =0) = 0.66 (evidence ratio = 1.9) 
 ROPE = [-0.18, 0.18], % HDI inside ROPE = 9%;  p  ROPE  = 0.08 
 comparison ‘null’ vs ‘ASPM’: [B= L< W<(15%:85%) K=]: anecdotal evidence for null against ASPM (BF=1.85), LOO=-2.20 [SE=1.49], WAIC=-1.73 [SE=0.89], KFOLD=0.55 [SE=2.12] 
 comparison ‘null’ vs ‘ASPM’: [B= L< W<(15%:85%) K=]: anecdotal evidence for null against ASPM (BF=1.85), LOO=-2.20 [SE=1.49], WAIC=-1.73 [SE=0.89], KFOLD=0.55 [SE=2.12] 
  
  MCPH1  only:
 
  β  = -1.5, 89%HDI = [-3.58, 0.81] 
 posterior probability  p ( β <0) = 0.87 (evidence ratio = 7),  p ( β =0) = 0.59 (evidence ratio = 1.4) 
 ROPE = [-0.18, 0.18], % HDI inside ROPE = 6.7%;  p  ROPE  = 0.06 
 comparison ‘null’ vs ‘MCPH1’: [B= L<< W<<(7%:93%) K<]: anecdotal evidence for null against MCPH1 (BF=1.44), LOO=-3.54 [SE=1.49], WAIC=-2.57 [SE=0.77], KFOLD=-2.53 [SE=1.67] 
 comparison ‘null’ vs ‘MCPH1’: [B= L<< W<<(7%:93%) K<]: anecdotal evidence for null against MCPH1 (BF=1.44), LOO=-3.54 [SE=1.49], WAIC=-2.57 [SE=0.77], KFOLD=-2.53 [SE=1.67] 
  
  both alleles :
 
 comparison ‘null’ vs ‘both’: [B> L<< W<<(1%:99%) K=]: moderate evidence for null against both (BF=3.18), LOO=-4.04 [SE=1.54], WAIC=-4.41 [SE=1.11], KFOLD=-1.87 [SE=2.04] 
 interaction:
 
 posterior probability  p (=0) = 0.64 (evidence ratio = 1.7) 
 ROPE = [-0.18, 0.18], % HDI inside ROPE = 8.6%;  p  ROPE  = 0.076 
 comparison ‘no interaction’ vs ‘with interaction’: [B= L< W<<(21%:79%) K>]: anecdotal evidence for no interaction against with interaction (BF=1.25), LOO=-1.44 [SE=0.86], WAIC=-1.30 [SE=0.47], KFOLD=1.08 [SE=1.05] 
  
  ASPM  (partial):
 
  β  = -1.02, 89%HDI = [-3.30, 1.32] 
 posterior probability  p ( β <0) = 0.77 (evidence ratio = 3.3),  p ( β =0) = 0.7 (evidence ratio = 2.3) 
 ROPE = [-0.18, 0.18], % HDI inside ROPE = 11.1%;  p  ROPE  = 0.098 
  
  MCPH1  (partial):
 
  β  = -1.53, 89%HDI = [-3.91, 0.86] 
 posterior probability  p ( β <0) = 0.86 (evidence ratio = 6.4),  p ( β =0) = 0.6 (evidence ratio = 1.5) 
 ROPE = [-0.18, 0.18], % HDI inside ROPE = 7.2%;  p  ROPE  = 0.064 
  
  
 
 
  
 
  Figure 181.    Posterior distributions (with 50% probability mass highlighted) versus 0.0 (the vertical line) for  ASPM -D (left) and  MCPH1 -D (right). 
 
 
 
  
 
  Figure 182.    Conditional effects of  ASPM -D (left) and  MCPH1 -D (right). 
 
 
 
  
 
  Figure 183.    Posterior predictive checks for  ASPM -D (left) and  MCPH1 -D (right). 
 
 
 
  
 
  Figure 184.    Confusion matrices for  ASPM -D (left) and  MCPH1 -D (right). 
 
 
 
 
 
 Mediation and path analysis 
 
 Mediation analysis 
 
  (g)lm  
 
 All data 
 For  ASPM -D: 
 
   total effect  (TE) of being in Africa on tone: 0.63 (0.37, 0.78),  p =0, decomposed into:  
   average direct effect  (ADE): 0.44 (0.15, 0.67),  p =0, and  
   average indirect effect  (ACME) mediated by  ASPM -D: 0.19 (0.07, 0.34),  p =0, mediating 30.0% (9.7%, 62.3%),  p =0 of the effect, resulting from: 
 
 effect of being in Africa on  ASPM -D: -1.17 ±0.25,  p =6.3e-06, and 
 effect of  ASPM -D on tone: -1.02 ±0.30,  p =0.00057. 
  
 
 For  MCPH1 -D: 
 
   TE : 0.64 (0.40, 0.79),  p =0, decomposed into:  
   ADE : 0.71 (0.38, 0.86),  p =0, and  
   ACME : -0.07 (-0.22, 0.22),  p =0.37, mediating -12.3% (-51.6%, 35.7%),  p =0.37 of the effect, resulting from: 
 
 effect of being in Africa on  MCPH1 -D: -2.45 ±0.15,  p =8.9e-32, and 
 effect of  MCPH1 -D on tone: 0.40 ±0.44,  p =0.37. 
  
 
 
 
 Restricted sampling 
 
 
 
  Figure 185.    Mediation analysis for 1000 restricted samples (i.e., picking one random language per family). The leftmost panels show the distribution of point estimates of the Total Effect (TE), the Direct Effect (ADE) and the Indirect Effect (ACME) for  ASPM -D and  MCPH1 -D; the middle panels show the distribution of the  p -values for the same effects, while the rightmost panels show the distribution of the regression slopes ( β ) for the two alleles, top: for the regression of the allele frequency on within vs outside Africa, and bottom: for the regression of tone on the allele while controlling for within vs outside Africa. The black vertical lines show: 0.0 (solid), 0.05 (dashed) and 0.10 (dotted). 
 
 
 For  ASPM -D: 
 
   TE : mean = 0.2, median = 0.15; 0.0% significant at  α -level 0.05 and 10.4% significant at  α -level 0.10; 100.0% > 0.0; one-sample one-sided t-test vs 0:  t (999) = 59.0,  p  = 0;  
   ADE : mean = 0.15, median = 0.11; 0.0% significant at  α -level 0.05 and 2.6% significant at  α -level 0.10; 98.8% > 0.0; one-sample one-sided t-test vs 0:  t (999) = 42.9,  p  = 5.1e-229;  
   ACME : mean = 0.057, median = 0.056; 0.0% significant at  α -level 0.05 and 2.4% significant at  α -level 0.10; 99.9% > 0.0; one-sample one-sided t-test vs 0:  t (999) = 86.9,  p  = 0;  
   β(Africa → allele) : mean = -0.63, median = -0.67; 5.0% significant at  α -level 0.05 and 38.4% significant at  α -level 0.10; 100.0% < 0.0; one-sample one-sided t-test vs 0:  t (999) = -123.7,  p  = 0;  
   β(allele → tone | Africa) : mean = -0.81, median = -0.8; 8.1% significant at  α -level 0.05 and 31.0% significant at  α -level 0.10; 99.9% < 0.0; one-sample one-sided t-test vs 0:  t (999) = -100.0,  p  = 0.  
 
 For  MCPH1 -D: 
 
   TE : mean = 0.2, median = 0.15; 0.0% significant at  α -level 0.05 and 10.4% significant at  α -level 0.10; 100.0% > 0.0; one-sample one-sided t-test vs 0:  t (999) = 57.6,  p  = 3.9e-320;  
   ADE : mean = 0.22, median = 0.2; 0.1% significant at  α -level 0.05 and 2.1% significant at  α -level 0.10; 98.9% > 0.0; one-sample one-sided t-test vs 0:  t (999) = 58.3,  p  = 0;  
   ACME : mean = -0.017, median = -0.04; 0.0% significant at  α -level 0.05 and 0.5% significant at  α -level 0.10; 34.7% > 0.0; one-sample one-sided t-test vs 0:  t (999) = -4.9,  p  = 1;  
   β(Africa → allele) : mean = -2.5, median = -2.5; 100.0% significant at  α -level 0.05 and 100.0% significant at  α -level 0.10; 100.0% < 0.0; one-sample one-sided t-test vs 0:  t (999) = -487.0,  p  = 0;  
   β(allele → tone | Africa) : mean = 0.37, median = 0.39; 0.0% significant at  α -level 0.05 and 0.4% significant at  α -level 0.10; 26.3% < 0.0; one-sample one-sided t-test vs 0:  t (999) = 21.3,  p  = 1.  
 
 Given the low sample size  N  = 30 unique families, relatively few effect sizes are big enough to be significant for each individual analysis; however, there are many more significant ACMEs for  ASPM -D than for  MCPH1 -D: 8.1% vs 0.0% (Inf times) for  α -level 0.05, and 31.0% vs 0.4% (77.5 times) for  α -level 0.10. 
 
 
 
  brms  
 
  
 
 
  Figure 186.    Graphical representation of the Bayesian mediation analysis for  ASPM -D showing the means of the effects and the actual partial regression coefficients, with their 89% HDIs and p-ROPEs. The colors reflect the sign of the mean estimate (blue=negative, red=positive, gray=(p-ROPE >= 0.05)); solid=(0 not in the HDI), dashed=(0 is in the HDI). 
 
 
 
  
 
 
  Figure 187.    Graphical representation of the Bayesian mediation analysis for  MCPH1 -D showing the means of the effects and the actual partial regression coefficients, with their 89% HDIs and p-ROPEs. The colors reflect the sign of the mean estimate (blue=negative, red=positive, gray=(p-ROPE >= 0.05)); solid=(0 not in the HDI), dashed=(0 is in the HDI). 
 
 
 
  
 
 
  Figure 188.    Graphical representation of the Bayesian mediation analysis for both  ASPM -D and  MCPH1 -D showing the means of the effects and the actual partial regression coefficients, with their 89% HDIs and p-ROPEs. The colors reflect the sign of the mean estimate (blue=negative, red=positive, gray=(p-ROPE >= 0.05)); solid=(0 not in the HDI), dashed=(0 is in the HDI). 
 
 
 
 
 
 Path analysis 
 
 All data 
 With Africa and  tone1  coded numerically, the model fit is:  χ  2 (1)=0.03,  p =0.86; CFI=1.00, TLI=1.03, NNFI=1.03 and RFI=1.00: 
 
  
 
 
  Figure 189.    Path analysis model with standardised coefficients and significance stars.  tone1  and  macroarea  (Africa vs non-Africa) are coded as numeric binary ( tone_bin_num  with Yes=1 and  Africa_num  with in Africa=1);  ASPM_z  is  ASPM -D and  MCPH1_z  is  MCPH1 -D. 
 
 
 Likewise, with Africa and  tone1  coded as ordered binary factors, the model fit is:  χ  2 (1)=0.08,  p =0.78; CFI=1.00, TLI=1.21, NNFI=1.21 and RFI=0.99: 
 
  
 
 
  Figure 190.    Path analysis model with standardised coefficients and significance stars.  tone1  and  macroarea  (Africa vs non-Africa) are coded as ordered binary factors ( tone_bin_ord  with No < Yes, and  Africa_ord  with outside Africa < in Africa);  ASPM_z  is  ASPM -D and  MCPH1_z  is  MCPH1 -D. 
 
 
 
 
 Restricted sampling 
 
 
 
  Figure 191.    Path analysis for 1000 restricted samples (i.e., picking one random language per family). The leftmost row of two plots shows the coefficient estimates and the  p -values, respectively, for the five paths in the model (see the path plots above). The rightmost plot shows the various fit indices. The black horiontal lines show: 0.0 (solid), 0.05 (dashed) and 1.0 (dotted). 
 
 
 
  models fits: 
 
 80.7% of the  p -values are not significant 
 mean(CFI) = 0.98, median(CFI) = 0.99, sd(CFI) = 0.03, IQR(CFI) = 0.04 
 mean(TLI) = 0.9, median(TLI) = 0.96, sd(TLI) = 0.2, IQR(TLI) = 0.27 
 mean(NNFI) = 0.9, median(NNFI) = 0.96, sd(NNFI) = 0.2, IQR(NNFI) = 0.27 
 mean(RFI) = 0.81, median(RFI) = 0.86, sd(RFI) = 0.17, IQR(RFI) = 0.24 
  
  Africa →  ASPM -D: mean = -0.62, median = -0.66, sd = 0.16, IQR = 0.21, 100.0% < 0; 79.4% significant at  α -level 0.05; one-sample one-sided  t -test vs 0:  t (999) = -1.2e+02,  p  = 0;  
  Africa →  MCPH1 -D: mean = -2.5, median = -2.5, sd = 0.16, IQR = 0.23, 100.0% < 0; 100.0% significant at  α -level 0.05; one-sample one-sided  t -test vs 0:  t (999) = -5.1e+02,  p  = 0;  
  Africa →  tone1 : mean = 0.49, median = 0.53, sd = 0.36, IQR = 0.52, 88.1% > 0; 20.3% significant at  α -level 0.05; one-sample one-sided  t -test vs 0:  t (999) = 43,  p  = 4.3e-232;  
   ASPM -D →  tone1 : mean = -0.16, median = -0.16, sd = 0.051, IQR = 0.069, 99.9% < 0; 52.2% significant at  α -level 0.05; one-sample one-sided  t -test vs 0:  t (999) = -1e+02,  p  = 0;  
   MCPH1 -D →  tone1 : mean = 0.0056, median = 0.02, as = 0.12, IQR = 0.17, 43.1% < 0; 1.0% significant at  α -level 0.05; one-sample one-sided  t -test vs 0:  t (999) = 1.4,  p  = 0.92.  
 
 
 
 
 
 
  tone2  
 The resulting dataset has 121 observations, distributed among 83 unique Glottolg codes in 30 families (ranging from a minimum of 1 language per family to a maximum of 36, with a mean 4 and median 1.5 languages per family) and 4 macroareas. 
 There are 121:95:83 unique samples:(meta)populations:languages retained. 
 
 
 
 
 
 
 
 
 
 
 
   
 Africa 
 Eurasia 
 America 
 Papunesia 
 Sum 
 
 
 
 
  No  
 8 
 82 
 9 
 4 
 103 
 
 
  Yes  
 6 
 11 
 1 
 0 
 18 
 
 
  Sum  
 14 
 93 
 10 
 4 
 121 
 
 
 
 
 
 
  Figure 192.    Distribution of  tone2 . 
 
 
 
 
 
  Figure 193.    Map of  tone2 . 
 
 
 
 
 
  Figure 194.    Relationship between  tone2 ,  ASPM -D and  MCPH1 -D. 
 
 
 
 Regressions 
 
  glmer  
 
 All data 
 
  null model : R 2  = 0.0%, ICC = 97.3% 
  macroarea :  p  macroarea/null  = 0.58 
  ASPM :
 
 by itself: R 2  = 0.7%,  β  = -0.89 ± 1.03,  p  ASPM/null  = 0.37 
 quadratic: R 2  = 51.9%,  β ASPM2   = -10.31 ± 6.43,  p  ASPM2/ASPM  = 0.0089 
 with  macroarea : R 2  = 11.3%,  p  macroarea/ASPM  = 0.74,  p  ASPM/macroarea  = 0.78 
  
  MCPH1 :
 
 by itself: R 2  = 1.2%,  β  = -1.18 ± 0.90,  p  MCPH1/null  = 0.15 
 quadratic: R 2  = 2.2%,  β MCPH12   = -1.38 ± 0.90,  p  MCPH12/MCPH1  = 0.31 
 with  macroarea : R 2  = 8.7%,  p  macroarea/MCPH1  = 0.94,  p  MCPH1/macroarea  = 0.47 
  
  both alleles  (no  macroarea ):
 
  ASPM  +  MCPH1 : R 2  = 1.5%,  β  ASPM  = -0.42 ± 1.20,  p  ASPM/MCPH1  = 0.73,  β  MCPH1  = -1.05 ± 0.96,  p  MCPH1/ASPM  = 0.24,  p  ASPM+MCPH1/null  = 0.33, 
 interaction: R 2  = 2.2%,  p  ASPM:MCPH1/ASPM+MCPH1  = 0.68 
  
 
 
 
 Randomization 
 I performed 1000 independent replications of each of these parameter combinations, and below are the distributions of the permuted values versus the original ones (i.e., those obtained on the original, non-permuted data). 
 
 Regressions on 1000 permuted data. The first 3 columns show the permutation constraints (if any), how the  macroarea  is considered (if at all), and what is permuted. The next columns show the percent of the permutations that, in order, have a better AIC compared to the original fit, are significantly better than the null model (thus testing the effect of both alleles simultaneously), have a significant effect of  ASPM -D, have a smaller effect ( β ) of  ASPM -D than the original fit, and the same for  MCPH1 -D. 
 
 
 Permute within 
 Macroarea 
 Permute 
 AIC 
 Signif. 
  p   ASPM -D  
  β   ASPM -D  
  p   MCPH1 -D  
  β   MCPH1 -D  
 
 
 
 
 unrestricted 
 none 
 tone 
 0% 
 6% 
 5% 
 7% 
 6% 
 0% 
 
 
 unrestricted 
 none 
 alleles-together 
 42% 
 11% 
 10% 
 25% 
 10% 
 6% 
 
 
 unrestricted 
 none 
 alleles-independent 
 42% 
 9% 
 9% 
 28% 
 7% 
 4% 
 
 
 unrestricted 
 fixef 
 tone 
 0% 
 6% 
 5% 
 13% 
 6% 
 1% 
 
 
 unrestricted 
 fixef 
 alleles-together 
 81% 
 10% 
 10% 
 30% 
 10% 
 8% 
 
 
 unrestricted 
 fixef 
 alleles-independent 
 80% 
 11% 
 8% 
 27% 
 11% 
 9% 
 
 
 macroareas 
 none 
 tone 
 0% 
 43% 
 2% 
 7% 
 52% 
 0% 
 
 
 macroareas 
 none 
 alleles-together 
 46% 
 8% 
 7% 
 41% 
 8% 
 28% 
 
 
 macroareas 
 none 
 alleles-independent 
 46% 
 6% 
 7% 
 41% 
 7% 
 28% 
 
 
 macroareas 
 fixef 
 tone 
 0% 
 6% 
 5% 
 15% 
 5% 
 1% 
 
 
 macroareas 
 fixef 
 alleles-together 
 79% 
 9% 
 7% 
 37% 
 9% 
 21% 
 
 
 macroareas 
 fixef 
 alleles-independent 
 79% 
 10% 
 10% 
 35% 
 9% 
 21% 
 
 
 families 
 none 
 tone 
 82% 
 9% 
 5% 
 44% 
 10% 
 24% 
 
 
 families 
 none 
 alleles-together 
 49% 
 6% 
 6% 
 42% 
 6% 
 16% 
 
 
 families 
 none 
 alleles-independent 
 52% 
 7% 
 7% 
 48% 
 6% 
 20% 
 
 
 families 
 fixef 
 tone 
 87% 
 6% 
 7% 
 53% 
 5% 
 25% 
 
 
 families 
 fixef 
 alleles-together 
 87% 
 12% 
 7% 
 38% 
 13% 
 18% 
 
 
 families 
 fixef 
 alleles-independent 
 88% 
 13% 
 9% 
 42% 
 12% 
 21% 
 
 
 
 
 
 
Regressions on 1000 permuted data. Each plot shows the original result (vertical dashed black line) and the distribution of the permutations for the three possible things to be permuted (colored curves) for each combination of permutation constraints (horizontal panels) and control for  macroarea  (vertical panels) in terms of the effect size  β ;  ASPM -D is on the left and  MCPH1 -D on the right. The vertical dotted black thin line is at 0.0.
 
 
 
 
 Restricted sampling 
 
 
 
  Figure 195.    Results for 1000 restricted samplings. For  ASPM -D (left): 99.7% of βs are negative when regressing tone on  ASPM  alone (one-sided  t -test < 0:  t (999) = -76.7, mean = -0.49,  p  = 0), 99.8%, when controlling for the macroarea ( t (999) = -76.3, mean = -0.77,  p  = 0), and 99.7% when controlling for both macroarea and  MCPH1  ( t (999) = -73.9, mean = -0.90,  p  = 0). For  MCPH1 -D (right): 73.3% of βs are negative when regressing tone on  MCPH1  alone (one-sided  t -test < 0:  t (999) = -29.3, mean = -0.24,  p  = 7.1e-137), 18.8% when controlling for the macroarea ( t (999) = 28.6, mean = 0.91,  p  = 1), and 14.9% when controlling for both macroarea and  ASPM  ( t (999) = 31.5, mean = 1.19,  p  = 1). 
 
 
 
 
 
  brms  
 
  ASPM  only:
 
  β  = -1.54, 89%HDI = [-4.00, 0.73] 
 posterior probability  p ( β <0) = 0.87 (evidence ratio = 6.6),  p ( β =0) = 0.59 (evidence ratio = 1.4) 
 ROPE = [-0.18, 0.18], % HDI inside ROPE = 7.4%;  p  ROPE  = 0.066 
 comparison ‘null’ vs ‘ASPM’: [B= L= W<(27%:73%) K=]: anecdotal evidence for null against ASPM (BF=1.04), LOO=-0.73 [SE=0.83], WAIC=-0.98 [SE=0.61], KFOLD=0.18 [SE=1.19] 
 comparison ‘null’ vs ‘ASPM’: [B= L= W<(27%:73%) K=]: anecdotal evidence for null against ASPM (BF=1.04), LOO=-0.73 [SE=0.83], WAIC=-0.98 [SE=0.61], KFOLD=0.18 [SE=1.19] 
  
  MCPH1  only:
 
  β  = -1.14, 89%HDI = [-2.96, 0.90] 
 posterior probability  p ( β <0) = 0.84 (evidence ratio = 5.4),  p ( β =0) = 0.64 (evidence ratio = 1.8) 
 ROPE = [-0.18, 0.18], % HDI inside ROPE = 8.3%;  p  ROPE  = 0.074 
 comparison ‘null’ vs ‘MCPH1’: [B= L< W<(30%:70%) K<]: anecdotal evidence for null against MCPH1 (BF=1.47), LOO=-1.02 [SE=0.99], WAIC=-0.86 [SE=0.58], KFOLD=-2.71 [SE=1.77] 
 comparison ‘null’ vs ‘MCPH1’: [B= L< W<(30%:70%) K<]: anecdotal evidence for null against MCPH1 (BF=1.47), LOO=-1.02 [SE=0.99], WAIC=-0.86 [SE=0.58], KFOLD=-2.71 [SE=1.77] 
  
  both alleles :
 
 comparison ‘null’ vs ‘both’: [B= L< W<<(11%:89%) K<]: anecdotal evidence for null against both (BF=2.2), LOO=-1.21 [SE=0.96], WAIC=-2.12 [SE=0.73], KFOLD=-2.65 [SE=1.67] 
 interaction:
 
 posterior probability  p (=0) = 0.68 (evidence ratio = 2.1) 
 ROPE = [-0.18, 0.18], % HDI inside ROPE = 10.1%;  p  ROPE  = 0.09 
 comparison ‘no interaction’ vs ‘with interaction’: [B= L= W<(41%:59%) K=]: anecdotal evidence for no interaction against with interaction (BF=1.83), LOO=-0.03 [SE=0.55], WAIC=-0.34 [SE=0.23], KFOLD=0.69 [SE=0.86] 
  
  ASPM  (partial):
 
  β  = -1.32, 89%HDI = [-3.80, 1.15] 
 posterior probability  p ( β <0) = 0.81 (evidence ratio = 4.3),  p ( β =0) = 0.62 (evidence ratio = 1.6) 
 ROPE = [-0.18, 0.18], % HDI inside ROPE = 7.6%;  p  ROPE  = 0.068 
  
  MCPH1  (partial):
 
  β  = -0.96, 89%HDI = [-2.92, 1.13] 
 posterior probability  p ( β <0) = 0.79 (evidence ratio = 3.7),  p ( β =0) = 0.68 (evidence ratio = 2.1) 
 ROPE = [-0.18, 0.18], % HDI inside ROPE = 10.6%;  p  ROPE  = 0.094 
  
  
 
 
  
 
  Figure 196.    Posterior distributions (with 50% probability mass highlighted) versus 0.0 (the vertical line) for  ASPM -D (left) and  MCPH1 -D (right). 
 
 
 
  
 
  Figure 197.    Conditional effects of  ASPM -D (left) and  MCPH1 -D (right). 
 
 
 
  
 
  Figure 198.    Posterior predictive checks for  ASPM -D (left) and  MCPH1 -D (right). 
 
 
 
  
 
  Figure 199.    Confusion matrices for  ASPM -D (left) and  MCPH1 -D (right). 
 
 
 
 
 
 Mediation and path analysis 
 
 Mediation analysis 
 
  (g)lm  
 
 All data 
 For  ASPM -D: 
 
   total effect  (TE) of being in Africa on tone: 0.32 (0.09, 0.57),  p =0.004, decomposed into:  
   average direct effect  (ADE): 0.14 (-0.07, 0.40),  p =0.23, and  
   average indirect effect  (ACME) mediated by  ASPM -D: 0.19 (0.03, 0.37),  p =0.004, mediating 58.1% (12.2%, 158.4%),  p =0.008 of the effect, resulting from: 
 
 effect of being in Africa on  ASPM -D: -1.34 ±0.26,  p =8.8e-07, and 
 effect of  ASPM -D on tone: -0.92 ±0.38,  p =0.016. 
  
 
 For  MCPH1 -D: 
 
   TE : 0.32 (0.09, 0.57),  p =0, decomposed into:  
   ADE : 0.36 (-0.11, 0.70),  p =0.13, and  
   ACME : -0.04 (-0.32, 0.36),  p =0.74, mediating -21.2% (-179.3%, 161.8%),  p =0.74 of the effect, resulting from: 
 
 effect of being in Africa on  MCPH1 -D: -2.56 ±0.16,  p =7e-31, and 
 effect of  MCPH1 -D on tone: 0.22 ±0.53,  p =0.68. 
  
 
 
 
 Restricted sampling 
 
 
 
  Figure 200.    Mediation analysis for 1000 restricted samples (i.e., picking one random language per family). The leftmost panels show the distribution of point estimates of the Total Effect (TE), the Direct Effect (ADE) and the Indirect Effect (ACME) for  ASPM -D and  MCPH1 -D; the middle panels show the distribution of the  p -values for the same effects, while the rightmost panels show the distribution of the regression slopes ( β ) for the two alleles, top: for the regression of the allele frequency on within vs outside Africa, and bottom: for the regression of tone on the allele while controlling for within vs outside Africa. The black vertical lines show: 0.0 (solid), 0.05 (dashed) and 0.10 (dotted). 
 
 
 For  ASPM -D: 
 
   TE : mean = 0.22, median = 0.23; 2.9% significant at  α -level 0.05 and 15.2% significant at  α -level 0.10; 100.0% > 0.0; one-sample one-sided t-test vs 0:  t (999) = 61.3,  p  = 0;  
   ADE : mean = 0.17, median = 0.18; 0.1% significant at  α -level 0.05 and 3.7% significant at  α -level 0.10; 95.8% > 0.0; one-sample one-sided t-test vs 0:  t (999) = 52.2,  p  = 9e-288;  
   ACME : mean = 0.043, median = 0.042; 0.0% significant at  α -level 0.05 and 0.0% significant at  α -level 0.10; 99.8% > 0.0; one-sample one-sided t-test vs 0:  t (999) = 72.0,  p  = 0;  
   β(Africa → allele) : mean = -0.74, median = -0.74; 9.7% significant at  α -level 0.05 and 65.4% significant at  α -level 0.10; 100.0% < 0.0; one-sample one-sided t-test vs 0:  t (999) = -319.7,  p  = 0;  
   β(allele → tone | Africa) : mean = -0.35, median = -0.35; 0.0% significant at  α -level 0.05 and 0.0% significant at  α -level 0.10; 99.6% < 0.0; one-sample one-sided t-test vs 0:  t (999) = -81.0,  p  = 0.  
 
 For  MCPH1 -D: 
 
   TE : mean = 0.22, median = 0.23; 2.8% significant at  α -level 0.05 and 14.9% significant at  α -level 0.10; 100.0% > 0.0; one-sample one-sided t-test vs 0:  t (999) = 61.3,  p  = 0;  
   ADE : mean = 0.33, median = 0.34; 0.0% significant at  α -level 0.05 and 2.5% significant at  α -level 0.10; 99.3% > 0.0; one-sample one-sided t-test vs 0:  t (999) = 82.5,  p  = 0;  
   ACME : mean = -0.11, median = -0.12; 0.0% significant at  α -level 0.05 and 0.9% significant at  α -level 0.10; 19.1% > 0.0; one-sample one-sided t-test vs 0:  t (999) = -28.4,  p  = 1;  
   β(Africa → allele) : mean = -2.6, median = -2.6; 100.0% significant at  α -level 0.05 and 100.0% significant at  α -level 0.10; 100.0% < 0.0; one-sample one-sided t-test vs 0:  t (999) = -725.0,  p  = 0;  
   β(allele → tone | Africa) : mean = 0.7, median = 0.66; 0.0% significant at  α -level 0.05 and 0.6% significant at  α -level 0.10; 10.7% < 0.0; one-sample one-sided t-test vs 0:  t (999) = 37.7,  p  = 1.  
 
 Given the low sample size  N  = 30 unique families, relatively few effect sizes are big enough to be significant for each individual analysis; however, there are many more significant ACMEs for  ASPM -D than for  MCPH1 -D: 0.0% vs 0.0% (NaN times) for  α -level 0.05, and 0.0% vs 0.6% (0.0 times) for  α -level 0.10. 
 
 
 
  brms  
 
  
 
 
  Figure 201.    Graphical representation of the Bayesian mediation analysis for  ASPM -D showing the means of the effects and the actual partial regression coefficients, with their 89% HDIs and p-ROPEs. The colors reflect the sign of the mean estimate (blue=negative, red=positive, gray=(p-ROPE >= 0.05)); solid=(0 not in the HDI), dashed=(0 is in the HDI). 
 
 
 
  
 
 
  Figure 202.    Graphical representation of the Bayesian mediation analysis for  MCPH1 -D showing the means of the effects and the actual partial regression coefficients, with their 89% HDIs and p-ROPEs. The colors reflect the sign of the mean estimate (blue=negative, red=positive, gray=(p-ROPE >= 0.05)); solid=(0 not in the HDI), dashed=(0 is in the HDI). 
 
 
 
  
 
 
  Figure 203.    Graphical representation of the Bayesian mediation analysis for both  ASPM -D and  MCPH1 -D showing the means of the effects and the actual partial regression coefficients, with their 89% HDIs and p-ROPEs. The colors reflect the sign of the mean estimate (blue=negative, red=positive, gray=(p-ROPE >= 0.05)); solid=(0 not in the HDI), dashed=(0 is in the HDI). 
 
 
 
 
 
 Path analysis 
 
 All data 
 With Africa and  tone1  coded numerically, the model fit is:  χ  2 (1)=0.14,  p =0.71; CFI=1.00, TLI=1.03, NNFI=1.03 and RFI=1.00: 
 
  
 
 
  Figure 204.    Path analysis model with standardised coefficients and significance stars.  tone1  and  macroarea  (Africa vs non-Africa) are coded as numeric binary ( tone_complex_num  with Yes=1 and  Africa_num  with in Africa=1);  ASPM_z  is  ASPM -D and  MCPH1_z  is  MCPH1 -D. 
 
 
 Likewise, with Africa and  tone1  coded as ordered binary factors, the model fit is:  χ  2 (1)=0.43,  p =0.51; CFI=1.00, TLI=1.69, NNFI=1.69 and RFI=0.77: 
 
  
 
 
  Figure 205.    Path analysis model with standardised coefficients and significance stars.  tone1  and  macroarea  (Africa vs non-Africa) are coded as ordered binary factors ( tone_complex_ord  with No < Yes, and  Africa_ord  with outside Africa < in Africa);  ASPM_z  is  ASPM -D and  MCPH1_z  is  MCPH1 -D. 
 
 
 
 
 Restricted sampling 
 
 
 
  Figure 206.    Path analysis for 1000 restricted samples (i.e., picking one random language per family). The leftmost row of two plots shows the coefficient estimates and the  p -values, respectively, for the five paths in the model (see the path plots above). The rightmost plot shows the various fit indices. The black horiontal lines show: 0.0 (solid), 0.05 (dashed) and 1.0 (dotted). 
 
 
 
  models fits: 
 
 84.6% of the  p -values are not significant 
 mean(CFI) = 0.98, median(CFI) = 0.99, sd(CFI) = 0.03, IQR(CFI) = 0.03 
 mean(TLI) = 0.9, median(TLI) = 0.94, sd(TLI) = 0.2, IQR(TLI) = 0.27 
 mean(NNFI) = 0.9, median(NNFI) = 0.94, sd(NNFI) = 0.2, IQR(NNFI) = 0.27 
 mean(RFI) = 0.81, median(RFI) = 0.84, sd(RFI) = 0.17, IQR(RFI) = 0.22 
  
  Africa →  ASPM -D: mean = -0.73, median = -0.74, sd = 0.072, IQR = 0.096, 100.0% < 0; 100.0% significant at  α -level 0.05; one-sample one-sided  t -test vs 0:  t (999) = -3.2e+02,  p  = 0;  
  Africa →  MCPH1 -D: mean = -2.6, median = -2.6, sd = 0.11, IQR = 0.16, 100.0% < 0; 100.0% significant at  α -level 0.05; one-sample one-sided  t -test vs 0:  t (999) = -7.3e+02,  p  = 0;  
  Africa →  tone1 : mean = 0.36, median = 0.37, sd = 0.22, IQR = 0.29, 95.1% > 0; 3.7% significant at  α -level 0.05; one-sample one-sided  t -test vs 0:  t (999) = 53,  p  = 1.7e-293;  
   ASPM -D →  tone1 : mean = -0.041, median = -0.04, sd = 0.027, IQR = 0.036, 93.9% < 0; 0.0% significant at  α -level 0.05; one-sample one-sided  t -test vs 0:  t (999) = -49,  p  = 6.9e-266;  
   MCPH1 -D →  tone1 : mean = 0.067, median = 0.073, as = 0.079, IQR = 0.093, 18.4% < 0; 0.1% significant at  α -level 0.05; one-sample one-sided  t -test vs 0:  t (999) = 27,  p  = 1.  
 
 
 
 
 
 
 Tone  counts  
 The resulting dataset has 121 observations, distributed among 83 unique Glottolg codes in 30 families (ranging from a minimum of 1 language per family to a maximum of 36, with a mean 4 and median 1.5 languages per family) and 4 macroareas. 
 There are 121:95:83 unique samples:(meta)populations:languages retained. 
 
 
 
 
 
 
 
 
 
 
 
   
 Africa 
 Eurasia 
 America 
 Papunesia 
 Sum 
 
 
 
 
  0  
 2 
 77 
 4 
 4 
 87 
 
 
  1  
 2 
 4 
 5 
 0 
 11 
 
 
  2  
 9 
 3 
 0 
 0 
 12 
 
 
  3  
 1 
 2 
 0 
 0 
 3 
 
 
  4  
 0 
 4 
 1 
 0 
 5 
 
 
  5  
 0 
 2 
 0 
 0 
 2 
 
 
  6  
 0 
 1 
 0 
 0 
 1 
 
 
  Sum  
 14 
 93 
 10 
 4 
 121 
 
 
 
 
 
 
  Figure 207.    Distribution of tone  counts . 
 
 
 
 
 
  Figure 208.    Distribution of tone  counts  across the world. 
 
 
 
 
 
  Figure 209.    Relationship between tone  counts  (colors) and the two alleles (frequency) by macroarea. 
 
 
 
 Regressions 
 
  glmer  
 
 All data 
 
  null model : R 2  = 0.0%, ICC = 100.0% 
 the Poisson model is  not  overdispersed:  χ  2 (119) = 60.4,  p  = 1 
  macroarea :  p  macroarea/null  = 0.037 
  ASPM :
 
 by itself: R 2  = 4.5%,  β  = -0.33 ± 0.29,  p  ASPM/null  = 0.28 
 quadratic: R 2  = 34.1%,  β ASPM2   = -0.72 ± 0.41,  p  ASPM2/ASPM  = 0.086 
 with  macroarea : R 2  = 82.1%,  p  macroarea/ASPM  = 0.061,  p  ASPM/macroarea  = 0.82 
  
  MCPH1 :
 
 by itself: R 2  = 5.1%,  β  = -0.37 ± 0.24,  p  MCPH1/null  = 0.12 
 quadratic: R 2  = 6.0%,  β MCPH12   = -0.37 ± 0.24,  p  MCPH12/MCPH1  = 0.38 
 with  macroarea : R 2  = 81.4%,  p  macroarea/MCPH1  = 0.11,  p  MCPH1/macroarea  = 0.92 
  
  both alleles  (no  macroarea ):
 
  ASPM  +  MCPH1 : R 2  = 8.6%,  β  ASPM  = -0.24 ± 0.31,  p  ASPM/MCPH1  = 0.43,  β  MCPH1  = -0.32 ± 0.24,  p  MCPH1/ASPM  = 0.17,  p  ASPM+MCPH1/null  = 0.22, 
 interaction: R 2  = 8.5%,  p  ASPM:MCPH1/ASPM+MCPH1  = 0.94 
  
 
 
 
 Randomization 
 We performed 1000 independent replications: 
 
 Regressions with randomizations for tone  counts . 
 
 
 Permute within 
 Macroarea 
 Permute 
 AIC 
 Signif. 
  p   ASPM -D  
  β   ASPM -D  
  p   MCPH1 -D  
  β   MCPH1 -D  
 
 
 
 
 unrestricted 
 none 
 tone 
 0% 
 22% 
 18% 
 15% 
 15% 
 7% 
 
 
 unrestricted 
 none 
 alleles-together 
 27% 
 6% 
 6% 
 6% 
 6% 
 2% 
 
 
 unrestricted 
 none 
 alleles-independent 
 26% 
 6% 
 6% 
 5% 
 7% 
 0% 
 
 
 unrestricted 
 fixef 
 tone 
 0% 
 28% 
 20% 
 34% 
 19% 
 46% 
 
 
 unrestricted 
 fixef 
 alleles-together 
 98% 
 8% 
 6% 
 30% 
 7% 
 37% 
 
 
 unrestricted 
 fixef 
 alleles-independent 
 98% 
 8% 
 6% 
 30% 
 8% 
 39% 
 
 
 macroareas 
 none 
 tone 
 0% 
 41% 
 22% 
 25% 
 30% 
 25% 
 
 
 macroareas 
 none 
 alleles-together 
 50% 
 18% 
 13% 
 26% 
 12% 
 28% 
 
 
 macroareas 
 none 
 alleles-independent 
 53% 
 17% 
 12% 
 26% 
 16% 
 31% 
 
 
 macroareas 
 fixef 
 tone 
 0% 
 35% 
 25% 
 39% 
 24% 
 42% 
 
 
 macroareas 
 fixef 
 alleles-together 
 98% 
 11% 
 11% 
 34% 
 8% 
 45% 
 
 
 macroareas 
 fixef 
 alleles-independent 
 98% 
 9% 
 11% 
 39% 
 8% 
 45% 
 
 
 families 
 none 
 tone 
 89% 
 34% 
 23% 
 62% 
 17% 
 41% 
 
 
 families 
 none 
 alleles-together 
 50% 
 22% 
 20% 
 54% 
 5% 
 19% 
 
 
 families 
 none 
 alleles-independent 
 58% 
 27% 
 28% 
 62% 
 10% 
 25% 
 
 
 families 
 fixef 
 tone 
 86% 
 11% 
 16% 
 70% 
 2% 
 48% 
 
 
 families 
 fixef 
 alleles-together 
 99% 
 11% 
 15% 
 65% 
 3% 
 37% 
 
 
 families 
 fixef 
 alleles-independent 
 97% 
 10% 
 14% 
 63% 
 5% 
 45% 
 
 
 
 
 
 
Regressions on 1000 permuted data. Each plot shows the original result (vertical dashed black line) and the distribution of the permutations for the three possible things to be permuted (colored curves) for each combination of permutation constraints (horizontal panels) and control for  macroarea  (vertical panels) in terms of the effect size  β ;  ASPM -D is on the left and  MCPH1 -D on the right. The vertical dotted black thin line is at 0.0.
 
 
 
 
 Restricted sampling 
 
 
 
  Figure 210.    Results for 1000 restricted samplings. For  ASPM -D (left): 100% of βs are negative when regressing tone on  ASPM  alone (one-sided  t -test < 0:  t (999) = -141.3, mean = -0.40,  p  = 0), 99.2%, when controlling for the macroarea ( t (999) = -65.0, mean = -0.48,  p  = 0), and 99.3% when controlling for both macroarea and  MCPH1  ( t (999) = -62.1, mean = -0.61,  p  = 0). For  MCPH1 -D (right): 94.4% of βs are negative when regressing tone on  MCPH1  alone (one-sided  t -test < 0:  t (999) = -52.3, mean = -0.14,  p  = 1.8e-288), 1.7% when controlling for the macroarea ( t (999) = 60.7, mean = 0.95,  p  = 1), and 1.3% when controlling for both macroarea and  ASPM  ( t (999) = 54.3, mean = 1.19,  p  = 1). 
 
 
 
 
 
  brms  
 
  ASPM  only:
 
  β  = -0.36, 89%HDI = [-1.06, 0.33] 
 posterior probability  p ( β <0) = 0.8 (evidence ratio = 3.9),  p ( β =0) = 0.84 (evidence ratio = 5.3) 
 ROPE = [-0.10, 0.10], % HDI inside ROPE = 14.1%;  p  ROPE  = 0.126 
 comparison ‘null’ vs ‘ASPM’: [B> L= W=(64%:36%) K=]: moderate evidence for null against ASPM (BF=7.92), LOO=0.10 [SE=0.91], WAIC=0.59 [SE=0.62], KFOLD=0.09 [SE=1.48] 
  
  MCPH1  only:
 
  β  = -0.12, 89%HDI = [-0.81, 0.56] 
 posterior probability  p ( β <0) = 0.63 (evidence ratio = 1.7),  p ( β =0) = 0.88 (evidence ratio = 7.2) 
 ROPE = [-0.10, 0.10], % HDI inside ROPE = 19%;  p  ROPE  = 0.169 
 comparison ‘null’ vs ‘MCPH1’: [B> L= W=(55%:45%) K=]: moderate evidence for null against MCPH1 (BF=7.2), LOO=0.43 [SE=0.49], WAIC=0.20 [SE=0.31], KFOLD=0.28 [SE=1.64] 
  
  both alleles :
 
 comparison ‘null’ vs ‘both’: [B>> L> W>(77%:23%) K=]: very strong evidence for null against both (BF=35.1), LOO=1.24 [SE=0.86], WAIC=1.22 [SE=0.72], KFOLD=0.58 [SE=1.48] 
 interaction:
 
 posterior probability  p (=0) = 0.86 (evidence ratio = 6.3) 
 ROPE = [-0.10, 0.10], % HDI inside ROPE = 16.2%;  p  ROPE  = 0.144 
 comparison ‘no interaction’ vs ‘with interaction’: [B> L<< W<<(32%:68%) K=]: moderate evidence for no interaction against with interaction (BF=7.21), LOO=-1.01 [SE=0.46], WAIC=-0.77 [SE=0.26], KFOLD=0.66 [SE=0.89] 
  
  ASPM  (partial):
 
  β  = -0.3, 89%HDI = [-1.01, 0.40] 
 posterior probability  p ( β <0) = 0.76 (evidence ratio = 3.2),  p ( β =0) = 0.86 (evidence ratio = 6.1) 
 ROPE = [-0.10, 0.10], % HDI inside ROPE = 16.5%;  p  ROPE  = 0.147 
  
  MCPH1  (partial):
 
  β  = -0.07, 89%HDI = [-0.73, 0.60] 
 posterior probability  p ( β <0) = 0.6 (evidence ratio = 1.5),  p ( β =0) = 0.88 (evidence ratio = 7.4) 
 ROPE = [-0.10, 0.10], % HDI inside ROPE = 20%;  p  ROPE  = 0.178 
  
  
 
 
  
 
  Figure 211.    Posterior distributions (with 50% probability mass highlighted) versus 0.0 (the vertical line) for  ASPM -D (left) and  MCPH1 -D (right). 
 
 
 
  
 
  Figure 212.    Conditional effects of  ASPM -D (left) and  MCPH1 -D (right). 
 
 
 
  
 
  Figure 213.    Posterior predictive checks for  ASPM -D (left) and  MCPH1 -D (right). 
 
 
 
 
 Mediation analysis 
 
  (g)lm  
 
 All data 
 For  ASPM -D: 
 
   total effect  (TE) of being in Africa on tone: 1.63 (0.64, 3.36),  p =0, decomposed into:  
   average direct effect  (ADE): 0.43 (-0.21, 1.26),  p =0.19, and  
   average indirect effect  (ACME) mediated by  ASPM -D: 1.20 (0.47, 2.56),  p =0, mediating 73.4% (44.5%, 123.6%),  p =0 of the effect, resulting from: 
 
 effect of being in Africa on  ASPM -D: -1.34 ±0.26,  p =8.8e-07, and 
 effect of  ASPM -D on tone: -0.75 ±0.16,  p =2e-06. 
  
 
 For  MCPH1 -D: 
 
   TE : 1.19 (0.53, 2.06),  p =0, decomposed into:  
   ADE : 3.69 (0.97, 9.95),  p =0, and  
   ACME : -2.50 (-8.35, -0.01),  p =0.05, mediating -165.7% (-811.6%, -0.5%),  p =0.05 of the effect, resulting from: 
 
 effect of being in Africa on  MCPH1 -D: -2.56 ±0.16,  p =7e-31, and 
 effect of  MCPH1 -D on tone: 0.43 ±0.24,  p =0.081. 
  
 
 
 
 Restricted sampling 
 
 
 
  Figure 214.    Mediation analysis for 1000 restricted samples (i.e., picking one random language per family). The leftmost panels show the distribution of point estimates of the Total Effect (TE), the Direct Effect (ADE) and the Indirect Effect (ACME) for  ASPM  and  MCPH1 ; the middle panels show the distribution of the  p -values for the same effects, while the rightmost panels show the distribution of the regression slopes ( β ) for the two alleles, top: for the regression of the allele frequency on within vs outside Africa, and bottom: for the regression of tone on the allele while controlling for within vs outside Africa. The black vertical lines show: 0.0 (dotted), 0.05 (solid) and 0.10 (dashed). 
 
 
 For  ASPM -D: 
 
   TE : mean = 1.2, median = 1.3; 44.2% significant at  α -level 0.05 and 67.0% significant at  α -level 0.10; 100.0% > 0.0; one-sample one-sided t-test vs 0:  t (999) = 116.8,  p  = 0;  
   ADE : mean = 0.89, median = 0.9; 14.8% significant at  α -level 0.05 and 32.0% significant at  α -level 0.10; 99.8% > 0.0; one-sample one-sided t-test vs 0:  t (999) = 86.8,  p  = 0;  
   ACME : mean = 0.35, median = 0.34; 0.0% significant at  α -level 0.05 and 1.2% significant at  α -level 0.10; 99.7% > 0.0; one-sample one-sided t-test vs 0:  t (999) = 72.3,  p  = 0;  
   β(Africa → allele) : mean = -0.73, median = -0.73; 10.3% significant at  α -level 0.05 and 64.3% significant at  α -level 0.10; 100.0% < 0.0; one-sample one-sided t-test vs 0:  t (999) = -310.5,  p  = 0;  
   β(allele → tone | Africa) : mean = -0.29, median = -0.3; 2.8% significant at  α -level 0.05 and 10.2% significant at  α -level 0.10; 99.8% < 0.0; one-sample one-sided t-test vs 0:  t (999) = -93.0,  p  = 0.  
 
 For  MCPH1 -D: 
 
   TE : mean = 1.3, median = 1.3; 37.1% significant at  α -level 0.05 and 61.0% significant at  α -level 0.10; 100.0% > 0.0; one-sample one-sided t-test vs 0:  t (999) = 101.0,  p  = 0;  
   ADE : mean = 43, median = 22; 73.6% significant at  α -level 0.05 and 87.0% significant at  α -level 0.10; 100.0% > 0.0; one-sample one-sided t-test vs 0:  t (999) = 23.8,  p  = 8.4e-100;  
   ACME : mean = -42, median = -21; 50.6% significant at  α -level 0.05 and 66.2% significant at  α -level 0.10; 0.0% > 0.0; one-sample one-sided t-test vs 0:  t (999) = -23.1,  p  = 1;  
   β(Africa → allele) : mean = -2.6, median = -2.6; 100.0% significant at  α -level 0.05 and 100.0% significant at  α -level 0.10; 100.0% < 0.0; one-sample one-sided t-test vs 0:  t (999) = -720.1,  p  = 0;  
   β(allele → tone | Africa) : mean = 0.88, median = 0.87; 50.8% significant at  α -level 0.05 and 67.8% significant at  α -level 0.10; 0.2% < 0.0; one-sample one-sided t-test vs 0:  t (999) = 78.3,  p  = 1.  
 
 Given the low sample size  N  = 35 unique families, relatively few effect sizes are big enough to be significant; however, there are many more significant indirect effects (ACME) for  ASPM -D than for  MCPH1 -D: 2.8% vs 50.8% (0.1 times) for  α -level 0.05, and 10.2% vs 67.8% (0.2 times) for  α -level 0.10. 
 
 
 
  brms  
 
  
 
 
  Figure 215.    Graphical representation of the Bayesian mediation analysis for  ASPM -D showing the means of the effects and the actual partial regression coefficients, with their 89% HDIs and p-ROPEs. The colors reflect the sign of the mean estimate (blue=negative, red=positive, gray=(p-ROPE >= 0.05)); solid=(0 not in the HDI), dashed=(0 is in the HDI). 
 
 
 
  
 
 
  Figure 216.    Graphical representation of the Bayesian mediation analysis for  MCPH1 -D showing the means of the effects and the actual partial regression coefficients, with their 89% HDIs and p-ROPEs. The colors reflect the sign of the mean estimate (blue=negative, red=positive, gray=(p-ROPE >= 0.05)); solid=(0 not in the HDI), dashed=(0 is in the HDI). 
 
 
 
  
 
 
  Figure 217.    Graphical representation of the Bayesian mediation analysis for both  ASPM -D and  MCPH1 -D showing the means of the effects and the actual partial regression coefficients, with their 89% HDIs and p-ROPEs. The colors reflect the sign of the mean estimate (blue=negative, red=positive, gray=(p-ROPE >= 0.05)); solid=(0 not in the HDI), dashed=(0 is in the HDI). 
 
 
 
 
 
 Path analysis 
 Please note that path analysis uses a linear model (so not a Poisson one) for the tone  counts ; also I only use the numeric coding for Africa. 
 
 All data 
 Coding Africa numerically, the model fits the data very well ( χ  2 (1)=0.14,  p =0.71; CFI=1.00, TLI=1.03, NNFI=1.03 and RFI=1.00): 
 
  
 
 
  Figure 218.    Path analysis model with standardised coefficients and significance stars. Here, macroarea (Africa vs non-Africa) is coded as numeric binary ( Africa_num  with in Africa=1);  ASPM_z  is  ASPM -D and  MCPH1_z  is  MCPH1 -D.. 
 
 
 
 
 Restricted sampling 
 
 
 
  Figure 219.    Path analysis for 1000 restricted samples (i.e., picking one random language per family). The leftmost row of two plots shows the coefficient estimates and the  p -values, respectively, for the five paths in the model (see the path plots above). The rightmost plot shows the various fit indices. The black horiontal lines show: 0.0 (solid), 0.05 (dashed) and 1.0 (dotted). 
 
 
 It can be seen that: 
 
  the models fits: 
 
 85.3% of the  p -values are not significant 
 mean(CFI) = 0.98, median(CFI) = 0.99, sd(CFI) = 0.03, IQR(CFI) = 0.04 
 mean(TLI) = 0.91, median(TLI) = 0.93, sd(TLI) = 0.19, IQR(TLI) = 0.28 
 mean(NNFI) = 0.91, median(NNFI) = 0.93, sd(NNFI) = 0.19, IQR(NNFI) = 0.28 
 mean(RFI) = 0.81, median(RFI) = 0.84, sd(RFI) = 0.16, IQR(RFI) = 0.23 
  
  Africa →  ASPM -D: mean = -0.73, median = -0.73, sd = 0.07, IQR = 0.095, 100.0% < 0; 100.0% significant at  α -level 0.05; one-sample one-sided  t -test vs 0:  t (999) = -3.3e+02,  p  = 0  
  Africa →  MCPH1 -D: mean = -2.6, median = -2.6, sd = 0.12, IQR = 0.15, 100.0% < 0; 100.0% significant at  α -level 0.05; one-sample one-sided  t -test vs 0:  t (999) = -7.1e+02,  p  = 0  
  Africa → tone  counts : mean = 2.7, median = 2.7, sd = 0.84, IQR = 1.3, 100.0% > 0; 65.4% significant at  α -level 0.05; one-sample one-sided  t -test vs 0:  t (999) = 1e+02,  p  = 0  
   ASPM -D → tone  counts : mean = -0.17, median = -0.17, sd = 0.12, IQR = 0.16, 92.2% < 0; 0.0% significant at  α -level 0.05; one-sample one-sided  t -test vs 0:  t (999) = -46,  p  = 2.5e-251  
   MCPH1 -D → tone  counts : mean = 0.68, median = 0.68, as = 0.28, IQR = 0.38, 0.8% < 0; 36.1% significant at  α -level 0.05; one-sample one-sided  t -test vs 0:  t (999) = 76,  p  = 1  
 
 
 
 
 
 
 
 Appendix V: Alternative agreement coding of  tone  
 The explicit hierarchy of the sources for  tone  as used in the paper is: 
 
 for  binary  and  3-way :  LAPSyD  >  WALS  >  DL2007  >  WPHON  >  PHOIBLE , and 
 for  counts :  LAPSyD  >  WPHON  >  PHOIBLE  
 
 but there can be other justified choices; among these choices, I test here the alternative hierarchy: 
 
 for  binary  and  3-way :  WALS  >  WPHON  >  LAPSyD  >  DL2007  >  PHOIBLE , and 
 for  counts :  WPHON  >  LaPSyD  >  PHOIBLE . 
 
 The “corrected” counts are computed as  LAPSyD  corr  = 0.074 +1.256 LAPSyD  -0.11 LAPSyD  2 , and  PHOIBLE  corr  = 0.415 +0.815 PHOIBLE  -0.049 PHOIBLE  2 , respectively. 
 
 Relationship between alternative and main agreement codings 
 
 Binary classification 
 
 
 
 
 
 
 
 
   
 No 
 Yes 
 
 
 
 
  No  
 2527 
 14 
 
 
  Yes  
 13 
 1244 
 
 
 
 
 
 
  Figure 220.    Relationship between the agreement and the `alternative’ agreement codings for the binary classification. 
 
 
 
 Pearson’s Chi-squared test with Yates’ continuity correction:  cooc_tab  
 
 
 
 
 
 
 
 Test statistic 
 df 
 P value 
 
 
 
 
 3673 
 1 
 0 * * * 
 
 
 
 
 Pearson’s Chi-squared test with simulated p-value (based on 10000 replicates):  cooc_tab  
 
 
 
 
 
 
 
 Test statistic 
 df 
 P value 
 
 
 
 
 3677 
 NA 
 9.999e-05 * * * 
 
 
 
 The disagreements are: 
 
 The 27 languages for which the original and the alternative binary codings disagree 
 
 
 
 
 
 
 
 
 
 
 
 
 
 
 
 glottocode 
 PHOIBLE 
 WALS 
 LAPSyD 
 LAPSyD (#) 
 DL2007 
 WPHON 
 agreement (orig) 
 decision (orig) 
 agreement (alt) 
 decision (alt) 
 
 
 
 
 amah1246 
 0 
 NA 
 None 
 0 
 NA 
 1 
 No 
 LAPSyD 
 Yes 
 WPHON 
 
 
 beja1238 
 0 
 Simple 
 None 
 0 
 NA 
 1 
 No 
 LAPSyD + WALS, LAPSyD winns except when WALS says Complex 
 Yes 
 WALS 
 
 
 broo1239 
 NA 
 NA 
 None 
 0 
 NA 
 1 
 No 
 LAPSyD 
 Yes 
 WPHON 
 
 
 chua1250 
 0 
 NA 
 None 
 0 
 NA 
 3 
 No 
 LAPSyD 
 Yes 
 WPHON 
 
 
 cofa1242 
 0 
 NA 
 None 
 0 
 NA 
 1 
 No 
 LAPSyD 
 Yes 
 WPHON 
 
 
 fuln1247 
 0 
 Simple 
 None 
 0 
 NA 
 0 
 No 
 LAPSyD + WALS, LAPSyD winns except when WALS says Complex 
 Yes 
 WALS 
 
 
 gras1249 
 0 
 Simple 
 None 
 0 
 NA 
 1 
 No 
 LAPSyD + WALS, LAPSyD winns except when WALS says Complex 
 Yes 
 WALS 
 
 
 hopi1249 
 0 
 Simple 
 None 
 0 
 NA 
 1 
 No 
 LAPSyD + WALS, LAPSyD winns except when WALS says Complex 
 Yes 
 WALS 
 
 
 mand1446 
 NA 
 NA 
 None 
 0 
 NA 
 1 
 No 
 LAPSyD 
 Yes 
 WPHON 
 
 
 meri1244 
 NA 
 NA 
 None 
 0 
 NA 
 1 
 No 
 LAPSyD 
 Yes 
 WPHON 
 
 
 naas1242 
 0 
 NA 
 None 
 0 
 No 
 1 
 No 
 LAPSyD + Dediu & Ladd, Dediu & Ladd winns except when LAPSyD says Moderately complex or Complex 
 Yes 
 WPHON 
 
 
 sapu1248 
 NA 
 NA 
 None 
 0 
 NA 
 1 
 No 
 LAPSyD 
 Yes 
 WPHON 
 
 
 sout2982 
 0 
 NA 
 None 
 0 
 NA 
 1 
 No 
 LAPSyD 
 Yes 
 WPHON 
 
 
 wano1243 
 NA 
 NA 
 None 
 0 
 NA 
 1 
 No 
 LAPSyD 
 Yes 
 WPHON 
 
 
 bora1263 
 0 
 NA 
 Simple 
 1 
 NA 
 0 
 Yes 
 LAPSyD 
 No 
 WPHON 
 
 
 brib1243 
 0 
 NA 
 Simple 
 1 
 NA 
 0 
 Yes 
 LAPSyD 
 No 
 WPHON 
 
 
 buru1296 
 2 
 None 
 NA 
 NA 
 Yes 
 0 
 Yes 
 WALS + Dediu & Ladd, Dediu & Ladd winns except when WALS says Complex 
 No 
 WALS 
 
 
 chim1309 
 0 
 NA 
 Simple 
 1 
 NA 
 0 
 Yes 
 LAPSyD 
 No 
 WPHON 
 
 
 darf1239 
 0 
 None 
 Simple 
 1 
 NA 
 2 
 Yes 
 LAPSyD + WALS, LAPSyD winns except when WALS says Complex 
 No 
 WALS 
 
 
 lepc1244 
 0 
 NA 
 Simple 
 1 
 NA 
 0 
 Yes 
 LAPSyD 
 No 
 WPHON 
 
 
 lith1251 
 0 
 NA 
 Simple 
 1 
 NA 
 0 
 Yes 
 LAPSyD 
 No 
 WPHON 
 
 
 mund1330 
 1 
 NA 
 Simple 
 1 
 NA 
 0 
 Yes 
 LAPSyD 
 No 
 WPHON 
 
 
 scot1245 
 0 
 NA 
 Simple 
 1 
 NA 
 0 
 Yes 
 LAPSyD 
 No 
 WPHON 
 
 
 sene1264 
 0 
 None 
 Simple 
 1 
 NA 
 1 
 Yes 
 LAPSyD + WALS, LAPSyD winns except when WALS says Complex 
 No 
 WALS 
 
 
 shek1245 
 NA 
 NA 
 Complex 
 8 
 NA 
 0 
 Yes 
 LAPSyD 
 No 
 WPHON 
 
 
 wich1260 
 0 
 None 
 Simple 
 1 
 NA 
 1 
 Yes 
 LAPSyD + WALS, LAPSyD winns except when WALS says Complex 
 No 
 WALS 
 
 
 yuru1263 
 0 
 NA 
 Simple 
 1 
 NA 
 0 
 Yes 
 LAPSyD 
 No 
 WPHON 
 
 
 
 
 
 3-way classification 
 
 
 
 
 
 
 
 
 
   
 None 
 Simple 
 Complex 
 
 
 
 
  None  
 2523 
 14 
 1 
 
 
  Simple  
 11 
 922 
 3 
 
 
  Complex  
 1 
 21 
 289 
 
 
 
 
 
 
  Figure 221.    Relationship between the agreement and the `alternative’ agreement codings for the 3-way classification. 
 
 
 
 Pearson’s Chi-squared test:  cooc_tab  
 
 
 
 
 
 
 
 Test statistic 
 df 
 P value 
 
 
 
 
 7027 
 4 
 0 * * * 
 
 
 
 
 Pearson’s Chi-squared test with simulated p-value (based on 10000 replicates):  cooc_tab  
 
 
 
 
 
 
 
 Test statistic 
 df 
 P value 
 
 
 
 
 7027 
 NA 
 9.999e-05 * * * 
 
 
 
 The disagreements are: 
 
 The 51 languages for which the original and the alternative 3-way codings disagree 
 
 
 
 
 
 
 
 
 
 
 
 
 
 
 
 glottocode 
 PHOIBLE 
 WALS 
 LAPSyD 
 LAPSyD (#) 
 DL2007 
 WPHON 
 agreement (orig) 
 decision (orig) 
 agreement (alt) 
 decision (alt) 
 
 
 
 
 amah1246 
 0 
 NA 
 None 
 0 
 NA 
 1 
 None 
 LAPSyD 
 Simple 
 WPHON 
 
 
 beja1238 
 0 
 Simple 
 None 
 0 
 NA 
 1 
 None 
 LAPSyD + WALS, LAPSyD winns except for Moderately complex 
 Simple 
 WALS 
 
 
 broo1239 
 NA 
 NA 
 None 
 0 
 NA 
 1 
 None 
 LAPSyD 
 Simple 
 WPHON 
 
 
 chua1250 
 0 
 NA 
 None 
 0 
 NA 
 3 
 None 
 LAPSyD 
 Simple 
 WPHON 
 
 
 cofa1242 
 0 
 NA 
 None 
 0 
 NA 
 1 
 None 
 LAPSyD 
 Simple 
 WPHON 
 
 
 fuln1247 
 0 
 Simple 
 None 
 0 
 NA 
 0 
 None 
 LAPSyD + WALS, LAPSyD winns except for Moderately complex 
 Simple 
 WALS 
 
 
 gras1249 
 0 
 Simple 
 None 
 0 
 NA 
 1 
 None 
 LAPSyD + WALS, LAPSyD winns except for Moderately complex 
 Simple 
 WALS 
 
 
 hopi1249 
 0 
 Simple 
 None 
 0 
 NA 
 1 
 None 
 LAPSyD + WALS, LAPSyD winns except for Moderately complex 
 Simple 
 WALS 
 
 
 mand1446 
 NA 
 NA 
 None 
 0 
 NA 
 1 
 None 
 LAPSyD 
 Simple 
 WPHON 
 
 
 meri1244 
 NA 
 NA 
 None 
 0 
 NA 
 1 
 None 
 LAPSyD 
 Simple 
 WPHON 
 
 
 naas1242 
 0 
 NA 
 None 
 0 
 No 
 1 
 None 
 LAPSyD wins 
 Simple 
 WPHON 
 
 
 sapu1248 
 NA 
 NA 
 None 
 0 
 NA 
 1 
 None 
 LAPSyD 
 Simple 
 WPHON 
 
 
 sout2982 
 0 
 NA 
 None 
 0 
 NA 
 1 
 None 
 LAPSyD 
 Simple 
 WPHON 
 
 
 wano1243 
 NA 
 NA 
 None 
 0 
 NA 
 1 
 None 
 LAPSyD 
 Simple 
 WPHON 
 
 
 ndut1239 
 0 
 Complex 
 None 
 0 
 NA 
 0 
 None 
 LAPSyD + WALS, LAPSyD winns except for Moderately complex 
 Complex 
 WALS 
 
 
 bora1263 
 0 
 NA 
 Simple 
 1 
 NA 
 0 
 Simple 
 LAPSyD 
 None 
 WPHON 
 
 
 brib1243 
 0 
 NA 
 Simple 
 1 
 NA 
 0 
 Simple 
 LAPSyD 
 None 
 WPHON 
 
 
 chim1309 
 0 
 NA 
 Simple 
 1 
 NA 
 0 
 Simple 
 LAPSyD 
 None 
 WPHON 
 
 
 darf1239 
 0 
 None 
 Simple 
 1 
 NA 
 2 
 Simple 
 LAPSyD + WALS, LAPSyD winns except for Moderately complex 
 None 
 WALS 
 
 
 lepc1244 
 0 
 NA 
 Simple 
 1 
 NA 
 0 
 Simple 
 LAPSyD 
 None 
 WPHON 
 
 
 lith1251 
 0 
 NA 
 Simple 
 1 
 NA 
 0 
 Simple 
 LAPSyD 
 None 
 WPHON 
 
 
 mund1330 
 1 
 NA 
 Simple 
 1 
 NA 
 0 
 Simple 
 LAPSyD 
 None 
 WPHON 
 
 
 scot1245 
 0 
 NA 
 Simple 
 1 
 NA 
 0 
 Simple 
 LAPSyD 
 None 
 WPHON 
 
 
 sene1264 
 0 
 None 
 Simple 
 1 
 NA 
 1 
 Simple 
 LAPSyD + WALS, LAPSyD winns except for Moderately complex 
 None 
 WALS 
 
 
 wich1260 
 0 
 None 
 Simple 
 1 
 NA 
 1 
 Simple 
 LAPSyD + WALS, LAPSyD winns except for Moderately complex 
 None 
 WALS 
 
 
 yuru1263 
 0 
 NA 
 Simple 
 1 
 NA 
 0 
 Simple 
 LAPSyD 
 None 
 WPHON 
 
 
 east2652 
 1 
 Complex 
 Simple 
 1 
 NA 
 1 
 Simple 
 LAPSyD + WALS, LAPSyD winns except for Moderately complex 
 Complex 
 WALS 
 
 
 nort2740 
 0 
 NA 
 Simple 
 1 
 NA 
 4 
 Simple 
 LAPSyD 
 Complex 
 WPHON 
 
 
 vani1248 
 0 
 Complex 
 Simple 
 1 
 NA 
 2 
 Simple 
 LAPSyD + WALS, LAPSyD winns except for Moderately complex 
 Complex 
 WALS 
 
 
 shek1245 
 NA 
 NA 
 Complex 
 8 
 NA 
 0 
 Complex 
 LAPSyD 
 None 
 WPHON 
 
 
 abun1252 
 NA 
 NA 
 Moderately complex 
 2 
 NA 
 1 
 Complex 
 LAPSyD 
 Simple 
 WPHON 
 
 
 bamu1253 
 0 
 NA 
 NA 
 NA 
 Yes 
 3 
 Complex 
 From n_tones 
 Simple 
 WPHON 
 
 
 bass1258 
 NA 
 NA 
 Moderately complex 
 3 
 NA 
 2 
 Complex 
 LAPSyD 
 Simple 
 WPHON 
 
 
 cacu1241 
 0 
 Simple 
 Complex 
 3 
 NA 
 3 
 Complex 
 LAPSyD + WALS, LAPSyD winns except for Moderately complex 
 Simple 
 WALS 
 
 
 cent2144 
 2 
 NA 
 Complex 
 3 
 NA 
 2 
 Complex 
 LAPSyD 
 Simple 
 WPHON 
 
 
 diga1241 
 NA 
 NA 
 Complex 
 3 
 NA 
 3 
 Complex 
 LAPSyD 
 Simple 
 WPHON 
 
 
 gaam1241 
 2 
 Simple 
 Complex 
 3 
 NA 
 1 
 Complex 
 LAPSyD + WALS, LAPSyD winns except for Moderately complex 
 Simple 
 WALS 
 
 
 hlai1239 
 NA 
 NA 
 Moderately complex 
 2 
 NA 
 3 
 Complex 
 LAPSyD 
 Simple 
 WPHON 
 
 
 jeme1245 
 0 
 NA 
 Moderately complex 
 2 
 NA 
 3 
 Complex 
 LAPSyD 
 Simple 
 WPHON 
 
 
 jica1244 
 2 
 NA 
 Moderately complex 
 2 
 NA 
 1 
 Complex 
 LAPSyD 
 Simple 
 WPHON 
 
 
 kala1373 
 NA 
 Simple 
 Complex 
 4 
 NA 
 1 
 Complex 
 LAPSyD + WALS, LAPSyD winns except for Moderately complex 
 Simple 
 WALS 
 
 
 kris1246 
 3 
 NA 
 Complex 
 3 
 NA 
 3 
 Complex 
 LAPSyD 
 Simple 
 WPHON 
 
 
 lele1276 
 2 
 NA 
 Moderately complex 
 2 
 NA 
 2 
 Complex 
 LAPSyD 
 Simple 
 WPHON 
 
 
 madi1260 
 3 
 NA 
 Moderately complex 
 2 
 Yes 
 2 
 Complex 
 LAPSyD wins 
 Simple 
 WPHON 
 
 
 nort2732 
 NA 
 NA 
 NA 
 NA 
 Yes 
 3 
 Complex 
 From n_tones 
 Simple 
 WPHON 
 
 
 nucl1620 
 0 
 NA 
 Moderately complex 
 2 
 NA 
 2 
 Complex 
 LAPSyD 
 Simple 
 WPHON 
 
 
 puin1248 
 0 
 NA 
 Complex 
 3 
 NA 
 1 
 Complex 
 LAPSyD 
 Simple 
 WPHON 
 
 
 sand1273 
 4 
 Simple 
 Complex 
 3 
 Yes 
 2 
 Complex 
 LAPSyD wins 
 Simple 
 WALS 
 
 
 xhos1239 
 1 
 NA 
 NA 
 NA 
 Yes 
 2 
 Complex 
 From n_tones 
 Simple 
 WPHON 
 
 
 yaka1272 
 NA 
 NA 
 NA 
 NA 
 Yes 
 2 
 Complex 
 From n_tones 
 Simple 
 WPHON 
 
 
 yuhu1238 
 0 
 NA 
 Complex 
 3 
 NA 
 1 
 Complex 
 LAPSyD 
 Simple 
 WPHON 
 
 
 
 
 
 Counts 
 
 
 
  Figure 222.    Relationship between the agreement and the `alternative’ agreement codings for the counts. 
 
 
 
 Pearson’s product-moment correlation:  agreement  and  agreement_alt  
 
 
 
 
 
 
 
 
 
 Test statistic 
 df 
 P value 
 Alternative hypothesis 
 cor 
 
 
 
 
 164.8 
 3783 
 0 * * * 
 two.sided 
 0.9369 
 
 
 
 
 Spearman’s rank correlation rho:  agreement  and  agreement_alt  
 
 
 
 
 
 
 
 
 Test statistic 
 P value 
 Alternative hypothesis 
 rho 
 
 
 
 
 193189299 
 0 * * * 
 two.sided 
 0.9786 
 
 
 
 Look at the serious disagreements (i.e., more than 1): 
 
 The 59 languages for which the original and the alternative count codings disagree, ordered by their absolute difference 
 
 
 
 
 
 
 
 
 
 
 
 
 
 
 glottocode 
 PHOIBLE 
 WALS 
 LAPSyD 
 LAPSyD (#) 
 DL2007 
 WPHON 
 agreement (orig) 
 agreement (alt) 
 difference (abs) 
 
 
 
 
 achu1247 
 0 
 Simple 
 Simple 
 1 
 NA 
 3 
 1 
 3 
 2 
 
 
 anga1290 
 0 
 Simple 
 Moderately complex 
 2 
 NA 
 0 
 2 
 0 
 2 
 
 
 awng1244 
 6 
 Simple 
 Simple 
 1 
 NA 
 3 
 1 
 3 
 2 
 
 
 cent2050 
 3 
 Simple 
 Simple 
 1 
 NA 
 3 
 1 
 3 
 2 
 
 
 efik1245 
 0 
 Simple 
 Moderately complex 
 2 
 NA 
 4 
 2 
 4 
 2 
 
 
 gaam1241 
 2 
 Simple 
 Complex 
 3 
 NA 
 1 
 3 
 1 
 2 
 
 
 gads1258 
 3 
 Complex 
 Complex 
 3 
 NA 
 1 
 3 
 1 
 2 
 
 
 hmon1333 
 NA 
 NA 
 NA 
 NA 
 NA 
 7 
 5 
 7 
 2 
 
 
 iumi1238 
 3 
 Complex 
 NA 
 NA 
 NA 
 7 
 5 
 7 
 2 
 
 
 kera1255 
 0 
 Complex 
 Moderately complex 
 2 
 NA 
 0 
 2 
 0 
 2 
 
 
 komc1235 
 6 
 NA 
 NA 
 NA 
 NA 
 7 
 5 
 7 
 2 
 
 
 koro1298 
 NA 
 None 
 None 
 0 
 NA 
 2 
 0 
 2 
 2 
 
 
 koyr1240 
 5 
 None 
 None 
 0 
 NA 
 2 
 0 
 2 
 2 
 
 
 kuta1241 
 NA 
 NA 
 NA 
 NA 
 NA 
 6 
 4 
 6 
 2 
 
 
 lamn1239 
 6 
 NA 
 NA 
 NA 
 NA 
 7 
 5 
 7 
 2 
 
 
 larg1235 
 NA 
 NA 
 NA 
 NA 
 NA 
 6 
 4 
 6 
 2 
 
 
 mind1253 
 0 
 Complex 
 NA 
 NA 
 NA 
 7 
 5 
 7 
 2 
 
 
 mruu1242 
 NA 
 NA 
 NA 
 NA 
 NA 
 7 
 5 
 7 
 2 
 
 
 murl1244 
 0 
 Simple 
 Simple 
 2 
 NA 
 4 
 2 
 4 
 2 
 
 
 nama1264 
 0 
 Complex 
 Complex 
 5 
 Yes 
 3 
 5 
 3 
 2 
 
 
 ncan1245 
 5 
 NA 
 NA 
 NA 
 NA 
 6 
 4 
 6 
 2 
 
 
 ngba1285 
 0 
 NA 
 NA 
 NA 
 NA 
 6 
 4 
 6 
 2 
 
 
 nort2747 
 NA 
 NA 
 NA 
 NA 
 NA 
 7 
 5 
 7 
 2 
 
 
 nort2819 
 2 
 Complex 
 NA 
 NA 
 NA 
 6 
 4 
 6 
 2 
 
 
 nucl1649 
 0 
 None 
 None 
 0 
 NA 
 2 
 0 
 2 
 2 
 
 
 nucl1770 
 NA 
 NA 
 NA 
 NA 
 NA 
 7 
 5 
 7 
 2 
 
 
 nung1283 
 0 
 Complex 
 Complex 
 5 
 NA 
 3 
 5 
 3 
 2 
 
 
 pira1253 
 0 
 Simple 
 Simple 
 1 
 NA 
 3 
 1 
 3 
 2 
 
 
 puin1248 
 0 
 NA 
 Complex 
 3 
 NA 
 1 
 3 
 1 
 2 
 
 
 puxi1243 
 NA 
 NA 
 NA 
 NA 
 NA 
 6 
 4 
 6 
 2 
 
 
 pwon1235 
 4 
 Complex 
 Complex 
 3 
 NA 
 5 
 3 
 5 
 2 
 
 
 smal1236 
 NA 
 NA 
 NA 
 NA 
 NA 
 7 
 5 
 7 
 2 
 
 
 sout2741 
 NA 
 NA 
 NA 
 NA 
 NA 
 6 
 4 
 6 
 2 
 
 
 sout2754 
 NA 
 NA 
 NA 
 NA 
 NA 
 7 
 5 
 7 
 2 
 
 
 sout2844 
 0 
 NA 
 NA 
 NA 
 NA 
 6 
 4 
 6 
 2 
 
 
 tain1252 
 0 
 NA 
 NA 
 NA 
 Yes 
 6 
 4 
 6 
 2 
 
 
 thak1245 
 0 
 NA 
 Simple 
 1 
 NA 
 3 
 1 
 3 
 2 
 
 
 timn1235 
 3 
 Simple 
 Simple 
 1 
 NA 
 3 
 1 
 3 
 2 
 
 
 veng1238 
 8 
 NA 
 NA 
 NA 
 NA 
 7 
 5 
 7 
 2 
 
 
 yako1252 
 NA 
 NA 
 NA 
 NA 
 NA 
 6 
 4 
 6 
 2 
 
 
 youn1235 
 NA 
 NA 
 NA 
 NA 
 NA 
 7 
 5 
 7 
 2 
 
 
 yuhu1238 
 0 
 NA 
 Complex 
 3 
 NA 
 1 
 3 
 1 
 2 
 
 
 bero1242 
 0 
 Complex 
 Complex 
 6 
 NA 
 3 
 6 
 3 
 3 
 
 
 chua1250 
 0 
 NA 
 None 
 0 
 NA 
 3 
 0 
 3 
 3 
 
 
 kala1373 
 NA 
 Simple 
 Complex 
 4 
 NA 
 1 
 4 
 1 
 3 
 
 
 lada1244 
 0 
 None 
 None 
 0 
 NA 
 3 
 0 
 3 
 3 
 
 
 mmen1238 
 4 
 NA 
 NA 
 NA 
 NA 
 8 
 5 
 8 
 3 
 
 
 nige1255 
 1 
 Complex 
 NA 
 NA 
 NA 
 8 
 5 
 8 
 3 
 
 
 nort2740 
 0 
 NA 
 Simple 
 1 
 NA 
 4 
 1 
 4 
 3 
 
 
 ticu1245 
 8 
 Complex 
 Complex 
 4 
 NA 
 7 
 4 
 7 
 3 
 
 
 aghe1239 
 1 
 Simple 
 Simple 
 1 
 NA 
 5 
 1 
 5 
 4 
 
 
 cent1394 
 NA 
 NA 
 Complex 
 3 
 NA 
 7 
 3 
 7 
 4 
 
 
 ejag1239 
 4 
 Complex 
 Complex 
 5 
 NA 
 1 
 5 
 1 
 4 
 
 
 monz1249 
 NA 
 NA 
 NA 
 NA 
 NA 
 9 
 5 
 9 
 4 
 
 
 gban1258 
 NA 
 NA 
 NA 
 NA 
 NA 
 10 
 5 
 10 
 5 
 
 
 vute1244 
 4 
 NA 
 NA 
 NA 
 NA 
 10 
 5 
 10 
 5 
 
 
 niel1243 
 2 
 Complex 
 NA 
 NA 
 NA 
 11 
 5 
 11 
 6 
 
 
 mali1285 
 NA 
 NA 
 Complex 
 10 
 NA 
 NA 
 10 
 2 
 8 
 
 
 shek1245 
 NA 
 NA 
 Complex 
 8 
 NA 
 0 
 8 
 0 
 8 
 
 
 
 
 
 Conclusion 
 So, the “original” and the “alternative” codings agree rather well… 
 
 
 
 Stats 
 Here, I re-do that stats using the “alternative” coding. 
 
  tone1  
 There are 181 observations, distributed among 119 unique Glottolg codes in 35 families (ranging from a minimum of 1 language per family to a maximum of 48, with a mean 5.2 and median 2 languages per family) and 4 macroareas. 
 There are 161:126:119 unique samples:(meta)populations:languages retained. 
 
 
 
 
 
 
 
 
 
 
 
   
 Africa 
 Eurasia 
 America 
 Papunesia 
 Sum 
 
 
 
 
  No  
 9 
 101 
 4 
 6 
 120 
 
 
  Yes  
 27 
 25 
 6 
 3 
 61 
 
 
  Sum  
 36 
 126 
 10 
 9 
 181 
 
 
 
 
 
 
  Figure 223.    Distribution of  tone1 . 
 
 
 
 
 
  Figure 224.    Map of  tone1 . 
 
 
 
 
 
  Figure 225.    Relationship between  tone1 ,  ASPM -D and  MCPH1 -D. 
 
 
 The agreement with the original  tone1  coding is extremely high: 
 
 
 
 
 
 
 
 
   
 No 
 Yes 
 
 
 
 
  No  
 119 
 1 
 
 
  Yes  
 1 
 60 
 
 
 
 
 
 
  Figure 226.    Relationship between the agreement and the `alternative’ agreement codings for  tone1 . 
 
 
 
 Pearson’s Chi-squared test with Yates’ continuity correction:  cooc_tab  
 
 
 
 
 
 
 
 Test statistic 
 df 
 P value 
 
 
 
 
 167.8 
 1 
 2.212e-38 * * * 
 
 
 
 
 Pearson’s Chi-squared test with simulated p-value (based on 10000 replicates):  cooc_tab  
 
 
 
 
 
 
 
 Test statistic 
 df 
 P value 
 
 
 
 
 172.2 
 NA 
 9.999e-05 * * * 
 
 
 
 The disagreements are: 
 
 The 2 languages for which the original and the alternative  tone1  disagree 
 
 
 glottocode 
 Pop_ID 
 metapopulation 
 family 
 macroarea 
 original 
 alternative 
 
 
 
 
 naas1242 
 SA002261L 
 Melanesian_Nasioi 
 South Bougainville 
 Papunesia 
 No 
 Yes 
 
 
 buru1296 
 SA001482P 
 Burusho 
 Burushaski 
 Eurasia 
 Yes 
 No 
 
 
 
 so I expect the results of the analysis to be virtually identical… 
 
 
  tone2  
 The resulting dataset has 180 observations, distributed among 118 unique Glottolg codes in 35 families (ranging from a minimum of 1 language per family to a maximum of 47, with a mean 5.1 and median 2 languages per family) and 4 macroareas. 
 There are 156:121:118 unique samples:(meta)populations:languages retained. 
 
 
 
 
 
 
 
 
 
 
 
   
 Africa 
 Eurasia 
 America 
 Papunesia 
 Sum 
 
 
 
 
  No  
 31 
 106 
 9 
 9 
 155 
 
 
  Yes  
 6 
 17 
 1 
 1 
 25 
 
 
  Sum  
 37 
 123 
 10 
 10 
 180 
 
 
 
 
 
 
  Figure 227.    Distribution of  tone2 . 
 
 
 
 
 
  Figure 228.    Map of  tone2 . 
 
 
 
 
 
  Figure 229.    Relationship between  tone2 ,  ASPM -D and  MCPH1 -D. 
 
 
 The agreement with the original  tone2  coding is extremely high: 
 
 
 
 
 
 
 
 
   
 No 
 Yes 
 
 
 
 
  No  
 151 
 0 
 
 
  Yes  
 4 
 25 
 
 
 
 
 
 
  Figure 230.    Relationship between the agreement and the `alternative’ agreement codings for  tone2 . 
 
 
 
 Pearson’s Chi-squared test with Yates’ continuity correction:  cooc_tab  
 
 
 
 
 
 
 
 Test statistic 
 df 
 P value 
 
 
 
 
 144 
 1 
 3.472e-33 * * * 
 
 
 
 
 Pearson’s Chi-squared test with simulated p-value (based on 10000 replicates):  cooc_tab  
 
 
 
 
 
 
 
 Test statistic 
 df 
 P value 
 
 
 
 
 151.2 
 NA 
 9.999e-05 * * * 
 
 
 
 The disagreements are: 
 
 The 4 languages for which the original and the alternative  tone2  disagree 
 
 
 glottocode 
 Pop_ID 
 metapopulation 
 family 
 macroarea 
 original 
 alternative 
 
 
 
 
 bamu1253 
 MB2005_Bamoun 
 Bamoun 
 Atlantic-Congo 
 Africa 
 Yes 
 No 
 
 
 sand1273 
 SA004366T 
 Sandawe 
 Sandawe 
 Africa 
 Yes 
 No 
 
 
 nort2732 
 SA001484R 
 Tujia 
 Sino-Tibetan 
 Eurasia 
 Yes 
 No 
 
 
 yaka1272 
 SA002256P 
 Biaka 
 Atlantic-Congo 
 Africa 
 Yes 
 No 
 
 
 
 so I expect the results of the analysis to be very similar… 
 
 
 Tone  counts  
 The resulting dataset has 183 observations, distributed among 120 unique Glottolg codes in 35 families (ranging from a minimum of 1 language per family to a maximum of 47, with a mean 5.2 and median 2 languages per family) and 4 macroareas. 
 There are 156:121:120 unique samples:(meta)populations:languages retained. 
 
 
 
 
 
 
 
 
 
 
 
   
 Africa 
 Eurasia 
 America 
 Papunesia 
 Sum 
 
 
 
 
  0  
 9 
 97 
 4 
 6 
 116 
 
 
  1  
 9 
 7 
 5 
 2 
 23 
 
 
  2  
 15 
 1 
 0 
 1 
 17 
 
 
  3  
 4 
 5 
 0 
 1 
 10 
 
 
  4  
 1 
 3 
 0 
 0 
 4 
 
 
  5  
 0 
 8 
 0 
 0 
 8 
 
 
  6  
 0 
 3 
 0 
 0 
 3 
 
 
  7  
 0 
 1 
 1 
 0 
 2 
 
 
  Sum  
 38 
 125 
 10 
 10 
 183 
 
 
 
 
 
 
  Figure 231.    Distribution of tone  counts . 
 
 
 
 
 
  Figure 232.    Distribution of tone  counts  across the world. 
 
 
 
 
 
  Figure 233.    Relationship between tone  counts  (colors) and the two alleles (frequency) by macroarea. 
 
 
 The original and alternative tone  counts  are very similar: 
 
 
 
  Figure 234.    Relationship between the agreement and the `alternative’ agreement for tone  counts . 
 
 
 
 Pearson’s product-moment correlation:  n_tones_orig  and  n_tones_alt  
 
 
 
 
 
 
 
 
 
 Test statistic 
 df 
 P value 
 Alternative hypothesis 
 cor 
 
 
 
 
 38.96 
 181 
 6.161e-90 * * * 
 two.sided 
 0.9452 
 
 
 
 
 Spearman’s rank correlation rho:  n_tones_orig  and  n_tones_alt  
 
 
 
 
 
 
 
 
 Test statistic 
 P value 
 Alternative hypothesis 
 rho 
 
 
 
 
 17364 
 3.499e-135 * * * 
 two.sided 
 0.983 
 
 
 
 Look at the serious disagreements (i.e., more than 1): 
 
 The 6 languages for which the original and the alternative tone  counts  disagree by more than 1 
 
 
 
 
 
 
 
 
 
 
 
 
 glottocode 
 Pop_ID 
 metapopulation 
 family 
 macroarea 
 original 
 alternative 
 difference 
 
 
 
 
 awng1244 
 SA004368V 
 Jews_Ethiopian 
 Afro-Asiatic 
 Africa 
 1 
 3 
 2 
 
 
 nama1264 
 SA001469U 
 San 
 Khoe-Kwadi 
 Africa 
 5 
 3 
 2 
 
 
 tain1252 
 SA001493R 
 Dai 
 Tai-Kadai 
 Eurasia 
 4 
 6 
 2 
 
 
 tain1252 
 SA004238R 
 Dai 
 Tai-Kadai 
 Eurasia 
 4 
 6 
 2 
 
 
 ticu1245 
 SA004389Y 
 Ticuna 
 Ticuna-Yuri 
 America 
 4 
 7 
 3 
 
 
 cent1394 
 SA001486T 
 Miao 
 Hmong-Mien 
 Eurasia 
 3 
 7 
 4 
 
 
 
 so I expect the results of the analysis to be very similar… 
 
 
 
 
 Appendix IV: Macroareas as units of analysis 
 
 Excluding Africa 
 Here I exclude from the analysis all the African data points. 
 
  tone1  
 There are 145 observations, distributed among 89 unique Glottolg codes in 28 families (ranging from a minimum of 1 language per family to a maximum of 48, with a mean 5.2 and median 2 languages per family) and 3 macroareas. 
 There are 134:102:89 unique samples:(meta)populations:languages retained. 
 
 
 
 
 
 
 
 
 
 
   
 America 
 Eurasia 
 Papunesia 
 Sum 
 
 
 
 
  No  
 4 
 100 
 7 
 111 
 
 
  Yes  
 6 
 26 
 2 
 34 
 
 
  Sum  
 10 
 126 
 9 
 145 
 
 
 
 
 
 
  Figure 235.    Distribution of  tone1 . 
 
 
 
 
 
  Figure 236.    Map of  tone1 . 
 
 
 
 
 
  Figure 237.    Relationship between  tone1 ,  ASPM -D and  MCPH1 -D. 
 
 
 
 Regressions 
 
  glmer  
 
 All data 
 
  null model : R 2  = 0.0%, ICC = 85.6% 
  macroarea :  p  macroarea/null  = 0.2 
  ASPM :
 
 by itself: R 2  = 0.3%,  β  = -0.23 ± 0.52,  p  ASPM/null  = 0.67 
 quadratic: R 2  = 1.6%,  β ASPM2   = -0.14 ± 0.72,  p  ASPM2/ASPM  = 0.28 
 with  macroarea : R 2  = 9.2%,  p  macroarea/ASPM  = 0.2,  p  ASPM/macroarea  = 0.71 
  
  MCPH1 :
 
 by itself: R 2  = 0.0%,  β  = 0.11 ± 0.42,  p  MCPH1/null  = 0.8 
 quadratic: R 2  = 0.7%,  β MCPH12   = 0.07 ± 0.48,  p  MCPH12/MCPH1  = 0.18 
 with  macroarea : R 2  = 9.3%,  p  macroarea/MCPH1  = 0.2,  p  MCPH1/macroarea  = 0.91 
  
  both alleles  (no  macroarea ):
 
  ASPM  +  MCPH1 : R 2  = 0.7%,  β  ASPM  = -0.30 ± 0.54,  p  ASPM/MCPH1  = 0.59,  β  MCPH1  = 0.17 ± 0.41,  p  MCPH1/ASPM  = 0.67,  p  ASPM+MCPH1/null  = 0.83, 
 interaction: R 2  = 2.1%,  p  ASPM:MCPH1/ASPM+MCPH1  = 0.27 
  
 
 
 
 Randomization 
 I performed 1000 independent replications of each of these parameter combinations, and below are the distributions of the permuted values versus the original ones (i.e., those obtained on the original, non-permuted data). 
 
 Regressions on 1000 permuted data. The first 3 columns show the permutation constraints (if any), how the  macroarea  is considered (if at all), and what is permuted. The next columns show the percent of the permutations that, in order, have a better AIC compared to the original fit, are significantly better than the null model (thus testing the effect of both alleles simultaneously), have a significant effect of  ASPM -D, have a smaller effect ( β ) of  ASPM -D than the original fit, and the same for  MCPH1 -D. 
 
 
 Permute within 
 Macroarea 
 Permute 
 AIC 
 Signif. 
  p   ASPM -D  
  β   ASPM -D  
  p   MCPH1 -D  
  β   MCPH1 -D  
 
 
 
 
 unrestricted 
 none 
 tone 
 0% 
 4% 
 4% 
 6% 
 5% 
 79% 
 
 
 unrestricted 
 none 
 alleles-together 
 85% 
 7% 
 7% 
 22% 
 8% 
 64% 
 
 
 unrestricted 
 none 
 alleles-independent 
 84% 
 7% 
 7% 
 22% 
 6% 
 64% 
 
 
 unrestricted 
 fixef 
 tone 
 0% 
 5% 
 4% 
 84% 
 5% 
 45% 
 
 
 unrestricted 
 fixef 
 alleles-together 
 93% 
 9% 
 9% 
 66% 
 7% 
 46% 
 
 
 unrestricted 
 fixef 
 alleles-independent 
 94% 
 6% 
 6% 
 70% 
 6% 
 46% 
 
 
 macroareas 
 none 
 tone 
 0% 
 19% 
 15% 
 33% 
 13% 
 47% 
 
 
 macroareas 
 none 
 alleles-together 
 87% 
 7% 
 7% 
 36% 
 7% 
 52% 
 
 
 macroareas 
 none 
 alleles-independent 
 87% 
 8% 
 7% 
 36% 
 9% 
 51% 
 
 
 macroareas 
 fixef 
 tone 
 0% 
 5% 
 6% 
 82% 
 6% 
 47% 
 
 
 macroareas 
 fixef 
 alleles-together 
 94% 
 7% 
 7% 
 65% 
 6% 
 46% 
 
 
 macroareas 
 fixef 
 alleles-independent 
 93% 
 7% 
 7% 
 69% 
 6% 
 48% 
 
 
 families 
 none 
 tone 
 88% 
 6% 
 7% 
 49% 
 3% 
 53% 
 
 
 families 
 none 
 alleles-together 
 91% 
 9% 
 10% 
 62% 
 5% 
 44% 
 
 
 families 
 none 
 alleles-independent 
 90% 
 9% 
 10% 
 57% 
 6% 
 55% 
 
 
 families 
 fixef 
 tone 
 81% 
 5% 
 5% 
 73% 
 2% 
 38% 
 
 
 families 
 fixef 
 alleles-together 
 93% 
 6% 
 7% 
 78% 
 3% 
 37% 
 
 
 families 
 fixef 
 alleles-independent 
 92% 
 5% 
 6% 
 75% 
 5% 
 47% 
 
 
 
 
 
 
Regressions on 1000 permuted data. Each plot shows the original result (vertical dashed black line) and the distribution of the permutations for the three possible things to be permuted (colored curves) for each combination of permutation constraints (horizontal panels) and control for  macroarea  (vertical panels) in terms of the effect size  β ;  ASPM -D is on the left and  MCPH1 -D on the right. The vertical dotted black thin line is at 0.0.
 
 
 
 
 Restricted sampling 
 
 
 
  Figure 238.    Results for 1000 restricted samplings. For  ASPM -D (left): 99.9% of βs are negative when regressing tone on  ASPM  alone (one-sided  t -test < 0:  t (999) = -66.0, mean = -0.71,  p  = 0), 95.2%, when controlling for the macroarea ( t (999) = -42.2, mean = -0.65,  p  = 1.2e-224), and 95.5% when controlling for both macroarea and  MCPH1  ( t (999) = -33.2, mean = -0.89,  p  = 8.2e-164). For  MCPH1 -D (right): 32.1% of βs are negative when regressing tone on  MCPH1  alone (one-sided  t -test < 0:  t (999) = 15.3, mean = 0.13,  p  = 1), 87.6% when controlling for the macroarea ( t (999) = -35.5, mean = -0.50,  p  = 1.2e-179), and 86.9% when controlling for both macroarea and  ASPM  ( t (999) = -30.3, mean = -0.80,  p  = 6e-144). 
 
 
 
 
 
  brms  
 
  ASPM  only:
 
  β  = -0.19, 89%HDI = [-1.54, 1.17] 
 posterior probability  p ( β <0) = 0.6 (evidence ratio = 1.5),  p ( β =0) = 0.8 (evidence ratio = 4.1) 
 ROPE = [-0.18, 0.18], % HDI inside ROPE = 19.9%;  p  ROPE  = 0.177 
 comparison ‘null’ vs ‘ASPM’: [B> L= W=(43%:57%) K=]: moderate evidence for null against ASPM (BF=3.3), LOO=0.23 [SE=0.70], WAIC=-0.29 [SE=0.32], KFOLD=0.74 [SE=0.91] 
 comparison ‘null’ vs ‘ASPM’: [B> L= W=(43%:57%) K=]: moderate evidence for null against ASPM (BF=3.3), LOO=0.23 [SE=0.70], WAIC=-0.29 [SE=0.32], KFOLD=0.74 [SE=0.91] 
  
  MCPH1  only:
 
  β  = 0.32, 89%HDI = [-0.76, 1.62] 
 posterior probability  p ( β <0) = 0.35 (evidence ratio = 0.54),  p ( β =0) = 0.81 (evidence ratio = 4.2) 
 ROPE = [-0.18, 0.18], % HDI inside ROPE = 21.5%;  p  ROPE  = 0.191 
 comparison ‘null’ vs ‘MCPH1’: [B> L= W<(33%:67%) K>]: moderate evidence for null against MCPH1 (BF=3.79), LOO=-0.58 [SE=0.74], WAIC=-0.69 [SE=0.36], KFOLD=2.03 [SE=1.93] 
 comparison ‘null’ vs ‘MCPH1’: [B> L= W<(33%:67%) K>]: moderate evidence for null against MCPH1 (BF=3.79), LOO=-0.58 [SE=0.74], WAIC=-0.69 [SE=0.36], KFOLD=2.03 [SE=1.93] 
  
  both alleles :
 
 comparison ‘null’ vs ‘both’: [B>> L> W=(37%:63%) K=]: strong evidence for null against both (BF=10.8), LOO=1.02 [SE=0.89], WAIC=-0.53 [SE=0.56], KFOLD=-0.24 [SE=3.20] 
 interaction:
 
 posterior probability  p (=0) = 0.77 (evidence ratio = 3.3) 
 ROPE = [-0.18, 0.18], % HDI inside ROPE = 16%;  p  ROPE  = 0.142 
 comparison ‘no interaction’ vs ‘with interaction’: [B> L< W<(27%:73%) K>>]: moderate evidence for no interaction against with interaction (BF=4.09), LOO=-1.73 [SE=0.94], WAIC=-0.98 [SE=0.65], KFOLD=9.48 [SE=3.05] 
  
  ASPM  (partial):
 
  β  = -0.16, 89%HDI = [-1.54, 1.43] 
 posterior probability  p ( β <0) = 0.58 (evidence ratio = 1.4),  p ( β =0) = 0.79 (evidence ratio = 3.7) 
 ROPE = [-0.18, 0.18], % HDI inside ROPE = 18.9%;  p  ROPE  = 0.168 
  
  MCPH1  (partial):
 
  β  = 0.37, 89%HDI = [-0.90, 1.61] 
 posterior probability  p ( β <0) = 0.33 (evidence ratio = 0.48),  p ( β =0) = 0.8 (evidence ratio = 4.1) 
 ROPE = [-0.18, 0.18], % HDI inside ROPE = 20.7%;  p  ROPE  = 0.184 
  
  
 
 
  
 
  Figure 239.    Posterior distributions (with 50% probability mass highlighted) versus 0.0 (the vertical line) for  ASPM -D (left) and  MCPH1 -D (right). 
 
 
 
  
 
  Figure 240.    Conditional effects of  ASPM -D (left) and  MCPH1 -D (right). 
 
 
 
  
 
  Figure 241.    Posterior predictive checks for  ASPM -D (left) and  MCPH1 -D (right). 
 
 
 
  
 
  Figure 242.    Confusion matrices for  ASPM -D (left) and  MCPH1 -D (right). 
 
 
 
 
 
 
  tone2  
 The resulting dataset has 143 observations, distributed among 87 unique Glottolg codes in 28 families (ranging from a minimum of 1 language per family to a maximum of 47, with a mean 5.1 and median 2 languages per family) and 3 macroareas. 
 There are 131:99:87 unique samples:(meta)populations:languages retained. 
 
 
 
 
 
 
 
 
 
 
   
 America 
 Eurasia 
 Papunesia 
 Sum 
 
 
 
 
  No  
 9 
 105 
 9 
 123 
 
 
  Yes  
 1 
 18 
 1 
 20 
 
 
  Sum  
 10 
 123 
 10 
 143 
 
 
 
 
 
 
  Figure 243.    Distribution of  tone2 . 
 
 
 
 
 
  Figure 244.    Map of  tone2 . 
 
 
 
 
 
  Figure 245.    Relationship between  tone2 ,  ASPM -D and  MCPH1 -D. 
 
 
 
 Regressions 
 
  glmer  
 
 All data 
 
  null model : R 2  = 0.0%, ICC = 98.3% 
  macroarea :  p  macroarea/null  = 0.97 
  ASPM :
 
 by itself: R 2  = 0.1%,  β  = 0.35 ± 1.11,  p  ASPM/null  = 0.75 
 quadratic: R 2  = 37.2%,  β ASPM2   = -8.33 ± 6.96,  p  ASPM2/ASPM  = 0.028 
 with  macroarea : R 2  = 0.1%,  p  macroarea/ASPM  = 0.93,  p  ASPM/macroarea  = 0.67 
  
  MCPH1 :
 
 by itself: R 2  = 0.0%,  β  = 0.20 ± 0.96,  p  MCPH1/null  = 0.83 
 quadratic: R 2  = 1.0%,  β MCPH12   = 0.07 ± 1.26,  p  MCPH12/MCPH1  = 0.15 
 with  macroarea : R 2  = 0.1%,  p  macroarea/MCPH1  = 0.98,  p  MCPH1/macroarea  = 0.87 
  
  both alleles  (no  macroarea ):
 
  ASPM  +  MCPH1 : R 2  = 0.1%,  β  ASPM  = 0.32 ± 1.12,  p  ASPM/MCPH1  = 0.77,  β  MCPH1  = 0.17 ± 1.01,  p  MCPH1/ASPM  = 0.86,  p  ASPM+MCPH1/null  = 0.94, 
 interaction: R 2  = 0.2%,  p  ASPM:MCPH1/ASPM+MCPH1  = 0.53 
  
 
 
 
 Randomization 
 I performed 1000 independent replications of each of these parameter combinations, and below are the distributions of the permuted values versus the original ones (i.e., those obtained on the original, non-permuted data). 
 
 Regressions on 1000 permuted data. The first 3 columns show the permutation constraints (if any), how the  macroarea  is considered (if at all), and what is permuted. The next columns show the percent of the permutations that, in order, have a better AIC compared to the original fit, are significantly better than the null model (thus testing the effect of both alleles simultaneously), have a significant effect of  ASPM -D, have a smaller effect ( β ) of  ASPM -D than the original fit, and the same for  MCPH1 -D. 
 
 
 Permute within 
 Macroarea 
 Permute 
 AIC 
 Signif. 
  p   ASPM -D  
  β   ASPM -D  
  p   MCPH1 -D  
  β   MCPH1 -D  
 
 
 
 
 unrestricted 
 none 
 tone 
 0% 
 6% 
 6% 
 87% 
 6% 
 72% 
 
 
 unrestricted 
 none 
 alleles-together 
 94% 
 8% 
 8% 
 65% 
 8% 
 60% 
 
 
 unrestricted 
 none 
 alleles-independent 
 95% 
 8% 
 8% 
 65% 
 7% 
 58% 
 
 
 unrestricted 
 fixef 
 tone 
 0% 
 6% 
 5% 
 96% 
 6% 
 56% 
 
 
 unrestricted 
 fixef 
 alleles-together 
 94% 
 8% 
 8% 
 74% 
 8% 
 54% 
 
 
 unrestricted 
 fixef 
 alleles-independent 
 94% 
 10% 
 8% 
 74% 
 9% 
 54% 
 
 
 macroareas 
 none 
 tone 
 0% 
 3% 
 4% 
 87% 
 5% 
 74% 
 
 
 macroareas 
 none 
 alleles-together 
 95% 
 9% 
 8% 
 70% 
 8% 
 54% 
 
 
 macroareas 
 none 
 alleles-independent 
 95% 
 10% 
 10% 
 68% 
 9% 
 58% 
 
 
 macroareas 
 fixef 
 tone 
 0% 
 6% 
 6% 
 95% 
 6% 
 53% 
 
 
 macroareas 
 fixef 
 alleles-together 
 93% 
 12% 
 10% 
 74% 
 11% 
 52% 
 
 
 macroareas 
 fixef 
 alleles-independent 
 92% 
 12% 
 10% 
 74% 
 10% 
 51% 
 
 
 families 
 none 
 tone 
 63% 
 8% 
 7% 
 77% 
 4% 
 45% 
 
 
 families 
 none 
 alleles-together 
 90% 
 6% 
 5% 
 75% 
 3% 
 45% 
 
 
 families 
 none 
 alleles-independent 
 89% 
 7% 
 7% 
 74% 
 6% 
 54% 
 
 
 families 
 fixef 
 tone 
 63% 
 8% 
 8% 
 82% 
 4% 
 34% 
 
 
 families 
 fixef 
 alleles-together 
 86% 
 6% 
 5% 
 76% 
 4% 
 40% 
 
 
 families 
 fixef 
 alleles-independent 
 89% 
 7% 
 9% 
 78% 
 7% 
 48% 
 
 
 
 
 
 
Regressions on 1000 permuted data. Each plot shows the original result (vertical dashed black line) and the distribution of the permutations for the three possible things to be permuted (colored curves) for each combination of permutation constraints (horizontal panels) and control for  macroarea  (vertical panels) in terms of the effect size  β ;  ASPM -D is on the left and  MCPH1 -D on the right. The vertical dotted black thin line is at 0.0.
 
 
 
 
 Restricted sampling 
 
 
 
  Figure 246.    Results for 1000 restricted samplings. For  ASPM -D (left): 100% of βs are negative when regressing tone on  ASPM  alone (one-sided  t -test < 0:  t (999) = -98.2, mean = -0.74,  p  = 0), 100%, when controlling for the macroarea ( t (999) = -77.7, mean = -1.35,  p  = 0), and 100% when controlling for both macroarea and  MCPH1  ( t (999) = -68.8, mean = -1.58,  p  = 0). For  MCPH1 -D (right): 62.1% of βs are negative when regressing tone on  MCPH1  alone (one-sided  t -test < 0:  t (999) = -9.5, mean = -0.10,  p  = 5.2e-21), 65.7% when controlling for the macroarea ( t (999) = -12.0, mean = -0.21,  p  = 1.5e-31), and 68.7% when controlling for both macroarea and  ASPM  ( t (999) = -14.4, mean = -0.42,  p  = 5.7e-43). 
 
 
 
 
 
  brms  
 
  ASPM  only:
 
  β  = -0.68, 89%HDI = [-2.49, 1.31] 
 posterior probability  p ( β <0) = 0.72 (evidence ratio = 2.6),  p ( β =0) = 0.72 (evidence ratio = 2.6) 
 ROPE = [-0.18, 0.18], % HDI inside ROPE = 11.5%;  p  ROPE  = 0.102 
 comparison ‘null’ vs ‘ASPM’: [B> L> W>(65%:35%) K>]: moderate evidence for null against ASPM (BF=3.61), LOO=1.75 [SE=1.00], WAIC=0.60 [SE=0.48], KFOLD=0.72 [SE=0.65] 
 comparison ‘null’ vs ‘ASPM’: [B> L> W>(65%:35%) K>]: moderate evidence for null against ASPM (BF=3.61), LOO=1.75 [SE=1.00], WAIC=0.60 [SE=0.48], KFOLD=0.72 [SE=0.65] 
  
  MCPH1  only:
 
  β  = 0.13, 89%HDI = [-1.41, 1.84] 
 posterior probability  p ( β <0) = 0.46 (evidence ratio = 0.84),  p ( β =0) = 0.78 (evidence ratio = 3.6) 
 ROPE = [-0.18, 0.18], % HDI inside ROPE = 17.9%;  p  ROPE  = 0.16 
 comparison ‘null’ vs ‘MCPH1’: [B> L= W=(51%:49%) K<]: moderate evidence for null against MCPH1 (BF=3.36), LOO=-0.12 [SE=0.43], WAIC=0.03 [SE=0.31], KFOLD=-0.96 [SE=0.51] 
 comparison ‘null’ vs ‘MCPH1’: [B> L= W=(51%:49%) K<]: moderate evidence for null against MCPH1 (BF=3.36), LOO=-0.12 [SE=0.43], WAIC=0.03 [SE=0.31], KFOLD=-0.96 [SE=0.51] 
  
  both alleles :
 
 comparison ‘null’ vs ‘both’: [B> L= W=(52%:48%) K>>]: moderate evidence for null against both (BF=7.53), LOO=0.20 [SE=0.62], WAIC=0.06 [SE=0.56], KFOLD=8.34 [SE=4.00] 
 interaction:
 
 posterior probability  p (=0) = 0.7 (evidence ratio = 2.4) 
 ROPE = [-0.18, 0.18], % HDI inside ROPE = 10.5%;  p  ROPE  = 0.094 
 comparison ‘no interaction’ vs ‘with interaction’: [B= L< W<(33%:67%) K<]: anecdotal evidence for no interaction against with interaction (BF=2.84), LOO=-0.76 [SE=0.51], WAIC=-0.69 [SE=0.35], KFOLD=-5.06 [SE=4.57] 
  
  ASPM  (partial):
 
  β  = -0.68, 89%HDI = [-2.85, 1.40] 
 posterior probability  p ( β <0) = 0.71 (evidence ratio = 2.4),  p ( β =0) = 0.71 (evidence ratio = 2.4) 
 ROPE = [-0.18, 0.18], % HDI inside ROPE = 11.5%;  p  ROPE  = 0.102 
  
  MCPH1  (partial):
 
  β  = 0.15, 89%HDI = [-1.44, 1.90] 
 posterior probability  p ( β <0) = 0.46 (evidence ratio = 0.84),  p ( β =0) = 0.78 (evidence ratio = 3.5) 
 ROPE = [-0.18, 0.18], % HDI inside ROPE = 17%;  p  ROPE  = 0.152 
  
  
 
 
  
 
  Figure 247.    Posterior distributions (with 50% probability mass highlighted) versus 0.0 (the vertical line) for  ASPM -D (left) and  MCPH1 -D (right). 
 
 
 
  
 
  Figure 248.    Conditional effects of  ASPM -D (left) and  MCPH1 -D (right). 
 
 
 
  
 
  Figure 249.    Posterior predictive checks for  ASPM -D (left) and  MCPH1 -D (right). 
 
 
 
  
 
  Figure 250.    Confusion matrices for  ASPM -D (left) and  MCPH1 -D (right). 
 
 
 
 
 
 
 Tone  counts  
 The resulting dataset has 146 observations, distributed among 89 unique Glottolg codes in 28 families (ranging from a minimum of 1 language per family to a maximum of 47, with a mean 5.2 and median 2 languages per family) and 3 macroareas. 
 There are 131:99:89 unique samples:(meta)populations:languages retained. 
 
 
 
 
 
 
 
 
 
 
   
 America 
 Eurasia 
 Papunesia 
 Sum 
 
 
 
 
  0  
 4 
 98 
 7 
 109 
 
 
  1  
 5 
 6 
 1 
 12 
 
 
  2  
 0 
 3 
 2 
 5 
 
 
  3  
 0 
 5 
 0 
 5 
 
 
  4  
 1 
 8 
 0 
 9 
 
 
  5  
 0 
 4 
 0 
 4 
 
 
  6  
 0 
 2 
 0 
 2 
 
 
  Sum  
 10 
 126 
 10 
 146 
 
 
 
 
 
 
  Figure 251.    Distribution of tone  counts . 
 
 
 
 
 
  Figure 252.    Distribution of tone  counts  across the world. 
 
 
 
 
 
  Figure 253.    Relationship between tone  counts  (colors) and the two alleles (frequency) by macroarea. 
 
 
 
 Regressions 
 
  glmer  
 
 All data 
 
  null model : R 2  = 0.0%, ICC = 100.0% 
 the Poisson model is  not  overdispersed:  χ  2 (144) = 78.8,  p  = 1 
  macroarea :  p  macroarea/null  = 0.6 
  ASPM :
 
 by itself: R 2  = 0.0%,  β  = 0.00 ± 0.25,  p  ASPM/null  = 1 
 quadratic: R 2  = 8.5%,  β ASPM2   = -0.33 ± 0.33,  p  ASPM2/ASPM  = 0.073 
 with  macroarea : R 2  = 2.3%,  p  macroarea/ASPM  = 0.58,  p  ASPM/macroarea  = 0.79 
  
  MCPH1 :
 
 by itself: R 2  = 0.2%,  β  = -0.08 ± 0.17,  p  MCPH1/null  = 0.63 
 quadratic: R 2  = 2.0%,  β MCPH12   = -0.04 ± 0.18,  p  MCPH12/MCPH1  = 0.16 
 with  macroarea : R 2  = 2.7%,  p  macroarea/MCPH1  = 0.53,  p  MCPH1/macroarea  = 0.5 
  
  both alleles  (no  macroarea ):
 
  ASPM  +  MCPH1 : R 2  = 0.2%,  β  ASPM  = -0.01 ± 0.25,  p  ASPM/MCPH1  = 0.96,  β  MCPH1  = -0.08 ± 0.17,  p  MCPH1/ASPM  = 0.63,  p  ASPM+MCPH1/null  = 0.89, 
 interaction: R 2  = 0.3%,  p  ASPM:MCPH1/ASPM+MCPH1  = 0.76 
  
 
 
 
 Randomization 
 We performed 1000 independent replications: 
 
 Regressions with randomizations for tone  counts . 
 
 
 Permute within 
 Macroarea 
 Permute 
 AIC 
 Signif. 
  p   ASPM -D  
  β   ASPM -D  
  p   MCPH1 -D  
  β   MCPH1 -D  
 
 
 
 
 unrestricted 
 none 
 tone 
 0% 
 35% 
 24% 
 40% 
 25% 
 35% 
 
 
 unrestricted 
 none 
 alleles-together 
 89% 
 3% 
 3% 
 43% 
 4% 
 24% 
 
 
 unrestricted 
 none 
 alleles-independent 
 88% 
 3% 
 4% 
 44% 
 4% 
 22% 
 
 
 unrestricted 
 fixef 
 tone 
 0% 
 36% 
 25% 
 56% 
 24% 
 28% 
 
 
 unrestricted 
 fixef 
 alleles-together 
 78% 
 4% 
 4% 
 68% 
 3% 
 15% 
 
 
 unrestricted 
 fixef 
 alleles-independent 
 74% 
 2% 
 3% 
 71% 
 3% 
 12% 
 
 
 macroareas 
 none 
 tone 
 0% 
 33% 
 20% 
 50% 
 24% 
 28% 
 
 
 macroareas 
 none 
 alleles-together 
 88% 
 3% 
 3% 
 54% 
 4% 
 17% 
 
 
 macroareas 
 none 
 alleles-independent 
 88% 
 3% 
 4% 
 55% 
 4% 
 17% 
 
 
 macroareas 
 fixef 
 tone 
 0% 
 35% 
 24% 
 54% 
 25% 
 28% 
 
 
 macroareas 
 fixef 
 alleles-together 
 77% 
 3% 
 4% 
 69% 
 3% 
 15% 
 
 
 macroareas 
 fixef 
 alleles-independent 
 76% 
 3% 
 4% 
 68% 
 4% 
 18% 
 
 
 families 
 none 
 tone 
 69% 
 5% 
 11% 
 79% 
 1% 
 33% 
 
 
 families 
 none 
 alleles-together 
 90% 
 6% 
 10% 
 80% 
 0% 
 23% 
 
 
 families 
 none 
 alleles-independent 
 88% 
 6% 
 13% 
 80% 
 1% 
 23% 
 
 
 families 
 fixef 
 tone 
 67% 
 5% 
 10% 
 84% 
 1% 
 23% 
 
 
 families 
 fixef 
 alleles-together 
 77% 
 7% 
 10% 
 86% 
 1% 
 22% 
 
 
 families 
 fixef 
 alleles-independent 
 77% 
 6% 
 11% 
 86% 
 1% 
 19% 
 
 
 
 
 
 
Regressions on 1000 permuted data. Each plot shows the original result (vertical dashed black line) and the distribution of the permutations for the three possible things to be permuted (colored curves) for each combination of permutation constraints (horizontal panels) and control for  macroarea  (vertical panels) in terms of the effect size  β ;  ASPM -D is on the left and  MCPH1 -D on the right. The vertical dotted black thin line is at 0.0.
 
 
 
 
 Restricted sampling 
 
 
 
  Figure 254.    Results for 1000 restricted samplings. For  ASPM -D (left): 99.9% of βs are negative when regressing tone on  ASPM  alone (one-sided  t -test < 0:  t (999) = -74.5, mean = -0.43,  p  = 0), 98.2%, when controlling for the macroarea ( t (999) = -59.5, mean = -0.60,  p  = 0), and 98.4% when controlling for both macroarea and  MCPH1  ( t (999) = -61.1, mean = -0.67,  p  = 0). For  MCPH1 -D (right): 35.2% of βs are negative when regressing tone on  MCPH1  alone (one-sided  t -test < 0:  t (999) = 15.6, mean = 0.13,  p  = 1), 43.2% when controlling for the macroarea ( t (999) = 8.4, mean = 0.11,  p  = 1), and 45.2% when controlling for both macroarea and  ASPM  ( t (999) = 8.2, mean = 0.15,  p  = 1). 
 
 
 
 
 
  brms  
 
  ASPM  only:
 
  β  = -0.12, 89%HDI = [-0.64, 0.40] 
 posterior probability  p ( β <0) = 0.64 (evidence ratio = 1.8),  p ( β =0) = 0.91 (evidence ratio = 9.9) 
 ROPE = [-0.10, 0.10], % HDI inside ROPE = 26.2%;  p  ROPE  = 0.233 
 comparison ‘null’ vs ‘ASPM’: [B>> L>> W>>(67%:33%) K=]: strong evidence for null against ASPM (BF=11.8), LOO=1.40 [SE=0.57], WAIC=0.71 [SE=0.33], KFOLD=-0.27 [SE=2.39] 
  
  MCPH1  only:
 
  β  = 0.01, 89%HDI = [-0.33, 0.34] 
 posterior probability  p ( β <0) = 0.48 (evidence ratio = 0.92),  p ( β =0) = 0.94 (evidence ratio = 16) 
 ROPE = [-0.10, 0.10], % HDI inside ROPE = 42.3%;  p  ROPE  = 0.376 
 comparison ‘null’ vs ‘MCPH1’: [B>> L= W=(57%:43%) K=]: strong evidence for null against MCPH1 (BF=12.8), LOO=0.01 [SE=0.58], WAIC=0.28 [SE=0.29], KFOLD=-1.27 [SE=2.61] 
  
  both alleles :
 
 comparison ‘null’ vs ‘both’: [B>> L>> W>>(77%:23%) K=]: very strong evidence for null against both (BF=80.5), LOO=1.83 [SE=0.81], WAIC=1.20 [SE=0.49], KFOLD=1.19 [SE=2.51] 
 interaction:
 
 posterior probability  p (=0) = 0.92 (evidence ratio = 11) 
 ROPE = [-0.10, 0.10], % HDI inside ROPE = 28.7%;  p  ROPE  = 0.255 
 comparison ‘no interaction’ vs ‘with interaction’: [B> L= W=(45%:55%) K=]: moderate evidence for no interaction against with interaction (BF=8.41), LOO=-0.69 [SE=0.85], WAIC=-0.21 [SE=0.30], KFOLD=-0.59 [SE=1.39] 
  
  ASPM  (partial):
 
  β  = -0.13, 89%HDI = [-0.67, 0.40] 
 posterior probability  p ( β <0) = 0.66 (evidence ratio = 1.9),  p ( β =0) = 0.91 (evidence ratio = 9.9) 
 ROPE = [-0.10, 0.10], % HDI inside ROPE = 25.9%;  p  ROPE  = 0.231 
  
  MCPH1  (partial):
 
  β  = 0, 89%HDI = [-0.34, 0.34] 
 posterior probability  p ( β <0) = 0.5 (evidence ratio = 0.98),  p ( β =0) = 0.94 (evidence ratio = 15) 
 ROPE = [-0.10, 0.10], % HDI inside ROPE = 40%;  p  ROPE  = 0.356 
  
  
 
 
  
 
  Figure 255.    Posterior distributions (with 50% probability mass highlighted) versus 0.0 (the vertical line) for  ASPM -D (left) and  MCPH1 -D (right). 
 
 
 
  
 
  Figure 256.    Conditional effects of  ASPM -D (left) and  MCPH1 -D (right). 
 
 
 
  
 
  Figure 257.    Posterior predictive checks for  ASPM -D (left) and  MCPH1 -D (right). 
 
 
 
 
 
 
 
 Only Africa 
 Here I use only the African data points. 
 
  tone1  
 There are 36 observations, distributed among 30 unique Glottolg codes in 8 families (ranging from a minimum of 1 language per family to a maximum of 16, with a mean 4.5 and median 1.5 languages per family) and 1 macroareas. 
 There are 27:24:30 unique samples:(meta)populations:languages retained. 
 
 
 
 
 
 
 
 
 
 
 
   
 Africa 
 Eurasia 
 America 
 Papunesia 
 Sum 
 
 
 
 
  No  
 9 
 0 
 0 
 0 
 9 
 
 
  Yes  
 27 
 0 
 0 
 0 
 27 
 
 
  Sum  
 36 
 0 
 0 
 0 
 36 
 
 
 
 
 
 
  Figure 258.    Distribution of  tone1 . 
 
 
 
 
 
  Figure 259.    Map of  tone1 . 
 
 
 
 
 
  Figure 260.    Relationship between  tone1 ,  ASPM -D and  MCPH1 -D. 
 
 
 
 Regressions 
 
  glmer  
 
 All data 
 trying to fit a random effects structure with language family as the random effects results in convergence problems ( boundary (singular) fit: see ?isSingular ) and the random effects seems to not matter at all ( Can't compute random effect variances. Some variance components equal zero. Your model may suffer from singulariy. Solution: Respecify random structure! ), so that I reverted to a “flat” model without random effects (using  glm()  instead of  glmer() ). As expected, the  anova()  comparisons produce the same  p -values for this  glm  “flat” approach as for the  glmer  with family as random effects, but without the convergence issues… 
 
  null model : R 2  = 0.0% 
  ASPM :
 
 by itself: R 2  = 3.6%,  β  = -0.38 ± 0.36,  p  ASPM/null  = 0.29 
 quadratic: R 2  = 9.2%,  β ASPM2   = 0.74 ± 0.94,  p  ASPM2/ASPM  = 0.18 
  
  MCPH1 :
 
 by itself: R 2  = 2.9%,  β  = -0.36 ± 0.38,  p  MCPH1/null  = 0.34 
 quadratic: R 2  = 7.7%,  β MCPH12   = 0.15 ± 0.57,  p  MCPH12/MCPH1  = 0.23 
  
  both alleles  (no  macroarea ):
 
  ASPM  +  MCPH1 : R 2  = 3.9%,  β  ASPM  = -0.28 ± 0.53,  p  ASPM/MCPH1  = 0.6,  β  MCPH1  = -0.14 ± 0.55,  p  MCPH1/ASPM  = 0.8,  p  ASPM+MCPH1/null  = 0.56, 
 interaction: R 2  = 12.1%,  p  ASPM:MCPH1/ASPM+MCPH1  = 0.085 
  
 
 
 
 Randomization 
 1000 independent replications. 
 
 Regressions on 1000 permuted data. The first 3 columns show the permutation constraints (if any), how the  macroarea  is considered (if at all), and what is permuted. The next columns show the percent of the permutations that, in order, have a better AIC compared to the original fit, are significantly better than the null model (thus testing the effect of both alleles simultaneously), have a significant effect of  ASPM -D, have a smaller effect ( β ) of  ASPM -D than the original fit, and the same for  MCPH1 -D. 
 
 
 Permute within 
 Macroarea 
 Permute 
 AIC 
 Signif. 
  p   ASPM -D  
  β   ASPM -D  
  p   MCPH1 -D  
  β   MCPH1 -D  
 
 
 
 
 unrestricted 
 none 
 tone 
 64% 
 6% 
 7% 
 31% 
 8% 
 38% 
 
 
 unrestricted 
 none 
 alleles-together 
 61% 
 7% 
 8% 
 28% 
 7% 
 40% 
 
 
 unrestricted 
 none 
 alleles-independent 
 61% 
 8% 
 8% 
 22% 
 6% 
 32% 
 
 
 families 
 none 
 tone 
 55% 
 5% 
 3% 
 10% 
 5% 
 60% 
 
 
 families 
 none 
 alleles-together 
 51% 
 5% 
 4% 
 10% 
 4% 
 59% 
 
 
 families 
 none 
 alleles-independent 
 55% 
 5% 
 5% 
 19% 
 6% 
 51% 
 
 
 
 
 
 
Regressions on 1000 permuted data. Each plot shows the original result (vertical dashed black line) and the distribution of the permutations for the three possible things to be permuted (colored curves) for each combination of permutation constraints (horizontal panels) and control for  macroarea  (vertical panels) in terms of the effect size  β ;  ASPM -D is on the left and  MCPH1 -D on the right. The vertical dotted black thin line is at 0.0.
 
 
 
 
 Restricted sampling 
 
 
 
  Figure 261.    Results for 1000 restricted samplings. For  ASPM -D (left): 33.7% of βs are negative when regressing tone on  ASPM  alone (one-sided  t -test < 0:  t (999) = 23.4, mean = 24.18,  p  = 1), and 34.1% when controlling for  MCPH1  ( t (999) = 5.6, mean = 58.21,  p  = 1). For  MCPH1 -D (right): 34.3% of βs are negative when regressing tone on  MCPH1  alone (one-sided  t -test < 0:  t (999) = 10.0, mean = 11.26,  p  = 1), and 43.6% when controlling for  ASPM  ( t (999) = -2.8, mean = -8.39,  p  = 0.003). 
 
 
 
 
 
  brms  
 
  ASPM  only:
 
  β  = -0.64, 89%HDI = [-1.50, 0.35] 
 posterior probability  p ( β <0) = 0.88 (evidence ratio = 7.1),  p ( β =0) = 0.78 (evidence ratio = 3.6) 
 ROPE = [-0.18, 0.18], % HDI inside ROPE = 17.5%;  p  ROPE  = 0.156 
 comparison ‘null’ vs ‘ASPM’: [B> L= W=(57%:43%) K=]: moderate evidence for null against ASPM (BF=3.4), LOO=0.43 [SE=1.11], WAIC=0.27 [SE=1.10], KFOLD=0.58 [SE=1.46] 
 comparison ‘null’ vs ‘ASPM’: [B> L= W=(57%:43%) K=]: moderate evidence for null against ASPM (BF=3.4), LOO=0.43 [SE=1.11], WAIC=0.27 [SE=1.10], KFOLD=0.58 [SE=1.46] 
  
  MCPH1  only:
 
  β  = -0.49, 89%HDI = [-1.30, 0.34] 
 posterior probability  p ( β <0) = 0.83 (evidence ratio = 5.1),  p ( β =0) = 0.82 (evidence ratio = 4.5) 
 ROPE = [-0.18, 0.18], % HDI inside ROPE = 21.7%;  p  ROPE  = 0.194 
 comparison ‘null’ vs ‘MCPH1’: [B> L= W=(68%:32%) K>]: moderate evidence for null against MCPH1 (BF=4.01), LOO=0.81 [SE=0.95], WAIC=0.75 [SE=0.95], KFOLD=1.98 [SE=1.30] 
 comparison ‘null’ vs ‘MCPH1’: [B> L= W=(68%:32%) K>]: moderate evidence for null against MCPH1 (BF=4.01), LOO=0.81 [SE=0.95], WAIC=0.75 [SE=0.95], KFOLD=1.98 [SE=1.30] 
  
  both alleles :
 
 comparison ‘null’ vs ‘both’: [B>> L> W>(78%:22%) K>]: strong evidence for null against both (BF=14.4), LOO=1.65 [SE=1.24], WAIC=1.25 [SE=1.23], KFOLD=2.22 [SE=1.46] 
 interaction:
 
 posterior probability  p (=0) = 0.55 (evidence ratio = 1.2) 
 ROPE = [-0.18, 0.18], % HDI inside ROPE = 2%;  p  ROPE  = 0.049 
 comparison ‘no interaction’ vs ‘with interaction’: [B= L= W=(37%:63%) K=]: anecdotal evidence for no interaction against with interaction (BF=1.01), LOO=-0.18 [SE=1.85], WAIC=-0.51 [SE=1.70], KFOLD=-0.79 [SE=2.41] 
  
  ASPM  (partial):
 
  β  = -0.62, 89%HDI = [-1.95, 0.72] 
 posterior probability  p ( β <0) = 0.77 (evidence ratio = 3.3),  p ( β =0) = 0.78 (evidence ratio = 3.6) 
 ROPE = [-0.18, 0.18], % HDI inside ROPE = 17.7%;  p  ROPE  = 0.158 
  
  MCPH1  (partial):
 
  β  = -0.11, 89%HDI = [-1.39, 1.03] 
 posterior probability  p ( β <0) = 0.56 (evidence ratio = 1.3),  p ( β =0) = 0.81 (evidence ratio = 4.3) 
 ROPE = [-0.18, 0.18], % HDI inside ROPE = 21%;  p  ROPE  = 0.187 
  
  
 
 
  
 
  Figure 262.    Posterior distributions (with 50% probability mass highlighted) versus 0.0 (the vertical line) for  ASPM -D (left) and  MCPH1 -D (right). 
 
 
 
  
 
  Figure 263.    Conditional effects of  ASPM -D (left) and  MCPH1 -D (right). 
 
 
 
  
 
  Figure 264.    Posterior predictive checks for  ASPM -D (left) and  MCPH1 -D (right). 
 
 
 
  
 
  Figure 265.    Confusion matrices for  ASPM -D (left) and  MCPH1 -D (right). 
 
 
 
 
 
 
  tone2  
 The resulting dataset has 37 observations, distributed among 31 unique Glottolg codes in 8 families (ranging from a minimum of 1 language per family to a maximum of 18, with a mean 4.6 and median 2 languages per family) and 1 macroareas. 
 There are 25:22:31 unique samples:(meta)populations:languages retained. 
 
 
 
 
 
 
 
 
 
 
 
   
 Africa 
 Eurasia 
 America 
 Papunesia 
 Sum 
 
 
 
 
  No  
 28 
 0 
 0 
 0 
 28 
 
 
  Yes  
 9 
 0 
 0 
 0 
 9 
 
 
  Sum  
 37 
 0 
 0 
 0 
 37 
 
 
 
 
 
 
  Figure 266.    Distribution of  tone2 . 
 
 
 
 
 
  Figure 267.    Map of  tone2 . 
 
 
 
 
 
  Figure 268.    Relationship between  tone2 ,  ASPM -D and  MCPH1 -D. 
 
 
 
 Regressions 
 
  glmer  
 
 All data 
 
  null model : R 2  = 0.0%, ICC = 3.1% 
  ASPM :
 
 by itself: R 2  = 1.6%,  β  = -0.23 ± 0.47,  p  ASPM/null  = 0.6 
 quadratic: R 2  = 5.8%,  β ASPM2   = 0.30 ± 0.94,  p  ASPM2/ASPM  = 0.5 
  
  MCPH1 :
 
 by itself: R 2  = 0.1%,  β  = -0.06 ± 0.43,  p  MCPH1/null  = 0.89 
 quadratic: R 2  = 5.3%,  β MCPH12   = 0.19 ± 0.56,  p  MCPH12/MCPH1  = 0.39 
  
  both alleles  (no  macroarea ):
 
  ASPM  +  MCPH1 : R 2  = 1.9%,  β  ASPM  = -0.31 ± 0.56,  p  ASPM/MCPH1  = 0.58,  β  MCPH1  = 0.14 ± 0.57,  p  MCPH1/ASPM  = 0.81,  p  ASPM+MCPH1/null  = 0.85, 
 interaction: R 2  = 1.9%,  p  ASPM:MCPH1/ASPM+MCPH1  = 0.98 
  
 
 
 
 Randomization 
 
 Regressions with randomizations for  tone2 . 
 
 
 Permute within 
 Macroarea 
 Permute 
 AIC 
 Signif. 
  p   ASPM -D  
  β   ASPM -D  
  p   MCPH1 -D  
  β   MCPH1 -D  
 
 
 
 
 unrestricted 
 none 
 tone 
 86% 
 7% 
 8% 
 34% 
 5% 
 61% 
 
 
 unrestricted 
 none 
 alleles-together 
 88% 
 9% 
 9% 
 40% 
 6% 
 60% 
 
 
 unrestricted 
 none 
 alleles-independent 
 87% 
 8% 
 7% 
 32% 
 7% 
 60% 
 
 
 families 
 none 
 tone 
 90% 
 7% 
 10% 
 55% 
 6% 
 39% 
 
 
 families 
 none 
 alleles-together 
 92% 
 7% 
 10% 
 54% 
 5% 
 43% 
 
 
 families 
 none 
 alleles-independent 
 87% 
 9% 
 11% 
 43% 
 6% 
 50% 
 
 
 
 
 
 
Regressions on 1000 permuted data. Each plot shows the original result (vertical dashed black line) and the distribution of the permutations for the three possible things to be permuted (colored curves) for each combination of permutation constraints (horizontal panels) and control for  macroarea  (vertical panels) in terms of the effect size  β ;  ASPM -D is on the left and  MCPH1 -D on the right. The vertical dotted black thin line is at 0.0.
 
 
 
 
 Restricted sampling 
 
 
 
  Figure 269.    Results for 1000 restricted samplings. For  ASPM -D (left): 29.6% of βs are negative when regressing tone on  ASPM  alone (one-sided  t -test < 0:  t (999) = 7.8, mean = 1.59,  p  = 1) and 45.1% when controlling for  MCPH1  ( t (999) = 9.3, mean = 2.47,  p  = 1). For  MCPH1 -D (right): 27% of βs are negative when regressing tone on  MCPH1  alone (one-sided  t -test < 0:  t (999) = 9.5, mean = 11.06,  p  = 1) and 32.6% when controlling for  ASPM  ( t (999) = 8.5, mean = 2.40,  p  = 1). 
 
 
 
 
 
  brms  
 
  ASPM  only:
 
  β  = -0.37, 89%HDI = [-1.88, 0.99] 
 posterior probability  p ( β <0) = 0.68 (evidence ratio = 2.1),  p ( β =0) = 0.79 (evidence ratio = 3.7) 
 ROPE = [-0.18, 0.18], % HDI inside ROPE = 18.4%;  p  ROPE  = 0.164 
 comparison ‘null’ vs ‘ASPM’: [B> L> W=(61%:39%) K>>]: moderate evidence for null against ASPM (BF=3.68), LOO=0.85 [SE=0.79], WAIC=0.45 [SE=0.59], KFOLD=2.38 [SE=1.05] 
 comparison ‘null’ vs ‘ASPM’: [B> L> W=(61%:39%) K>>]: moderate evidence for null against ASPM (BF=3.68), LOO=0.85 [SE=0.79], WAIC=0.45 [SE=0.59], KFOLD=2.38 [SE=1.05] 
  
  MCPH1  only:
 
  β  = -0.3, 89%HDI = [-1.61, 0.97] 
 posterior probability  p ( β <0) = 0.65 (evidence ratio = 1.8),  p ( β =0) = 0.81 (evidence ratio = 4.4) 
 ROPE = [-0.18, 0.18], % HDI inside ROPE = 21%;  p  ROPE  = 0.187 
 comparison ‘null’ vs ‘MCPH1’: [B> L>> W=(57%:43%) K=]: moderate evidence for null against MCPH1 (BF=4.69), LOO=1.13 [SE=0.45], WAIC=0.29 [SE=0.38], KFOLD=-0.04 [SE=0.93] 
 comparison ‘null’ vs ‘MCPH1’: [B> L>> W=(57%:43%) K=]: moderate evidence for null against MCPH1 (BF=4.69), LOO=1.13 [SE=0.45], WAIC=0.29 [SE=0.38], KFOLD=-0.04 [SE=0.93] 
  
  both alleles :
 
 comparison ‘null’ vs ‘both’: [B>> L> W=(58%:42%) K>>]: strong evidence for null against both (BF=14), LOO=1.57 [SE=0.80], WAIC=0.34 [SE=0.61], KFOLD=7.10 [SE=2.10] 
 interaction:
 
 posterior probability  p (=0) = 0.79 (evidence ratio = 3.7) 
 ROPE = [-0.18, 0.18], % HDI inside ROPE = 18.1%;  p  ROPE  = 0.161 
 comparison ‘no interaction’ vs ‘with interaction’: [B> L= W=(43%:57%) K<<]: moderate evidence for no interaction against with interaction (BF=3.54), LOO=0.27 [SE=0.81], WAIC=-0.28 [SE=0.34], KFOLD=-4.42 [SE=2.17] 
  
  ASPM  (partial):
 
  β  = -0.2, 89%HDI = [-2.07, 1.53] 
 posterior probability  p ( β <0) = 0.58 (evidence ratio = 1.4),  p ( β =0) = 0.76 (evidence ratio = 3.1) 
 ROPE = [-0.18, 0.18], % HDI inside ROPE = 15.5%;  p  ROPE  = 0.138 
  
  MCPH1  (partial):
 
  β  = -0.24, 89%HDI = [-1.83, 1.37] 
 posterior probability  p ( β <0) = 0.57 (evidence ratio = 1.3),  p ( β =0) = 0.78 (evidence ratio = 3.5) 
 ROPE = [-0.18, 0.18], % HDI inside ROPE = 17.9%;  p  ROPE  = 0.159 
  
  
 
 
  
 
  Figure 270.    Posterior distributions (with 50% probability mass highlighted) versus 0.0 (the vertical line) for  ASPM -D (left) and  MCPH1 -D (right). 
 
 
 
  
 
  Figure 271.    Conditional effects of  ASPM -D (left) and  MCPH1 -D (right). 
 
 
 
  
 
  Figure 272.    Posterior predictive checks for  ASPM -D (left) and  MCPH1 -D (right). 
 
 
 
  
 
  Figure 273.    Confusion matrices for  ASPM -D (left) and  MCPH1 -D (right). 
 
 
 
 
 
 
 Tone  counts  
 The resulting dataset has 38 observations, distributed among 32 unique Glottolg codes in 8 families (ranging from a minimum of 1 language per family to a maximum of 19, with a mean 4.8 and median 2 languages per family) and 1 macroareas. 
 There are 25:22:32 unique samples:(meta)populations:languages retained. 
 
 
 
 
 
 
 
 
 
 
 
   
 Africa 
 Eurasia 
 America 
 Papunesia 
 Sum 
 
 
 
 
  0  
 9 
 0 
 0 
 0 
 9 
 
 
  1  
 10 
 0 
 0 
 0 
 10 
 
 
  2  
 16 
 0 
 0 
 0 
 16 
 
 
  3  
 2 
 0 
 0 
 0 
 2 
 
 
  5  
 1 
 0 
 0 
 0 
 1 
 
 
  Sum  
 38 
 0 
 0 
 0 
 38 
 
 
 
 
 
 
  Figure 274.    Distribution of tone  counts . 
 
 
 
 
 
  Figure 275.    Distribution of tone  counts  across the world. 
 
 
 
 
 
  Figure 276.    Relationship between tone  counts  (colors) and the two alleles (frequency) by macroarea. 
 
 
 
 Regressions 
 
  glmer  
 
 All data 
 trying to fit a random effects structure with language family as the random effects results in convergence problems ( boundary (singular) fit: see ?isSingular ) and the random effects seems to not matter at all ( Can't compute random effect variances. Some variance components equal zero. Your model may suffer from singulariy. Solution: Respecify random structure! ), so that I reverted to a “flat” model without random effects (using  glm()  instead of  glmer() ). As expected, the  anova()  comparisons produce the same  p -values for this  glm  “flat” approach as for the  glmer  with family as random effects, but without the convergence issues… 
 
  null model : 
  ASPM :
 
 by itself: ,  β  = -0.07 ± 0.15,  p  ASPM/null  = 0.64 
 quadratic: ,  β ASPM2   = 0.02 ± 0.32,  p  ASPM2/ASPM  = 0.75 
  
  MCPH1 :
 
 by itself: ,  β  = -0.06 ± 0.14,  p  MCPH1/null  = 0.66 
 quadratic: ,  β MCPH12   = -0.03 ± 0.17,  p  MCPH12/MCPH1  = 0.7 
  
  both alleles  (no  macroarea ):
 
  ASPM  +  MCPH1 : ,  β  ASPM  = -0.05 ± 0.18,  p  ASPM/MCPH1  = 0.79,  β  MCPH1  = -0.04 ± 0.17,  p  MCPH1/ASPM  = 0.82,  p  ASPM+MCPH1/null  = 0.88, 
 interaction: ,  p  ASPM:MCPH1/ASPM+MCPH1  = 0.53 
  
 
 
 
 Randomization 
 We performed 1000 independent replications: 
 
 Regressions with randomizations for tone  counts . 
 
 
 Permute within 
 Macroarea 
 Permute 
 AIC 
 Signif. 
  p   ASPM -D  
  β   ASPM -D  
  p   MCPH1 -D  
  β   MCPH1 -D  
 
 
 
 
 unrestricted 
 none 
 tone 
 87% 
 2% 
 3% 
 41% 
 3% 
 40% 
 
 
 unrestricted 
 none 
 alleles-together 
 87% 
 4% 
 4% 
 42% 
 2% 
 42% 
 
 
 unrestricted 
 none 
 alleles-independent 
 86% 
 3% 
 4% 
 36% 
 2% 
 39% 
 
 
 families 
 none 
 tone 
 96% 
 1% 
 0% 
 33% 
 2% 
 76% 
 
 
 families 
 none 
 alleles-together 
 80% 
 0% 
 0% 
 36% 
 2% 
 74% 
 
 
 families 
 none 
 alleles-independent 
 86% 
 1% 
 0% 
 52% 
 3% 
 74% 
 
 
 
 
 
 
Regressions on 1000 permuted data. Each plot shows the original result (vertical dashed black line) and the distribution of the permutations for the three possible things to be permuted (colored curves) for each combination of permutation constraints (horizontal panels) and control for  macroarea  (vertical panels) in terms of the effect size  β ;  ASPM -D is on the left and  MCPH1 -D on the right. The vertical dotted black thin line is at 0.0.
 
 
 
 
 Restricted sampling 
 
 
 
  Figure 277.    Results for 1000 restricted samplings. For  ASPM -D (left): 39.3% of βs are negative when regressing tone on  ASPM  alone (one-sided  t -test < 0:  t (999) = 9.9, mean = 0.07,  p  = 1) and 29.7% when controlling for  MCPH1  ( t (999) = 15.4, mean = 0.10,  p  = 1). For  MCPH1 -D (right): 47.5% of βs are negative when regressing tone on  MCPH1  alone (one-sided  t -test < 0:  t (999) = 5.2, mean = 0.04,  p  = 1) and 56.2% when controlling for  ASPM  ( t (999) = -3.1, mean = -0.02,  p  = 0.00086). 
 
 
 
 
 
  brms  
 
  ASPM  only:
 
  β  = -0.1, 89%HDI = [-0.38, 0.17] 
 posterior probability  p ( β <0) = 0.7 (evidence ratio = 2.4),  p ( β =0) = 0.94 (evidence ratio = 17) 
 ROPE = [-0.10, 0.10], % HDI inside ROPE = 44.6%;  p  ROPE  = 0.397 
 comparison ‘null’ vs ‘ASPM’: [B>> L> W>(71%:29%) K=]: strong evidence for null against ASPM (BF=16.5), LOO=1.00 [SE=0.58], WAIC=0.89 [SE=0.53], KFOLD=-0.93 [SE=1.69] 
 comparison ‘null’ vs ‘ASPM’: [B>> L> W>(71%:29%) K=]: strong evidence for null against ASPM (BF=16.5), LOO=1.00 [SE=0.58], WAIC=0.89 [SE=0.53], KFOLD=-0.93 [SE=1.69] 
  
  MCPH1  only:
 
  β  = -0.05, 89%HDI = [-0.31, 0.20] 
 posterior probability  p ( β <0) = 0.62 (evidence ratio = 1.6),  p ( β =0) = 0.95 (evidence ratio = 20) 
 ROPE = [-0.10, 0.10], % HDI inside ROPE = 50.4%;  p  ROPE  = 0.448 
 comparison ‘null’ vs ‘MCPH1’: [B>> L>> W>>(73%:27%) K<]: strong evidence for null against MCPH1 (BF=19.3), LOO=0.97 [SE=0.35], WAIC=0.99 [SE=0.31], KFOLD=-1.71 [SE=1.51] 
 comparison ‘null’ vs ‘MCPH1’: [B>> L>> W>>(73%:27%) K<]: strong evidence for null against MCPH1 (BF=19.3), LOO=0.97 [SE=0.35], WAIC=0.99 [SE=0.31], KFOLD=-1.71 [SE=1.51] 
  
  both alleles :
 
 comparison ‘null’ vs ‘both’: [B>> L>> W>>(85%:15%) K>>]: extreme evidence for null against both (BF=280), LOO=1.89 [SE=0.63], WAIC=1.74 [SE=0.59], KFOLD=1.47 [SE=0.69] 
 interaction:
 
 posterior probability  p (=0) = 0.95 (evidence ratio = 20) 
 ROPE = [-0.10, 0.10], % HDI inside ROPE = 49.6%;  p  ROPE  = 0.441 
 comparison ‘no interaction’ vs ‘with interaction’: [B>> L> W>(70%:30%) K=]: strong evidence for no interaction against with interaction (BF=17.6), LOO=1.04 [SE=0.85], WAIC=0.86 [SE=0.74], KFOLD=0.67 [SE=1.07] 
  
  ASPM  (partial):
 
  β  = -0.1, 89%HDI = [-0.42, 0.25] 
 posterior probability  p ( β <0) = 0.68 (evidence ratio = 2.1),  p ( β =0) = 0.93 (evidence ratio = 14) 
 ROPE = [-0.10, 0.10], % HDI inside ROPE = 38.3%;  p  ROPE  = 0.341 
  
  MCPH1  (partial):
 
  β  = 0, 89%HDI = [-0.31, 0.33] 
 posterior probability  p ( β <0) = 0.5 (evidence ratio = 0.99),  p ( β =0) = 0.94 (evidence ratio = 16) 
 ROPE = [-0.10, 0.10], % HDI inside ROPE = 43.6%;  p  ROPE  = 0.388 
  
  
 
 
  
 
  Figure 278.    Posterior distributions (with 50% probability mass highlighted) versus 0.0 (the vertical line) for  ASPM -D (left) and  MCPH1 -D (right). 
 
 
 
  
 
  Figure 279.    Conditional effects of  ASPM -D (left) and  MCPH1 -D (right). 
 
 
 
  
 
  Figure 280.    Posterior predictive checks for  ASPM -D (left) and  MCPH1 -D (right). 
 
 
 
 
 
 
 
 Only Eurasia 
 Here I use only the Eurasian data points. 
 
  tone1  
 There are 126 observations, distributed among 74 unique Glottolg codes in 19 families (ranging from a minimum of 1 language per family to a maximum of 48, with a mean 6.6 and median 3 languages per family) and 1 macroareas. 
 There are 118:89:74 unique samples:(meta)populations:languages retained. 
 
 
 
 
 
 
 
 
 
 
 
   
 Africa 
 Eurasia 
 America 
 Papunesia 
 Sum 
 
 
 
 
  No  
 0 
 100 
 0 
 0 
 100 
 
 
  Yes  
 0 
 26 
 0 
 0 
 26 
 
 
  Sum  
 0 
 126 
 0 
 0 
 126 
 
 
 
 
 
 
  Figure 281.    Distribution of  tone1 . 
 
 
 
 
 
  Figure 282.    Map of  tone1 . 
 
 
 
 
 
  Figure 283.    Relationship between  tone1 ,  ASPM -D and  MCPH1 -D. 
 
 
 
 Regressions 
 
  glmer  
 
 All data 
 
  null model : R 2  = 0.0%, ICC = 99.0% 
  ASPM :
 
 by itself: R 2  = 0.0%,  β  = 0.10 ± 0.56,  p  ASPM/null  = 0.86 
 quadratic: R 2  = 0.6%,  β ASPM2   = 0.37 ± 0.86,  p  ASPM2/ASPM  = 0.057 
  
  MCPH1 :
 
 by itself: R 2  = 0.0%,  β  = -0.07 ± 0.41,  p  MCPH1/null  = 0.87 
 quadratic: R 2  = 0.6%,  β MCPH12   = -0.15 ± 0.48,  p  MCPH12/MCPH1  = 0.06 
  
  both alleles  (no  macroarea ):
 
  ASPM  +  MCPH1 : R 2  = 0.0%,  β  ASPM  = 0.16 ± 0.60,  p  ASPM/MCPH1  = 0.79,  β  MCPH1  = -0.12 ± 0.45,  p  MCPH1/ASPM  = 0.8,  p  ASPM+MCPH1/null  = 0.95, 
 interaction: R 2  = 0.0%,  p  ASPM:MCPH1/ASPM+MCPH1  = 0.64 
  
 
 
 
 Randomization 
 1000 independent replications. 
 
 Regressions on 1000 permuted data. The first 3 columns show the permutation constraints (if any), how the  macroarea  is considered (if at all), and what is permuted. The next columns show the percent of the permutations that, in order, have a better AIC compared to the original fit, are significantly better than the null model (thus testing the effect of both alleles simultaneously), have a significant effect of  ASPM -D, have a smaller effect ( β ) of  ASPM -D than the original fit, and the same for  MCPH1 -D. 
 
 
 Permute within 
 Macroarea 
 Permute 
 AIC 
 Signif. 
  p   ASPM -D  
  β   ASPM -D  
  p   MCPH1 -D  
  β   MCPH1 -D  
 
 
 
 
 unrestricted 
 none 
 tone 
 0% 
 4% 
 5% 
 74% 
 6% 
 28% 
 
 
 unrestricted 
 none 
 alleles-together 
 95% 
 4% 
 6% 
 60% 
 4% 
 34% 
 
 
 unrestricted 
 none 
 alleles-independent 
 96% 
 5% 
 6% 
 62% 
 4% 
 35% 
 
 
 families 
 none 
 tone 
 74% 
 4% 
 5% 
 66% 
 3% 
 30% 
 
 
 families 
 none 
 alleles-together 
 96% 
 5% 
 4% 
 66% 
 4% 
 31% 
 
 
 families 
 none 
 alleles-independent 
 97% 
 6% 
 5% 
 68% 
 6% 
 37% 
 
 
 
 
 
 
Regressions on 1000 permuted data. Each plot shows the original result (vertical dashed black line) and the distribution of the permutations for the three possible things to be permuted (colored curves) for each combination of permutation constraints (horizontal panels) and control for  macroarea  (vertical panels) in terms of the effect size  β ;  ASPM -D is on the left and  MCPH1 -D on the right. The vertical dotted black thin line is at 0.0.
 
 
 
 
 Restricted sampling 
 
 
 
  Figure 284.    Results for 1000 restricted samplings. For  ASPM -D (left): 99.9% of βs are negative when regressing tone on  ASPM  alone (one-sided  t -test < 0:  t (999) = -86.0, mean = -0.93,  p  = 0), and 99.9% when controlling for  MCPH1  ( t (999) = -4.2, mean = -7.89,  p  = 1.4e-05). For  MCPH1 -D (right): 98.9% of βs are negative when regressing tone on  MCPH1  alone (one-sided  t -test < 0:  t (999) = -58.5, mean = -1.18,  p  = 0), and 98.5% when controlling for  ASPM  ( t (999) = -3.5, mean = -11.46,  p  = 0.00025). 
 
 
 
 
 
  brms  
 
  ASPM  only:
 
  β  = -0.14, 89%HDI = [-1.48, 1.34] 
 posterior probability  p ( β <0) = 0.57 (evidence ratio = 1.3),  p ( β =0) = 0.8 (evidence ratio = 3.9) 
 ROPE = [-0.18, 0.18], % HDI inside ROPE = 19.2%;  p  ROPE  = 0.171 
 comparison ‘null’ vs ‘ASPM’: [B> L= W=(54%:46%) K=]: moderate evidence for null against ASPM (BF=3.77), LOO=0.25 [SE=0.63], WAIC=0.14 [SE=0.32], KFOLD=2.38 [SE=3.34] 
 comparison ‘null’ vs ‘ASPM’: [B> L= W=(54%:46%) K=]: moderate evidence for null against ASPM (BF=3.77), LOO=0.25 [SE=0.63], WAIC=0.14 [SE=0.32], KFOLD=2.38 [SE=3.34] 
  
  MCPH1  only:
 
  β  = -0.04, 89%HDI = [-1.16, 1.19] 
 posterior probability  p ( β <0) = 0.55 (evidence ratio = 1.2),  p ( β =0) = 0.83 (evidence ratio = 4.8) 
 ROPE = [-0.18, 0.18], % HDI inside ROPE = 24%;  p  ROPE  = 0.214 
 comparison ‘null’ vs ‘MCPH1’: [B> L= W=(51%:49%) K=]: moderate evidence for null against MCPH1 (BF=4.7), LOO=0.22 [SE=0.55], WAIC=0.06 [SE=0.28], KFOLD=-1.06 [SE=2.93] 
 comparison ‘null’ vs ‘MCPH1’: [B> L= W=(51%:49%) K=]: moderate evidence for null against MCPH1 (BF=4.7), LOO=0.22 [SE=0.55], WAIC=0.06 [SE=0.28], KFOLD=-1.06 [SE=2.93] 
  
  both alleles :
 
 comparison ‘null’ vs ‘both’: [B>> L= W=(50%:50%) K=]: strong evidence for null against both (BF=18.5), LOO=0.90 [SE=0.93], WAIC=0.00 [SE=0.43], KFOLD=-1.63 [SE=1.82] 
 interaction:
 
 posterior probability  p (=0) = 0.8 (evidence ratio = 3.9) 
 ROPE = [-0.18, 0.18], % HDI inside ROPE = 18.6%;  p  ROPE  = 0.166 
 comparison ‘no interaction’ vs ‘with interaction’: [B> L= W=(48%:52%) K>]: moderate evidence for no interaction against with interaction (BF=3.76), LOO=0.55 [SE=0.60], WAIC=-0.07 [SE=0.38], KFOLD=4.85 [SE=2.79] 
  
  ASPM  (partial):
 
  β  = -0.11, 89%HDI = [-1.68, 1.37] 
 posterior probability  p ( β <0) = 0.54 (evidence ratio = 1.2),  p ( β =0) = 0.78 (evidence ratio = 3.6) 
 ROPE = [-0.18, 0.18], % HDI inside ROPE = 18.3%;  p  ROPE  = 0.163 
  
  MCPH1  (partial):
 
  β  = -0.06, 89%HDI = [-1.28, 1.29] 
 posterior probability  p ( β <0) = 0.54 (evidence ratio = 1.2),  p ( β =0) = 0.82 (evidence ratio = 4.5) 
 ROPE = [-0.18, 0.18], % HDI inside ROPE = 22.6%;  p  ROPE  = 0.202 
  
  
 
 
  
 
  Figure 285.    Posterior distributions (with 50% probability mass highlighted) versus 0.0 (the vertical line) for  ASPM -D (left) and  MCPH1 -D (right). 
 
 
 
  
 
  Figure 286.    Conditional effects of  ASPM -D (left) and  MCPH1 -D (right). 
 
 
 
  
 
  Figure 287.    Posterior predictive checks for  ASPM -D (left) and  MCPH1 -D (right). 
 
 
 
  
 
  Figure 288.    Confusion matrices for  ASPM -D (left) and  MCPH1 -D (right). 
 
 
 
 
 
 
  tone2  
 The resulting dataset has 123 observations, distributed among 71 unique Glottolg codes in 19 families (ranging from a minimum of 1 language per family to a maximum of 47, with a mean 6.5 and median 3 languages per family) and 1 macroareas. 
 There are 115:86:71 unique samples:(meta)populations:languages retained. 
 
 
 
 
 
 
 
 
 
 
 
   
 Africa 
 Eurasia 
 America 
 Papunesia 
 Sum 
 
 
 
 
  No  
 0 
 105 
 0 
 0 
 105 
 
 
  Yes  
 0 
 18 
 0 
 0 
 18 
 
 
  Sum  
 0 
 123 
 0 
 0 
 123 
 
 
 
 
 
 
  Figure 289.    Distribution of  tone2 . 
 
 
 
 
 
  Figure 290.    Map of  tone2 . 
 
 
 
 
 
  Figure 291.    Relationship between  tone2 ,  ASPM -D and  MCPH1 -D. 
 
 
 
 Regressions 
 
  glmer  
 
 All data 
 
  null model : R 2  = 0.0%, ICC = 98.7% 
  ASPM :
 
 by itself: R 2  = 0.0%,  β  = 0.03 ± 1.46,  p  ASPM/null  = 1 
 quadratic: R 2  = 81.7%,  β ASPM2   = -191.71 ± 77.88,  p  ASPM2/ASPM  = 4e-05 
  
  MCPH1 :
 
 by itself: R 2  = 0.1%,  β  = -0.63 ± 1.12,  p  MCPH1/null  = 0.6 
 quadratic: R 2  = 0.4%,  β MCPH12   = -0.42 ± 1.31,  p  MCPH12/MCPH1  = 0.25 
  
  both alleles  (no  macroarea ):
 
  ASPM  +  MCPH1 : R 2  = 0.2%,  β  ASPM  = -0.02 ± 1.39,  p  ASPM/MCPH1  = 1,  β  MCPH1  = -0.63 ± 1.11,  p  MCPH1/ASPM  = 0.6,  p  ASPM+MCPH1/null  = 0.87, 
 interaction: R 2  = 0.3%,  p  ASPM:MCPH1/ASPM+MCPH1  = 0.72 
  
 
 
 
 Randomization 
 
 Regressions with randomizations for  tone2 . 
 
 
 Permute within 
 Macroarea 
 Permute 
 AIC 
 Signif. 
  p   ASPM -D  
  β   ASPM -D  
  p   MCPH1 -D  
  β   MCPH1 -D  
 
 
 
 
 unrestricted 
 none 
 tone 
 0% 
 5% 
 6% 
 44% 
 5% 
 0% 
 
 
 unrestricted 
 none 
 alleles-together 
 90% 
 12% 
 12% 
 48% 
 10% 
 27% 
 
 
 unrestricted 
 none 
 alleles-independent 
 88% 
 12% 
 12% 
 49% 
 10% 
 28% 
 
 
 families 
 none 
 tone 
 49% 
 9% 
 10% 
 62% 
 4% 
 19% 
 
 
 families 
 none 
 alleles-together 
 80% 
 8% 
 10% 
 60% 
 4% 
 16% 
 
 
 families 
 none 
 alleles-independent 
 84% 
 10% 
 12% 
 68% 
 6% 
 19% 
 
 
 
 
 
 
Regressions on 1000 permuted data. Each plot shows the original result (vertical dashed black line) and the distribution of the permutations for the three possible things to be permuted (colored curves) for each combination of permutation constraints (horizontal panels) and control for  macroarea  (vertical panels) in terms of the effect size  β ;  ASPM -D is on the left and  MCPH1 -D on the right. The vertical dotted black thin line is at 0.0.
 
 
 
 
 Restricted sampling 
 
 
 
  Figure 292.    Results for 1000 restricted samplings. For  ASPM -D (left): 99.9% of βs are negative when regressing tone on  ASPM  alone (one-sided  t -test < 0:  t (999) = -72.3, mean = -1.10,  p  = 0) and 99.8% when controlling for  MCPH1  ( t (999) = -65.0, mean = -1.31,  p  = 0). For  MCPH1 -D (right): 75.3% of βs are negative when regressing tone on  MCPH1  alone (one-sided  t -test < 0:  t (999) = -20.5, mean = -0.38,  p  = 9.5e-79) and 76.2% when controlling for  ASPM  ( t (999) = -21.1, mean = -0.64,  p  = 2.1e-82). 
 
 
 
 
 
  brms  
 
  ASPM  only:
 
  β  = -1.44, 89%HDI = [-3.63, 1.13] 
 posterior probability  p ( β <0) = 0.84 (evidence ratio = 5.4),  p ( β =0) = 0.58 (evidence ratio = 1.4) 
 ROPE = [-0.18, 0.18], % HDI inside ROPE = 6.9%;  p  ROPE  = 0.062 
 comparison ‘null’ vs ‘ASPM’: [B= L= W=(47%:53%) K>]: anecdotal evidence for null against ASPM (BF=1.49), LOO=-0.11 [SE=0.84], WAIC=-0.13 [SE=0.66], KFOLD=2.88 [SE=2.04] 
 comparison ‘null’ vs ‘ASPM’: [B= L= W=(47%:53%) K>]: anecdotal evidence for null against ASPM (BF=1.49), LOO=-0.11 [SE=0.84], WAIC=-0.13 [SE=0.66], KFOLD=2.88 [SE=2.04] 
  
  MCPH1  only:
 
  β  = -0.42, 89%HDI = [-2.51, 1.61] 
 posterior probability  p ( β <0) = 0.64 (evidence ratio = 1.8),  p ( β =0) = 0.72 (evidence ratio = 2.5) 
 ROPE = [-0.18, 0.18], % HDI inside ROPE = 12.8%;  p  ROPE  = 0.114 
 comparison ‘null’ vs ‘MCPH1’: [B= L= W<(45%:55%) K=]: anecdotal evidence for null against MCPH1 (BF=2.44), LOO=0.09 [SE=0.28], WAIC=-0.21 [SE=0.19], KFOLD=0.33 [SE=0.47] 
 comparison ‘null’ vs ‘MCPH1’: [B= L= W<(45%:55%) K=]: anecdotal evidence for null against MCPH1 (BF=2.44), LOO=0.09 [SE=0.28], WAIC=-0.21 [SE=0.19], KFOLD=0.33 [SE=0.47] 
  
  both alleles :
 
 comparison ‘null’ vs ‘both’: [B> L< W<(31%:69%) K>]: moderate evidence for null against both (BF=3.62), LOO=-0.77 [SE=0.75], WAIC=-0.78 [SE=0.67], KFOLD=5.80 [SE=3.74] 
 interaction:
 
 posterior probability  p (=0) = 0.71 (evidence ratio = 2.5) 
 ROPE = [-0.18, 0.18], % HDI inside ROPE = 11.8%;  p  ROPE  = 0.105 
 comparison ‘no interaction’ vs ‘with interaction’: [B= L>> W<(47%:53%) K<]: anecdotal evidence for no interaction against with interaction (BF=2.31), LOO=0.47 [SE=0.24], WAIC=-0.12 [SE=0.09], KFOLD=-5.61 [SE=3.74] 
  
  ASPM  (partial):
 
  β  = -1.44, 89%HDI = [-4.09, 1.20] 
 posterior probability  p ( β <0) = 0.82 (evidence ratio = 4.6),  p ( β =0) = 0.59 (evidence ratio = 1.5) 
 ROPE = [-0.18, 0.18], % HDI inside ROPE = 6.8%;  p  ROPE  = 0.06 
  
  MCPH1  (partial):
 
  β  = -0.52, 89%HDI = [-2.53, 1.68] 
 posterior probability  p ( β <0) = 0.66 (evidence ratio = 2),  p ( β =0) = 0.72 (evidence ratio = 2.5) 
 ROPE = [-0.18, 0.18], % HDI inside ROPE = 12.3%;  p  ROPE  = 0.11 
  
  
 
 
  
 
  Figure 293.    Posterior distributions (with 50% probability mass highlighted) versus 0.0 (the vertical line) for  ASPM -D (left) and  MCPH1 -D (right). 
 
 
 
  
 
  Figure 294.    Conditional effects of  ASPM -D (left) and  MCPH1 -D (right). 
 
 
 
  
 
  Figure 295.    Posterior predictive checks for  ASPM -D (left) and  MCPH1 -D (right). 
 
 
 
  
 
  Figure 296.    Confusion matrices for  ASPM -D (left) and  MCPH1 -D (right). 
 
 
 
 
 
 
 Tone  counts  
 The resulting dataset has 126 observations, distributed among 73 unique Glottolg codes in 19 families (ranging from a minimum of 1 language per family to a maximum of 47, with a mean 6.6 and median 3 languages per family) and 1 macroareas. 
 There are 115:86:73 unique samples:(meta)populations:languages retained. 
 
 
 
 
 
 
 
 
 
 
 
   
 Africa 
 Eurasia 
 America 
 Papunesia 
 Sum 
 
 
 
 
  0  
 0 
 98 
 0 
 0 
 98 
 
 
  1  
 0 
 6 
 0 
 0 
 6 
 
 
  2  
 0 
 3 
 0 
 0 
 3 
 
 
  3  
 0 
 5 
 0 
 0 
 5 
 
 
  4  
 0 
 8 
 0 
 0 
 8 
 
 
  5  
 0 
 4 
 0 
 0 
 4 
 
 
  6  
 0 
 2 
 0 
 0 
 2 
 
 
  Sum  
 0 
 126 
 0 
 0 
 126 
 
 
 
 
 
 
  Figure 297.    Distribution of tone  counts . 
 
 
 
 
 
  Figure 298.    Distribution of tone  counts  across the world. 
 
 
 
 
 
  Figure 299.    Relationship between tone  counts  (colors) and the two alleles (frequency) by macroarea. 
 
 
 
 Regressions 
 
  glmer  
 
 All data 
 
  null model : R 2  = 0.0%, ICC = 100.0% 
 the Poisson model is  not  overdispersed:  χ  2 (124) = 64.3,  p  = 1 
  ASPM :
 
 by itself: R 2  = 0.0%,  β  = 0.05 ± 0.27,  p  ASPM/null  = 0.84 
 quadratic: R 2  = 27.2%,  β ASPM2   = -1.02 ± 0.60,  p  ASPM2/ASPM  = 0.013 
  
  MCPH1 :
 
 by itself: R 2  = 0.4%,  β  = -0.20 ± 0.17,  p  MCPH1/null  = 0.26 
 quadratic: R 2  = 0.8%,  β MCPH12   = -0.15 ± 0.18,  p  MCPH12/MCPH1  = 0.15 
  
  both alleles  (no  macroarea ):
 
  ASPM  +  MCPH1 : R 2  = 0.4%,  β  ASPM  = 0.00 ± 0.26,  p  ASPM/MCPH1  = 0.99,  β  MCPH1  = -0.20 ± 0.17,  p  MCPH1/ASPM  = 0.27,  p  ASPM+MCPH1/null  = 0.53, 
 interaction: R 2  = 0.5%,  p  ASPM:MCPH1/ASPM+MCPH1  = 0.64 
  
 
 
 
 Randomization 
 We performed 1000 independent replications: 
 
 Regressions with randomizations for tone  counts . 
 
 
 Permute within 
 Macroarea 
 Permute 
 AIC 
 Signif. 
  p   ASPM -D  
  β   ASPM -D  
  p   MCPH1 -D  
  β   MCPH1 -D  
 
 
 
 
 unrestricted 
 none 
 tone 
 0% 
 37% 
 25% 
 47% 
 26% 
 16% 
 
 
 unrestricted 
 none 
 alleles-together 
 46% 
 3% 
 3% 
 50% 
 3% 
 4% 
 
 
 unrestricted 
 none 
 alleles-independent 
 48% 
 2% 
 3% 
 52% 
 3% 
 4% 
 
 
 families 
 none 
 tone 
 47% 
 5% 
 11% 
 74% 
 1% 
 10% 
 
 
 families 
 none 
 alleles-together 
 49% 
 5% 
 10% 
 74% 
 1% 
 9% 
 
 
 families 
 none 
 alleles-independent 
 47% 
 4% 
 8% 
 70% 
 1% 
 7% 
 
 
 
 
 
 
Regressions on 1000 permuted data. Each plot shows the original result (vertical dashed black line) and the distribution of the permutations for the three possible things to be permuted (colored curves) for each combination of permutation constraints (horizontal panels) and control for  macroarea  (vertical panels) in terms of the effect size  β ;  ASPM -D is on the left and  MCPH1 -D on the right. The vertical dotted black thin line is at 0.0.
 
 
 
 
 Restricted sampling 
 
 
 
  Figure 300.    Results for 1000 restricted samplings. For  ASPM -D (left): 99.9% of βs are negative when regressing tone on  ASPM  alone (one-sided  t -test < 0:  t (999) = -86.3, mean = -0.69,  p  = 0) and 99.9% when controlling for  MCPH1  ( t (999) = -88.2, mean = -0.76,  p  = 0). For  MCPH1 -D (right): 62.9% of βs are negative when regressing tone on  MCPH1  alone (one-sided  t -test < 0:  t (999) = -9.3, mean = -0.11,  p  = 4.3e-20) and 63.7% when controlling for  ASPM  ( t (999) = -7.7, mean = -0.13,  p  = 1.7e-14). 
 
 
 
 
 
  brms  
 
  ASPM  only:
 
  β  = -0.19, 89%HDI = [-0.77, 0.37] 
 posterior probability  p ( β <0) = 0.7 (evidence ratio = 2.4),  p ( β =0) = 0.89 (evidence ratio = 8.2) 
 ROPE = [-0.10, 0.10], % HDI inside ROPE = 20.9%;  p  ROPE  = 0.186 
 comparison ‘null’ vs ‘ASPM’: [B> L>> W>>(75%:25%) K=]: moderate evidence for null against ASPM (BF=9.85), LOO=1.67 [SE=0.55], WAIC=1.09 [SE=0.41], KFOLD=0.57 [SE=1.33] 
 comparison ‘null’ vs ‘ASPM’: [B> L>> W>>(75%:25%) K=]: moderate evidence for null against ASPM (BF=9.85), LOO=1.67 [SE=0.55], WAIC=1.09 [SE=0.41], KFOLD=0.57 [SE=1.33] 
  
  MCPH1  only:
 
  β  = -0.1, 89%HDI = [-0.44, 0.23] 
 posterior probability  p ( β <0) = 0.69 (evidence ratio = 2.2),  p ( β =0) = 0.93 (evidence ratio = 13) 
 ROPE = [-0.10, 0.10], % HDI inside ROPE = 37%;  p  ROPE  = 0.329 
 comparison ‘null’ vs ‘MCPH1’: [B>> L>> W>>(79%:21%) K=]: strong evidence for null against MCPH1 (BF=16.8), LOO=1.75 [SE=0.48], WAIC=1.30 [SE=0.38], KFOLD=-0.32 [SE=1.41] 
 comparison ‘null’ vs ‘MCPH1’: [B>> L>> W>>(79%:21%) K=]: strong evidence for null against MCPH1 (BF=16.8), LOO=1.75 [SE=0.48], WAIC=1.30 [SE=0.38], KFOLD=-0.32 [SE=1.41] 
  
  both alleles :
 
 comparison ‘null’ vs ‘both’: [B>> L>> W>>(85%:15%) K=]: very strong evidence for null against both (BF=92.8), LOO=2.74 [SE=0.92], WAIC=1.75 [SE=0.66], KFOLD=-0.19 [SE=1.57] 
 interaction:
 
 posterior probability  p (=0) = 0.9 (evidence ratio = 9.3) 
 ROPE = [-0.10, 0.10], % HDI inside ROPE = 24.8%;  p  ROPE  = 0.221 
 comparison ‘no interaction’ vs ‘with interaction’: [B>> L= W=(52%:48%) K>>]: strong evidence for no interaction against with interaction (BF=10.4), LOO=-0.14 [SE=0.62], WAIC=0.09 [SE=0.31], KFOLD=3.56 [SE=1.62] 
  
  ASPM  (partial):
 
  β  = -0.21, 89%HDI = [-0.79, 0.36] 
 posterior probability  p ( β <0) = 0.72 (evidence ratio = 2.5),  p ( β =0) = 0.89 (evidence ratio = 8) 
 ROPE = [-0.10, 0.10], % HDI inside ROPE = 21.6%;  p  ROPE  = 0.192 
  
  MCPH1  (partial):
 
  β  = -0.12, 89%HDI = [-0.49, 0.19] 
 posterior probability  p ( β <0) = 0.71 (evidence ratio = 2.5),  p ( β =0) = 0.93 (evidence ratio = 13) 
 ROPE = [-0.10, 0.10], % HDI inside ROPE = 34.4%;  p  ROPE  = 0.306 
  
  
 
 
  
 
  Figure 301.    Posterior distributions (with 50% probability mass highlighted) versus 0.0 (the vertical line) for  ASPM -D (left) and  MCPH1 -D (right). 
 
 
 
  
 
  Figure 302.    Conditional effects of  ASPM -D (left) and  MCPH1 -D (right). 
 
 
 
  
 
  Figure 303.    Posterior predictive checks for  ASPM -D (left) and  MCPH1 -D (right). 
 
 
 
 
 
 
 
 Only America 
 Too little data… 
 
 
 Only Papunesia 
 Too little data… 
 
 
 
 Appendix VII: Draw diagrams for paper 
 
 Generic mediation model with a single mediator 
  
 
 
 
 Generic mediation model with a two mediators or a path analysis 
  
 
 
 
  tone1 : simultaneous mediation through both alleles 
  
 
 
 
  tone1 : path analysis (numeric coding) 
  
 
 
 
  tone1 : path analysis (restricted sampling) 
  
 
 
 
  tone2 : simultaneous mediation through both alleles 
  
 
 
 
  tone2 : path analysis (numeric coding) 
  
 
 
 
  tone2 : path analysis (restricted sampling) 
  
 
 
 
 Tone  counts : simultaneous mediation through both alleles 
  
 
 
 
 Tone  counts : path analysis 
  
 
 
 
 Tone  counts : path analysis (restricted sampling) 
  
 
 
 
 
 Session information 
  CPU:  AMD Ryzen 7 3700X 8-Core Processor (16 threads) 
  RAM (memory):  67.5 GB 
  R version 4.0.5 (2021-03-31)  
  Platform:  x86_64-pc-linux-gnu (64-bit) 
  locale:   LC_CTYPE=en_US.UTF-8 ,  LC_NUMERIC=C ,  LC_TIME=en_US.UTF-8 ,  LC_COLLATE=en_US.UTF-8 ,  LC_MONETARY=en_US.UTF-8 ,  LC_MESSAGES=en_US.UTF-8 ,  LC_PAPER=en_US.UTF-8 ,  LC_NAME=C ,  LC_ADDRESS=C ,  LC_TELEPHONE=C ,  LC_MEASUREMENT=en_US.UTF-8  and  LC_IDENTIFICATION=C  
  attached base packages:   grid ,  stats ,  graphics ,  grDevices ,  utils ,  datasets ,  methods  and  base  
  other attached packages:   benchmarkme(v.1.0.7) ,  magick(v.2.7.2) ,  pdftools(v.3.0.1) ,  rsvg(v.2.1.2) ,  DiagrammeRsvg(v.0.1) ,  simr(v.1.0.5) ,  phytools(v.0.7-70) ,  ape(v.5.5) ,  tidybayes(v.2.3.1) ,  bayestestR(v.0.9.0) ,  brms(v.2.15.0) ,  Rcpp(v.1.0.6) ,  dagitty(v.0.3-1) ,  e1071(v.1.7-6) ,  cowplot(v.1.1.1) ,  maps(v.3.3.0) ,  lavaanPlot(v.0.5.1) ,  lavaan(v.0.6-8) ,  randomForest(v.4.6-14) ,  caret(v.6.0-86) ,  lattice(v.0.20-44) ,  partykit(v.1.2-13) ,  libcoin(v.1.0-8) ,  rsample(v.0.1.0) ,  mediation(v.4.5.0) ,  sandwich(v.3.0-0) ,  mvtnorm(v.1.1-1) ,  MASS(v.7.3-54) ,  DiagrammeR(v.1.0.6.1) ,  pbapply(v.1.4-3) ,  sjPlot(v.2.8.7) ,  ggnewscale(v.0.4.5) ,  glmmTMB(v.1.0.2.1) ,  data.table(v.1.14.0) ,  reshape2(v.1.4.4) ,  dplyr(v.1.0.6) ,  lmerTest(v.3.1-3) ,  lme4(v.1.1-26) ,  Matrix(v.1.3-3) ,  performance(v.0.7.1) ,  png(v.0.1-7) ,  jpeg(v.0.1-8.1) ,  tiff(v.0.1-8) ,  gridExtra(v.2.3) ,  ggplot2(v.3.3.3) ,  stringr(v.1.4.0) ,  pander(v.0.6.3) ,  knitr(v.1.33)  and  RhpcBLASctl(v.0.20-137)  
  loaded via a namespace (and not attached):   estimability(v.1.3) ,  ModelMetrics(v.1.2.2.2) ,  coda(v.0.19-4) ,  tidyr(v.1.1.3) ,  clusterGeneration(v.1.3.7) ,  dygraphs(v.1.1.1.6) ,  rpart(v.4.1-15) ,  inline(v.0.3.17) ,  doParallel(v.1.0.16) ,  generics(v.0.1.0) ,  callr(v.3.7.0) ,  combinat(v.0.0-8) ,  proxy(v.0.4-25) ,  future(v.1.21.0) ,  RLRsim(v.3.1-6) ,  lubridate(v.1.7.10) ,  httpuv(v.1.6.1) ,  StanHeaders(v.2.21.0-7) ,  assertthat(v.0.2.1) ,  gower(v.0.2.2) ,  xfun(v.0.22) ,  hms(v.1.0.0) ,  ggdist(v.2.4.0) ,  jquerylib(v.0.1.4) ,  bayesplot(v.1.8.0) ,  evaluate(v.0.14) ,  promises(v.1.2.0.1) ,  fansi(v.0.4.2) ,  readxl(v.1.3.1) ,  igraph(v.1.2.6) ,  DBI(v.1.1.1) ,  tmvnsim(v.1.0-2) ,  htmlwidgets(v.1.5.3) ,  stats4(v.4.0.5) ,  benchmarkmeData(v.1.0.4) ,  purrr(v.0.3.4) ,  ellipsis(v.0.3.2) ,  crosstalk(v.1.1.1) ,  backports(v.1.2.1) ,  binom(v.1.1-1) ,  V8(v.3.4.2) ,  pbivnorm(v.0.6.0) ,  insight(v.0.14.0) ,  markdown(v.1.1) ,  RcppParallel(v.5.1.4) ,  vctrs(v.0.3.8) ,  sjlabelled(v.1.1.8) ,  abind(v.1.4-5) ,  withr(v.2.4.2) ,  checkmate(v.2.0.0) ,  emmeans(v.1.6.0) ,  xts(v.0.12.1) ,  prettyunits(v.1.1.1) ,  mnormt(v.2.0.2) ,  cluster(v.2.1.2) ,  crayon(v.1.4.1) ,  recipes(v.0.1.16) ,  pkgconfig(v.2.0.3) ,  nlme(v.3.1-152) ,  nnet(v.7.3-16) ,  rlang(v.0.4.11) ,  globals(v.0.14.0) ,  lifecycle(v.1.0.0) ,  miniUI(v.0.1.1.1) ,  colourpicker(v.1.1.0) ,  modelr(v.0.1.8) ,  cellranger(v.1.1.0) ,  distributional(v.0.2.2) ,  matrixStats(v.0.58.0) ,  phangorn(v.2.7.0) ,  loo(v.2.4.1) ,  carData(v.3.0-4) ,  boot(v.1.3-28) ,  zoo(v.1.8-9) ,  base64enc(v.0.1-3) ,  gamm4(v.0.2-6) ,  ggridges(v.0.5.3) ,  processx(v.3.5.2) ,  parameters(v.0.13.0) ,  visNetwork(v.2.0.9) ,  pROC(v.1.17.0.1) ,  parallelly(v.1.25.0) ,  qpdf(v.1.1) ,  shinystan(v.2.5.0) ,  ggeffects(v.1.1.0) ,  scales(v.1.1.1) ,  lpSolve(v.5.6.15) ,  magrittr(v.2.0.1) ,  plyr(v.1.8.6) ,  threejs(v.0.3.3) ,  compiler(v.4.0.5) ,  rstantools(v.2.1.1) ,  RColorBrewer(v.1.1-2) ,  plotrix(v.3.8-1) ,  cli(v.2.5.0) ,  listenv(v.0.8.0) ,  ps(v.1.6.0) ,  TMB(v.1.7.20) ,  Brobdingnag(v.1.2-6) ,  htmlTable(v.2.1.0) ,  Formula(v.1.2-4) ,  mgcv(v.1.8-35) ,  tidyselect(v.1.1.1) ,  stringi(v.1.6.1) ,  forcats(v.0.5.1) ,  projpred(v.2.0.2) ,  yaml(v.2.2.1) ,  askpass(v.1.1) ,  svUnit(v.1.0.6) ,  latticeExtra(v.0.6-29) ,  bridgesampling(v.1.1-2) ,  sass(v.0.4.0) ,  fastmatch(v.1.1-0) ,  tools(v.4.0.5) ,  rio(v.0.5.26) ,  parallel(v.4.0.5) ,  rstudioapi(v.0.13) ,  foreach(v.1.5.1) ,  foreign(v.0.8-81) ,  inum(v.1.0-4) ,  prodlim(v.2019.11.13) ,  scatterplot3d(v.0.3-41) ,  farver(v.2.1.0) ,  digest(v.0.6.27) ,  shiny(v.1.6.0) ,  lava(v.1.6.9) ,  quadprog(v.1.5-8) ,  car(v.3.0-10) ,  broom(v.0.7.6) ,  later(v.1.2.0) ,  httr(v.1.4.2) ,  rsconnect(v.0.8.17) ,  effectsize(v.0.4.4-1) ,  sjstats(v.0.18.1) ,  colorspace(v.2.0-1) ,  splines(v.4.0.5) ,  statmod(v.1.4.36) ,  expm(v.0.999-6) ,  shinythemes(v.1.2.0) ,  xtable(v.1.8-4) ,  jsonlite(v.1.7.2) ,  nloptr(v.1.2.2.2) ,  timeDate(v.3043.102) ,  rstan(v.2.21.2) ,  ipred(v.0.9-11) ,  R6(v.2.5.0) ,  Hmisc(v.4.5-0) ,  pillar(v.1.6.0) ,  htmltools(v.0.5.1.1) ,  mime(v.0.10) ,  glue(v.1.4.2) ,  fastmap(v.1.1.0) ,  minqa(v.1.2.4) ,  DT(v.0.18) ,  class(v.7.3-19) ,  codetools(v.0.2-18) ,  pkgbuild(v.1.2.0) ,  furrr(v.0.2.2) ,  utf8(v.1.2.1) ,  bslib(v.0.2.5) ,  tibble(v.3.1.1) ,  pbkrtest(v.0.5.1) ,  numDeriv(v.2016.8-1.1) ,  arrayhelpers(v.1.1-0) ,  curl(v.4.3.1) ,  gtools(v.3.8.2) ,  zip(v.2.1.1) ,  openxlsx(v.4.2.3) ,  shinyjs(v.2.0.0) ,  survival(v.3.2-11) ,  rmarkdown(v.2.8) ,  munsell(v.0.5.0) ,  iterators(v.1.0.13) ,  haven(v.2.4.1) ,  sjmisc(v.2.8.7)  and  gtable(v.0.3.0)  
 
 
 References 
 
 
Dediu, D., & Ladd, D. R. (2007). Linguistic tone is related to the population frequency of the adaptive haplogroups of two brain size genes,  ASPM  and  Microcephalin .  Proc Natl Acad Sci U S A ,  104 (26), 10944–10949.  https://doi.org/10.1073/pnas.0610848104 
 
 
Evans, P. D., Gilbert, S. L., Mekel-Bobrov, N., Vallender, E. J., Anderson, J. R., Vaez-Azizi, L. M., … Lahn, B. T. (2005). Microcephalin, a gene regulating brain size, continues to evolve adaptively in humans.  Science ,  309 (5741), 1717–1720.  https://doi.org/10.1126/science.1113722 
 
 
McElreath, R. (2020).  Statistical  Rethinking :  A   Bayesian   Course  with  Examples  in  R  and  Stan   (2nd ed.). CRC Press LLC.
 
 
Mekel-Bobrov, N., Gilbert, S. L., Evans, P. D., Vallender, E. J., Anderson, J. R., Hudson, R. R., … Lahn, B. T. (2005). Ongoing adaptive evolution of  ASPM , a brain size determinant in  Homo  sapiens.  Science ,  309 (5741), 1720–1722.  https://doi.org/10.1126/science.1116815 
 
 
Wong, Patrick C. M., Chandrasekaran, B., & Zheng, J. (2012). The  Derived   Allele  of  ASPM   Is   Associated  with  Lexical   Tone   Perception .  PLoS One ,  7 (4), e34243.  https://doi.org/10.1371/journal.pone.0034243 
 
 
Wong, Patrick C. M., Kang, X., Wong, K. H. Y., So, H.-C., Choy, K. W., & Geng, X. (2020).  ASPM -lexical tone association in speakers of a tone language:  Direct  evidence for the genetic-biasing hypothesis of language evolution.  Science Advances ,  6 (22), eaba5090.  https://doi.org/10.1126/sciadv.aba5090 
 
 
 
 
 
 
  For mixed-effects models, this is Nakagawa’s R 2  estimate, where the  marginal  estimate considers only the fixed effects, while the  conditional  also considers the random effects as well. Here, we show only the marginal ICC, as we are interested in the fixed effects. See  ?performance::r2  for more details. ↩︎   
  ICC represents the proportion of the variance explained by the grouping due to the random effects, and varies between 0% (the grouping contains no info) to 100% (basically all individual observations in a given group are identical); the  adjusted  ICC only considers the random effect, while the  conditional  ICC also considers the fixed effects as well; they are equal when there are no fixed effects (i.e., for the null models). Here, we show only the adjusted ICC, as we are interested in the random effects. See  ?performance::icc  for more details. ↩︎   
  Here I use model comparisons to estimate the  p -value of adding (or removing) a predictor,  v , by comparing the model without the predictor ( m ) with the model with the predictor ( m_v ),  anova(m, m_v)  and report the  p -value denoted as  p  v/m  to make clear what predictor is added to which model. ↩︎   
  The ROPE (region of practical equivalence) is a small interval around 0.0 (usually, [-0.1, 0.1] but can vary depending on the particular model). Can be used either to estimate the percent of the HDI that falls within this interval, or the proportion of the whole posterior distribution that does so. It can be used in a manner similar to that of frequentist  p -values to judge if 0.0 can be ruled out as a probable value of the parameter of interest. ↩︎   
  This compares two  brms  models, m1 and m2, using Bayes Factors (BF), LOO, WAIC and KFOLD. For the latter three, I show the difference between m1 and m2 (in this order), and the SE of this difference; if the difference is  negative  (<0) then  m1 is worse , while if it is  positive  (>0)  m1 is better , but the “significance” of this difference can be interpreted only in the context of the SE. These results are summarized using the [B? L? W?(x%:y%) K?] notation, where the symbol * can be “=” when the models are pretty much  equivalent , “<” if m1 is  worse  than m2 (and “<<” if this difference is really big), or “>” if m1 is  better  than m2 (and “>>” if this difference is really big); for WAIC (“W”) I also give the relative weights of the two models as (x%:y%). ↩︎   
  Please note that for path analyses/SEM models, we want the goodness-of-fit  χ  2  test to be  non-significant , meaning that there is no reason to reject the hypothesis that the model fits the data. On the other hand, there is a plethora of goodness of fit indices (we show a few) where the idea is that the closer they are to 1.00 the better the model fits to the data. ↩︎   
 
 


 
 

 

 

 

 

 

 

 
 

 
 
